# Nickel-Catalyzed Cross-Coupling of Aryl Chlorides by Heated Mechanochemistry: Scalable Suzuki–Miyaura Reactions via Twin-Screw Extrusion

**DOI:** 10.1021/jacsau.5c00934

**Published:** 2025-12-01

**Authors:** Sarah E. Raby-Buck, Renan R. Mattioli, Robert R. A. Bolt, Katharine Ingram, Julio C. Pastre, Duncan L. Browne

**Affiliations:** † Department of Pharmaceutical and Biological Chemistry, 4919 University College London, School of Pharmacy, 29-39 Brunswick Square, Bloomsbury, London W1CN 1AX, U.K.; ‡ Institute of Chemistry, 28132 State University of Campinas (UNICAMP), 13083-970 Campinas, SP Brazil; § Syngenta, Jealott’s Hill International Research Centre, Bracknell, Berkshire RG42 6EY, U.K.

**Keywords:** mechanochemistry, cross-coupling, nickel catalysis, twin-screw extrusion, aryl chlorides

## Abstract

The direct use of aryl chlorides in Suzuki–Miyaura cross-coupling remains a long-standing challenge due to the inert nature of the C–Cl bond. Herein, we report the first nickel-catalyzed Suzuki–Miyaura cross-coupling of aryl chlorides under solvent-minimized, heated mechanochemical conditions. Employing liquid-assisted grinding (LAG) and thermal input, a broad range of electron-deficient and electron-rich aryl chlorides were successfully coupled with aryl boronic acids in under 1 h. The methodology was translated to a twin-screw extrusion (TSE) process, enabling continuous production at scales up to 400 mmol and 65 g isolated product. This work demonstrates a sustainable, scalable strategy for C–C bond formation using readily available feedstocks, highlighting the synergy between nickel catalysis, mechanochemistry, and continuous flow processing.

## Graphical abstract

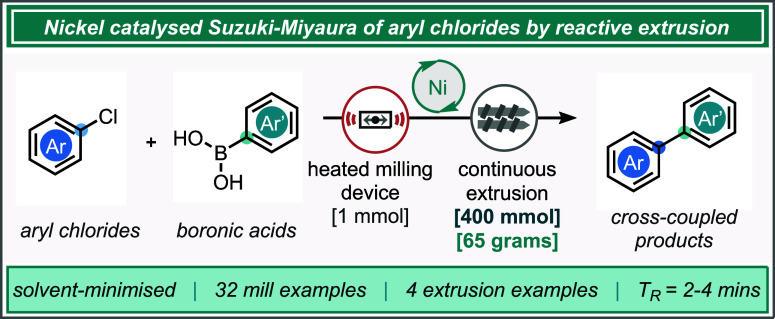

## Introduction

The Suzuki–Miyaura cross-coupling reaction remains among the most widely utilized C­(sp^2^)–C­(sp^2^) bond-forming processes in contemporary organic synthesis. Typically palladium-catalyzed, the coupling of boronic acids with aryl (pseudo)­halides offers a robust and functionally tolerant methodology under relatively mild conditions, rendering it well-suited to late-stage derivatization.^1^ Owing to its versatility, the transformation is frequently adopted as a benchmark reaction for evaluating alternative reactor technologies, including continuous flow,^2^ microwave irradiation,^3^ and, more recently, mechanochemical approaches.^4^

Interest in mechanochemical synthesis has not only centered on its capacity to eliminate bulk solvent and reduce environmental impact,^5–7^ but also studies have highlighted broader advantages. These include reduced reaction times, enhanced chemoselectivity, and improved handling of air- and moisture-sensitive reagents.^6–9^ Our own efforts in this area have focused on expanding the utility of solvent-minimized ball-milling protocols and translating these methods to reactive extrusion, where solvent reduction can yield operational and environmental gains at scale.^10–12^

The evolution of solution-phase Suzuki–Miyaura (S–M) cross-coupling has been elegantly conceptualized by Snieckus and co-workers as a series of research “waves”, each representing a concentrated phase of methodological innovation and mechanistic understanding.^13^

This wave-based framework can be analogously applied to the development of alternative reactor technologies,^2,3,14^ including mechanochemistry,^11,15–22^ where challenges unique to the S–M reaction — such as substrate reactivity,^19,23–26^ catalyst stability,^24–34^ and solvent effects^23,35,36^ — have similarly shaped the pathway of progress. Early mechanochemical studies established proof-of-concept, with palladium-catalyzed S–M couplings conducted under ball-milling conditions. Work from Leadbeater, and subsequently from other groups, including Ondruschka, Su, Ito, and Borchadt extended the substrate scope to include aryl bromides and iodides, and in select cases, aryl chlorides — albeit typically limited to electron-deficient systems or requiring ligand or additive intervention (Scheme 1A).^20,37–42^

**Scheme 1:**
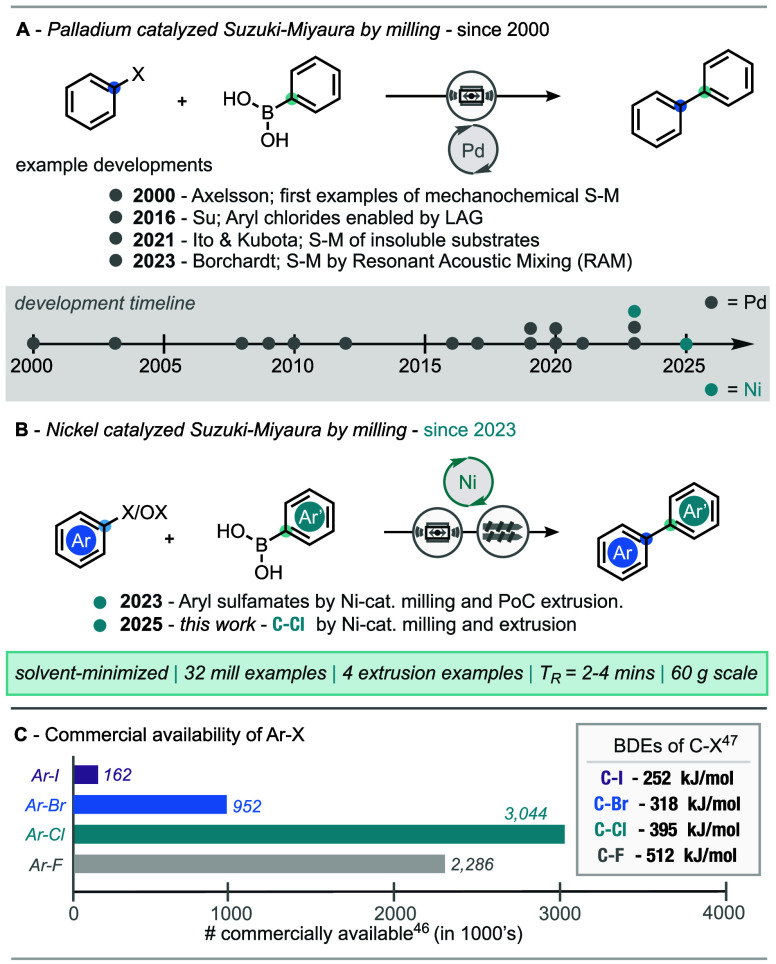
(A) Palladium Catalyzed Suzuki–Miyaura Cross Coupling by Ball-Milling. (B) Nickel-Catalyzed Suzuki–Miyaura Cross-Coupling by Ball-Milling. (C) Commercial Availability^38^ and Bond-Dissociation Energies (BDEs) for Carbon–Halogen Bonds^39^

In 2023, following prior work on cross-electrophile coupling,^43–45^ we reported a nickel-catalyzed mechanochemical coupling of aryl sulfamates, wherein controlled heating during milling enabled access to more challenging electrophiles under reduced-solvent conditions (Scheme 1B).^11^ We reasoned that the ability to thermally treat our mechanochemical processes may permit the ability to activate C–Cl bonds and engage them in S–M coupling mediated by nickel catalysis. Aryl chlorides are inexpensive, and readily available compared to their brominated and iodinated counterparts (Scheme 1C).^46–48^ They are commonly found in commodity chemical feedstocks and industrial intermediates, making them highly desirable for large-scale applications. Additionally, their greater oxidative and hydrolytic stability offers practical advantages in storage and handling, especially under moisture- or air-sensitive conditions.^46^ The higher bond dissociation energy of the C–Cl bond (395 kJ/mol) and its poor leaving group ability pose significant barriers to efficient oxidative addition, particularly in nickel- or palladium-catalyzed cross-coupling. Overcoming this limitation often requires electron-rich and sterically demanding ligands or high reaction temperatures, which can compromise functional group tolerance and sustainability. Herein, we report the first nickel-catalyzed Suzuki–Miyaura coupling of aryl chlorides under heated mechanochemical conditions. The developed protocol has been translated to twin-screw extrusion, furnishing a continuous and scalable route to biaryl products from readily available aryl chloride precursors.

## Results and Discussion

Initial reaction conditions featured 1-chloronaphthalene (0.5 mmol) as the electrophilic coupling partner, 4-fluoro phenylboronic acid as the nucleophilic component, K_3_PO_4_ as base and NiCl_2_(PPh_3_)_2_ as catalyst. Sodium chloride was used as a grinding auxiliary and *n*-hexanol as liquid additive. The reaction was milled for 30 min at 30 Hz, using a stepwise heating profile (moving from room temperature up to 100 °C).^11^ Under these initial conditions the coupled product (**4**) was given in a 62% NMR yield (Table 1, entry 1). Varying the ball mass across a range of 3 to 12 g, demonstrated that the initial 4 g ball was optimum, whereupon increasing or decreasing the ball mass led to a reduced yield and greater recovery of starting material (Table 1, entries 2 and 3 and Supporting Information (SI)). Increasing or decreasing the equivalents of K_3_PO_4_ resulted in a drop in yield, as did adjusting the loading of the grinding auxiliary; NaCl (Table 1, entries 4–10). Using alternative grinding auxiliaries including MgSO_4_, Na_2_SO_4_, silica and Celite all led to a reduced yield. Sodium chloride was selected as the optimal grinding auxiliary, consistent with its established role in enhancing mixing and energy transfer under mechanochemical conditions. While it is possible that halide identity or hygroscopicity may further influence reactivity, we did not explore these variables in detail here.

**Table 1:**
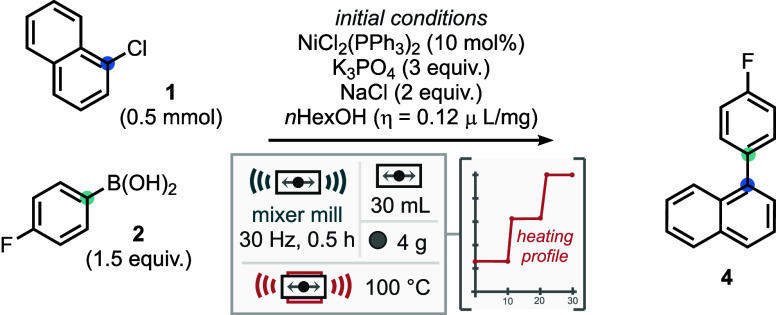

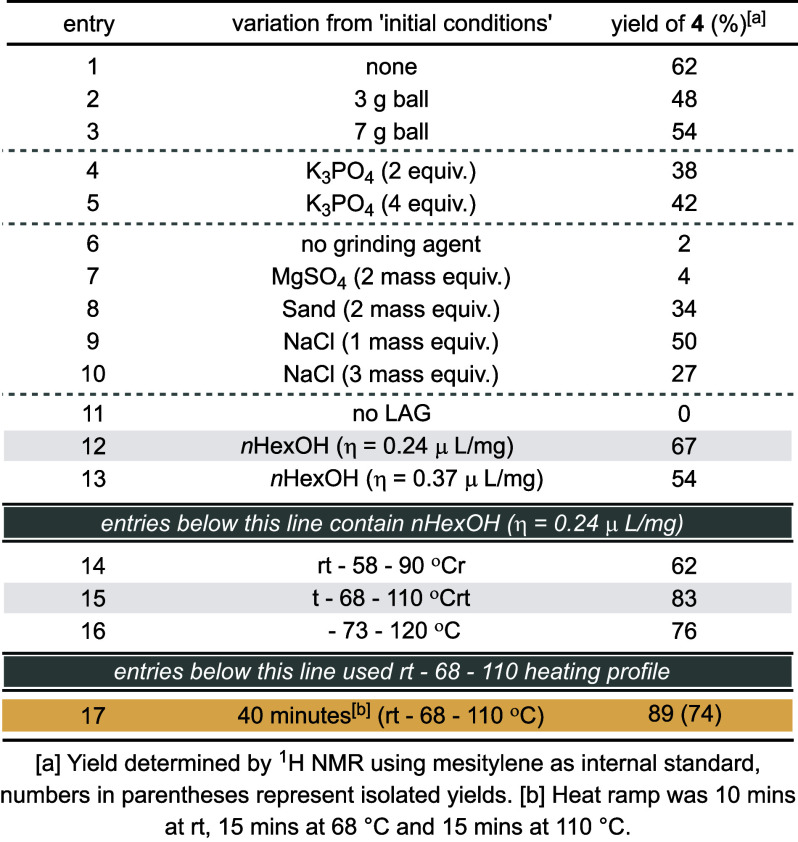
Optimization of the Mechanochemical Nickel-Catalyzed Cross Coupling of Aryl Halides^a^

a Initial conditions; 1-chloro naphthalene (0.5 mmol), 4-fluorophenylboronic acid (1 mmol), dichlorobis­(triphenylphosphine) nickel­(II) (10 mol %), potassium phosphate (1.5 mmol), *n*-hexanol (0.12 μL/mg) and sodium chloride (2 mass equiv).

Moving from assessing solid auxiliaries to the liquid assisted grinding (LAG) agent identified that doubling the *n*-hexanol loading to 0.244 μL mg^–1^ gave a modest improvement in yield from 62 to 67%, further variations in LAG loading led to a negative impact on yield (Table 1, entries 11–13). Examining the reaction temperature and heating profile identified that increasing the maximum temperature to 110 °C (midpoint 68 °C) improved the yield to 83%, further increases to 120 °C (76%) or 130 °C (75%) gave no further improvements in yield and reduced recovery of starting material (see SI). Finally, the reaction time was increased, the initial mixing time at room temperature was kept at 10 min, but the time at 68 and 110 °C was increased to 15 min each, giving a total reaction time of 0.5 h; this improved the yield to 89% with a 74% isolated yield and represents the optimal conditions for this process.

### Substrate Scope

With optimal conditions in hand for the model substrate pairing, the scope of the reaction was explored. A range of 1 and 2-halo-napthalenes (-iodo, -bromo and -chloro) were found to be competent substrates with the 2-iodo, 2-chloro and 1-bromo naphthalenes achieving over 80% isolated yields (Scheme 2A). Examining a range of electronically diverse aryl chlorides found that the methodology performed well for electron poor aryl chlorides **12–16**. *Ortho*-substituted aryl chlorides performed poorly with both electron donating and electron withdrawing substituents (**8, 11, 14** and **19**). Pleasingly hetero aromatic 2-chloro pyridine **18** performed very well under the optimized conditions giving the coupled product in 88% yield.

**Scheme 2:**
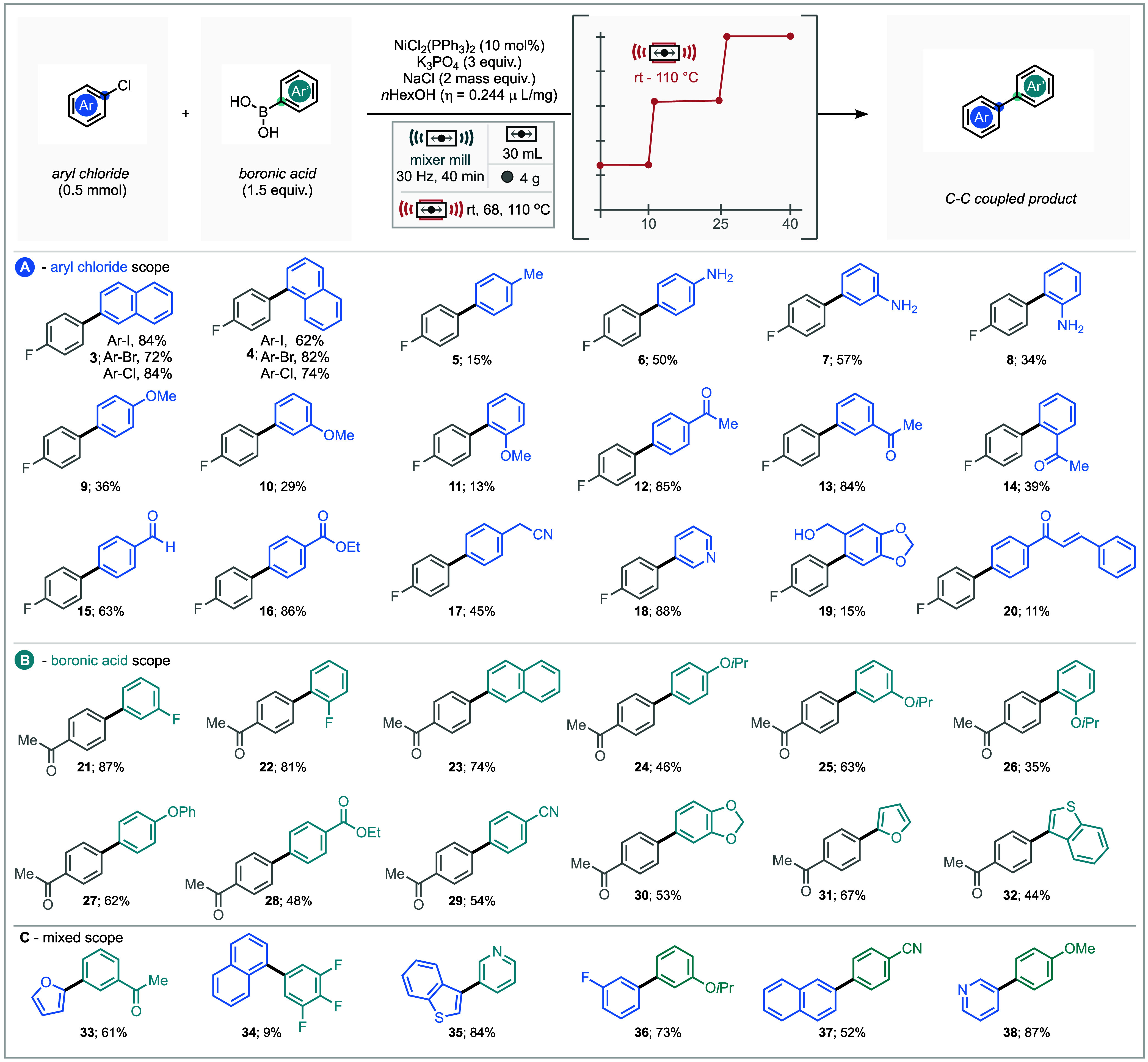
Reaction Scope Using Optimized Conditions: (A) Aryl Chlorides Coupled with 4-Fluorophenyl Boronic Acid. (B) Boronic Acids Coupled with 4-Chloro Acetophenone. (C) Mixed Pairings of Aryl Chlorides and Boronic Acid Substrates^a^

a Optimized reaction conditions: aryl chloride (0.5 mmol), boronic acid (1 mmol), dichlorobis­(triphenylphosphine)­nickel­(II) (10 mol %), potassium phosphate (1.5 mmol), *n*-hexanol (0.24 μL/mg) and sodium chloride (2 mass equiv), 10 min at room temperature, 15 min at 68 °C and 15 min at 110 °C.

The substrate scope with respect to the boronic acid component was assessed using 4-chloroacetophenone as the model electrophile (Scheme 2B). *Ortho-* and *meta-* structural isomers of the model boronic acid gave good yields of the cross-coupled product in 81 and 87% respectively. A series of electron rich (*iso*propoxy and phenoxy, **24**–**27**) boronic acids successfully underwent nickel-catalyzed cross-coupling, as did electron poor ethyl ester and cyano substrates (**28** and **29** respectively). Heteroaromatic boronic acids such as 2-furanylboronic acid and 3-benzothiophene boronic acid performed well, giving coupled products **31** (67%) and **32** (44%); whereas 3-pyridylboronic acid was unsuccessful by these reaction conditions. Both 4-nitrophenylboronic acid and 4-(methylsulfonyl)­phenylboronic acid were also unsuccessful by this methodology.

Pairing 2-furanylboronic acid with 3-chloroacetophenone gave coupled product **33** in a 61% yield, while the reaction between 1-chloro naphthalene and (3,4,5-trifluorophenyl)­boronic acid gave the cross-coupled product (**34**) in a disappointing yield of 9%. By contrast, certain substrates proved unreactive under the optimized conditions, including 3-pyridylboronic acid, 4-nitrophenylboronic acid, and 4-(methylsulfonyl)­phenylboronic acid (see SI, Figure S1 for further examples).

### Extrusion

Aiming to demonstrate the scalability of this methodology our attention was turned to translating the reaction for twin-screw extrusion. Extrusion has become a key technology in answering the need for scaling mechanochemical reactions for applications beyond academia. Extrusion presents a continuous flow approach to mechanochemistry, minimizing the risks posed by of batch scale-up using large mills in the absence of bulk reaction solvent to act as a heat sink.^10^ Over the past decade several groups have provided insights into the scale up of mechanochemical reactions via twin-screw extrusion.^10,11,49,50^ Switching to extrusion changes the primary mechanical force from oblique collisions of the milling ball with the reaction vessel to shearing forces exerted by the kneading sections of the screw. This gives us several new parameters to consider, including screw configuration, screw speed and feed rate.

For these reactions, two kneading sections comprised of forward 60° and alternator 90° kneading elements were chosen for the screw configuration and the screw speed was set to 75 rpm (Scheme 3).

**Scheme 3:**
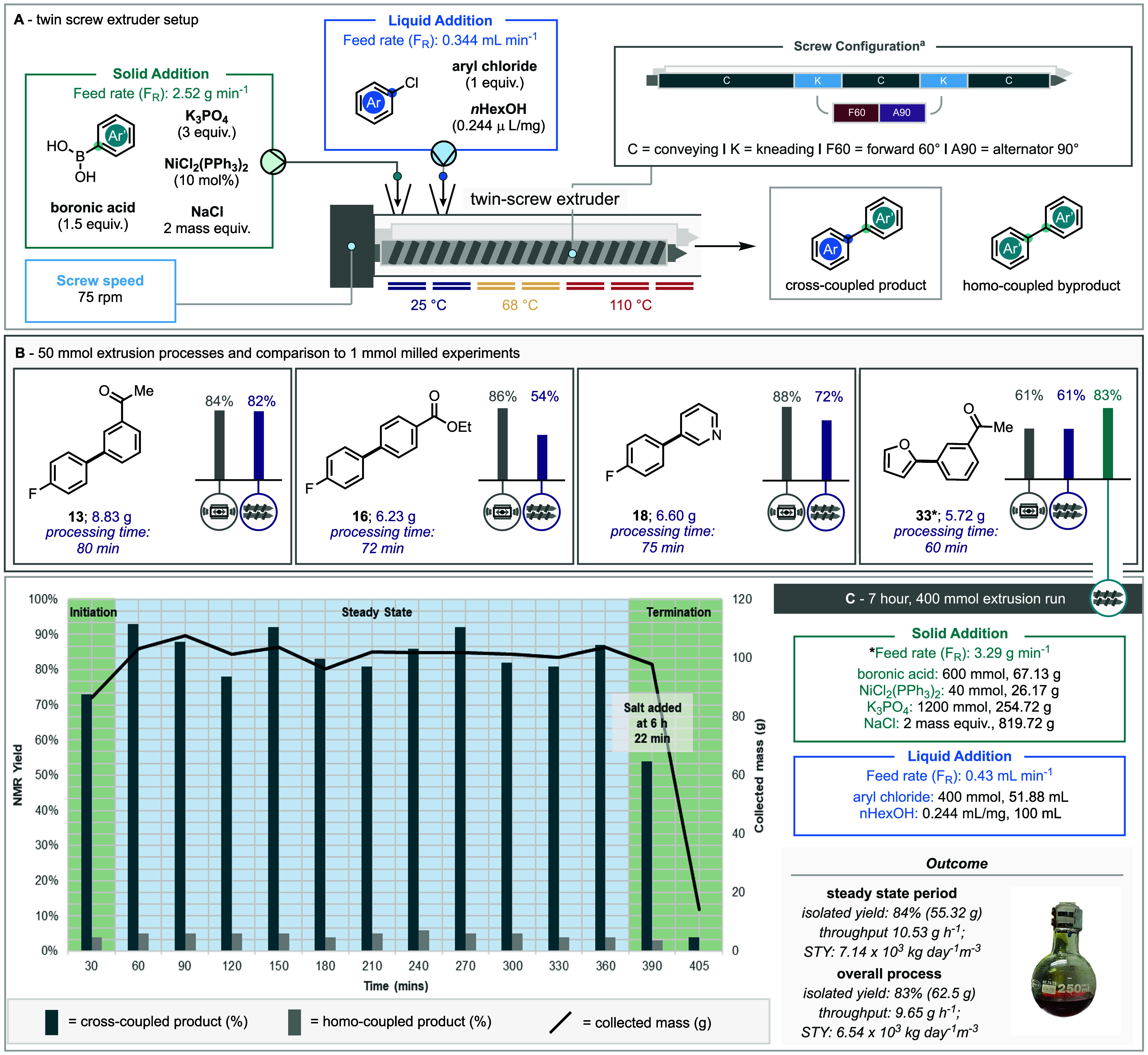
Conversion of Milled Suzuki–Miyaura Process to Extruder (A) General Setup and Operating Parameters of Extrusion Process (B) Comparison of a 1 mmol Ball-Milled Reaction Process to a 50 mmol Extrusion Run (C) 7 h Continual Extrusion Run, Processing 400 mmol of Limiting Aryl Chloride. STY = Space-Time-Yield

The extruder’s seven heating elements were set to mimic the heating profile of the milled reaction, with the first 2 elements set at room temperature, the second two at 68 °C and the final 3 heating elements set to 110 °C (see SI for more details). Three of the best performing aryl chlorides were chosen for this scale-up, 3-chloroacetophenone (**13**), ethyl 4-chloro benzoate (16), and 3-chloropyridine (**18**), coupled with 4-fluorophenylboronic acid (**2**). A fourth extrusion run using 2-furanylboronic acid and 3-chloroacetophenone was also studied. The reactions were scaled up 100-fold, to use 50 mmol of aryl chloride starting material. The solid starting materials – boronic acid (75 mmol), NiCl_2_(PPh_3_)_2_ (5 mmol), K_3_PO_4_ (150 mmol) and NaCl (2 mass equiv) – were premixed by hand in a beaker before loading into the gravimetric hopper over the solid feed port. The liquid aryl chloride was mixed with the *n*-hexanol (0.244 μL/mg) and loaded into a syringe which was connected to the liquid inlet positioned at the second port of the extruder. To achieve a feed rate of 0.811 mmol min^–1^ the gravimetric hopper was set to 2.52 g min^–1^ and the liquid feeder was set to match the stoichiometry of the hopper (see SI). Each extrusion run was carried out until the solid feeder was empty (∼1 h), at which point 25 g of NaCl was added to flush the remaining reaction mixture from extruder.

The residence times ranged from 2 min 3 s to 3 min 13 s, likely due to variations in the rheology of the reagents. The overall run times were similarly varied with the shortest, only 1 h (**33**) and the longest 1 h and 20 min (***13***). These differences in run time are not correlated with the mass of material used in each reaction. It seems likely that these differences are instead related to the rheology of the material affecting the speed of progress through the reactor. All four runs produced over 5 g of product in under 90 min. Both ***13*** and **33** translated to the extruder with no loss in yield compared to the milled reactions, while **18** had a moderate drop in yield from 88 to 72%. The yield of **16** decreased significantly between the mill and extruder from 86% down to 54%.

A larger extrusion run was carried out to gain a clearer view of changes in reactivity as the process reaches steady state. The reaction between 2-furanylboronic acid and 3-chloroacetophenone was chosen as it translated well from the mill and was easiest to purify on a large scale. Aiming to increase the running time from 44 min to 6 h, the reaction was scaled from 50 to 400 mmol with regards to the 3-chloroacetophenone. The addition of reagents was split into 4 equal portions every 1 h and 30 min, with 307.39 g of solid added to the gravimetric hopper and 38 mL added to the syringe pump. To minimize water absorption by the K_3_PO_4_, the solid reagents were weighed out and mixed immediately before each addition. The screw configuration and screw speed were unchanged from the initial extrusion run, while feed rate was fine-tuned from the first run. The solid addition was much faster than expected, calculated to be 3.29 g min^–1^ rather than 2.52 g.min^–1^ as calibrated *ex-situ* with NaCl. For this larger run the solid addition was kept at 3.29 g min^–1^ and the liquid addition increased to 0.43 mL min^–1^ to match the stoichiometry.

The process was run for 6 h and 45 min with a residence time of 2 min 46 s, the extruded material was collected every 30 min. NMR analysis of each section using mesitylene as an internal standard was used to determine the yield of coupled product **33** and the bifuran byproduct **29**. No starting material was observed in any of the collected samples. We first observed an initiation period as the reactor filled with crude reaction material, during the first 30 min the collected mass was lower (86.66 g) than the average at steady state (102.07 g). The corresponding NMR yield of this 30 min sample (73%) was also slightly lower than that of the initial 50 mmol 1 h extrusion run (75% NMR yield), suggesting that much of the 50 mmol process was still in the initiation period.

For the next 5.5 h of processing, *i.e*. from 30 to 360 min, the process moved into a “steady state” period characterized by a consistent collected mass of between 107.51 and 96.41 g (mean 102.07g), and a high mean NMR yield of 91%. Within this period of high productivity there were noticeable fluctuations in both yield and collected mass. We attribute this to the ebb and flow nature of the material progressing through the extruder where material accumulates in the kneading sections until enough pressure is created for it to be pushed through.

After the complete addition of all the reagents (6 h 22 min), NaCl (25 g) was added to the gravimetric hopper and passed through the extruder to push out the remaining reaction mixture. The final 45 min of the extrusion run were the final “termination” section. Although the material collected between 360 and 390 min was 97.88 g due to the addition of NaCl, the NMR yield (54%) was much lower than at steady state. The reaction was continued up to 6 h 45 min, only 14 g of material was collected in the last 15 min, with an NMR yield of just 4%.

The overall NMR yield for the process was 91% (based on 400 mmol of 4-chloroacetophenone), after purification by column chromatography, 65.13 g of product was isolated giving a yield of 83% for the whole process. This corresponds to a throughput rate of 9.26 g h^–1^ and a space time yield of 6538.22 kg day^–1^ m^–3^. These metrics include the initiation and termination periods, in theory as this process is extended these would become increasingly insignificant in comparison to an extended steady state. This theory is corroborated by the increase in overall yield for the 6 h 45 min extrusion run (83%) compared to the 1-h extrusion run (61%). Considering only the output during steady state gives an isolated yield of 57.95 g (88%), which translates to a throughput rate of 10.54 g h^–1^ and a space time yield of 7139.56 kg days^–1^ m^–3^. The calculated PMI, E-factor, and space–time yield for both milling and extrusion are provided in the SI (Section 9), and these compare favorably with representative solution-phase Suzuki–Miyaura protocols.

## Conclusion

This work demonstrates the use of heated mechanochemistry for nickel-catalyzed Suzuki–Miyaura cross-coupling using aryl halides as electrophiles. The reaction was optimized for the use of aryl chlorides as the most atom economic electrophile for this reaction. Increasing the reaction time, temperature and LAG allowed for the coupling of a broad range of electron-rich and electron-poor boronic acids with various aryl chlorides, including heterocycles. Four example reactions were translated from the mixer mill to the twin-screw extruder. With comparable yields, this demonstrates the robustness of this methodology and highlights the potential for continuous production. A longer extrusion run demonstrated the ability to generate substantial quantities (65.13 g) of product within a reasonable time frame (under 7 h). We note that mixture rheology can evolve dynamically during both milling and extrusion, and such effects are likely to play an important role in conversion, selectivity, and scale-up. A systematic exploration of these factors will be an important focus for future studies. Overall, this work paves the way for a more sustainable and scalable Suzuki–Miyaura coupling strategy utilizing readily available aryl chlorides and solvent-minimized mechanochemical activation.

## Supplementary Material



## Notes

Unreactive substrates; screw configuration utilised in this process; sustainability metrics of milled reactions; ^1^H NMR spectrum; ^13^C NMR spectrum; ^19^F NMR spectrum (PDF)

## Notes

This work is dedicated to Professor Steven V. Ley in celebration of his 80th birthday, with deep appreciation for his visionary leadership in organic synthesis and the inspiration and mentorship he has generously shared with his former coworkers and the wider community.
