# Supplementary material for: Nickel-Catalyzed Cross-Coupling of Aryl Chlorides by Heated Mechanochemistry: Scalable Suzuki–Miyaura Reactions via Twin-Screw Extrusion
Source: JACS Au. 2025 Dec 1;5(12):6052–9. doi: 10.1021/jacsau.5c00934 (PMC12728636; doi:10.1021/jacsau.5c00934)
Supplement: Supplementary file 1 [file au5c00934_si_001.pdf]

Supporting Information for

Nickel-Catalyzed Cross-Coupling of Aryl Chlorides by Heated Mechanochemistry: Scalable Suzuki–Miyaura Reactions via Twin-Screw Extrusion

Sarah E. Raby-Buck[a], Renan R. Mattioli[a,b], Robert R. A Bolt[a], Katharine Ingram[c], Julio C. Pastre*[b] and Duncan L. Browne*[a]

[a] Sarah E. Raby-Buck, Dr. Renan R. Mattioli, Dr. Robert R. A. Bolt, Prof. Dr. Duncan L. Browne
Department of Pharmaceutical and Biological Chemistry, University College London (UCL), School of Pharmacy, 29-39 Brunswick Square, Bloomsbury, London, WC1N 1AX, United Kingdom
E-mail: duncan.browne@ucl.ac.uk

[b] Dr. Renan R. Mattioli, Prof. Dr. Julio C. Pastre
Institute of Chemistry, State University of Campinas (UNICAMP), 13083-970, Campinas, SP, Brazil
E-mail: jpastre@unicamp.br

[c] Dr. Katharine Ingram
Syngenta, Jealott's Hill International Research Centre, Bracknell, Berkshire, RG42 6EY, United Kingdom

Table of Contents

General Information....S2
Equipment used....S3
Optimisation....S5
Unreactive substrates....S9
Extrusion Protocols....S10
General Extrusion Procedure....S10
Extrusion runs at 50 mmol....S11
Extrusion run at 400 mmol....S13
Sustainability metrics....S14
Process mass intensity (PMI)....S14
9.2: E-factor....S14
9.3: Space time yield (STY)....S14
Experimental Procedures....S18
Procedure A: Initial investigations into Suzuki–Miyaura coupling of aryl halides S18
Procedure B: Synthesis of biaryls by Suzuki–Miyaura coupling (3–38)....S18
Structural Characterisation....S20
NMR Spectra....S39
References....S99

General Information

All commercial chemicals and solvents were purchased and used as received without further purification unless otherwise stated.

Ball milling was carried out on Retsch MM 400 mixer mill equipped with a homemade heating band system.[1] Stainless steel milling jars and stainless steel grinding balls were used unless stated otherwise.

All spectral data obtained for known compounds were in agreement with the ones reported in the literature.

Thin layer chromatography (TLC) was carried out in aluminium sheets with silica gel 60 matrix and fluorescent indicator at 254 nm. Visualisation of spots were performed with UV irradiation at 254 nm and staining with potassium permanganate basic aqueous solution.

Column chromatography was performed with silica gel at 60 Å pore size and 40−63 µm particle size in Biotage Selekt flash chromatography system.

Melting points (mp) were determined on OptiMelt MPA100 automated melting point system.

Infrared (FTIR) was recorded on Agilent Cary 630 spectrometer using a diamond ATR sampling accessory. Wavenumbers of infrared bands are reported as $\tilde{\nu}$ values (cm^{-1}).

^{1}H NMR, ^{19}F NMR and ^{13}C NMR spectra were recorded on Bruker Avance 400 MHz spectrometer operating at 400 MHz (^{1}H nuclei), 376 MHz (^{19}F nuclei) and 100 MHz (^{13}C nuclei) frequencies at 293 K. Chemical shifts are reported as δ values (ppm) referenced to the signal of trace $CHCl_3$ in $CDCl_3$ chloroform (^{1}H: 7.26 ppm, ^{13}C: 77.2 ppm). Multiplicities are given as "s" (singlet), "br s" (broad singlet), "d" (doublet), "dd" (doublet of doublets), "ddd" (doublet of doublets of doublets), "t" (triplet), "dt" (doublet of triplets), "td" (triplet of doublets), "q" (quartet), "hept" (heptet) and "m" (multiplet). Coupling constants (*J*) are reported in Hertz (Hz). Samples were prepared in deuterated solvent immediately before spectral acquisition. For previously reported compounds, NMR data was compared with the literature results, and the reference is cited.

High-resolution mass spectrometry (HRMS) was carried out on Agilent Q-TOF 6545 mass spectrometer equipped with an electrospray source at 298 K. Molecular ions are reported as *m*/*z* values. Average mass errors are reported as $|\Delta m/z|$ values (ppm) related to the mass difference between calculated and experimental protonated molecular ion $[M+H]^+$ masses. Samples were prepared in HPLC gradient grade methanol immediately before experimental acquisition.

Equipment used

Mixer Mills: Mechanochemical reactions were conducted using a Retsch MM400 mixer mill at the frequency denoted where relevant (below see www.retsch.com for more details).

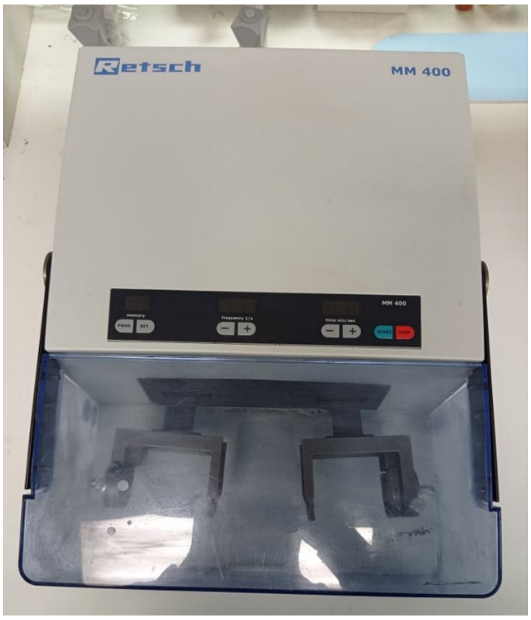

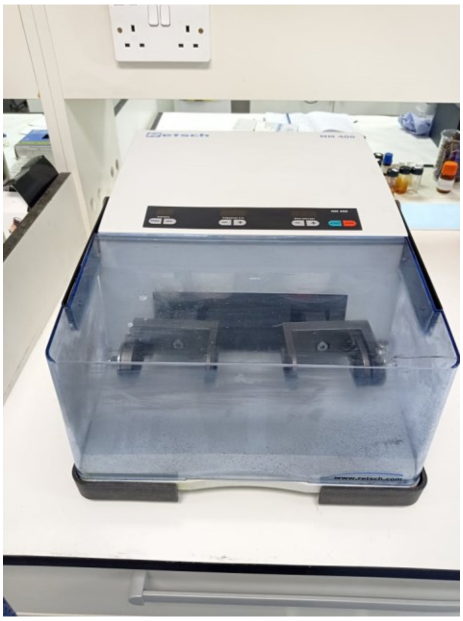

Jars: Mechanochemical reactions were conducted using stainless steel jars (316 SS grade) with Teflon seals which were fabricated in-house (15 mL left, 30 mL right).

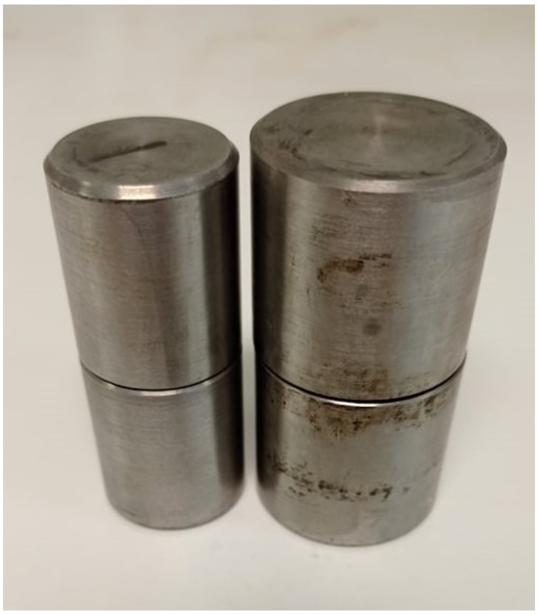

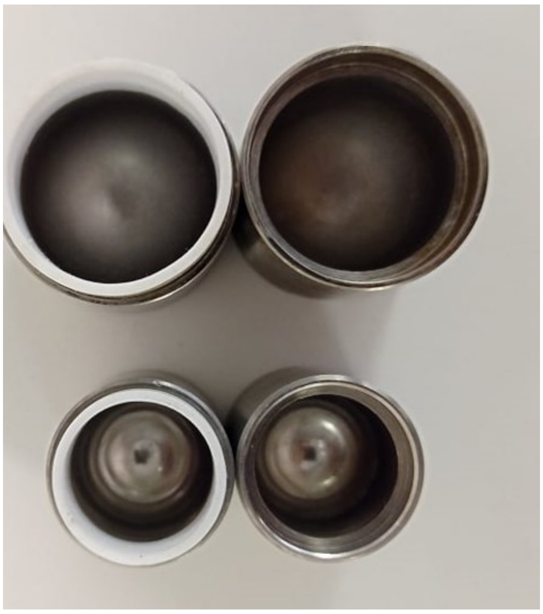

Balls: Stainless steel balls (316 SS grade) were purchased from Bearing Boys limited (www.bearingboys.co.uk) at a variety of sizes (see below, left to right: 2 g, 3 g, 4 g, 7 g, 9 g and 12 g).

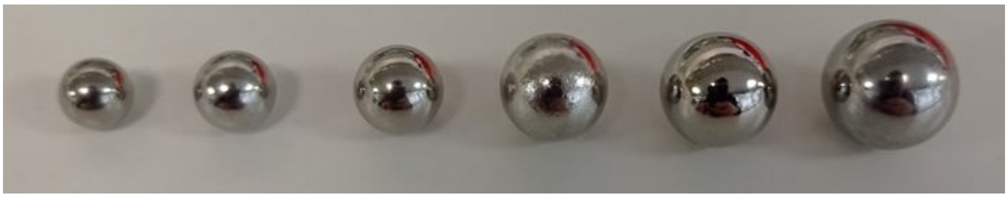

Band Heater: PID controlled heater prototyped and built in house, for further details see previous work by Browne et al 2022.[1]

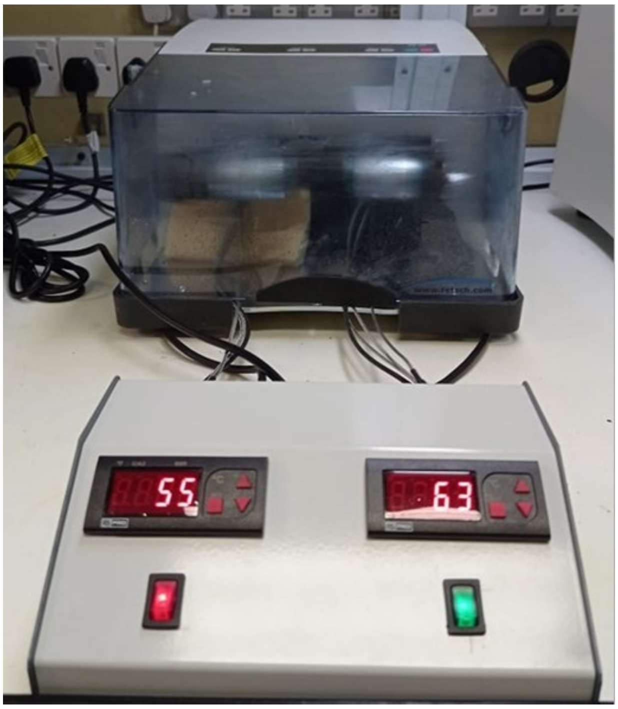

Retsch MM400 milling arm cover and base plate removal: For heat band experiments both the milling arms covers, and the base plate needs to be removed. The plastic cover on the milling arms is removed using a Phillips screwdriver (see below).

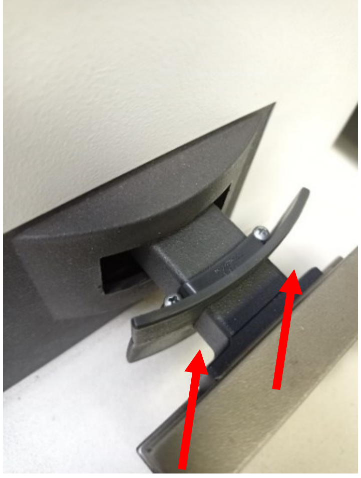

To remove the base plate, place the instrument on its side and remove the three screws with a M4 Allen key (see below).

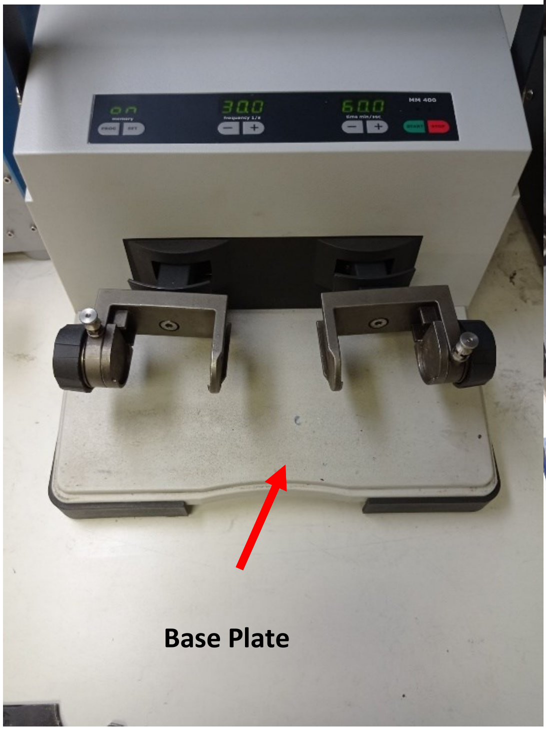

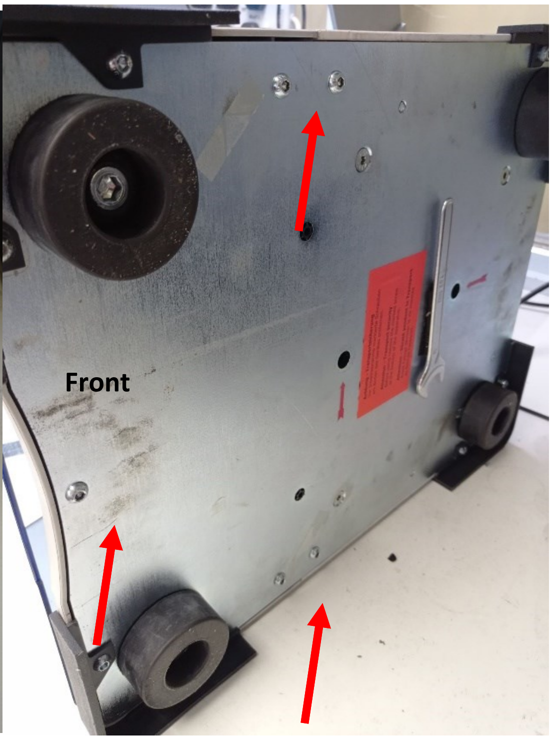

Optimisation

To a 30 mL stainless steel milling jar were added a stainless steel milling ball, 4-fluorophenylboronic acid (**2a**, 104.9 mg, 0.75 mmol), 1-chloronaphthalene (**1cc**, 68.1 µL, 0.50 mmol), catalyst (0.05 mmol, 10 mol%), tripotassium phosphate as base, grinding agent, and *n*-hexanol as liquid-assisted grinding agent. A band heater encased the jar and the jar was milled at 30 Hz. After the reaction time the milling was stopped and the jar was cooled down to room temperature before any further manipulation. The reaction mixture was removed from the jar into a conical flask with ethyl acetate (30 mL) and water (30 mL). The mixture was sonicated for 5 minutes, the organic layer was separated, washed with 1.1 M solution of sodium hydroxide (25 mL), water (50 mL), brine (50 mL), dried over magnesium sulfate (20 g) and evaporated in rotary evaporator under reduced pressure to give the crude residue. To the resulting crude were added $CDCl_3$ (600 µL) and mesitylene (23.2 µL). The yield of the reaction was determined by ^1H NMR analysis.

Table S1. Screening of ball sizes on Suzuki–Miyaura reaction in milling

Entry	Ball size (g)	NaphCl (%)	Yield(%)	Balance (%)[a]
**1**	3	41	**48**	89
**2**	4	29	**62**	91
**3**	7	43	**54**	92
**4**	9	38	**45**	83
**5**	12	47	**45**	92

Conditions: 4-Fluorophenylboronic acid (**2a**, 104.9 mg, 0.75 mmol), 1-chloronaphthalene (**1cc**, 68.1 µL, 0.50 mmol), bis(triphenylphosphine)nickel(**II**) dichloride (32.7 mg, 0.05 mmol), tripotassium phosphate (318.4 mg, 1.5 mmol), sodium chloride (1074.6 mg, 2.0 mass equiv.), *n*-hexanol (65.6 µL, 0.122 µL/mg), at room temperature for 10 minutes, then 63 °C for 10 minutes and then 100 °C for 10 minutes. [a]Sum of individual NMR yields from product **3c** and residual 1-chloronaphthalene (**1cc**).

Table S2. Screening of tripotassium phosphate equivalents on Suzuki–Miyaura reaction in milling

Entry	K_3PO_4 (equiv.)	NaphCl (%)	Yield (%)	Balance (%)[a]
**1**	1.0	63	**29**	92
**2**	2.0	60	**38**	98
**3**	3.0	29	**62**	91
**4**	4.0	42	**42**	84
**5**	5.0	31	**54**	85

Conditions: 4-Fluorophenylboronic acid (**2a**, 104.9 mg, 0.75 mmol), 1-chloronaphthalene (**1cc**, 68.1 µL, 0.50 mmol), bis(triphenylphosphine)nickel(**II**) dichloride (32.7 mg, 0.05 mmol), tripotassium phosphate, sodium chloride (2.0 mass equiv.), *n*-hexanol (0.122 µL/mg), using 4 g diameter milling ball at room temperature for 10 minutes, then 63 °C for 10 minutes and then 100 °C for 10 minutes. [a]Sum of individual NMR yields from product **3c** and residual 1-chloronaphthalene (**1cc**).

Table S3. Screening of grinding agents on Suzuki–Miyaura reaction in milling

Entry	Grinding agent	NaphCl (%)	Yield (%)	Balance (%)[a]
**1**	NaCl	29	**62**	91
**2**	Na_2SO_4	56	**19**	75
**3**	$MgSO_4$	65	**4**	69
**4**	Sand	21	**34**	55
**5**	Celite	78	**4**	82
**6**	-	79	**2**	81

Conditions: 4-Fluorophenylboronic acid (**2a**, 104.9 mg, 0.75 mmol), 1-chloronaphthalene (**1cc**, 68.1 µL, 0.50 mmol), bis(triphenylphosphine)nickel(**II**) dichloride (32.7 mg, 0.05 mmol), tripotassium phosphate (318.4 mg, 1.5 mmol), grinding agent (1074.6 mg, 2.0 mass equiv.), *n*-hexanol (65.6 µL, 0.122 µL/mg), using 4 g diameter milling ball at room temperature for 10 minutes, then 63 °C for 10 minutes and then 100 °C for 10 minutes. [a] Sum of individual NMR yields from product **3c** and residual 1-chloronaphthalene (**1cc**).

Table S4. Screening of sodium chloride mass equivalents on Suzuki–Miyaura reaction in milling

Entry	NaCl (mass equiv.)	NaphCl (%)	Yield (%)	Balance (%)[a]
**1**	0	36	**2**	38
**2**	1.0	42	**50**	92
**3**	1.5	41	**36**	77
**4**	2.0	29	**62**	91
**5**	2.5	50	**27**	77
**6**	3.0	53	**27**	80

Conditions: 4-Fluorophenylboronic acid (**2a**, 104.9 mg, 0.75 mmol), 1-chloronaphthalene (**1cc**, 68.1 µL, 0.50 mmol), bis(triphenylphosphine)nickel(**II**) dichloride (32.7 mg, 0.05 mmol), tripotassium phosphate (318.4 mg, 1.5 mmol), sodium chloride, *n*-hexanol (65.6 µL, 0.122 µL/mg), using 4 g diameter milling ball at room temperature for 10 minutes, then 63 °C for 10 minutes and then 100 °C for 10 minutes. [a] Sum of individual NMR yields from product **3c** and residual 1-chloronaphthalene (**1cc**).

Table S5. Screening of *n*-hexanol equivalents on Suzuki–Miyaura reaction in milling

Entry	*n*-Hexanol (µL/mg)	NaphCl (%)	Yield (%)	Balance (%)[a]
**1**	0	77	**0**	77
**2**	0.061	56	**23**	79
**3**	0.122	29	**62**	91
**4**	0.183	24	**64**	88
**5**	0.244	19	**67**	86
**6**	0.305	14	**67**	81
**7**	0.366	26	**54**	80

Conditions: 4-Fluorophenylboronic acid (**2a**, 104.9 mg, 0.75 mmol), 1-chloronaphthalene (**1cc**, 68.1 µL, 0.50 mmol), bis(triphenylphosphine)nickel(**II**) dichloride (32.7 mg, 0.05 mmol), tripotassium phosphate (318.4 mg, 1.5 mmol), sodium chloride (1074.6 mg, 2.0 mass equiv.), *n*-hexanol, using 4 g diameter milling ball at room temperature for 10 minutes, then 63 °C for 10 minutes and then 100 °C for 10 minutes. [a] Sum of individual NMR yields from product **3c** and residual 1-chloronaphthalene (**1cc**).

Table S6. Screening of temperature on Suzuki–Miyaura reaction in milling

Entry	Temperatures (T_1, T_2)	NaphCl (%)	Yield (%)	Balance (%)[a]
**1**	58, 90	21	**62**	83
**2**	63, 100	19	**67**	86
**3**	68, 110	5	**83**	88
**4**	73, 120	0	**76**	76
**5**	78, 130	2	**75**	77

Conditions: 4-Fluorophenylboronic acid (**2a**, 104.9 mg, 0.75 mmol), 1-chloronaphthalene (**1cc**, 68.1 µL, 0.50 mmol), bis(triphenylphosphine)nickel(**II**) dichloride (32.7 mg, 0.05 mmol), tripotassium phosphate (318.4 mg, 1.5 mmol), sodium chloride (1074.6 mg, 2.0 mass equiv.), *n*-hexanol (131.1 µL, 0.244 µL/mg), using 4 g diameter milling ball at room temperature for 10 minutes, then indicated T_1 temperature for 10 minutes and then indicated T_2 temperature for 10 minutes. [a] Sum of individual NMR yields from product **3c** and residual 1-chloronaphthalene (**1cc**).

Table S7. Screening of catalysts on Suzuki–Miyaura reaction in milling

Entry	Catalyst (10 mol%)	NaphCl (%)	Yield (%)	Balance (%)[a]
**1**	$Ni(PPh_3)_2Cl_2$	5	**83**	88
**2**	$Ni(PPh_3)_2Cl_2$ + 5 mol% PPh_3	0	**79**	79
**3**	$Ni(PPh_3)_2Cl_2$ + 20 mol% PPh_3	5	**78**	83
**4**	$Ni(PCy)_2Cl_2$	15	**47**	62
**5[b]**	$Ni(PPh_3)_2Cl_2$	0	**89**	89

Conditions: 4-Fluorophenylboronic acid (**2a**, 104.9 mg, 0.75 mmol), 1-chloronaphthalene (**1cc**, 68.1 μL, 0.50 mmol), catalyst, tripotassium phosphate (318.4 mg, 1.5 mmol), sodium chloride (2.0 mass equiv.), *n*-hexanol (0.244 μL/mg), using 4 g diameter milling ball at room temperature for 10 minutes, then 68 °C for 10 minutes and then 110 °C for 10 minutes. [a] Sum of individual NMR yields from product **3c** and residual 1-chloronaphthalene (**1cc**). [b]The heating ramp profile was room temperature for 10 minutes, then 68 °C for 15 minutes and then 110 °C for 15 minutes.

Table S8. Screening of 1-naphthyl halides on Suzuki–Miyaura reaction in milling

Entry	Halide	NaphX[a]	Yield (%)	Balance (%)[a]
**1**	Chloride	0	**89 (74[b])**	89
**2**	Bromide	0	**86 (82[b])**	86
**3**	Iodide	0	**83 (62[b])**	83

Conditions: 4-Fluorophenylboronic acid (**2a**, 104.9 mg, 0.75 mmol), 1-naphthyl halide (0.50 mmol), bis(triphenylphosphine)nickel(**II**) dichloride (32.7 mg, 0.05 mmol), tripotassium phosphate (318.4 mg, 1.5 mmol), sodium chloride (1074.6 mg, 2.0 mass equiv.), *n*-hexanol (131.1 μL, 0.244 μL/mg), using 4 g diameter milling ball at room temperature for 10 minutes, then 68 °C for 15 minutes and then 110 °C for 15 minutes. [a] Sum of individual NMR yields from product and residual 1-naphthyl halide.[b]Isolated yield (see *Procedure B* section for more purification details and scope).

Unreactive substrates

Figure S1. Unreactive substrates.

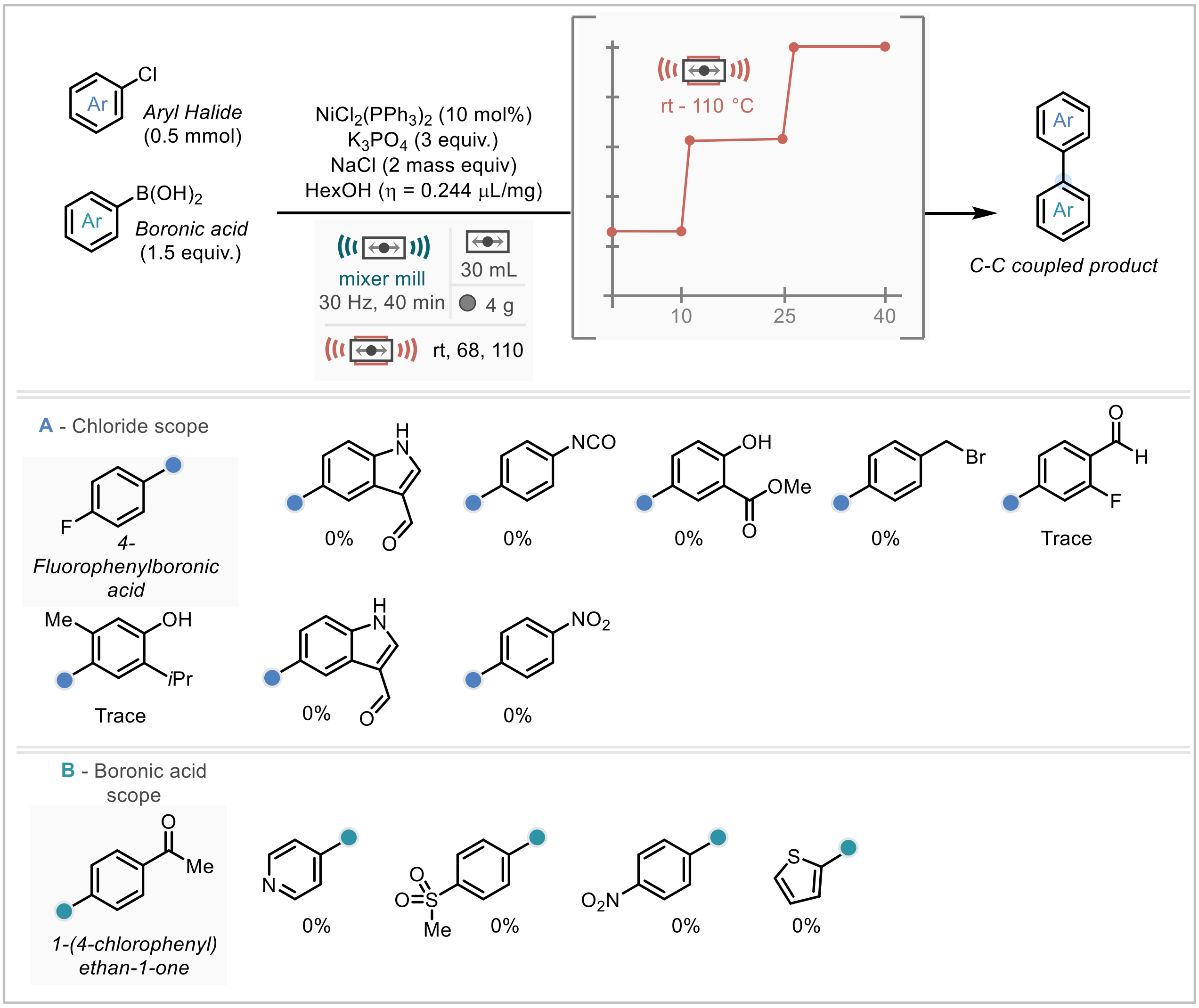

Extrusion Protocols

The large-scale extrusion protocols were carried out using a Thermo Scientific™ Process 11 Twin-screw Extruder (TSE) with 7 controllable heating sections.

General Extrusion Procedure

To a large beaker was added aryl chloride (1 equiv.), boronic acid (1.5 equiv.), bis(triphenylphosphine)nickel(II) dichloride (10 mol%), sodium chloride (2.0 mass equiv.) and tripotassium phosphate (3.0 equiv.). The mixture was mixed by hand using a spatula. The pre-mixed mixture was added to the gravimetric hopper situated at the first port (Main Feed, Figure S2) with the relevant feed rates indicated below – calibrated ex situ. *n*-Hexanol (0.244 µL/mg) was added via syringe pump through the second port (Liquid feeding) at the rates indicated below.

The TSE was set at 75 rpm and each of the twin screw extruder seven heating zones were heated to the correct temperature (2 x 25 °C, 2 x 68 °C, 3 x 110 °C).

The screw configuration was arranged as shown below in Figure S2.

Figure S2. Screw configuration utilised in this process, provided by Thermo Fisher Scientific.

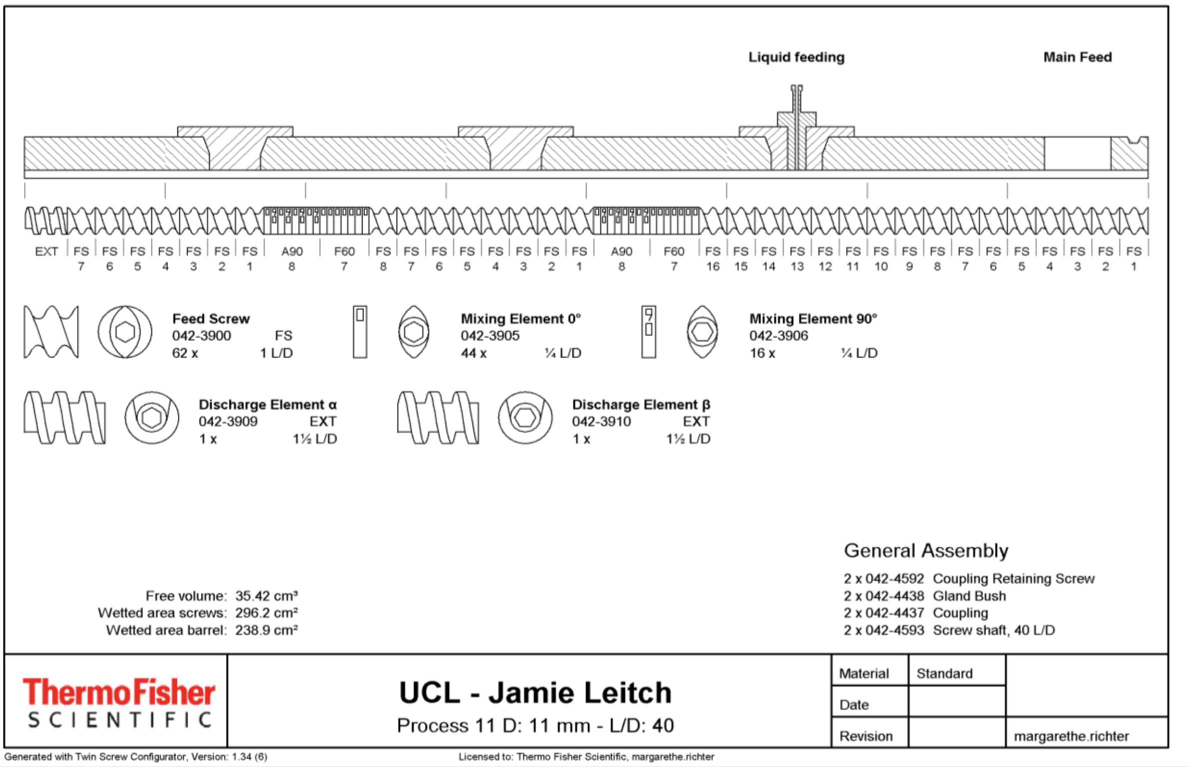

The reaction mixture came out of the twin screw extruder and was collected in a beaker filled with water (~ 100 mL). At the end of the run the mixture was added to a separating funnel and ethyl acetate added (~ 100 mL). The reaction mixture was then washed with 1.1 M sodium hydroxide solution, water and brine, dried over magnesium sulfate and concentrated in vacuo to give the crude reaction mixture.

Extrusion runs at 50 mmol

3-(4-Fluorophenyl)acetophenone (13):

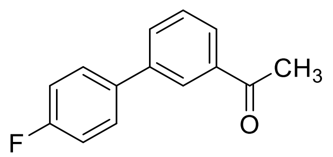

To a large beaker was added 4-fluorophenylboronic acid (10.49 g, 75 mmol, 1.5 equiv.), bis(triphenylphosphine) nickel(II) dichloride (3.27 g, 10 mol%), sodium chloride (106.38 g, 2 mass equiv.) and tripotassium phosphate (31.84 g, 3.0 equiv.). The mixture was mixed by hand using a spatula. The pre-mixed mixture was added to the gravimetric hopper situated at the first port (Main Feed, Figure S2) with a feed rate of 2.52 $gmin^{-1}$ – calibrated ex situ. 1-(3-Chlorophenyl)ethan-1-one (6.48 mL, 50 mmol, 1.0 equiv.), and *n*-hexanol (13.01 mL, 0.244 μL/mg) were added via syringe pump through the second port (Liquid feeding) at a rate of 0.323 mL min^{-1}.

The TSE was set at 75 rpm and each of the twin screw extruder seven heating zones were heated to the correct temperature (2 x 25 °C, 2 x 68 °C, 3 x 110 °C).

Material started appearing at the end of the extruder after ~ 2 minutes and 7 seconds, and torque values between 1.8 and 3.2 Nm were maintained throughout the process. After 59 minutes and 43 seconds, sodium chloride (25 g) was added to flush out the remaining reaction mixture, 1 hour and 16 minutes.

The reaction mixture was collected as it came out of the twin screw extruder in a beaker filled with water (~ 100 mL). At the end of the run the mixture was added to a separating funnel and ethyl acetate added (~ 100 mL). The reaction mixture was then washed with 1.1 M sodium hydroxide solution, water and brine, dried over magnesium sulfate and concentrated in vacuo to give the crude reaction mixture.

The reaction was purified by column chromatography (hexane: ethyl acetate 9:1) to give 3-(4-fluorophenyl)acetophenone as a yellow oil (8.83 g, 82%).

Ethyl 4-(4-fluorophenyl)benzoate (16):

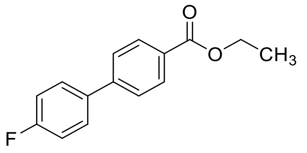

To a large beaker was added 4-fluorophenylboronic acid (10.49 g, 75 mmol, 1.5 equiv.), bis(triphenylphosphine) nickel(II) dichloride (3.27 g, 10 mol%), sodium chloride (109.66 g, 2.0 mass equiv.) and tripotassium phosphate (31.84 g, 3.0 equiv.). The mixture was mixed by hand using a spatula. The pre-mixed mixture was added to the gravimetric hopper situated at the first port (Main Feed, Figure S2) with a feed rate of 2.52 $gmin^{-1}$ – calibrated ex situ. Ethyl 4-chlorobenzoate (7.82 mL, 50 mmol, 1.0 equiv.), and *n*-hexanol (13.37 mL, 0.244 μL/mg) were added via syringe pump through the second port (Liquid feeding) at a rate of 0.344 mL min^{-1}.

The TSE was set at 75 rpm and each of the twin screw extruder seven heating zones were heated to the correct temperature (2 x 25 °C, 2 x 68 °C, 3 x 110 °C).

Material started appearing at the end of the extruder after ~ 3 minutes and 13 seconds, and torque values between 1.8 and 3.2 Nm were maintained throughout the process. After 1 hour and 3 minutes, sodium chloride (25 g) was added to flush out the remaining reaction mixture, the reaction was stopped after 1 hour and 16 minutes.

The reaction mixture was collected as it came out of the twin screw extruder in a beaker filled with water (~ 100 mL). At the end of the run the mixture was added to a separating funnel and ethyl acetate added (~ 100 mL). The reaction mixture was then washed with 1.1 M sodium hydroxide solution, water and brine, dried over magnesium sulfate and concentrated in vacuo to give the crude reaction mixture.

The reaction was purified by flash chromatography (ethyl acetate/hexane 3:7) to give ethyl 4-(4-fluorophenyl)benzoate Off-white solid (6.60 g, 54%).

3-(4-Fluorophenyl)pyridine (18):

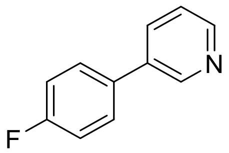

To a large beaker was added 4-fluorophenylboronic acid (10.49 g, 75 mmol, 1.5 equiv.), bis(triphenylphosphine)nickel(II) dichloride (3.27 g, 10 mol%), sodium chloride (91.2 g, 2.0 mass equiv.) and tripotassium phosphate (31.84 g, 3.0 equiv.). The mixture was mixed by hand using a spatula. The pre-mixed mixture was added to the gravimetric hopper situated at the first port (Main Feed, Figure S2) with a feed rate of 2.52 gmin^{-1} – calibrated ex situ. 3-Chloropyridine (4.75 mL, 50 mmol, 1.0 equiv.), and *n*-hexanol (11.12 mL, 0.244 μL/mg) were added via syringe pump through the second port (Liquid feeding) at a rate of 0.294 mL min^{-1}.

The TSE was set at 75 rpm and each of the twin screw extruder seven heating zones were heated to the correct temperature (2 x 25 °C, 2 x 68 °C, 3 x 110 °C). After 57 minutes, sodium chloride (25 g) was added to flush out the remaining reaction mixture.

Material started appearing at the end of the extruder after ~ 2 minutes and 30 seconds, and torque values between 1.8 and 3.2 Nm were maintained throughout the process. After 1 hour, sodium chloride (25 g) was added to flush out the remaining reaction mixture, the reaction was stopped after 1 hour and 18 minutes.

The reaction mixture was collected as it came out of the twin screw extruder in a beaker filled with water (~ 100 mL). At the end of the run the mixture was added to a separating funnel and ethyl acetate added (~ 100 mL). The reaction mixture was then washed with 1.1 M sodium hydroxide solution, water and brine, dried over magnesium sulfate and concentrated in vacuo to give the crude reaction mixture.

The reaction was purified by column chromatography (hexane/ethyl acetate 9:1) to give 3-(4-fluorophenyl)pyridine as a yellow oil (6.23 g, 72%).

3-(2-Furanyl)acetophenone (33):

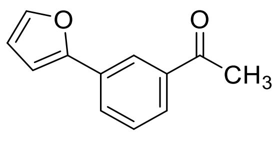

To a large beaker was added 2-furanylboronic acid (8.39 g, 75 mmol, 1.5 equiv.), bis(triphenylphosphine)nickel(II) dichloride (3.27 g, 10 mol%), sodium chloride (102.46 g, 2.0 mass equiv.) and tripotassium phosphate (31.84 g, 3.0 equiv.). The mixture was mixed by hand using a spatula. The pre-mixed mixture was added to the gravimetric hopper situated at the first port (Main Feed, Figure S2) with a feed rate of 3.29 gmin^{-1} – calibrated ex situ. 1-(3-Chlorophenyl)ethan-1-one (6.48 mL, 50 mmol, 1 equiv.), and *n*-hexanol (12.5 mL, 0.244 µL/mg) were added via syringe pump through the second port (Liquid feeding) at a rate of 0.399 mL min^{-1}.

The TSE was set at 75 rpm and each of the twin screw extruder seven heating zones were heated to the correct temperature (2 x 25 °C, 2 x 68 °C, 3 x 110 °C).

Material started appearing at the end of the extruder after ~ 2 minutes and 3 seconds, and torque values between 2 and 3.2 Nm were maintained throughout the process. After 47 minutes, sodium chloride (25 g) was added to flush out the remaining reaction mixture, the reaction was stopped after 60 minutes and 32 seconds.

The reaction mixture was collected as it came out of the twin screw extruder in a beaker filled with water ~ 100 mL). At the end of the run the mixture was added to a separating funnel and ethyl acetate added (~ 100 mL). The reaction mixture was then washed with 1.1 M sodium hydroxide solution, water and brine, dried over magnesium sulfate and concentrated in vacuo to give the crude reaction mixture.

The reaction was purified by column chromatography (hexane/ethyl acetate 9:1) to give 1-(3-(furan-2-yl)phenyl)ethan-1-one as a yellow oil (5.72 g, 61.4%).

Extrusion run at 400 mmol

3-(2-Furanyl)acetophenone (33):

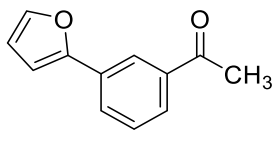

To a large beaker was added 2-furanylboronic acid (67.13 g, 600 mmol, 1.5 equiv.), bis(triphenylphosphine)nickel(II) dichloride (26.17 g, 10 mol%), sodium chloride (819.72 g, 2.0 mass equiv.) and tripotassium phosphate (254.72 g, 3.0 equiv.). The mixture was mixed by hand using a spatula. The pre-mixed mixture was added to the gravimetric hopper situated at the first port (Main Feed, Figure S2) with a feed rate of 3.29 gmin^{-1} – calibrated ex situ. 1-(3-Chlorophenyl)ethan-1-one (51.88 mL, 400 mmol, 1.0 equiv.), and *n*-hexanol (100 mL, 0.244 µL/mg) were added via syringe pump through the second port (Liquid feeding) at a rate of 0.43 mL min^{-1}. The solid addition was split into 4 additions of 307.39 g every 1 hour and 30 minutes, while the liquid addition was split into 4 additions of 37.97 mL every 1 hour and 30 minutes. To minimise water absorption by the tripotassium phosphate – which is slightly hydroscopic – the solid reagents were weighed out just before each addition; 2-furanylboronic acid (16.784 g, 150 mmol), bis(triphenylphosphine)nickel(II) dichloride (6.54 g, 10 mol%), sodium chloride (204.92 g, 2.0 mass equiv.) and tripotassium phosphate (63.68 g, 3.0 equiv.).

The TSE was set at 75 rpm and each of the twin screw extruder seven heating zones were heated to the correct temperature (2 x 25 °C, 2 x 68 °C, 3 x 110 °C).

Material started appearing at the end of the extruder after ~ 2 minutes and 46 seconds, and torque values between 2 and 3.2 Nm were maintained throughout the process. After 6 hours and 22 minutes, sodium chloride (25 g) was added to flush out the remaining reaction mixture, the reaction was stopped after 6 hours and 45 minutes.

The reaction mixture was collected as it came out of the twin screw extruder every 30 minutes in a beaker filled with water (~ 100 mL). At the end of the run the mixture was added to a separating funnel and ethyl acetate added (~ 100 mL). The reaction mixture was then washed with 1.1 M sodium hydroxide solution, water and brine, dried over magnesium sulfate and concentrated in vacuo to give the crude reaction mixture.

The reaction was purified by column chromatography (hexane/ethyl acetate 9:1) to give 1-(3-(furan-2-yl)phenyl)ethan-1-one as a yellow oil (62.5 g, 83%).

Sustainability metrics

Process mass intensity (PMI)

$$PMI_{reaction} = \frac{Total\ mass\ of\ step}{Mass\ of\ product}$$

9.2: E-factor

$$E\ factor = \frac{mass\ of\ input - mass\ of\ product}{mass\ of\ product}$$

9.3: Space time yield (STY)

$$STY\ (kg\ days^{-1} m^{-3}) = \frac{mass\ of\ product\ (kg)}{duration\ of\ reaction\ (days) \times volume\ of\ reactor (m^3)}$$

Milled reactions

Figure S3. Sustainability metrics of milled reactions.

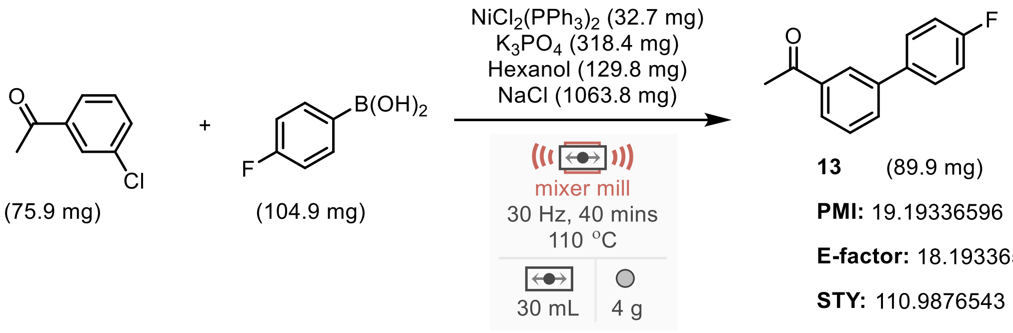

13 (89.9 mg)

PMI: 19.19336596

E-factor: 18.19336596

STY: 110.9876543 kg days^{-1}m^{-3}

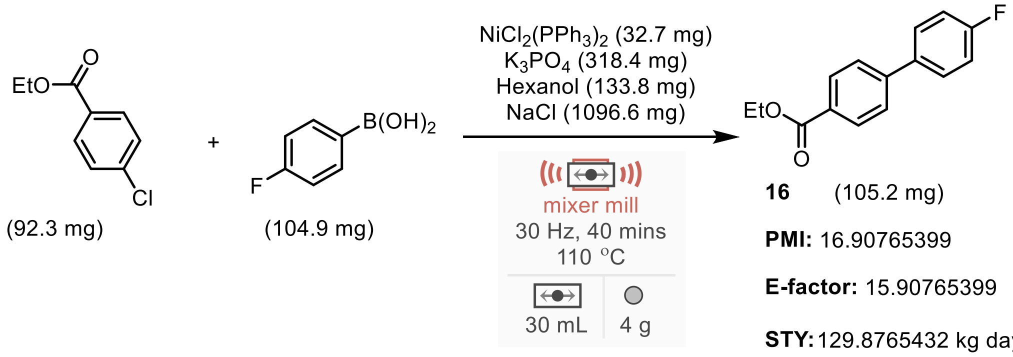

16 (105.2 mg)

PMI: 16.90765399

E-factor: 15.90765399

STY: 129.8765432 kg days^{-1}m^{-3}

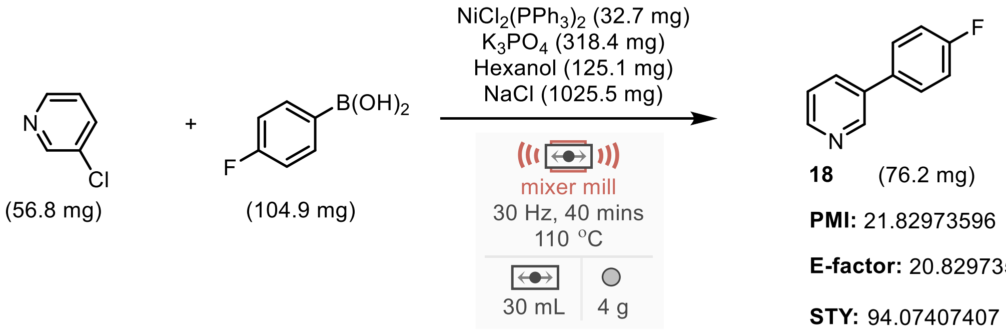

18 (76.2 mg)

PMI: 21.82973596

E-factor: 20.82973596

STY: 94.07407407 kg days^{-1}m^{-3}

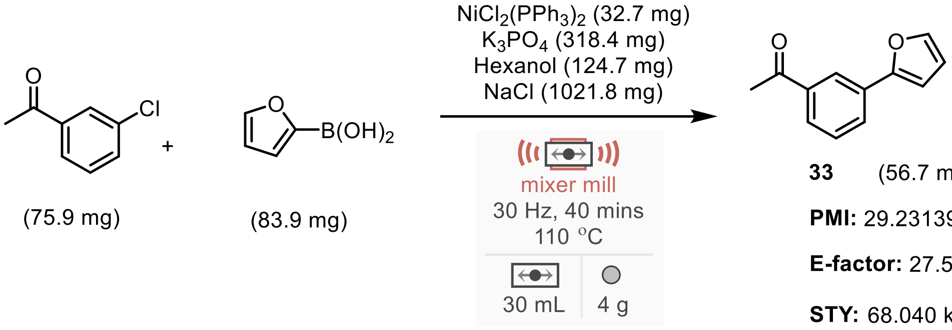

33 (56.7 mg)

PMI: 29.2313933

E-factor: 27.51198942

STY: 68.040 kg days^{-1}m^{-3}

Extruded reactions

Figure S4. Sustainability metrics of milled reactions.

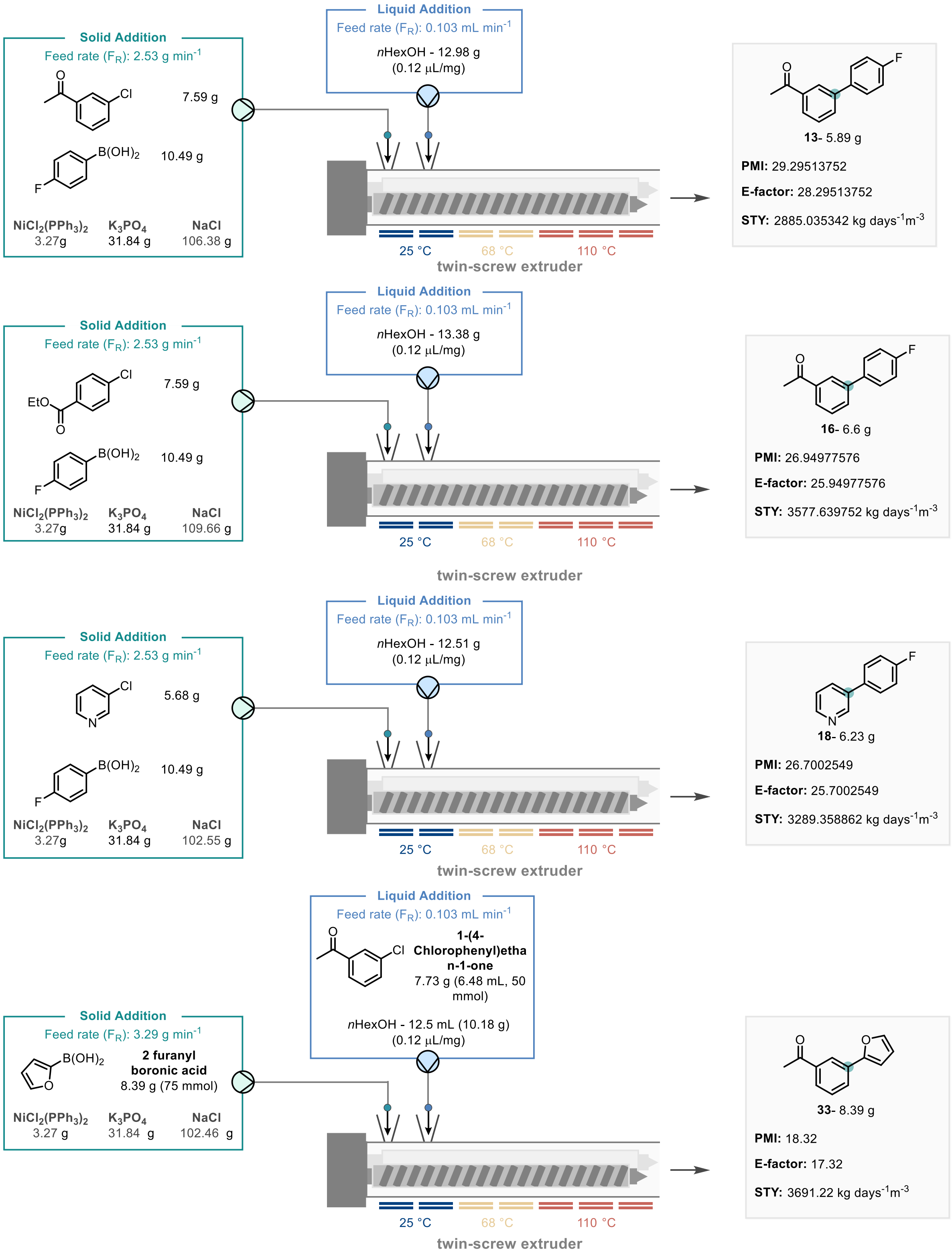

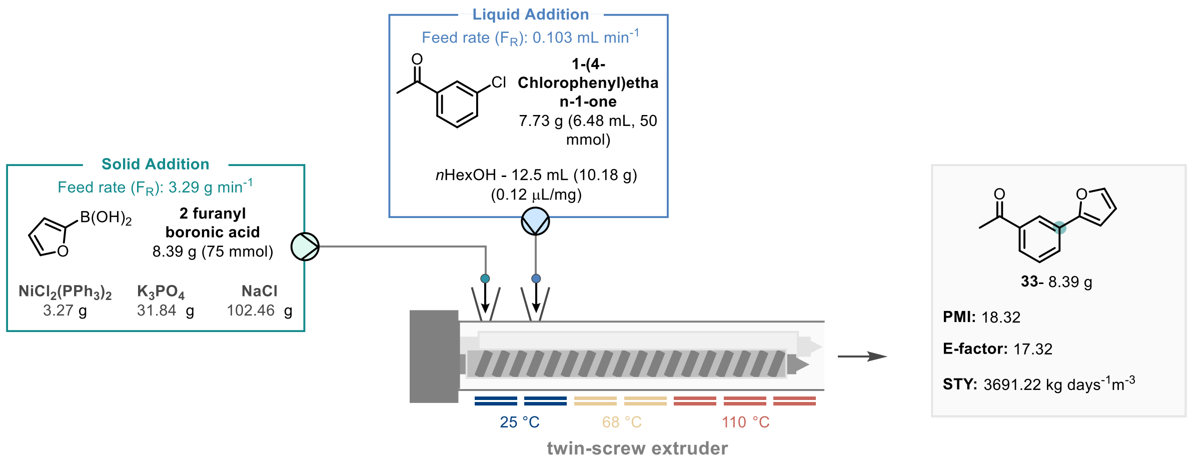
Liquid Addition
Feed rate (F_R): 0.103 mL min-1
1-(4-Chlorophenyl)ethan-1-one
7.73 g (6.48 mL, 50 mmol)
nHexOH - 12.5 mL (10.18 g)
(0.12 µL/mg)
Solid Addition
Feed rate (F_R): 3.29 g min-1
B(OH)2
2 furanyl boronic acid
8.39 g (75 mmol)
NiCl2(PPh3)2
3.27 g
K3PO4
31.84 g
NaCl
102.46 g
25 °C
68 °C
110 °C
twin-screw extruder
33- 8.39 g
PMI: 18.32
E-factor: 17.32
STY: 3691.22 kg days-1m-3

Experimental Procedures

Procedure A: Initial investigations into Suzuki–Miyaura coupling of aryl halides

To a 30 mL stainless steel milling jar was added a 4 g stainless steel ball, aryl halide or aryl sulfamate (0.5 mmol), 4-fluorophenylboronic acid (0.75 mmol), bis(triphenyl phosphine)nickel(II) dichloride (10 mol%), sodium chloride (2 mass equiv.), *n*-hexanol (0.122 µL/mg) and tripotassium phosphate (1.5 mmol). A band heater encased the jar and milled at 30 Hz for 30 minutes. The first 10 minutes were conducted at rt. With the next 10 minutes heated to 63 °C and the remaining 10 minutes to 100 °C. After this time the milling was stopped, and jar allowed to cool to ~ 40 °C before further manipulation. The reaction mixture was removed from the jar into a conical flask with ethyl acetate (~ 30 mL) and water (~ 30 mL).* The organic layer was washed with 1.1 M sodium hydroxide solution (25 mL), water (50 mL), brine (50 mL), dried over magnesium sulfate, and concentrated in vacuo to give the crude reaction mixture.** The resulting crude residue was purified by silica gel flash column chromatography using hexane/ ethyl acetate solvent system to give the pure product unless stated otherwise.***

* A sonicator was used to break up any large pieces.
** A dilute hydrogen peroxide solution (~ 25 mL) was also used for boronic acids containing electron-donating groups to facilitate removal of phosphine ligand, which could co-elute with C–C coupled products.
*** According to literature procedure.[1]

Procedure B: Synthesis of biaryls by Suzuki–Miyaura coupling (**3**–**38**)

To a 30 mL stainless steel milling jar were added a 4 g stainless steel ball, boronic acid (0.75 mmol, 1.5 equiv.), bis(triphenylphosphine)nickel(II) dichloride (0.05 mmol, 10 mol%), sodium chloride (2.0 mass equiv.), aryl chloride (0.50 mmol, 1.0 equiv.), *n*-hexanol (0.244 µL/mg) and tripotassium phosphate (1.5 mmol, 3.0 equiv.). A band heater encased the jar and milled at 30 Hz for 40 minutes. The first 10 minutes were conducted at room temperature. With the next 15 minutes heated to 68 °C and the remaining 15 minutes to 110 °C. After this time the milling was stopped and the jar was cooled down to room temperature before any further manipulation. The reaction mixture was removed from the jar into a conical flask with ethyl acetate (30 mL) and water (30 mL).* The mixture was sonicated for 5 minutes, the organic layer was separated, washed with 1.1 M sodium hydroxide solution (25 mL), water (50 mL), brine (50 mL), dried over magnesium sulfate (20 g) and evaporated in rotary evaporator under reduced pressure to give the crude residue.** Upon completion of the extraction the formation of the product was observed by TLC analysis using hexane or hexane/ethyl acetate mixture as mobile phase. The crude residue was purified by flash chromatography using hexane/ethyl acetate mixture (4.0 L) and silica gel (100 g). The fractions were combined, evaporated in rotary evaporator under reduced pressure, dried in a high vacuum system and stored at room temperature. See *Structural Characterisation* section for more experimental details.

* A sonicator was used to break up any large pieces.
** A dilute hydrogen peroxide solution (~ 25 mL) was also used for boronic acids containing electron-donating groups to facilitate removal of phosphine ligand, which could co-elute with C–C coupled products.

Structural Characterisation

2-(4-Fluorophenyl)naphthalene (**3**)

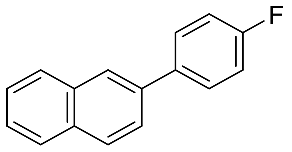

Prepared according to general *Procedure B*, starting with 2-chloronaphthalene (68.1 µL, 0.50 mmol) and 4-fluorophenylboronic acid (104.9 mg, 0.75 mmol). Purified by flash chromatography (4.0 L hexane); Rf = 0.20 (hexane); Yield = 87% (97.0 mg, 0.44 mmol);White solid; mp = 102–105 °C; ^{1}H NMR ($CDCl_3$, 400 MHz) δ 8.00–7.89 (s, 1H), 7.89–7.76 (m, 3H), 7.70–7.53 (m, 3H), 7.53–7.38 (m, 2H), 7.20–7.02 (m, 2 H); ^{13}C NMR ($CDCl_3$, 100 MHz) δ 162.7 (d, J = 246.5 Hz), 137.8, 137.4 (d, J = 2.8 Hz), 133.8, 132.7, 129.2 (d, J = 8.3 Hz), 128.7, 128.3, 127.8, 126.6, 126.2, 125.8, 125.6, 115.9 (d, J = 21.2 Hz) ppm.

This data is consistent with the literature.[2]

1-(4-Fluorophenyl)naphthalene (**4**)

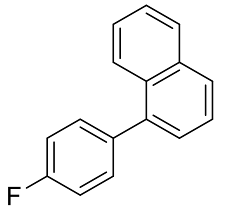

Prepared according to general *Procedure B*, starting with 1-chloronaphthalene (68.1 µL, 0.50 mmol) and 4-fluorophenylboronic acid (104.9 mg, 0.75 mmol). Purified by flash chromatography (4.0 L hexane/ethyl acetate 0–2%); R_f = 0.25 (hexane); Yield = 72% (79.5 mg, 0.36 mmol); White solid; mp = 73–74 °C; FTIR (ATR) $\tilde{\nu}$ 3068, 3042, 2922, 2855, 1603, 1588, 1543, 1502, 1461, 1394, 1334, 1290, 1275, 1237, 1219, 1182, 1155, 1114, 1092, 1055, 1014, 962, 939, 913, 868, 835, 798, 775, 745, 723, 671, 660 cm^{-1}; ^{1}H NMR ($CDCl_3$, 400 MHz) δ 7.87 (d, J = 8.2 Hz, 1H), δ 7.83 (d, J = 8.2 Hz, 2H), δ 7.50–7.38 (m, 5H), δ 7.36 (dd, J = 7.0, 1.0 Hz, 1H), δ 7.17–7.11 (m, 2H); ^{19}F NMR ($CDCl_3$, 376 MHz) δ –115.3 ppm; ^{13}C NMR ($CDCl_3$, 100 MHz) δ 163.6, 161.2, 139.3, 136.8(1), 136.7(8), 133.9, 131.8, 131.7(3), 131.6(5), 128.5, 127.9, 127.1(4), 127.1(3), 126.3, 126.0, 125.9, 125.5, 115.4, 115.2 ppm.

This data is consistent with the literature.[3]

4-(4-Fluorophenyl)toluene (**5**)

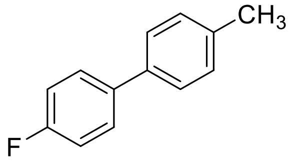

Prepared according to general *Procedure B*, starting with 4-chlorotoluene (59.1 µL, 0.50 mmol) and 4-fluorophenylboronic acid (104.9 mg, 0.75 mmol). Purified by flash chromatography (4.0 L hexane/ethyl acetate 0–2%); R_f = 0.25 (hexane); Yield = 15% (14.4 mg, 0.08 mmol); White solid; mp = 76–77 °C; FTIR (ATR) $\tilde{\nu}$ 3030, 2919, 2859, 2732, 1893, 1655, 1599, 1558, 1528, 1495, 1394, 1379, 1323, 1308, 1234, 1211, 1193, 1159, 1133, 1100, 1040, 1003, 950, 943, 835, 805, 716, 704, 656 cm^{-1}; ^1H NMR ($CDCl_3$, 400 MHz) δ 7.52 (dd, J = 8.5, 5.5 Hz, 2H), δ 7.44 (d, J = 7.9 Hz, 2H), δ 7.24 (d, J = 8.5 Hz, 2H), δ 7.11 (t, J = 8.7 Hz, 2H), δ 2.39 (s, 3H); ^{19}F NMR ($CDCl_3$, 376 MHz) δ –116.3 ppm; ^{13}C NMR ($CDCl_3$, 100 MHz) δ 163.6, 161.2, 137.5, 137.4(1), 137.3(8), 137.2, 129.7, 128.6(4), 128.5(6), 127.0, 115.8, 115.6, 21.2 ppm.

This data is consistent with the literature.[4]

4-(4-Fluorophenyl)aniline (**6**)

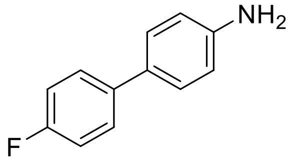

Prepared according to general *Procedure B*, starting with 4-chloroaniline (63.8 mg, 0.50 mmol) and 4-fluorophenylboronic acid (104.9 mg, 0.75 mmol). Purified by flash chromatography (4.0 L hexane/ethyl acetate 0–60%); R_f = 0.20 (hexane/ethyl acetate 30%); Yield = 50% (46.5 mg, 0.25 mmol); Pale yellow solid; mp = 118–120 °C; FTIR (ATR) $\tilde{\nu}$ 3452, 3418, 3295, 3191, 3068, 3034, 3008, 2956, 2922, 2851, 1890, 1700, 1685, 1633, 1600, 1491, 1398, 1286, 1267, 1223, 1159, 1137, 1103, 1003, 947, 842, 813, 723, 701 cm^{-1}; ^1H NMR ($CDCl_3$, 400 MHz) δ 7.51–7.48 (m, 2H), δ 7.38 (d, J = 8.4 Hz, 2H), δ 7.12–7.08 (m, 2H), δ 6.76 (d, J = 8.4 Hz, 2H), δ 3.74 (br s, 2H); ^{19}F NMR ($CDCl_3$, 376 MHz) δ –117.3 ppm; ^{13}C NMR ($CDCl_3$, 100 MHz) δ 163.1, 160.7, 145.9, 137.4(1), 137.3(8), 130.7, 128.0, 127.9, 115.6, 115.5, 115.4 ppm.

This data is consistent with the literature.[5]

3-(4-Fluorophenyl)aniline (**7**)

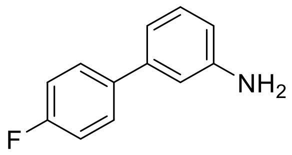

Prepared according to general *Procedure B*, starting with 3-chloroaniline (52.9 µL, 0.50 mmol) and 4-fluorophenylboronic acid (104.9 mg, 0.75 mmol). Purified by flash chromatography (4.0 L hexane/ethyl acetate 0–60%); R_f = 0.20 (hexane/ethyl acetate 30%); Yield = 57% (53.3 mg, 0.28 mmol); Yellow solid; mp = 61–62 °C; FTIR (ATR) $\tilde{\nu}$ 3407, 3306, 3206, 3056, 3030, 2956, 2922, 2855, 1893, 1625, 1599, 1584, 1517, 1483, 1457, 1401, 1342, 1308, 1293, 1219, 1178, 1159, 1103, 991, 865, 831, 775, 719, 708, 682 cm^{-1}; ^{1}H NMR ($CDCl_3$, 400 MHz) δ 7.51–7.46 (m, 2H), δ 7.20 (t, *J* = 7.8 Hz, 1H), δ 7.11–7.05 (m, 2H), δ 6.91 (d, *J* = 7.8 Hz, 1H), δ 6.81 (t, *J* = 2.2 Hz, 1H), δ 6.64 (ddd, *J* = 7.8, 2.2, 1.5 Hz, 1H), δ 3.71 (br s, 2H); ^{19}F NMR ($CDCl_3$, 376 MHz) δ –115.8 ppm; ^{13}C NMR ($CDCl_3$, 100 MHz) δ 163.7, 161.2, 146.9, 141.5, 137.6, 137.5, 129.8, 128.7, 128.6, 117.5, 115.7, 115.4, 114.1, 113.7(74), 113.7(69) ppm.

This data is consistent with the literature.[6]

2-(4-Fluorophenyl)aniline (**8**)

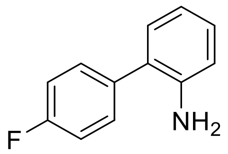

Prepared according to general *Procedure B*, starting with 2-chloroaniline (52.7 µL, 0.50 mmol) and 4-fluorophenylboronic acid (104.9 mg, 0.75 mmol). Purified by flash chromatography (4.0 L hexane/ethyl acetate 0–20%); R_f = 0.15 (hexane/ethyl acetate 10%); Yield = 34% (31.8 mg, 0.17 mmol); Light brown oil; FTIR (ATR) $\tilde{\nu}$ 3463, 3370, 3206, 3064, 3034, 2956, 2922, 2851, 1897, 1614, 1577, 1510, 1487, 1450, 1401, 1293, 1219, 1155, 1092, 1047, 1006, 965, 936, 835, 809, 749, 686 cm^{-1}; ^{1}H NMR ($CDCl_3$, 400 MHz) δ 7.46–7.41 (m, 2H), δ 7.20–7.10 (m, 4H), δ 6.84 (td, *J* = 7.5, 1.1 Hz, 1H), δ 6.78 (dd, *J* = 7.9, 0.8 Hz, 1H), δ 3.72 (br s, 2H); ^{19}F NMR ($CDCl_3$, 376 MHz) δ –115.2 ppm; ^{13}C NMR ($CDCl_3$, 100 MHz) δ 163.4, 160.9, 143.7, 135.5(4), 135.5(0), 130.9, 130.8, 130.5(73), 130.5(67), 128.8, 126.7, 118.8, 115.9, 115.8, 115.7 ppm.

This data is consistent with the literature.[7]

4-(4-Fluorophenyl)anisole (**9**)

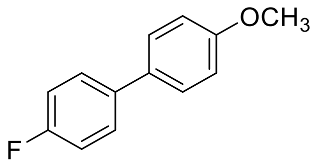

Prepared according to general *Procedure B*, starting with 4-chloroanisole (61.2 µL, 0.50 mmol) and 4-fluorophenylboronic acid (104.9 mg, 0.75 mmol). Purified by flash chromatography (4.0 L hexane/ethyl acetate 0–50%); R_f = 0.30 (hexane/ethyl acetate 5%); Yield = 36% (36.7 mg, 0.18 mmol); Off-white solid; mp = 86–89 °C; FTIR (ATR) $\tilde{\nu}$ 3068, 3042, 3015, 2963, 2922, 2844, 1893, 1871, 1733, 1655, 1595, 1573, 1491, 1457, 1439, 1398, 1327, 1308, 1290, 1267, 1256, 1230, 1182, 1159, 1133, 1118, 1100, 1036, 1010, 954, 824, 809, 790, 704, 660 cm^{-1}; ^{1}H NMR ($CDCl_3$, 400 MHz) δ 7.53–7.47 (m, 4H), δ 7.14–7.09 (m, 2H), δ 7.01–6.97 (m, 2H), δ 3.86 (s, 3H); ^{19}F NMR ($CDCl_3$, 376 MHz) δ –116.7 ppm; ^{13}C NMR ($CDCl_3$, 100 MHz) δ 163.4, 161.0, 159.3, 137.1(1), 137.0(8), 133.0, 128.4, 128.3, 128.1(5), 128.1(4), 115.8, 115.5, 114.4, 55.5 ppm.

This data is consistent with the literature.[8]

3-(4-Fluorophenyl)anisole (**10**)

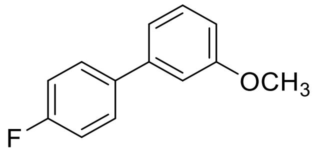

Prepared according to general *Procedure B*, starting with 3-chloroanisole (61.2 µL, 0.50 mmol) and 4-fluorophenylboronic acid (104.9 mg, 0.75 mmol). Purified by flash chromatography (4.0 L hexane/ethyl acetate 0–15%); R_f = 0.25 (hexane/ethyl acetate 5%); Yield = 29% (29.8 mg, 0.15 mmol); Light brown oil; FTIR (ATR) $\tilde{\nu}$ 3053, 3001, 2956, 2933, 2855, 2837, 1890, 1729, 1599, 1577, 1513, 1480, 1435, 1398, 1319, 1297, 1264, 1211, 1170, 1159, 1096, 1051, 1029, 1014, 995, 965, 868, 857, 831, 805, 775, 719, 693 cm^{-1}; ^{1}H NMR ($CDCl_3$, 400 MHz) δ 7.58–7.53 (m, 2H), δ 7.36 (t, *J* = 7.9 Hz, 1H), δ 7.16–7.08 (m, 4H), δ 6.91 (ddd, *J* = 7.9, 1.9, 0.6 Hz, 1H), δ 3.87 (s, 3H); ^{19}F NMR ($CDCl_3$, 376 MHz) δ –115.6 ppm; ^{13}C NMR ($CDCl_3$, 100 MHz) δ 163.9, 161.4, 160.1, 141.9, 137.4, 137.3, 130.0, 128.9, 128.8, 119.6(62), 119.6(57), 115.8, 115.6, 113.0(2), 113.0(1), 112.7, 55.4 ppm.

This data is consistent with the literature.[9]

2-(4-Fluorophenyl)anisole (**11**)

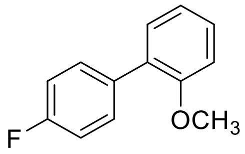

Prepared according to general *Procedure B*, starting with 2-chloroanisole (63.5 µL, 0.50 mmol) and 4-fluorophenylboronic acid (104.9 mg, 0.75 mmol). Purified by flash chromatography (4.0 L hexane/ethyl acetate 0–2%); R_f = 0.10 (hexane); Yield = 13% (12.8 mg, 0.06 mmol); Light yellow oil; FTIR (ATR) $\tilde{\nu}$ 3068, 3027, 3001, 2956, 2933, 2837, 1893, 1685, 1595, 1577, 1513, 1483, 1457, 1435, 1401, 1297, 1256, 1234, 1219, 1178, 1159, 1122, 1092, 1055, 1025, 1006, 932, 854, 831, 790, 745, 716, 690 cm^{-1}; ^{1}H NMR ($CDCl_3$, 400 MHz) δ 7.52–7.49 (m, 2H), δ 7.35–7.29 (m, 2H), δ 7.12–7.08 (m, 2H), δ 7.06–6.99 (m, 2H), δ 3.82 (s, 3H); ^{19}F NMR ($CDCl_3$, 376 MHz) δ –116.0 ppm; ^{13}C NMR ($CDCl_3$, 100 MHz) δ 163.4, 160.9, 156.5, 134.6, 134.5, 131.3, 131.2, 130.9, 129.8, 128.9, 121.0, 115.1, 114.9, 111.4, 55.7 ppm.

This data is consistent with the literature.[8,10]

4-(4-Fluorophenyl)acetophenone (**12**)

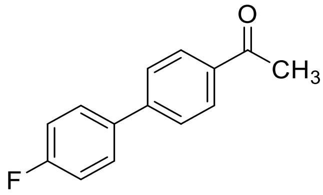

Prepared according to general *Procedure B*, starting with 4-chloroacetophenone (64.8 µL, 0.50 mmol) and 4-fluorophenylboronic acid (104.9 mg, 0.75 mmol). Purified by flash chromatography (4.0 L hexane/ethyl acetate 0–30%); R_f = 0.20 (hexane/ethyl acetate 15%); Yield = 85% (91.1 mg, 0.43 mmol); White solid; mp = 103–104 °C; FTIR (ATR) $\tilde{\nu}$ 3343, 3045, 2960, 2922, 2855, 1897, 1681, 1595, 1562, 1524, 1491, 1420, 1394, 1357, 1323, 1278, 1249, 1193, 1159, 1129, 1100, 1081, 1014, 1003, 958, 839, 816, 731, 704 cm^{-1}; ^{1}H NMR ($CDCl_3$, 400 MHz) δ 8.02 (d, J = 8.2 Hz, 2H), δ 7.63 (d, J = 8.2 Hz, 2H), δ 7.60–7.57 (m, 2H), δ 7.17–7.13 (m, 2H), δ 2.63 (s, 3H); ^{19}F NMR ($CDCl_3$, 376 MHz) δ –114.0 ppm; ^{13}C NMR ($CDCl_3$, 100 MHz) δ 197.8, 164.3, 161.8, 144.8, 136.1, 136.0, 135.9, 129.0(7), 129.0(6), 129.0, 127.1, 116.1, 115.9, 26.8 ppm.

This data is consistent with the literature.[11]

3-(4-Fluorophenyl)acetophenone (**13**)

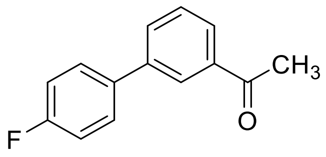

Prepared according to general *Procedure B*, starting with 3-chloroacetophenone (64.9 µL, 0.50 mmol) and 4-fluorophenylboronic acid (104.9 mg, 0.75 mmol). Purified by flash chromatography (4.0 L hexane/ethyl acetate 0–20%); R_f = 0.15 (hexane/ethyl acetate 10%); Yield = 84% (89.9 mg, 0.42 mmol); Yellow oil; FTIR (ATR) $\tilde{\nu}$ 3355, 3064, 3004, 2960, 2922, 2851, 1893, 1733, 1681, 1595, 1580, 1513, 1480, 1435, 1398, 1357, 1297, 1230, 1159, 1100, 1040, 1014, 962, 913, 839, 794, 719, 693 cm^{-1}; ^{1}H NMR ($CDCl_3$, 400 MHz) δ 8.13 (t, *J* = 1.7 Hz, 1H), δ 7.92 (dt, *J* = 7.7, 1.3 Hz, 1H), δ 7.73 (ddd, *J* = 7.7, 1.7, 1.3 Hz, 1H), δ 7.59–7.51 (m, 3H), δ 7.17–7.12 (m, 2H), δ 2.65 (s, 3H); ^{19}F NMR ($CDCl_3$, 376 MHz) δ –114.8 ppm; ^{13}C NMR ($CDCl_3$, 100 MHz) δ 198.1, 164.1, 161.6, 140.8, 137.8, 136.4(2), 136.3(8), 131.6(5), 131.6(4), 129.2, 128.9(5), 128.8(7), 127.3, 126.8, 116.0, 115.8, 26.8 ppm.

This data is consistent with the literature.[12]

2-(4-Fluorophenyl)acetophenone (**14**)

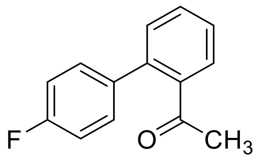

Prepared according to general *Procedure B*, starting with 2-chloroacetophenone (65.0 µL, 0.50 mmol) and 4-fluorophenylboronic acid (104.9 mg, 0.75 mmol). Purified by flash chromatography (4.0 L hexane/ethyl acetate 0–10%); R_f = 0.15 (hexane/ethyl acetate 5%); Yield = 39% (41.4 mg, 0.19 mmol); Light yellow oil; FTIR (ATR) $\tilde{\nu}$ 3358, 3060, 2926, 2855, 1733, 1685, 1603, 1565, 1510, 1476, 1442, 1424, 1353, 1286, 1267, 1223, 1159, 1114, 1096, 1077, 1040, 1006, 954, 880, 839, 820, 764, 719, 678 cm^{-1}; ^{1}H NMR ($CDCl_3$, 400 MHz) δ 7.55 (dd, *J* = 7.5, 1.3 Hz, 1H), δ 7.50 (td, *J* = 7.5, 1.3 Hz, 1H), δ 7.42 (td, *J* = 7.5, 1.3 Hz, 1H), δ 7.73 (dd, *J* = 7.5, 1.3 Hz, 1H), δ 7.33–7.28 (m, 2H), δ 7.14–7.09 (m, 2H), δ 2.05 (s, 3H); ^{19}F NMR ($CDCl_3$, 376 MHz) δ –114.3 ppm; ^{13}C NMR ($CDCl_3$, 100 MHz) δ 204.5, 164.0, 161.5, 140.9, 139.5, 136.9(0), 136.8(7), 130.9, 130.6, 130.5, 130.4(2), 130.4(1), 128.0, 127.7, 115.9, 115.7, 30.5 ppm.

This data is consistent with the literature.[13]

4-(4-Fluorophenyl)benzaldehyde (**15**)

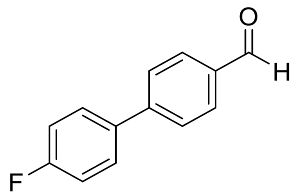

Prepared according to general *Procedure B*, starting with 4-chlorobenzaldehyde (70.3 mg, 0.50 mmol) and 4-fluorophenylboronic acid (104.9 mg, 0.75 mmol). Purified by flash chromatography (4.0 L hexane/ethyl acetate 0–30%); R_f = 0.25 (hexane/ethyl acetate 15%); Yield = 63% (62.6 mg, 0.31 mmol); Pale yellow solid; mp = 77–78 °C; FTIR (ATR) $\tilde{\nu}$ 3358, 3068, 2956, 2922, 2840, 2814, 2740, 1681, 1599, 1565, 1517, 1495, 1428, 1387, 1316, 1293, 1226, 1170, 1159, 1100, 1006, 839, 805, 727, 697 cm^{-1}; ^{1}H NMR ($CDCl_3$, 400 MHz) δ 10.04 (s, 1H), δ 7.95–7.93 (m, 2H), δ 7.71–7.68 (m, 2H), δ 7.62–7.57 (m, 2H), δ 7.19–7.13 (m, 2H); ^{19}F NMR ($CDCl_3$, 376 MHz) δ –113.5 ppm; ^{13}C NMR ($CDCl_3$, 100 MHz) δ 191.9, 164.4, 162.0, 146.2, 135.9(1), 135.8(8), 135.2, 130.4, 129.2, 129.1, 127.6(02), 127.5(97), 116.2, 116.0 ppm.

This data is consistent with the literature.[14]

Ethyl 4-(4-fluorophenyl)benzoate (**16**)

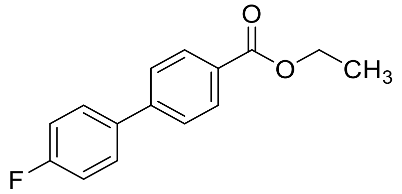

Prepared according to general *Procedure B*, starting with ethyl 4-chlorobenzoate (78.2 µL, 0.50 mmol) and 4-fluorophenylboronic acid (104.9 mg, 0.75 mmol). Purified by flash chromatography (4.0 L hexane/ethyl acetate 0–30%); R_f = 0.25 (hexane/ethyl acetate 5%); Yield = 86% (105.2 mg, 0.43 mmol); Off-white solid; mp = 63–64 °C; FTIR (ATR) $\tilde{\nu}$ 3407, 3045, 2989, 2941, 2904, 1890, 1715, 1595, 1565, 1524, 1491, 1461, 1424, 1394, 1364, 1319, 1267, 1237, 1185, 1163, 1103, 1021, 1003, 868, 827, 768, 716, 697 cm^{-1}; ^{1}H NMR ($CDCl_3$, 400 MHz) δ 8.11–8.09 (m, 2H), δ 7.60–7.54 (m, 4H), δ 7.17–7.11 (m, 2H), δ 4.40 (q, *J* = 7.1 Hz, 2H), δ 1.41 (t, *J* = 7.1 Hz, 3H); ^{19}F NMR ($CDCl_3$, 376 MHz) δ –114.2 ppm; ^{13}C NMR ($CDCl_3$, 100 MHz) δ 166.5, 164.2, 161.8, 144.5, 136.2, 130.2, 129.3, 129.0, 128.9, 126.9, 116.0, 115.8, 61.1, 14.4 ppm.

This data is consistent with the literature.[15]

4-(4-Fluorophenyl)benzeneacetonitrile (**17**)

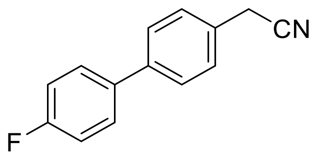

Prepared according to general *Procedure B*, starting with 4-chlorobenzeneacetonitrile (63.7 µL, 0.50 mmol) and 4-fluorophenylboronic acid (104.9 mg, 0.75 mmol). Purified by flash chromatography (4.0 L hexane/ethyl acetate 0–30%); R_f = 0.15 (hexane/ethyl acetate 15%); Yield = 45% (47.1 mg, 0.22 mmol); Off-white solid; mp = 105–106 °C; FTIR (ATR) $\tilde{\nu}$ 3071, 3038, 2956, 2922, 2855, 2251, 1893, 1655, 1603, 1528, 1495, 1428, 1401, 1304, 1249, 1204, 1163, 1133, 1100, 1006, 950, 936, 909, 839, 805, 794, 716, 704, 678 cm^{-1}; ^{1}H NMR ($CDCl_3$, 400 MHz) δ 7.56–7.51 (m, 4H), δ 7.40 (d, J = 8.4 Hz, 2H), δ 7.17–7.11 (m, 2H), δ 3.79 (s, 2H); ^{19}F NMR ($CDCl_3$, 376 MHz) δ –115.1 ppm; ^{13}C NMR ($CDCl_3$, 100 MHz) δ 164.0, 161.5, 140.2, 136.5, 136.4, 129.0, 128.8, 128.7, 128.5, 127.8(0), 127.7(9), 117.9, 116.0, 115.8, 23.4 ppm; HRMS (ESI-TOF) *m/z* $[M+H]^+$ Calculated for $C_{14}H_{11}FN^+$ 212.0870, found 212.0865 (|Δ*m/z*|= 2.4 ppm).

3-(4-Fluorophenyl)pyridine (**18**)

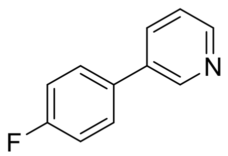

Prepared according to general *Procedure B*, starting with 3-chloropyridine (47.5 µL, 0.50 mmol) and 4-fluorophenylboronic acid (104.9 mg, 0.75 mmol). Purified by flash chromatography (4.0 L hexane/ethyl acetate 0–70%); R_f = 0.15 (hexane/ethyl acetate 40%); Yield = 88% (76.2 mg, 0.44 mmol); Yellow oil; FTIR (ATR) $\tilde{\nu}$ 3038, 2922, 2851, 1890, 1655, 1606, 1592, 1569, 1513, 1472, 1428, 1394, 1334, 1297, 1275, 1223, 1189, 1159, 1129, 1100, 1059, 1025, 1003, 954, 839, 801, 727, 708 cm^{-1}; ^{1}H NMR ($CDCl_3$, 400 MHz) δ 8.78 (s, 1H), δ 8.57 (d, J = 3.9 Hz, 1H), δ 7.79 (dt, J = 7.9, 1.9 Hz, 1H), δ 7.53–7.48 (m, 2H), δ 7.33 (dd, J = 7.7, 4.9 Hz, 1H), δ 7.17–7.11 (m, 2H); ^{19}F NMR ($CDCl_3$, 376 MHz) δ –114.2 ppm; ^{13}C NMR ($CDCl_3$, 100 MHz) δ 164.2, 161.7, 148.5, 148.2, 135.7, 134.2, 133.9(9), 133.9(6), 128.9, 128.8, 123.6, 116.2, 116.0 ppm.

This data is consistent with the literature.[9]

6-(4-Fluorophenyl)piperonyl alcohol (**19**)

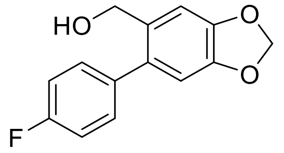

Prepared according to general *Procedure B*, starting with 6-chloropiperonyl alcohol (93.3 mg, 0.50 mmol) and 4-fluorophenylboronic acid (104.9 mg, 0.75 mmol). Purified by flash chromatography (4.0 L hexane/ethyl acetate 0–50%); R_f = 0.20 (hexane/ethyl acetate 30%); Yield = 15% (18.6 mg, 0.08 mmol); Light yellow solid; mp = 87–91 °C; FTIR (ATR) $\tilde{\nu}$ 3291, 3060, 3015, 2892, 2866, 2773, 1856, 1621, 1603, 1513, 1498, 1480, 1442, 1428, 1401, 1364, 1342, 1301, 1275, 1223, 1159, 1129, 1081, 1036, 1006, 962, 932, 880, 865, 850, 827, 813, 783, 768, 738, 693, 671 cm^{-1}; ^{1}H NMR ($CDCl_3$, 400 MHz) δ 7.32–7.27 (m, 2H), δ 7.11–7.06 (m, 2H), δ 7.01 (s, 1H), δ 6.73 (s, 1H), δ 5.99 (s, 2H), δ 4.46 (d, J = 3.0 Hz, 2H), δ 1.54 (t, J = 4.1 Hz, 1H); ^{19}F NMR ($CDCl_3$, 376 MHz) δ –115.4 ppm; ^{13}C NMR ($CDCl_3$, 100 MHz) δ 163.5, 161.1, 147.4, 147.1, 136.6, 136.5, 134.5, 131.9(6), 131.9(5), 131.1, 131.0, 115.4, 115.2, 110.3(0), 110.2(9), 109.0, 101.4, 63.1 ppm; HRMS (ESI-TOF) *m/z* $[M+H]^+$ Calculated for $C_{14}H_{12}FO_3^+$ 247.0765, found 247.0764 (|Δ*m/z*|= 0.4 ppm).

Also produced the byproduct below:

6-(4-Fluorophenyl)piperonyl aldehyde

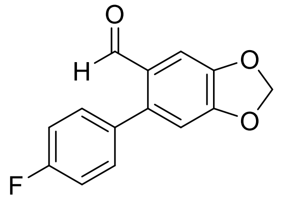

Purified by flash chromatography (4.0 L hexane/ethyl acetate 0–50%); R_f = 0.35 (hexane/ethyl acetate 30%); Yield = 3% (3.5 mg, 0.01 mmol); Light yellow solid; mp = 109–113 °C; FTIR (ATR) $\tilde{\nu}$ 3317, 3213, 3101, 3068, 3053, 2922, 2896, 2851, 2635, 1901, 1737, 1662, 1603, 1506, 1472, 1428, 1416, 1364, 1297, 1264, 1219, 1204, 1159, 1122, 1088, 1025, 973, 921, 887, 876, 854, 835, 813, 786, 734, 719, 697, 675 cm^{-1}; ^{1}H NMR ($CDCl_3$, 400 MHz) δ 9.73 (s, 1H), δ 7.46 (s, 1H), δ 7.33–7.29 (m, 2H), δ 7.17–7.11 (m, 2H), δ 6.81 (s, 1H), δ 6.10 (s, 2H); ^{19}F NMR ($CDCl_3$, 376 MHz) δ –113.7 ppm; ^{13}C NMR ($CDCl_3$, 100 MHz) δ 190.4, 164.1, 161.7, 152.3, 148.1, 142.6, 133.7(2), 133.6(8), 131.9, 131.8, 129.1, 115.7, 115.5, 110.4, 106.6, 102.3 ppm; HRMS (ESI-TOF) *m/z* $[M+H]^+$ Calculated for $C_{14}H_{10}FO_3^+$ 245.0608, found 245.0607 (|Δ*m/z*|= 0.4 ppm).

4’-(4-Fluorophenyl)chalcone (**20**)

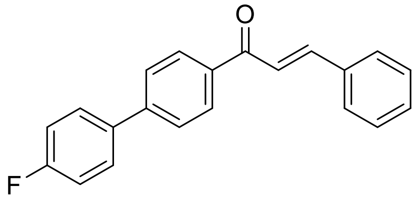

Prepared according to general *Procedure B*, starting with 4’-chlorochalcone (121.4 mg, 0.50 mmol) and 4-fluorophenylboronic acid (104.9 mg, 0.75 mmol). Purified by flash chromatography (4.0 L hexane/ethyl acetate 0–15%); R_f = 0.35 (hexane/ethyl acetate 20%); Yield = 11% (17.1 mg, 0.06 mmol); Yellow solid; mp = 146–150 °C; FTIR (ATR) $\tilde{\nu}$ 3053, 3027, 2956, 2926, 2855, 1893, 1718, 1685, 1655, 1592, 1573, 1521, 1491, 1446, 1420, 1398, 1338, 1308, 1290, 1252, 1215, 1193, 1159, 1126, 1092, 1032, 1010, 999, 984, 924, 891, 839, 820, 764, 734, 716, 693, 671 cm^{-1}; ^{1}H NMR ($CDCl_3$, 400 MHz) δ 8.12–8.09 (m, 2H), δ 7.85 (d, J = 15.7 Hz, 1H), δ 7.69–7.59 (m, 7H), δ 7.44–7.42 (m, 3H), δ 7.20–7.14 (m, 2H); ^{19}F NMR ($CDCl_3$, 376 MHz) δ –114.1 ppm; ^{13}C NMR ($CDCl_3$, 100 MHz) δ 190.0, 164.4, 161.9, 145.0, 144.6, 137.1, 136.2(3), 136.2(0), 135.1, 130.7, 129.3, 129.1, 129.0, 128.6, 127.2(8), 127.2(7), 122.1, 116.2, 116.0 ppm; HRMS (ESI-TOF) *m/z* [M+H]$^+$ Calculated for $C_{21}H_{16}FO^+$ 303.1180, found 303.1180 (|Δ*m/z*|= 0 ppm).

4-(3-Fluorophenyl)acetophenone (**21**)

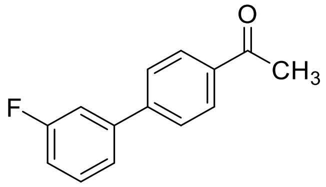

Prepared according to general *Procedure B*, starting with 4-chloroacetophenone (64.8 µL, 0.50 mmol) and 3-fluorophenylboronic acid (104.9 mg, 0.75 mmol). Purified by flash chromatography (4.0 L hexane/ethyl acetate 0–20%); R_f = 0.15 (hexane/ethyl acetate 10%); Yield = 87% (93.1 mg, 0.43 mmol); White solid; mp = 92–93 °C; FTIR (ATR) $\tilde{\nu}$ 3343, 3071, 3012, 2922, 1681, 1599, 1584, 1562, 1521, 1476, 1439, 1424, 1398, 1360, 1323, 1267, 1200, 1185, 1163, 1122, 1081, 1021, 1010, 999, 958, 906, 876, 846, 831, 790, 757, 731, 682, 671 cm^{-1}; ^{1}H NMR ($CDCl_3$, 400 MHz) δ 8.01 (d, J = 8.4 Hz, 2H), δ 7.63 (d, J = 8.4 Hz, 2H), δ 7.44–7.37 (m, 2H), δ 7.29 (td, J = 9.9, 2.1 Hz, 2H), δ 7.10–7.05 (m, 1H), δ 2.62 (s, 3H); ^{19}F NMR ($CDCl_3$, 376 MHz) δ –112.5 ppm; ^{13}C NMR ($CDCl_3$, 100 MHz) δ 197.6, 164.5, 162.0, 144.4, 144.3, 142.2, 142.1, 136.4, 130.6, 130.5, 129.0, 127.2, 123.0, 122.9, 115.2, 115.0, 114.3, 114.1, 26.7 ppm.

This data is consistent with the literature.[11]

4-(2-Fluorophenyl)acetophenone (**22**)

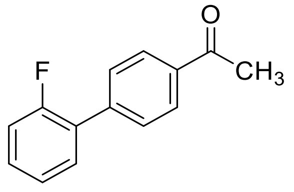

Prepared according to general *Procedure B*, starting with 4-chloroacetophenone (64.8 µL, 0.50 mmol) and 2-fluorophenylboronic acid (104.9 mg, 0.75 mmol). Purified by flash chromatography (4.0 L hexane/ethyl acetate 0–20%); R_f = 0.15 (hexane/ethyl acetate 10%); Yield = 81% (86.7 mg, 0.40 mmol); Off-white solid; mp = 85–86 °C; FTIR (ATR) $\tilde{\nu}$ 3340, 3075, 3042, 3004, 2956, 2922, 2855, 1674, 1618, 1599, 1580, 1558, 1510, 1480, 1454, 1424, 1401, 1353, 1305, 1293, 1264, 1252, 1208, 1155, 1103, 1073, 1036, 1006, 954, 857, 835, 820, 757, 719, 663 cm^{-1}; ^{1}H NMR ($CDCl_3$, 400 MHz) δ 8.01 (d, J = 8.4 Hz, 2H), δ 7.63 (d, J = 8.4 Hz, 2H), δ 7.44–7.37 (m, 2H), δ 7.29 (td, J = 9.9, 2.1 Hz, 2H), δ 7.10–7.05 (m, 1H), δ 2.62 (s, 3H); ^{19}F NMR ($CDCl_3$, 376 MHz) δ –117.4 ppm; ^{13}C NMR ($CDCl_3$, 100 MHz) δ 197.7, 161.0, 158.5, 140.5(8), 140.5(6), 136.2, 130.7, 130.6, 130.0, 129.9, 129.2(4), 129.2(1), 128.5, 128.0, 127.8, 124.6(3), 124.5(9), 116.4, 116.2, 26.7 ppm.

This data is consistent with the literature.[16]

4-(2-Naphthyl)acetophenone (**23**)

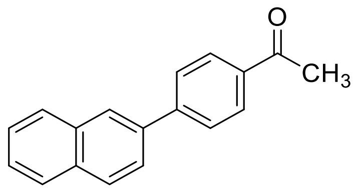

Prepared according to general *Procedure B*, starting with 4-chloroacetophenone (64.8 µL, 0.50 mmol) and 2-naphthylboronic acid (129.0 mg, 0.75 mmol). Purified by flash chromatography (4.0 L hexane/ethyl acetate 0–20%); R_f = 0.15 (hexane/ethyl acetate 10%); Yield = 74% (90.9 mg, 0.37 mmol); Off-white solid; mp = 135–136 °C; FTIR (ATR) $\tilde{\nu}$ 3340, 3060, 3042, 3001, 2922, 2855, 1674, 1595, 1558, 1498, 1461, 1431, 1401, 1357, 1301, 1252, 1178, 1144, 1129, 1111, 1073, 1014, 958, 902, 868, 857, 816, 775, 753, 656 cm^{-1}; ^{1}H NMR ($CDCl_3$, 400 MHz) δ 8.08–8.05 (m, 3H), δ 7.94–7.87 (m, 3H), δ 7.81–7.78 (m, 2H), δ 7.76–7.74 (m, 1H), δ 7.56–7.50 (m, 2H), δ 2.64 (m, 3H); ^{13}C NMR ($CDCl_3$, 100 MHz) δ 197.8, 145.7, 137.2, 135.9, 133.6, 133.1, 129.0, 128.8, 128.4, 127.8, 127.5, 126.6(3), 126.5(6), 126.4, 125.2, 26.7 ppm.

This data is consistent with the literature.[17]

4-(4-Isopropoxyphenyl)acetophenone (**24**)

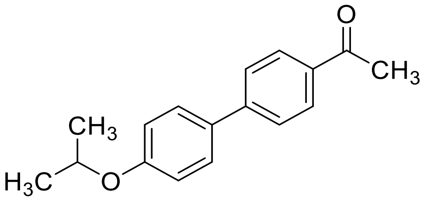

Prepared according to general *Procedure B*, starting with 4-chloroacetophenone (64.8 μL, 0.50 mmol) and 4-isopropoxyphenylboronic acid (135.0 mg, 0.75 mmol). Purified by flash chromatography (4.0 L hexane/ethyl acetate 0–20%); R_f = 0.15 (hexane/ethyl acetate 10%); Yield = 46% (57.9 mg, 0.23 mmol); White solid; mp = 145–146 °C; FTIR (ATR) $\tilde{\nu}$ 3332, 3187, 3090, 3042, 2993, 2974, 2922, 1670, 1592, 1554, 1521, 1491, 1469, 1420, 1401, 1383, 1360, 1323, 1308, 1286, 1271, 1249, 1196, 1137, 1122, 1107, 1077, 1025, 999, 950, 865, 820, 809, 757, 734, 712, 656 cm^{-1}; ^{1}H NMR ($CDCl_3$, 400 MHz) δ 8.00–7.98 (m, 2H), δ 7.64–7.62 (m, 2H), δ 7.57–7.53 (m, 2H), δ 6.99–6.96 (m, 2H), δ 4.60 (hept, *J* = 6.0 Hz, 1H), δ 2.61 (s, 3H), δ 1.37 (d, *J* = 6.0 Hz, 6H); ^{13}C NMR ($CDCl_3$, 100 MHz) δ 197.7, 158.4, 145.5, 135.3, 132.0, 129.0, 128.4, 126.6, 116.3, 70.1, 26.7, 22.1 ppm; HRMS (ESI-TOF) *m/z* $[M+H]^+$ Calculated for $C_{17}H_{19}O_2^+$ 255.1380, found 255.1379 (|Δ*m/z*|= 0.4 ppm).

4-(3-Isopropoxyphenyl)acetophenone (**25**)

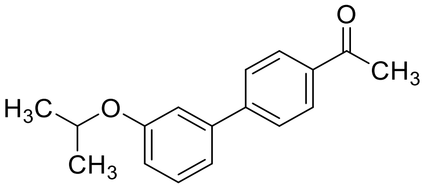

Prepared according to general *Procedure B*, starting with 4-chloroacetophenone (64.8 μL, 0.50 mmol) and 3-isopropoxyphenylboronic acid (135.0 mg, 0.75 mmol). Purified by flash chromatography (4.0 L hexane/ethyl acetate 0–15%); R_f = 0.20 (hexane/ethyl acetate 10%); Yield = 63% (80.2 mg, 0.32 mmol); White solid; mp = 87–88 °C; FTIR (ATR) $\tilde{\nu}$ 3332, 3071, 2974, 2933, 2870, 1674, 1588, 1558, 1510, 1480, 1465, 1435, 1398, 1383, 1360, 1327, 1301, 1267, 1215, 1163, 1137, 1114, 1047, 1014, 995, 977, 958, 872, 842, 827, 786, 753, 734, 693, 671 cm^{-1}; ^{1}H NMR ($CDCl_3$, 400 MHz) δ 8.02–8.00 (m, 2H), δ 7.67–7.65 (m, 2H), δ 7.35 (t, *J* = 7.9 Hz, 1H), δ 6.93 (dd, *J* = 7.9, 2.1 Hz, 1H), δ 4.63 (hept, *J* = 6.0 Hz, 1H), δ 2.62 (s, 3H), δ 1.38 (d, *J* = 6.0 Hz, 6H); ^{13}C NMR ($CDCl_3$, 100 MHz) δ 197.7, 158.4, 145.7, 141.4, 136.0, 130.0, 128.9, 127.2, 119.5, 115.3, 115.2, 70.0, 26.7, 22.1 ppm; HRMS (ESI-TOF) *m/z* $[M+H]^+$ Calculated for $C_{17}H_{19}O_2^+$ 255.1380, found 255.1380 (|Δ*m/z*|= 0).

4-(2-Isopropoxyphenyl)acetophenone (**26**)

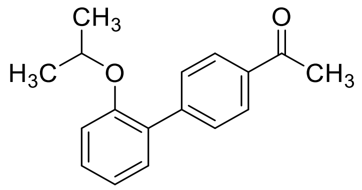

Prepared according to general *Procedure B*, starting with 4-chloroacetophenone (64.8 µL, 0.50 mmol) and 2-isopropoxyphenylboronic acid (135.0 mg, 0.75 mmol). Purified by flash chromatography (4.0 L hexane/ethyl acetate 0–20%); R_f = 0.10 (hexane/ethyl acetate 5%); Yield = 35% (44.4 mg, 0.17 mmol); Light yellow solid; mp = 75–77 °C; FTIR (ATR) $\tilde{\nu}$ 3340, 3090, 3071, 3034, 3001, 2974, 2922, 2855, 1737, 1674, 1599, 1580, 1510, 1480, 1450, 1420, 1401, 1387, 1375, 1357, 1334, 1297, 1256, 1245, 1185, 1137, 1122, 1073, 1059, 1021, 1003, 980, 947, 857, 827, 790, 753, 723, 671 cm^{-1}; ^{1}H NMR ($CDCl_3$, 400 MHz) δ 8.01–7.98 (m, 2H), δ 7.68–7.65 (m, 2H), δ 7.36–7.30 (m, 2H), δ 7.05–7.00 (m, 2H), δ 4.50 (hept, J = 6.0 Hz, 1H), δ 2.64 (s, 3H), δ 1.27 (d, J = 6.0 Hz, 6H); ^{13}C NMR ($CDCl_3$, 100 MHz) δ 198.1, 155.0, 144.1, 135.4, 131.0, 130.8, 129.9, 129.4, 128.0, 121.0, 115.0, 71.0, 26.7, 22.1 ppm.

This data is consistent with the literature.[18]

4-(4-Phenoxyphenyl)acetophenone (**27**)

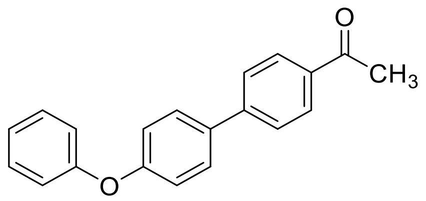

Prepared according to general *Procedure B*, starting with 4-chloroacetophenone (64.8 µL, 0.50 mmol) and 4-phenoxyphenylboronic acid (160.5 mg, 0.75 mmol). Purified by flash chromatography (4.0 L hexane/ethyl acetate 0–20%); R_f = 0.15 (hexane/ethyl acetate 10%); Yield = 62% (89.1 mg, 0.31 mmol); White solid; mp = 129–130 °C; FTIR (ATR) $\tilde{\nu}$ 3343, 3064, 3042, 1677, 1588, 1562, 1521, 1487, 1457, 1420, 1398, 1357, 1331, 1308, 1271, 1256, 1208, 1189, 1167, 1151, 1137, 1110, 1073, 1025, 999, 958, 898, 872, 820, 794, 764, 742, 712, 690, 656 cm^{-1}; ^{1}H NMR ($CDCl_3$, 400 MHz) δ 8.04–8.01 (m, 2H), δ 7.67–7.65 (m, 2H), δ 7.61–7.58 (m, 2H), δ 7.41–7.35 (m, 2H), δ 7.16 (t, J = 7.4 Hz, 1H), δ 7.11–7.07 (m, 4H), δ 2.63 (s, 3H); ^{13}C NMR ($CDCl_3$, 100 MHz) δ 197.6, 157.9, 156.8, 145.1, 135.7, 134.7, 129.9, 129.0, 128.7, 126.9, 123.8, 119.3, 119.0, 26.7 ppm.

This data is consistent with the literature.[16]

4-(4-Ethoxycarbonylphenyl)acetophenone (**28**)

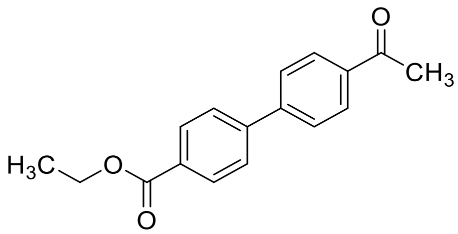

Prepared according to general *Procedure B*, starting with 4-chloroacetophenone (64.8 µL, 0.50 mmol) and 4-ethoxycarbonylphenylboronic acid (145.5 mg, 0.75 mmol). Purified by flash chromatography (4.0 L hexane/ethyl acetate 0–60%); R_f = 0.20 (hexane/ethyl acetate 30%); Yield = 48% (63.9 mg, 0.24 mmol); White solid; mp = 101–103 °C; FTIR (ATR) $\tilde{\nu}$ 3414, 3358, 3071, 2982, 2933, 2907, 1711, 1685, 1603, 1577, 1524, 1465, 1416, 1394, 1360, 1290, 1267, 1211, 1174, 1103, 1018, 1003, 958, 854, 827, 768, 727, 693 cm^{-1}; ^{1}H NMR ($CDCl_3$, 400 MHz) δ 8.10 (d, J = 8.2 Hz, 2H), δ 8.01 (d, J = 8.2 Hz, 2H), δ 7.66 (t, J = 8.6 Hz, 4H), δ 4.38 (q, J = 7.1 Hz, 2H), δ 2.61 (s, 3H), δ 1.39 (t, J = 7.1 Hz, 3H); ^{13}C NMR ($CDCl_3$, 100 MHz) δ 197.6, 166.3, 144.5, 144.1, 136.5, 130.2(1), 130.1(7), 129.0, 127.4, 127.2, 61.1, 26.7, 14.4 ppm.

This data is consistent with the literature.[19]

4-(4-Cyanophenyl)acetophenone (**29**)

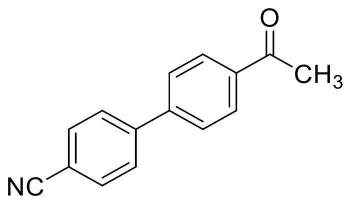

Prepared according to general *Procedure B*, starting with 4-chloroacetophenone (64.8 µL, 0.50 mmol) and 4-cyanophenylboronic acid (110.2 mg, 0.75 mmol). Purified by flash chromatography (4.0 L hexane/ethyl acetate 0–60%); R_f = 0.15 (hexane/ethyl acetate 30%); Yield = 54% (59.8 mg, 0.27 mmol); Light yellow solid; mp = 114–115 °C; FTIR (ATR) $\tilde{\nu}$ 3407, 3340, 3042, 2993, 2960, 2922, 2855, 2371, 2344, 2225, 1677, 1599, 1577, 1551, 1524, 1491, 1461, 1420, 1394, 1357, 1316, 1293, 1264, 1208, 1182, 1114, 1077, 1018, 1003, 954, 861, 831, 813, 742, 716 cm^{-1}; ^{1}H NMR ($CDCl_3$, 400 MHz) δ 8.07–8.05 (m, 2H), δ 7.77–7.70 (m, 4H), δ 7.69–7.67 (m, 2H), δ 2.64 (s, 3H); ^{13}C NMR ($CDCl_3$, 100 MHz) δ 197.6, 144.4, 143.6, 137.0, 132.8, 129.2, 128.0, 127.6, 118.7, 112.0, 26.8 ppm.

This data is consistent with the literature.[20]

4-(3,4-(Methylenedioxy)phenyl)acetophenone (**30**)

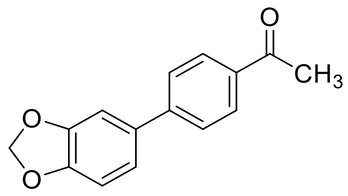

Prepared according to general *Procedure B*, starting with 4-chloroacetophenone (64.8 μL, 0.50 mmol) and 3,4-(methylenedioxy)phenylboronic acid (124.5 mg, 0.75 mmol). Purified by flash chromatography (4.0 L hexane/ethyl acetate 0–20%); R_f = 0.10 (hexane/ethyl acetate 10%); Yield = 53% (63.9 mg, 0.27 mmol); Off-white solid; mp = 132–134 °C; FTIR (ATR) $\tilde{\nu}$ 3336, 3075, 3042, 3001, 2956, 2904, 2781, 1674, 1599, 1558, 1517, 1498, 1476, 1439, 1428, 1405, 1357, 1338, 1305, 1286, 1267, 1245, 1223, 1185, 1148, 1107, 1032, 1025, 958, 932, 891, 872, 835, 801, 764, 734, 719, 678 cm^{-1}; ^{1}H NMR ($CDCl_3$, 400 MHz) δ 7.99 (d, J = 8.4 Hz, 2H), δ 7.59 (d, J = 8.4 Hz, 2H), δ 7.12–7.09 (m, 2H), δ 6.90 (d, J = 7.9 Hz, 1H), δ 6.01 (s, 2H), δ 2.62 (s, 3H); ^{13}C NMR ($CDCl_3$, 100 MHz) δ 197.7, 148.5, 148.0, 145.5, 135.6, 134.2, 129.0, 126.9, 121.2, 108.9, 107.7, 101.5, 26.7 ppm.

This data is consistent with the literature.[21]

4-(2-Furanyl)acetophenone (**31**)

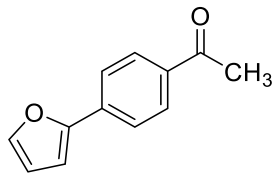

Prepared according to general *Procedure B*, starting with 4-chloroacetophenone (64.8 μL, 0.50 mmol) and 2-furanylboronic acid (83.9 mg, 0.75 mmol). Purified by flash chromatography (4.0 L hexane/ethyl acetate 0–20%); R_f = 0.15 (hexane/ethyl acetate 10%); Yield = 67% (62.2 mg, 0.33 mmol); White solid; mp = 103–104 °C; FTIR (ATR) $\tilde{\nu}$ 3325, 3209, 3142, 3105, 3045, 3001, 2922, 2855, 1666, 1603, 1558, 1510, 1476, 1435, 1413, 1353, 1297, 1275, 1260, 1223, 1185, 1163, 1152, 1118, 1077, 1044, 1018, 977, 958, 902, 880, 839, 813, 742, 663 cm^{-1}; ^{1}H NMR ($CDCl_3$, 400 MHz) δ 7.95 (d, J = 8.5 Hz, 2H), δ 7.71 (d, J = 8.5 Hz, 2H), δ 7.50 (d, J = 1.7 Hz, 1H), δ 6.77 (d, J = 3.3 Hz, 1H), δ 6.49 (dd, J = 3.3, 1.7 Hz, 1H), δ 2.58 (s, 3H); ^{13}C NMR ($CDCl_3$, 100 MHz) δ 197.3, 152.9, 143.3, 135.6, 134.9, 129.0, 123.6, 112.1, 107.5, 26.6 ppm.

This data is consistent with the literature.[22]

4-(3-Thianaphthenyl)acetophenone (**32**)

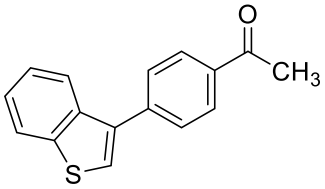

Prepared according to general *Procedure B*, starting with 4-chloroacetophenone (64.8 µL, 0.50 mmol) and 3-thianaphthenylboronic acid (133.5 mg, 0.75 mmol). Purified by flash chromatography (4.0 L hexane/ethyl acetate 0–20%); R_f = 0.15 (hexane/ethyl acetate 10%); Yield = 44% (55.6 mg, 0.22 mmol); Light purple solid; mp = 90–91 °C; FTIR (ATR) $\tilde{\nu}$ 3340, 3198, 3056, 2997, 1674, 1640, 1599, 1562, 1551, 1524, 1487, 1454, 1424, 1405, 1375, 1353, 1342, 1301, 1267, 1211, 1185, 1667, 1141, 1107, 1073, 1059, 1025, 1010, 984, 962, 939, 850, 831, 790, 764, 753, 727, 697 cm^{-1}; ^1H NMR ($CDCl_3$, 400 MHz) δ 8.09 (d, J = 8.4 Hz, 2H), δ 7.97–7.89 (m, 2H), δ 7.70 (d, J = 8.4 Hz, 2H), δ 7.50 (s, 1H), δ 7.45–7.39 (m, 2H), δ 2.67 (s, 3H); ^{13}C NMR ($CDCl_3$, 100 MHz) δ 197.8, 140.9, 137.5, 137.0, 136.1, 129.0, 128.8, 124.9, 124.8(2), 124.7(6), 123.2, 122.8, 26.8 ppm.

This data is consistent with the literature.[23]

3-(2-Furanyl)acetophenone (**33**)

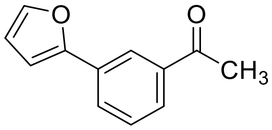

Prepared according to general *Procedure B*, starting with 3-chloroacetophenone (64.8 µL, 0.50 mmol) and 2-furanylboronic acid (83.9 mg, 0.75 mmol). Purified by flash chromatography (4.0 L hexane/ethyl acetate 0–10%); R_f = 0.20 (hexane/ethyl acetate 10%); Yield = 61% (56.7 mg, 0.30 mmol); Orange oil; FTIR (ATR) $\tilde{\nu}$ 3355, 3116, 3064, 3004, 2922, 1897, 1826, 1733, 1681, 1610, 1498, 1420, 1357, 1293, 1238, 1156, 1111, 1081, 1014, 965, 910, 887, 790, 734, 686 cm^{-1}; ^1H NMR ($CDCl_3$, 400 MHz) δ 8.25 (t, J = 1.9 Hz, 1H), 7.85 (t, J = 8.1 Hz, 2H), 7.50–7.46 (m, 2H), 6.75 (d, J = 3.4 Hz, 1H), 6.50 (dd, J = 3.4, 1.8 Hz, 1H), 2.65 (s, 3H); ^{13}C NMR ($CDCl_3$, 100 MHz) δ 198.0, 153.1, 142.7, 137.7, 131.5, 129.1, 128.2, 127.2, 123.7, 112.0, 106.1, 26.9 ppm.

Also produced the byproduct (2,2'-bifuran) below during the extrusion:

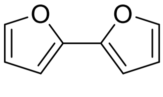

^1H NMR ($CDCl_3$, 400 MHz) δ 7.41 (d, J = 1.8 Hz, 2H), 6.55 (d, J = 3.3 Hz, 2H), 6.45 (dd, J = 3.3, 1.8 Hz, 2H).

This data is consistent with the literature.[24,25]

1-(3,4,5-Trifluorophenyl)naphthalene (**34**)

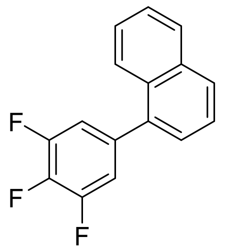

Prepared according to general *Procedure B*, starting with 1-chloronaphthalene (68.1 μL, 0.50 mmol) and 3,4,5-trifluorophenylboronic acid (131.9 mg, 0.75 mmol). Purified by flash chromatography (4.0 L hexane/ethyl acetate 0–2%); R_f = 0.25 (hexane); Yield = 9% (11.1 mg, 0.04 mmol); Colourless oil; FTIR (ATR) $\tilde{\nu}$ 3060, 2956, 2926, 2851, 1931, 1901, 1830, 1651, 1614, 1517, 1510, 1476, 1461, 1442, 1420, 1401, 1353, 1334, 1297, 1264, 1241, 1219, 1159, 1103, 1092, 1040, 1010, 969, 954, 913, 898, 861, 835, 801, 772, 734, 708, 667, 652 cm^{-1}; ^{1}H NMR ($CDCl_3$, 400 MHz) δ 7.94–7.89 (m, 2H), δ 7.82–7.80 (m, 1H), δ 7.55–7.47 (m, 3H), δ 7.37 (dd, J = 7.1, 1.1 Hz, 1H), δ 7.16–7.08 (m, 2H); ^{19}F NMR ($CDCl_3$, 376 MHz) δ –134.7 (d, J = 20.2 Hz, 2F), –162.5 (s, 1F) ppm; ^{13}C NMR ($CDCl_3$, 100 MHz) δ 152.5, 152.4(4), 152.3(8), 152.3, 149.9(9), 149.9(5), 149.8(9), 149.8(5), 140.6, 137.3, 137.2(4), 137.2(3), 136.9(1), 136.8(8), 136.8(3), 133.9, 131.2, 128.9, 128.7, 127.9, 127.1(24), 127.1(17), 126.8, 126.3, 125.4, 125.2, 114.4(3), 114.3(8), 114.2(8), 114.2(2) ppm.

3-(3-Thianaphthenyl)pyridine (**35**)

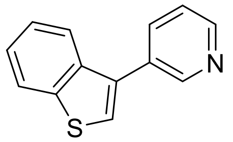

Prepared according to general *Procedure B*, starting with 3-chloropyridine (47.5 μL, 0.50 mmol) and 3-thianaphthenylboronic acid (133.5 mg, 0.75 mmol). Purified by flash chromatography (4.0 L hexane/ethyl acetate 0–2%); Yield = 84% (64.8 mg, 0.42 mmol); Red oil; FTIR (ATR) $\tilde{\nu}$ 3053, 1573, 1517, 1469, 1424, 1349, 1312, 1260, 1223, 1185, 1144, 1062, 1025, 939, 839, 809, 760, 712 cm^{-1}; ^{1}H NMR ($CDCl_3$, 400 MHz) δ 8.87 (s, 1H), 8.67 (s, 1H), 7.97–7.92 (m, 2H), 7.88–7.83 (m, 1H), 7.48 (s, 1H), 7.43 (dt, J = 6.2, 3.6 Hz, 3H); ^{13}C NMR ($CDCl_3$, 100 MHz) δ 149.4, 148.6, 140.7, 137.5, 135.7, 134.3, 131.8, 128.4, 124.7(0), 124.6(5), 123.5, 123.0, 122.3 ppm.

This data is consistent with the literature.[26]

3-(3-Fluorophenyl)isopropoxybenzene (**36**)

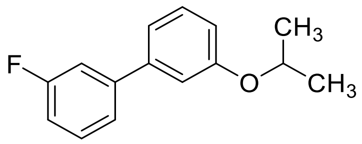

Prepared according to general *Procedure B*, starting with 3-fluorochlorobenzene (53.5 µL, 0.50 mmol) and 3-isopropoxyphenylboronic acid (135.0 mg, 0.75 mmol). Purified by flash chromatography (4.0 L hexane/ethyl acetate 0–2%); Yield = 73% (83.6 mg, 0.37 mmol); Colourless oil; FTIR (ATR) $\tilde{\nu}$ 3056, 2974, 2930, 1573, 1476, 1416, 1383, 1301, 1219, 1178, 115, 1029, 973, 910, 850, 775, 693 cm^{-1}; ^{1}H NMR ($CDCl_3$, 400 MHz) δ 7.33–7.26 (m, 2H), 7.24–7.17 (m, 2H), 7.06–7.01 (m, 2H), 6.95 (t, J = 7.4 Hz, 1H), 6.83–6.78 (m, 1H), 4.54 (hept, J = 5.8 Hz, 1H), 1.29 (d, J = 6.1 Hz, 6H); ^{19}F NMR ($CDCl_3$, 376 MHz) δ –113.2 ppm; ^{13}C NMR ($CDCl_3$, 100 MHz) δ 163.3 (d, J = 245.5 Hz), 158.5, 143.6 (d, J = 7.7 Hz), 141.6 (d, J = 2.2 Hz), 133.9 (d, J = 19.2 Hz), 130.3 (d, J = 8.4 Hz), 130.0, 128.7 (d, J = 7.4 Hz), 122.9 (d, J = 2.8 Hz), 119.5, 115.2 (d, J = 12.0 Hz), 114.2 (dd, J = 21.6, 6.2 Hz), 70.2, 22.2 ppm.

4-(2-Naphthalenyl)benzonitrile (**37**)

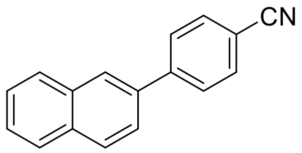

Prepared according to general *Procedure B*, starting with 2-chloronapthalene (67.8 µL, 0.50 mmol) and 4-cyanobenzeneboronic acid (110.2 mg, 0.75 mmol). Purified by flash chromatography (4.0 L hexane/ethyl acetate 0–2%); Yield = 51% (58.7 mg, 0.26 mmol); White solid; mp = 141–146 °C; FTIR (ATR) $\tilde{\nu}$ 3038, 1431, 1249, 1178, 1025, 839, 705 cm^{-1}; ^{1}H NMR ($CDCl_3$, 400 MHz) δ 8.06 (s, 1H), 7.96 (d, J = 8.5 Hz, 1H), 7.91 (td, J = 9.4, 3.9 Hz, 2H), 7.83–7.76 (m, 4H), 7.73–7.68 (m, 2H), 7.57–7.52 (m, 1H); ^{13}C NMR ($CDCl_3$, 100 MHz) δ 145.7, 136.5, 133.6, 133.3, 133.0, 132.8, 129.1, 128.5, 128.1, 128.0, 127.8, 126.9(1), 126.8(8), 126.7, 125.0, 119.1, 111.1 ppm.

This data is consistent with the literature.[27]

3-(4-Methoxyphenyl)pyridine (**38**)

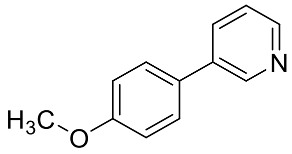

Prepared according to general *Procedure B*, starting with 3-chloropyridine (47.5 µL, 0.50 mmol) and 4-methoxyphenylboronic acid (114.0 mg, 0.75 mmol). Purified by flash chromatography (4.0 L hexane/ethyl acetate 0–2%); Yield = 87% (79.8 mg, 0.44 mmol); White solid; mp = 56–58 °C; ^{1}H NMR ($CDCl_3$, 400 MHz) δ 8.82 (s, 1H), 8.55 (d, J = 4.0 Hz, 1H), 7.85 (dt, J = 7.9, 1.9 Hz, 1H), 7.54–7.50 (m, 2H), 7.35 (dd, J = 7.8, 4.9 Hz, 1H), 7.03–7.00 (m, 2H), 3.86 (s, 3H). ^{13}C NMR ($CDCl_3$, 100 MHz) δ 160.0, 147.8, 147.6, 134.3, 133.6, 130.2, 128.4, 127.9, 123.8, 114.7, 114.3, 55.5 ppm.

This data is consistent with the literature.[28]

NMR Spectra

Figure S5. ^{1}H NMR Spectrum (400 MHz, $CDCl_3$) for 2-(4-fluorophenyl)naphthalene (**3**)

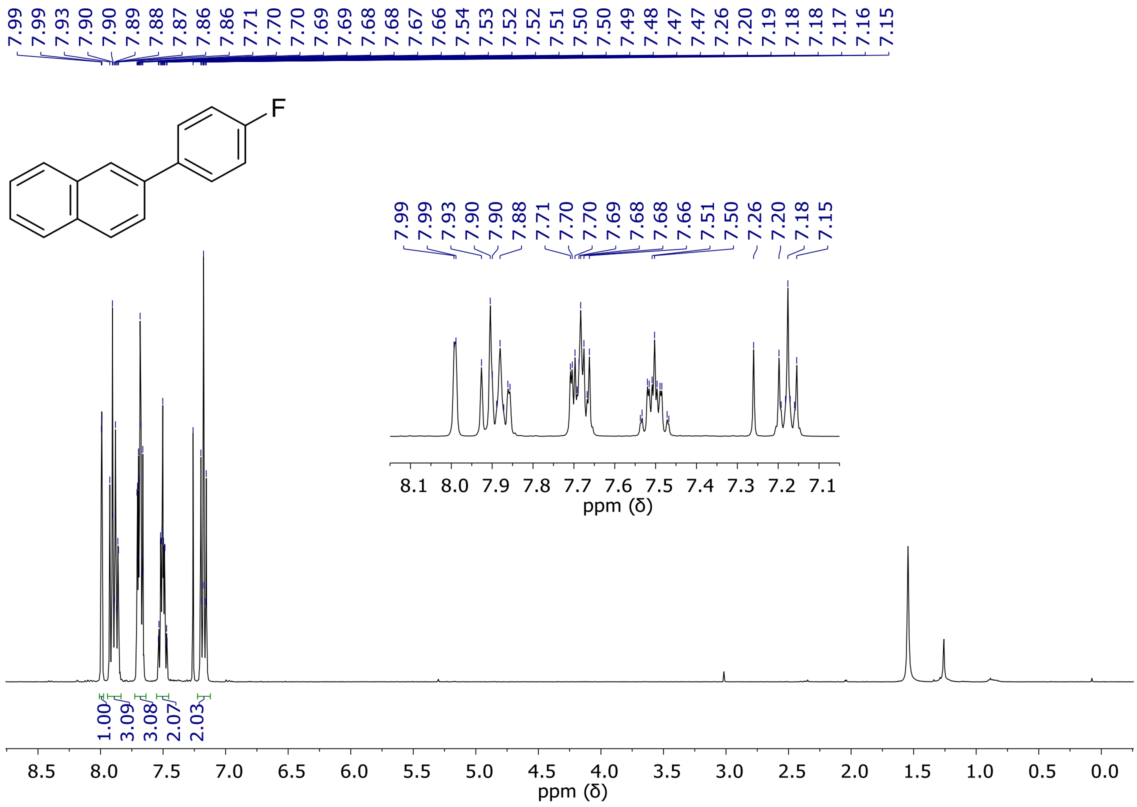

Figure S6. ^{13}C NMR Spectrum (100 MHz, $CDCl_3$) for 2-(4-fluorophenyl)naphthalene (**3**)

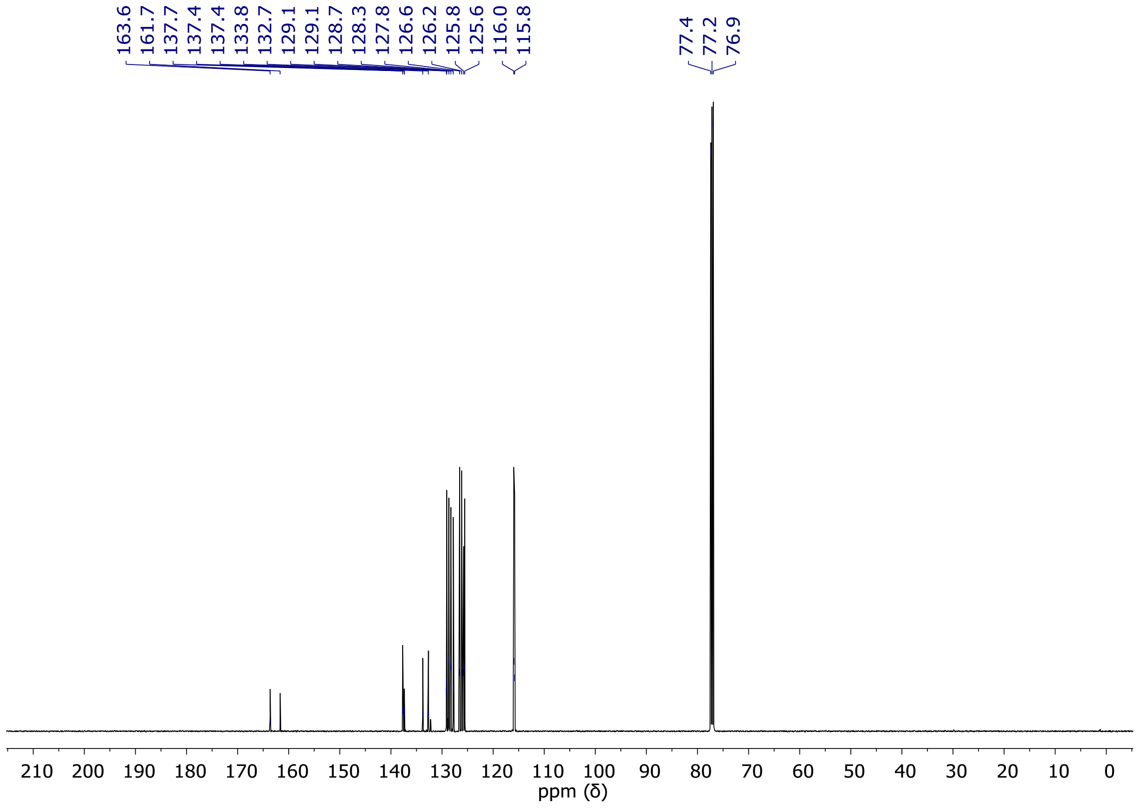

Figure S7. ^{1}H NMR Spectrum (400 MHz, $CDCl_3$) for 1-(4-fluorophenyl)naphthalene (**4**)

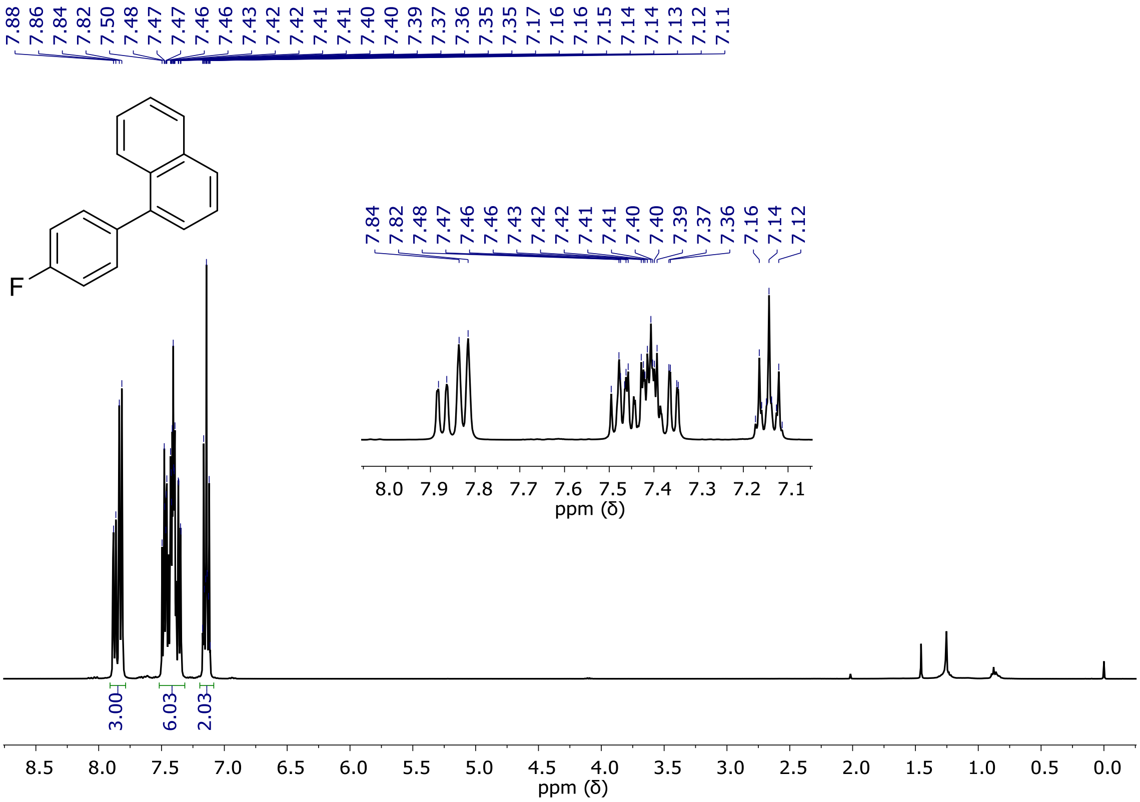

Figure S8. ^{13}C NMR Spectrum (100 MHz, $CDCl_3$) for 1-(4-fluorophenyl)naphthalene (**4**)

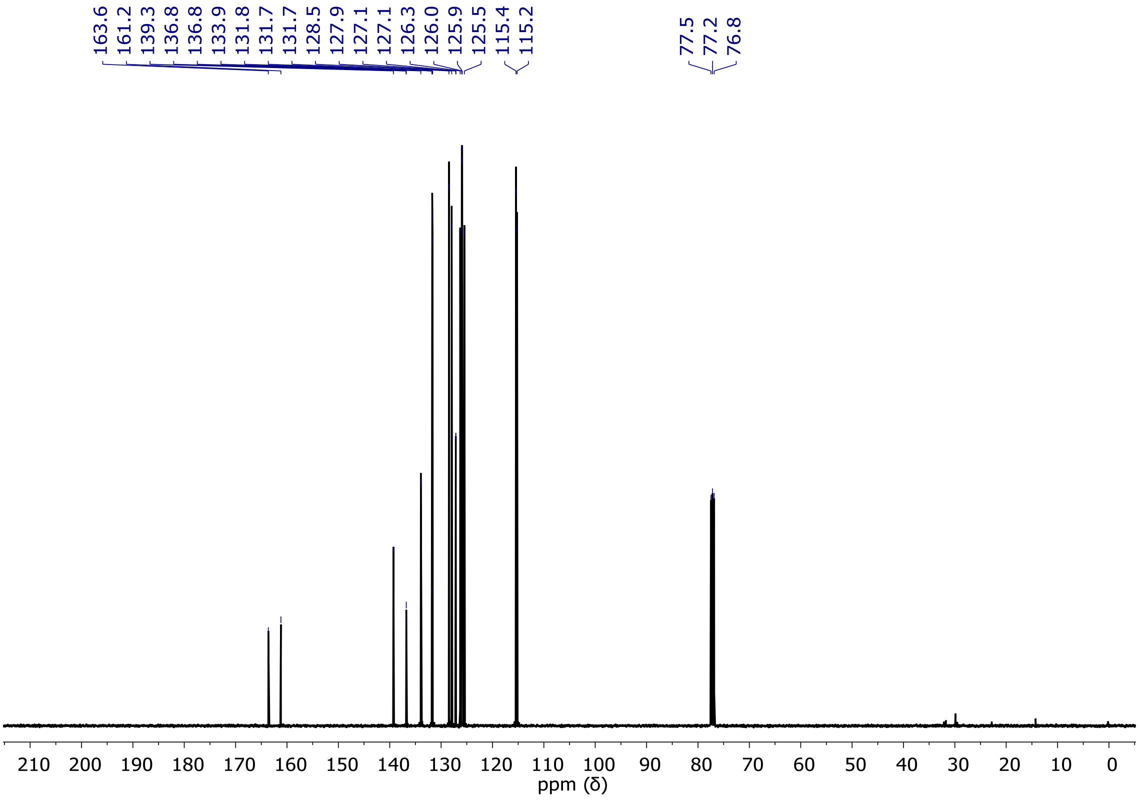

Figure S9. ^{19}F NMR Spectrum (376 MHz, $CDCl_3$) for 1-(4-fluorophenyl)naphthalene (**4**)

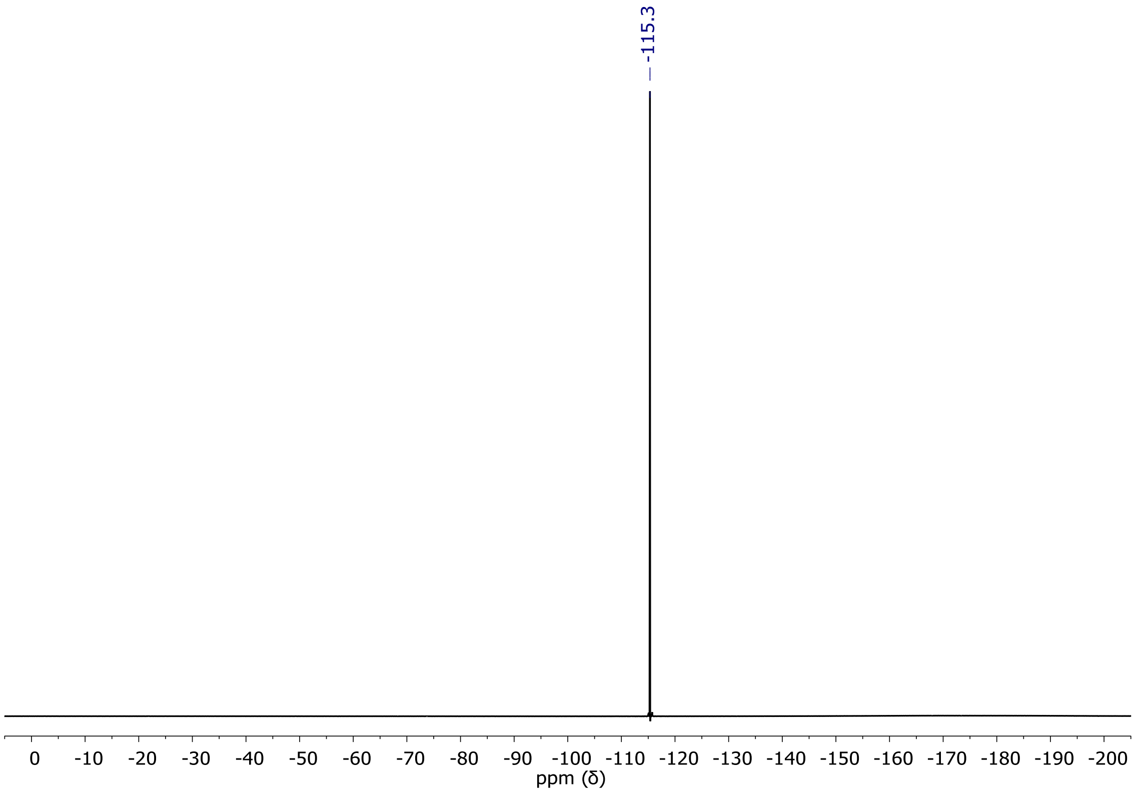

Figure S10. ^{1}H NMR Spectrum (400 MHz, $CDCl_3$) for 4-(4-fluorophenyl)toluene (**5**)

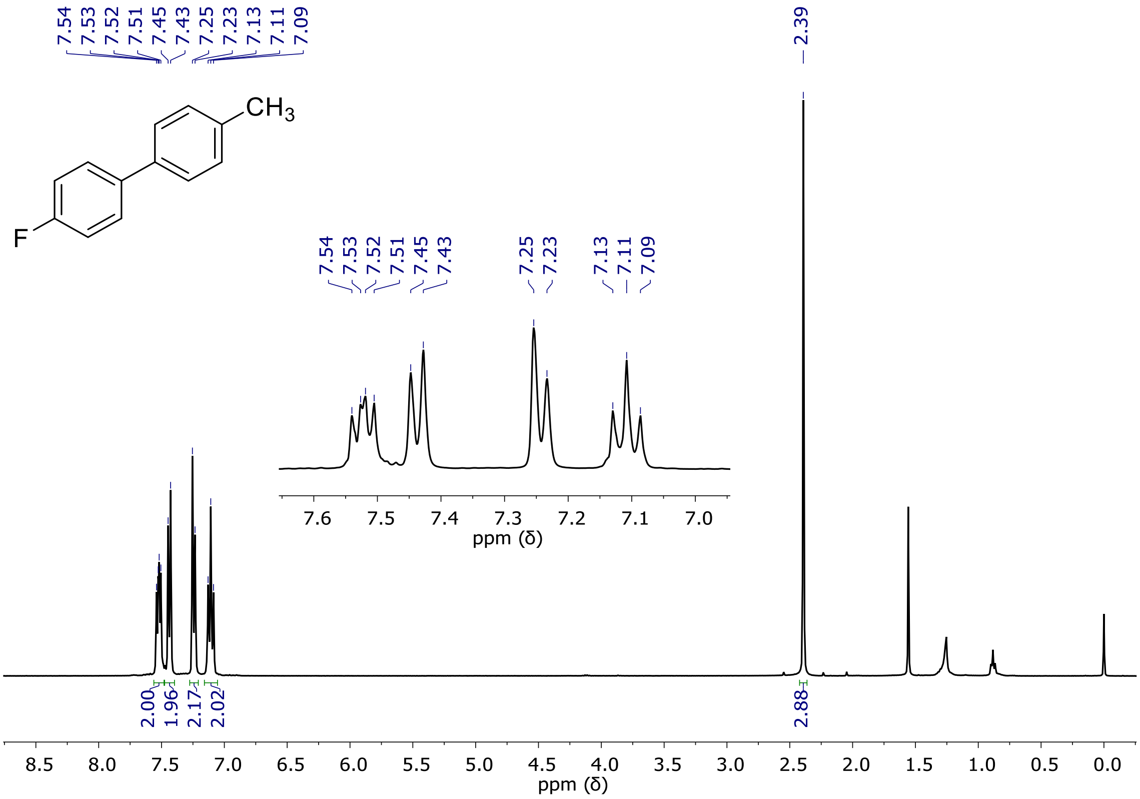

Figure S11. ^{13}C NMR Spectrum (100 MHz, $CDCl_3$) for 4-(4-fluorophenyl)toluene (**5**)

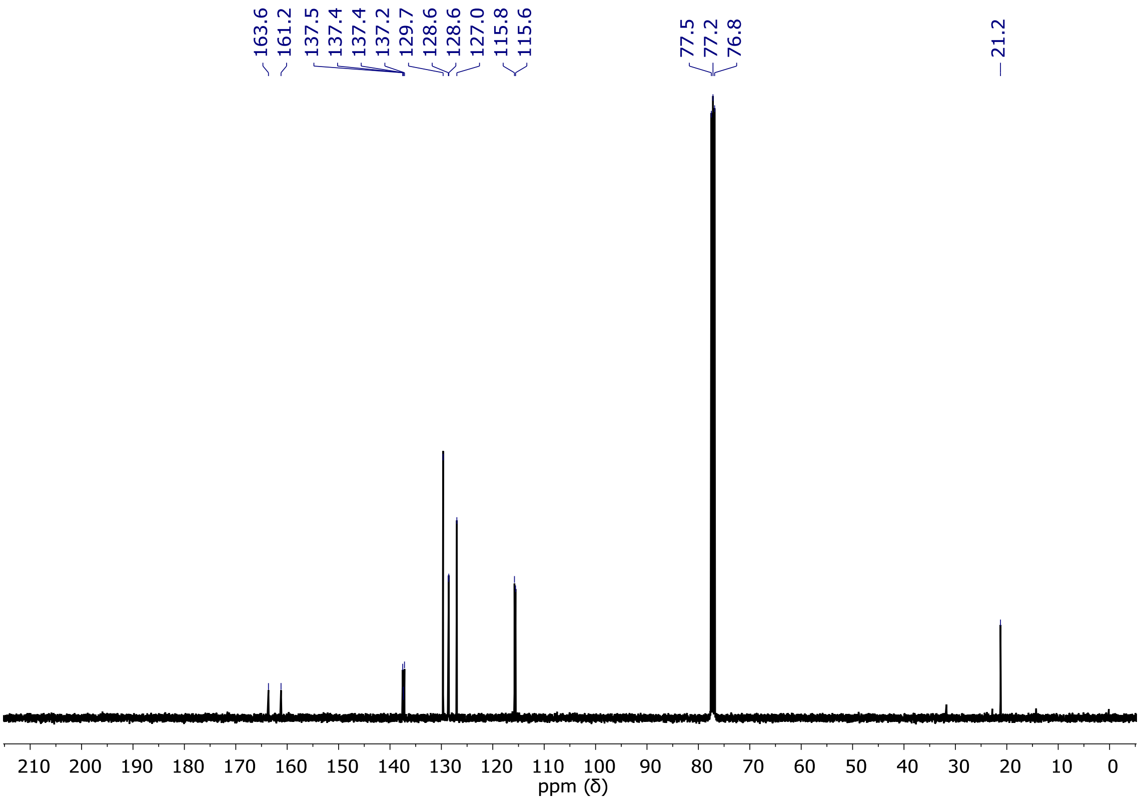

Figure S12. ^{19}F NMR Spectrum (376 MHz, $CDCl_3$) for 4-(4-fluorophenyl)toluene (**5**)

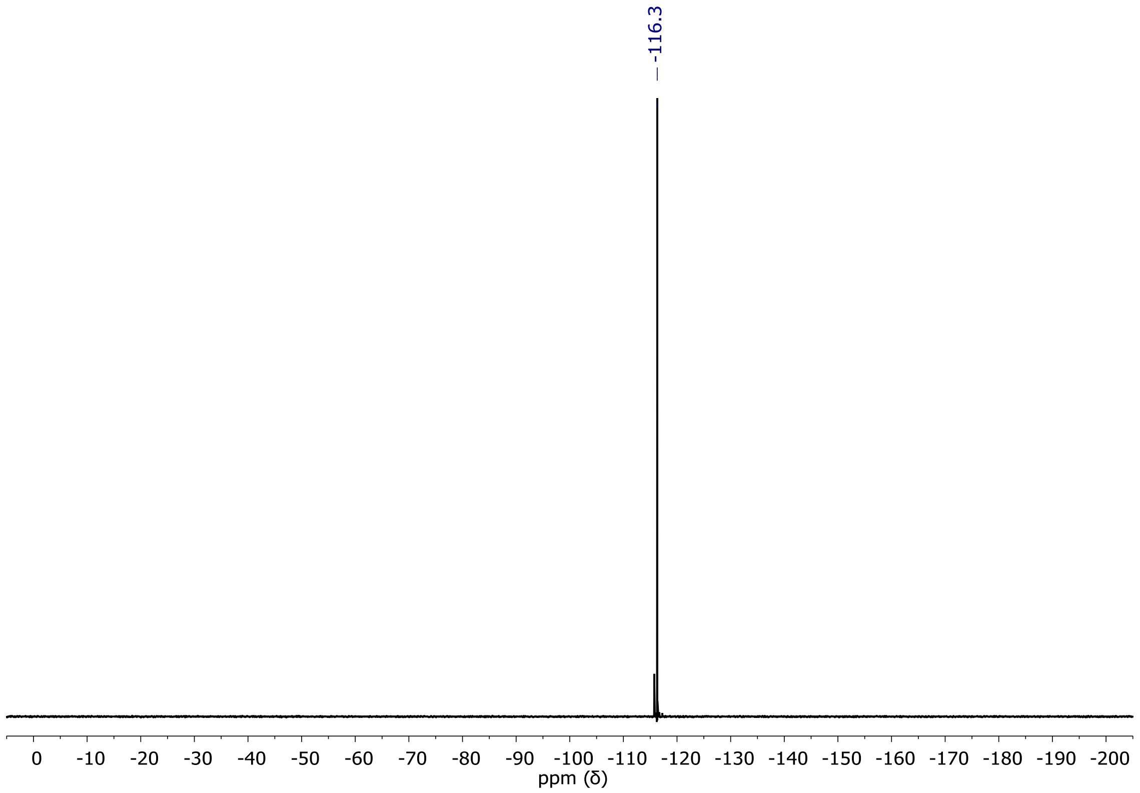

Figure S13. ^{1}H NMR Spectrum (400 MHz, $CDCl_3$) for 4-(4-fluorophenyl)aniline (**6**)

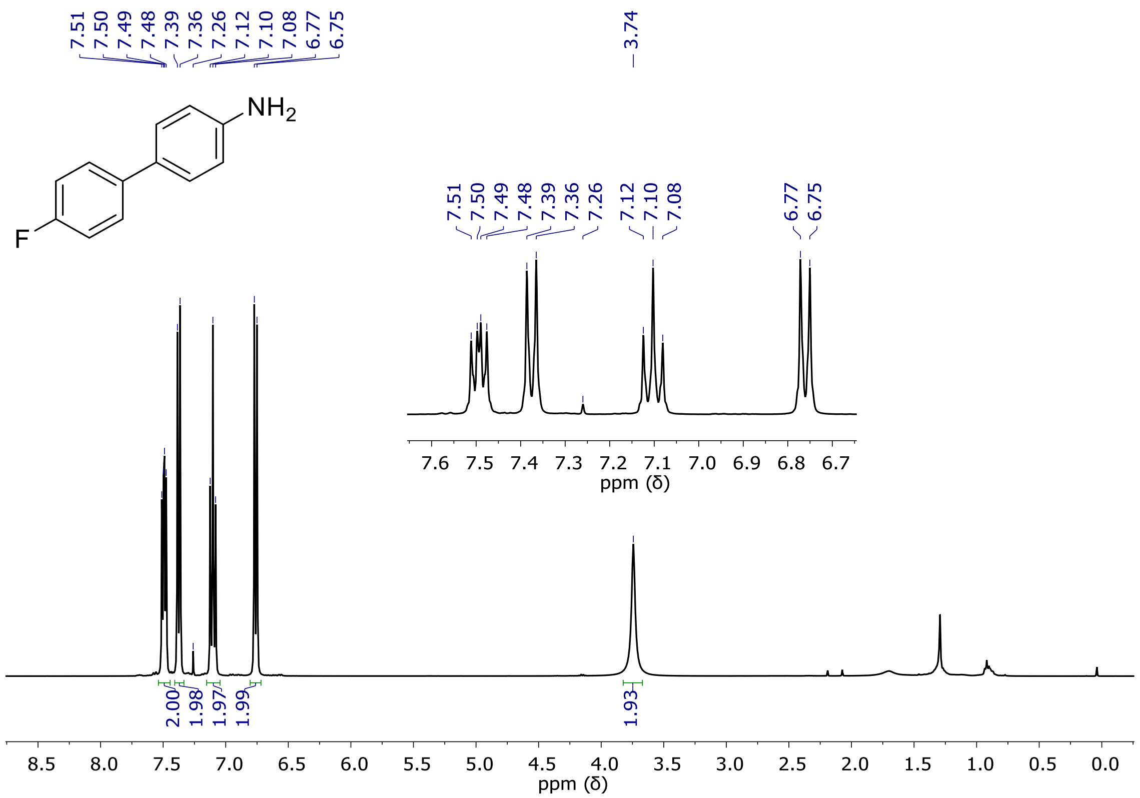

Figure S14. ^{13}C NMR Spectrum (100 MHz, $CDCl_3$) for 4-(4-fluorophenyl)aniline (**6**)

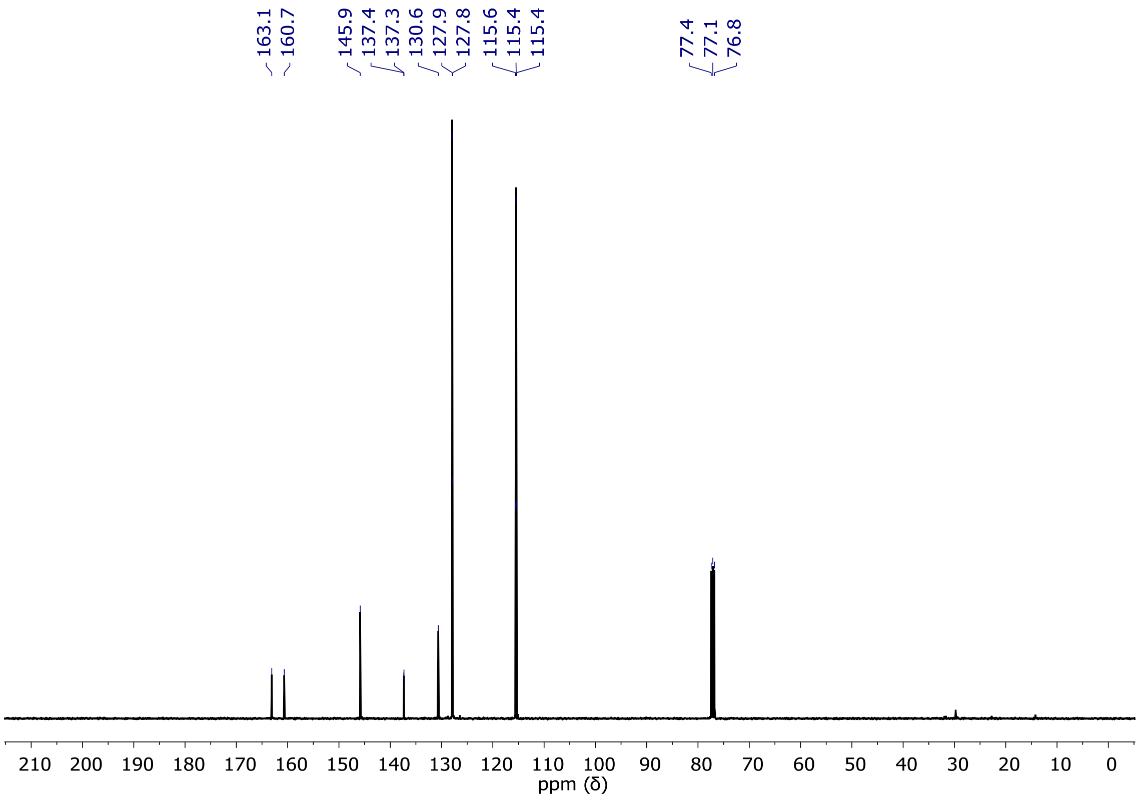

Figure S15. ^{19}F NMR Spectrum (376 MHz, $CDCl_3$) for 4-(4-fluorophenyl)aniline (**6**)

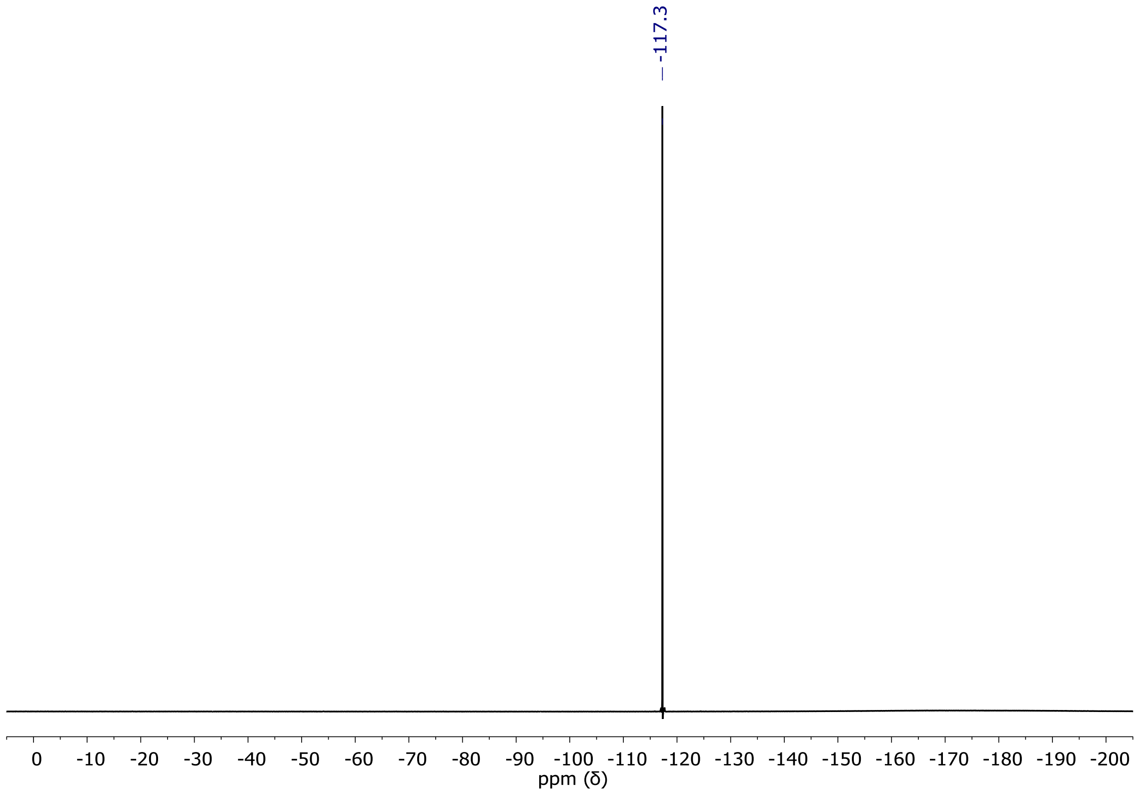

Figure S16. ^{1}H NMR Spectrum (400 MHz, $CDCl_3$) for 3-(4-fluorophenyl)aniline (**7**)

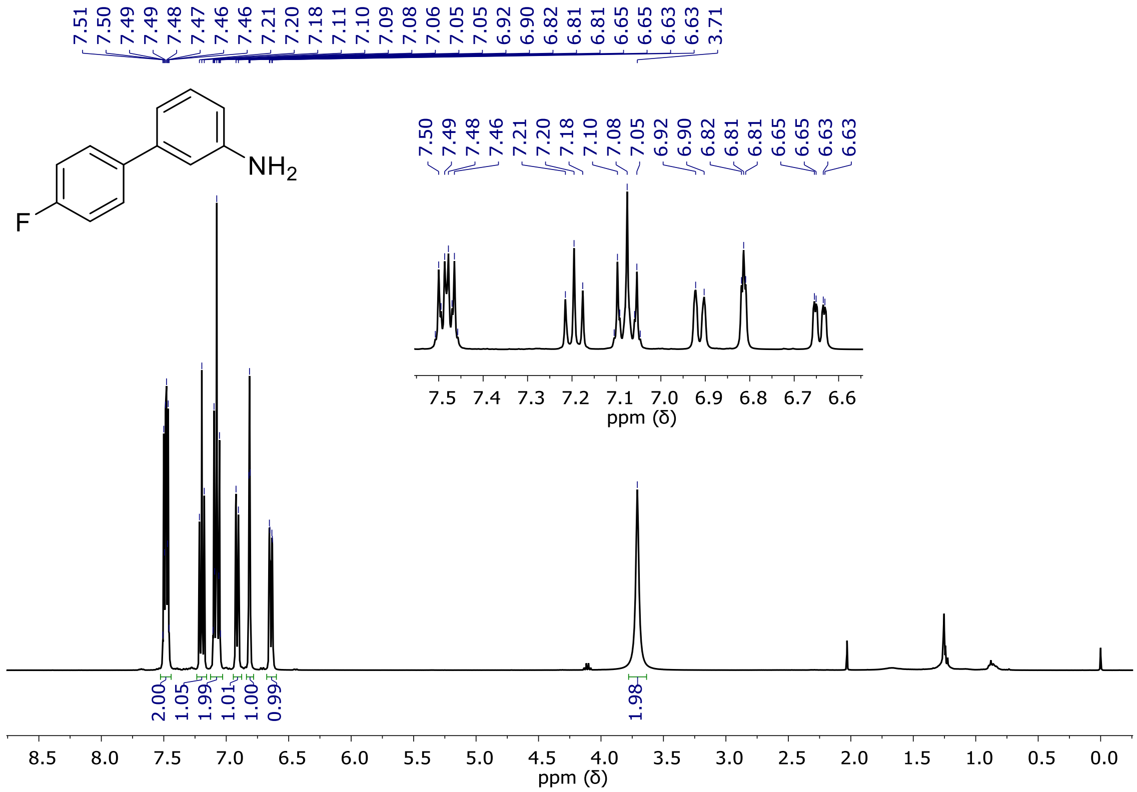

Figure S17. ^{13}C NMR Spectrum (100 MHz, $CDCl_3$) for 3-(4-fluorophenyl)aniline (**7**)

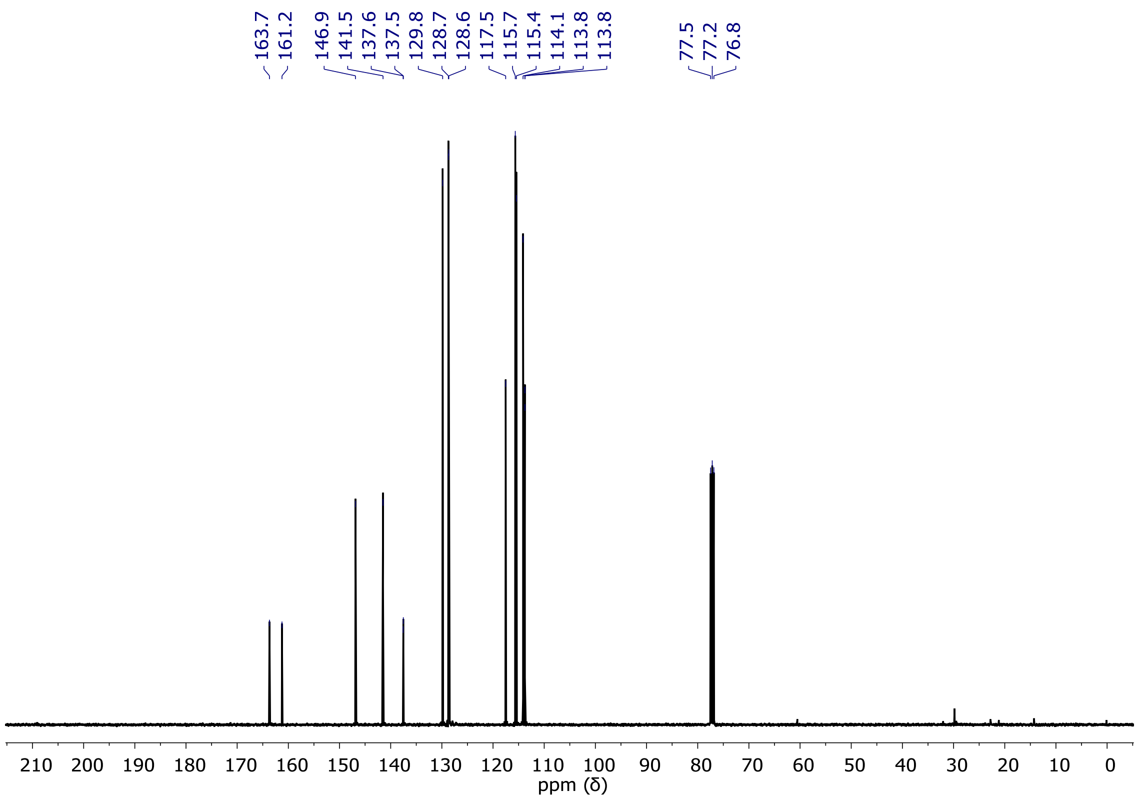

Figure S18. ^{19}F NMR Spectrum (376 MHz, $CDCl_3$) for 3-(4-fluorophenyl)aniline (**7**)

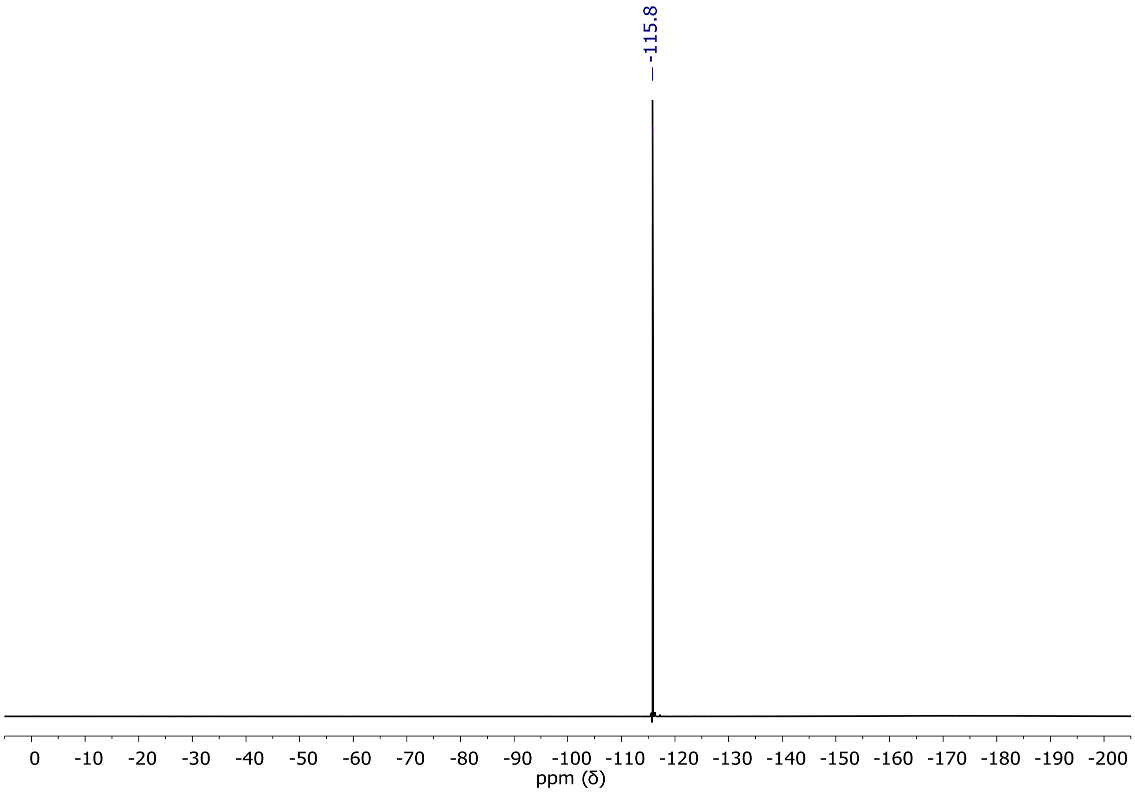

Figure S19. ^{1}H NMR Spectrum (400 MHz, $CDCl_3$) for 2-(4-fluorophenyl)aniline (**8**)

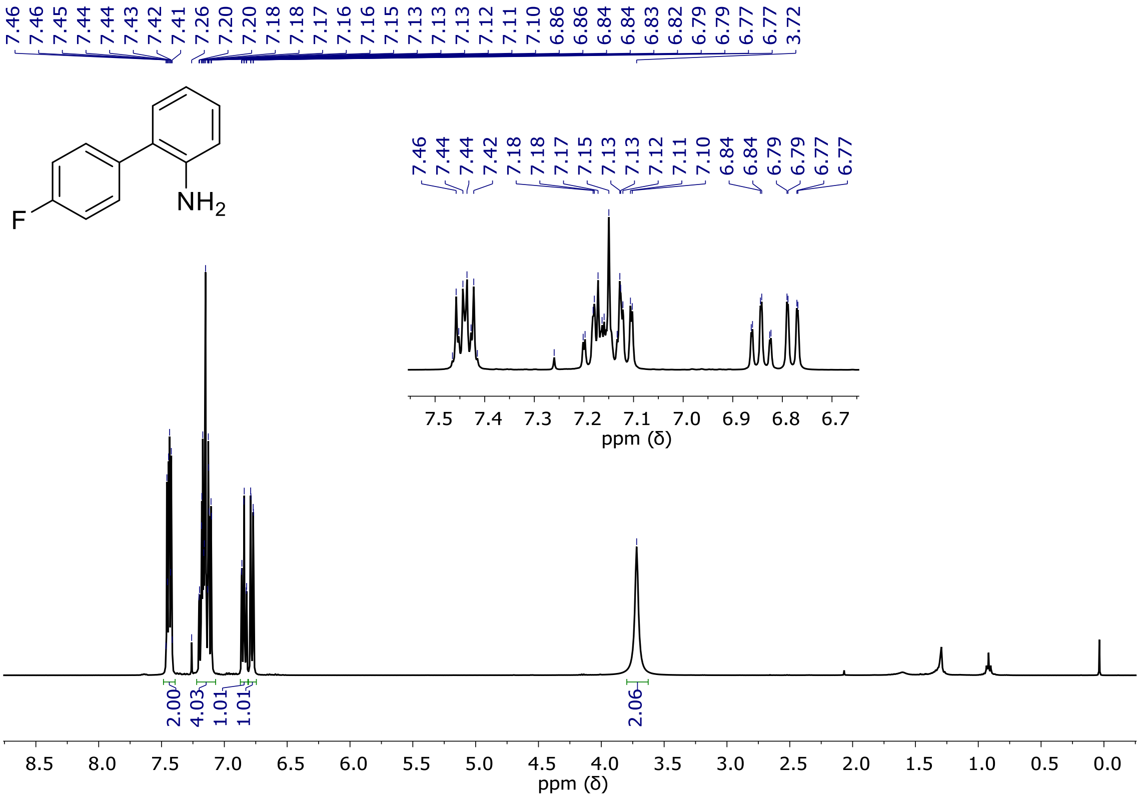

Figure S20. ^{13}C NMR Spectrum (100 MHz, $CDCl_3$) for 2-(4-fluorophenyl)aniline (**8**)

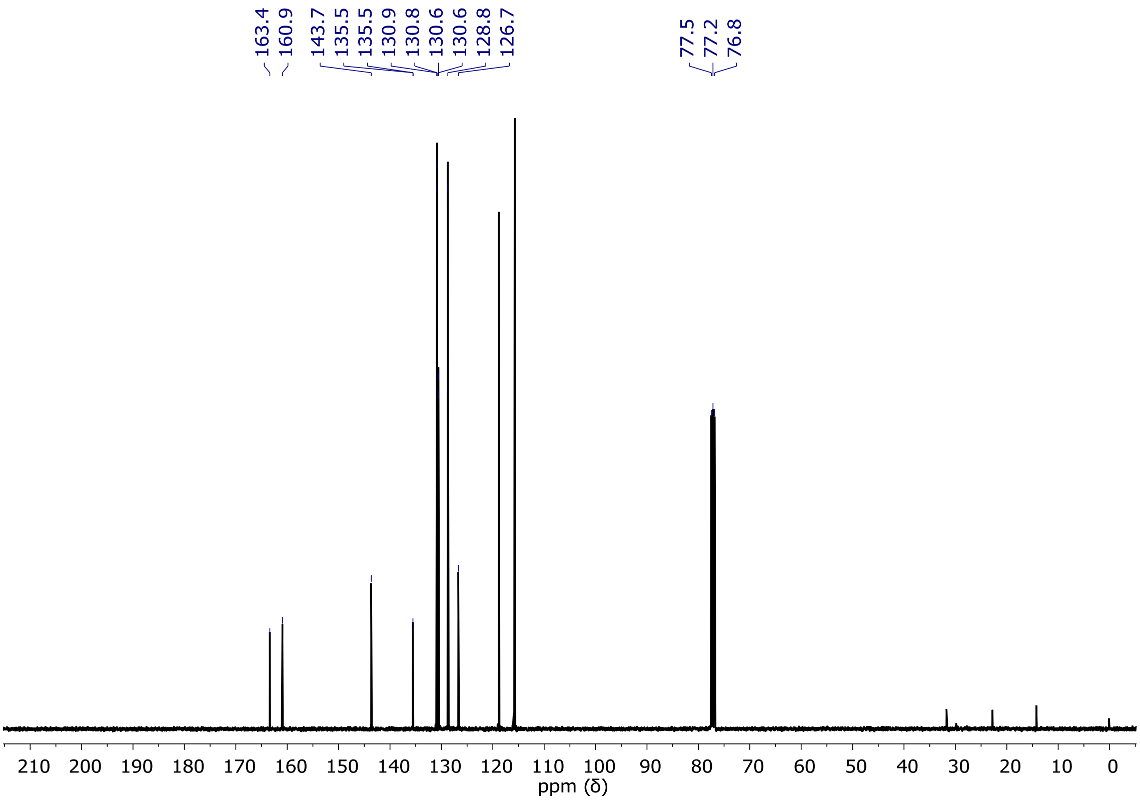

Figure S21. ^{19}F NMR Spectrum (376 MHz, $CDCl_3$) for 2-(4-fluorophenyl)aniline (**8**)

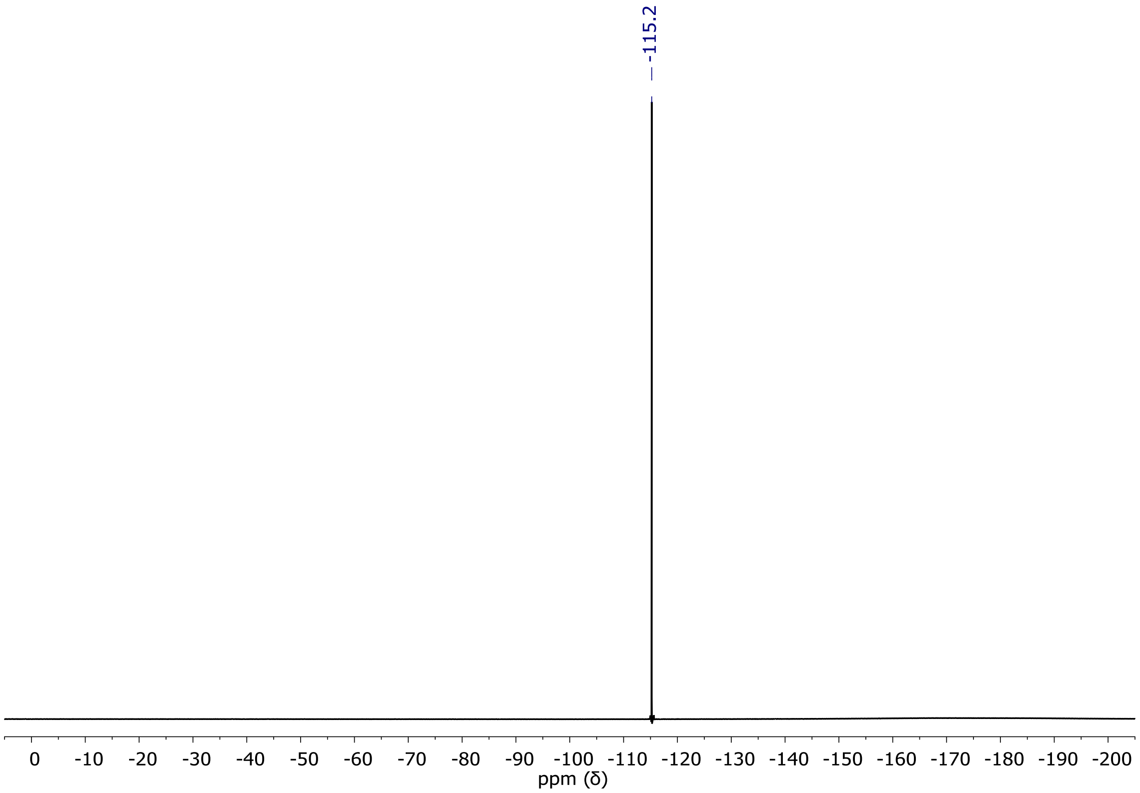

Figure S22. ^{1}H NMR Spectrum (400 MHz, $CDCl_3$) for 4-(4-fluorophenyl)anisole (**9**)

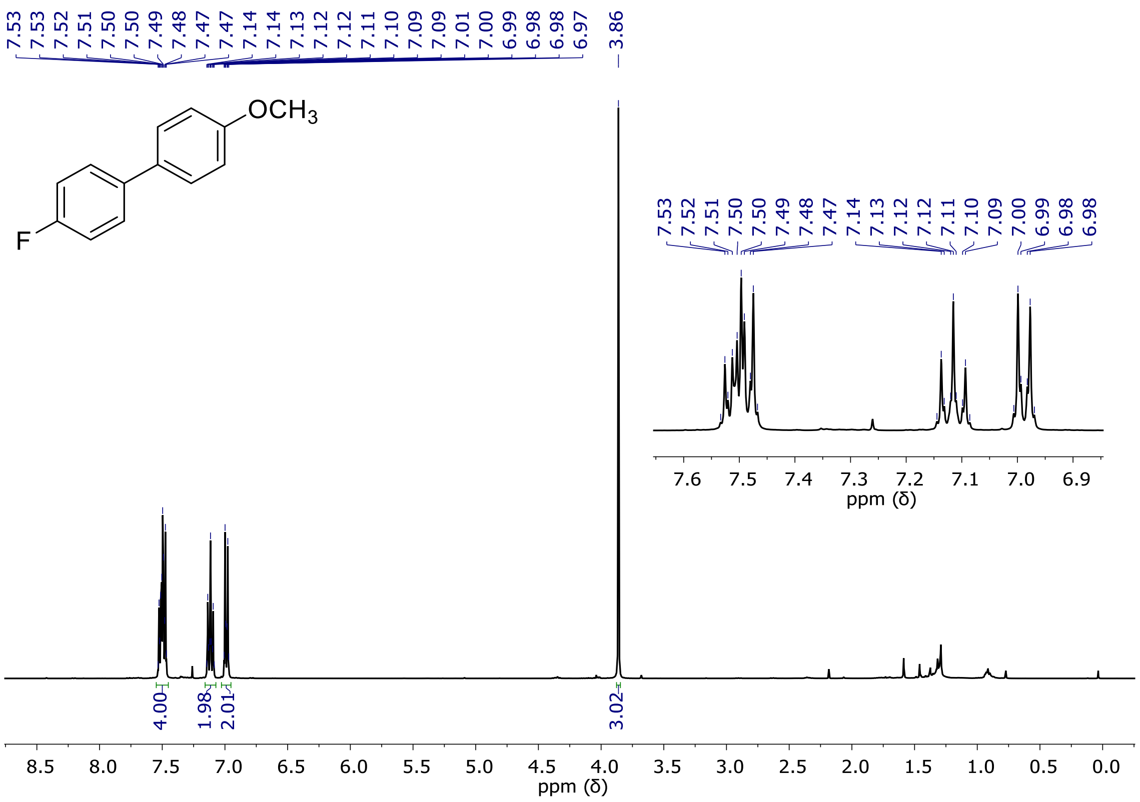

Figure S23. ^{13}C NMR Spectrum (100 MHz, $CDCl_3$) for 4-(4-fluorophenyl)anisole (**9**)

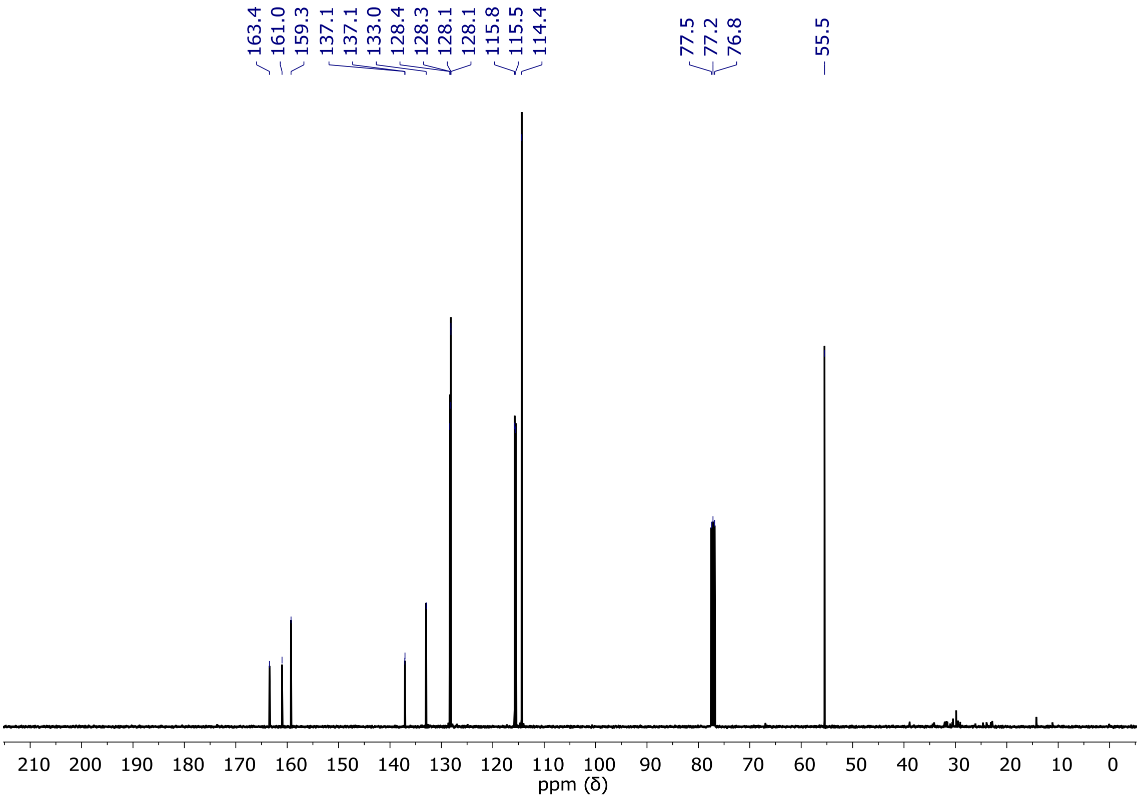

Figure S24. ^{19}F NMR Spectrum (376 MHz, $CDCl_3$) for 4-(4-fluorophenyl)anisole (**9**)

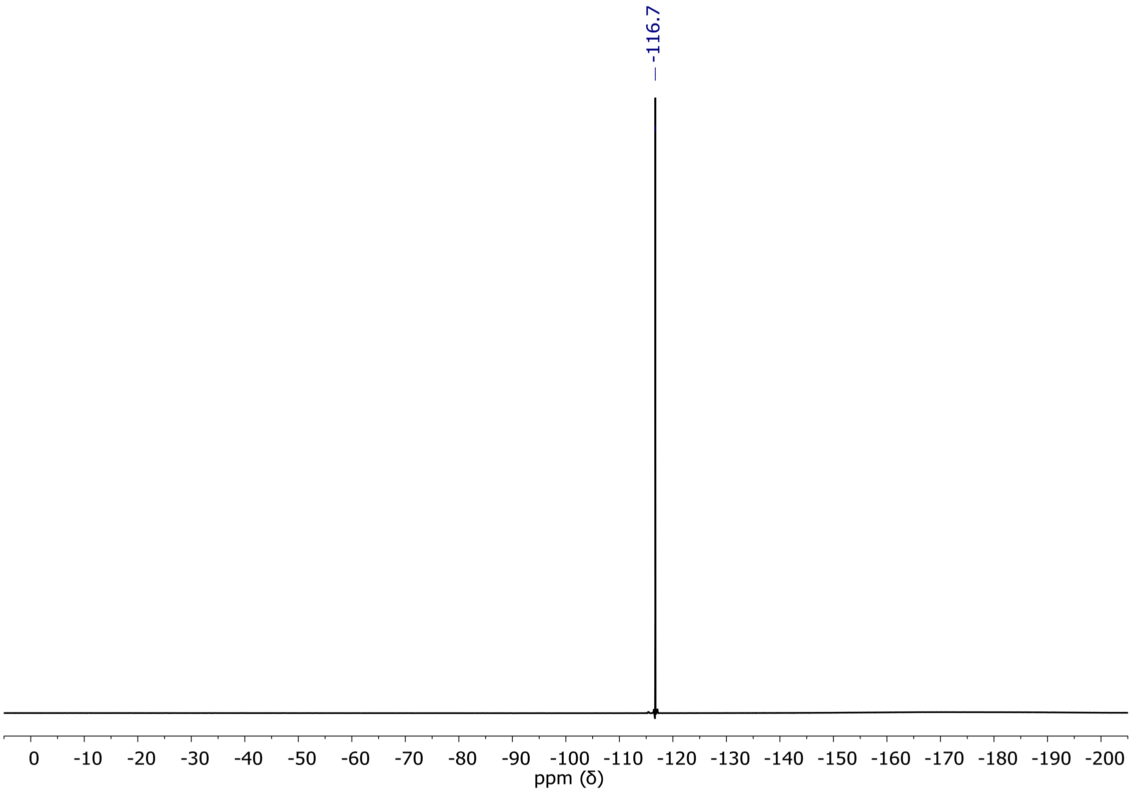

Figure S25. ^{1}H NMR Spectrum (400 MHz, $CDCl_3$) for 3-(4-fluorophenyl)anisole (**10**)

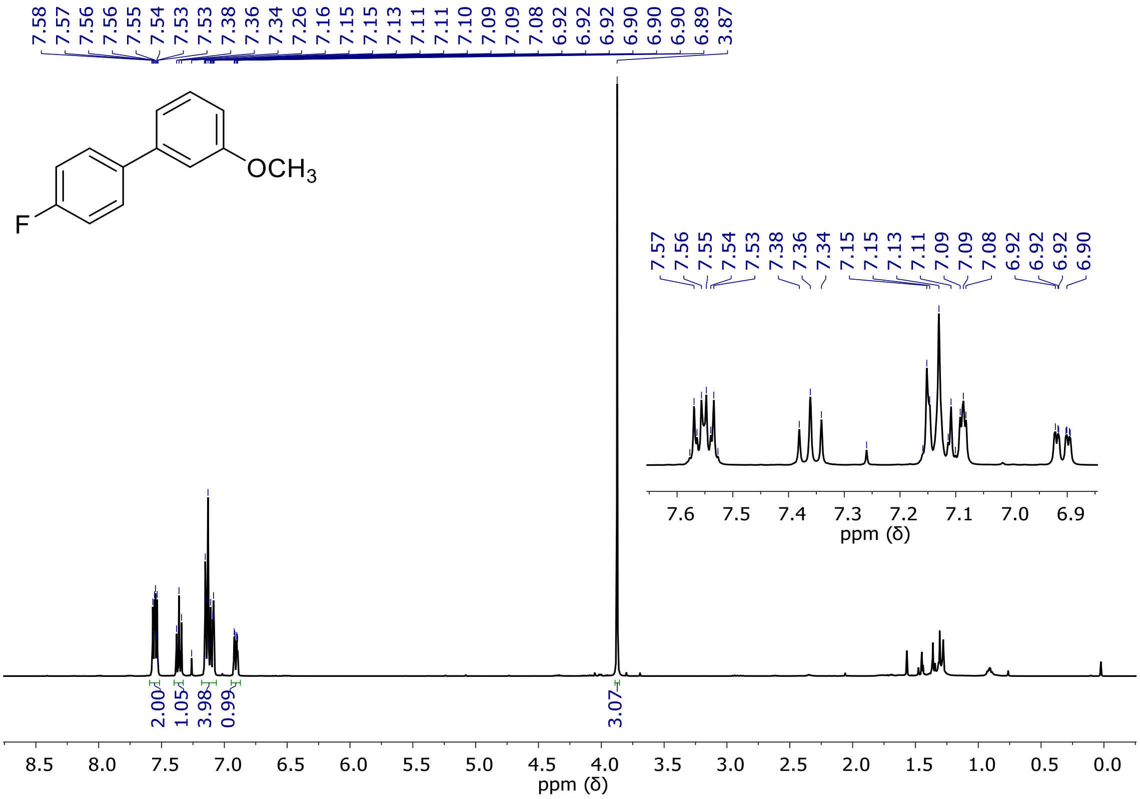

Figure S26. ^{13}C NMR Spectrum (100 MHz, $CDCl_3$) for 3-(4-fluorophenyl)anisole (**10**)

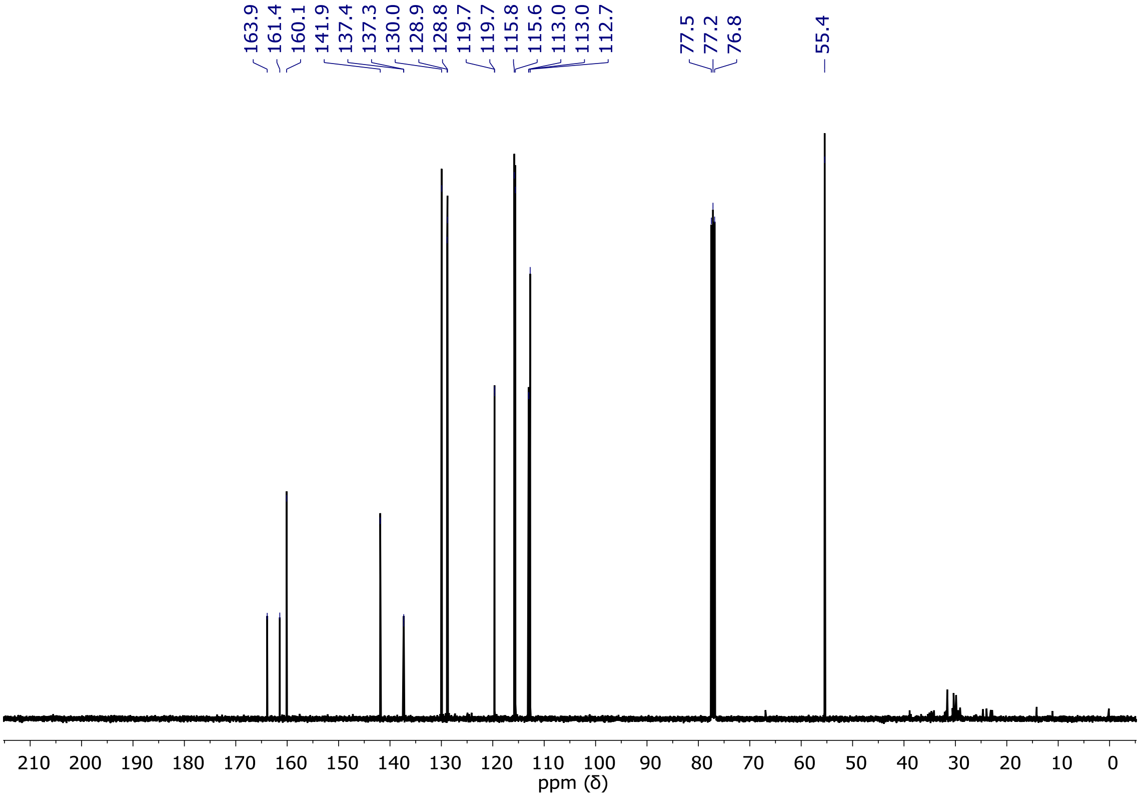

Figure S27. ^{19}F NMR Spectrum (376 MHz, $CDCl_3$) for 3-(4-fluorophenyl)anisole (**10**)

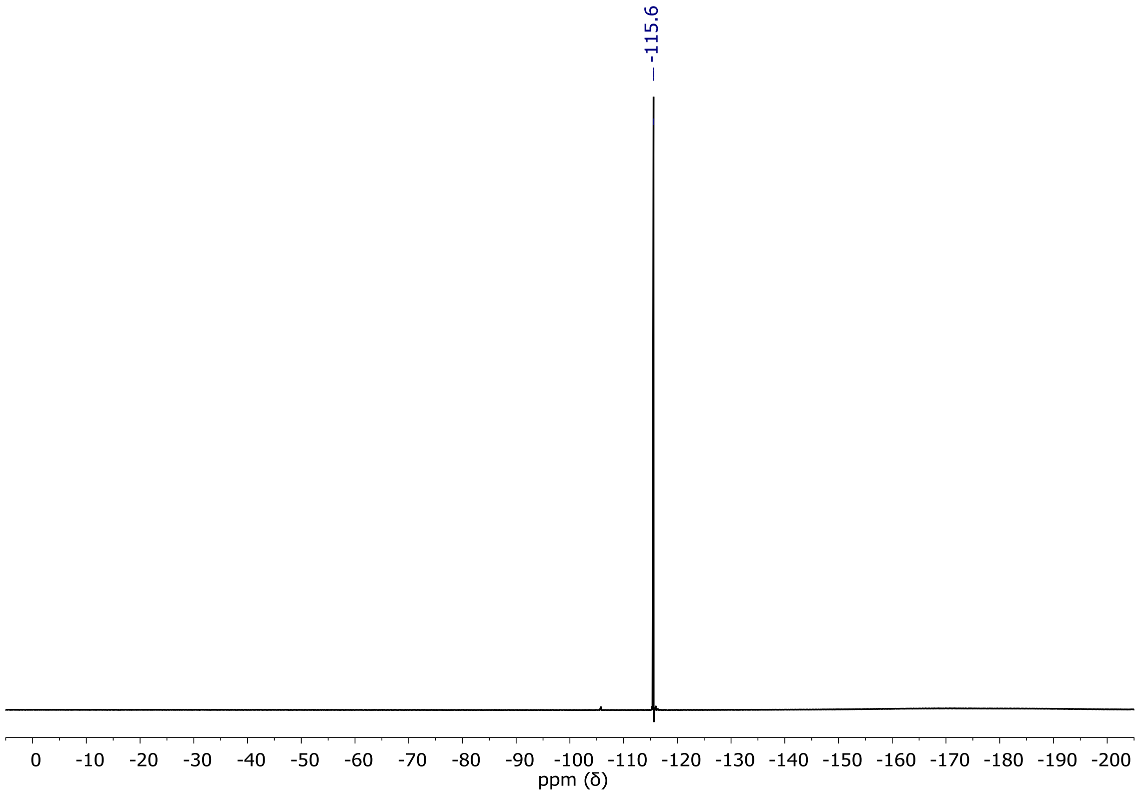

Figure S28. ^{1}H NMR Spectrum (400 MHz, $CDCl_3$) for 2-(4-fluorophenyl)anisole (**11**)

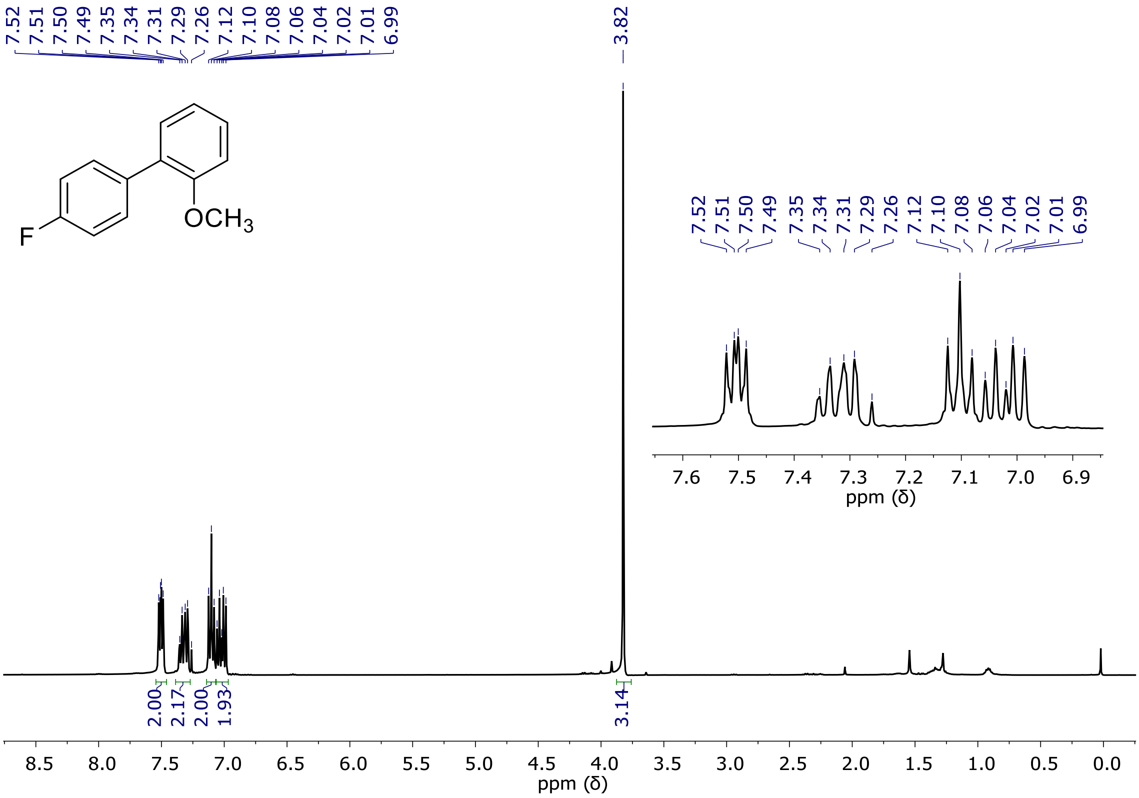

Figure S29. ^{13}C NMR Spectrum (100 MHz, $CDCl_3$) for 2-(4-fluorophenyl)anisole (**11**)

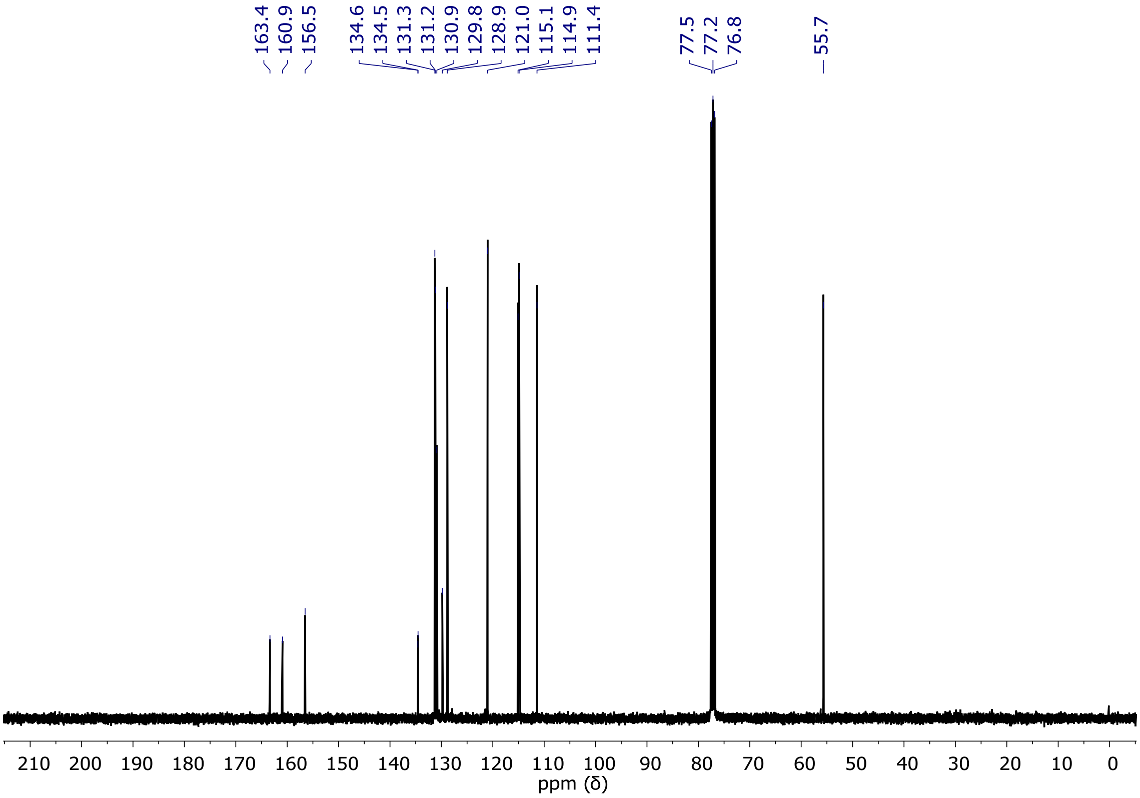

Figure S30. ^{19}F NMR Spectrum (376 MHz, $CDCl_3$) for 2-(4-fluorophenyl)anisole (**11**)

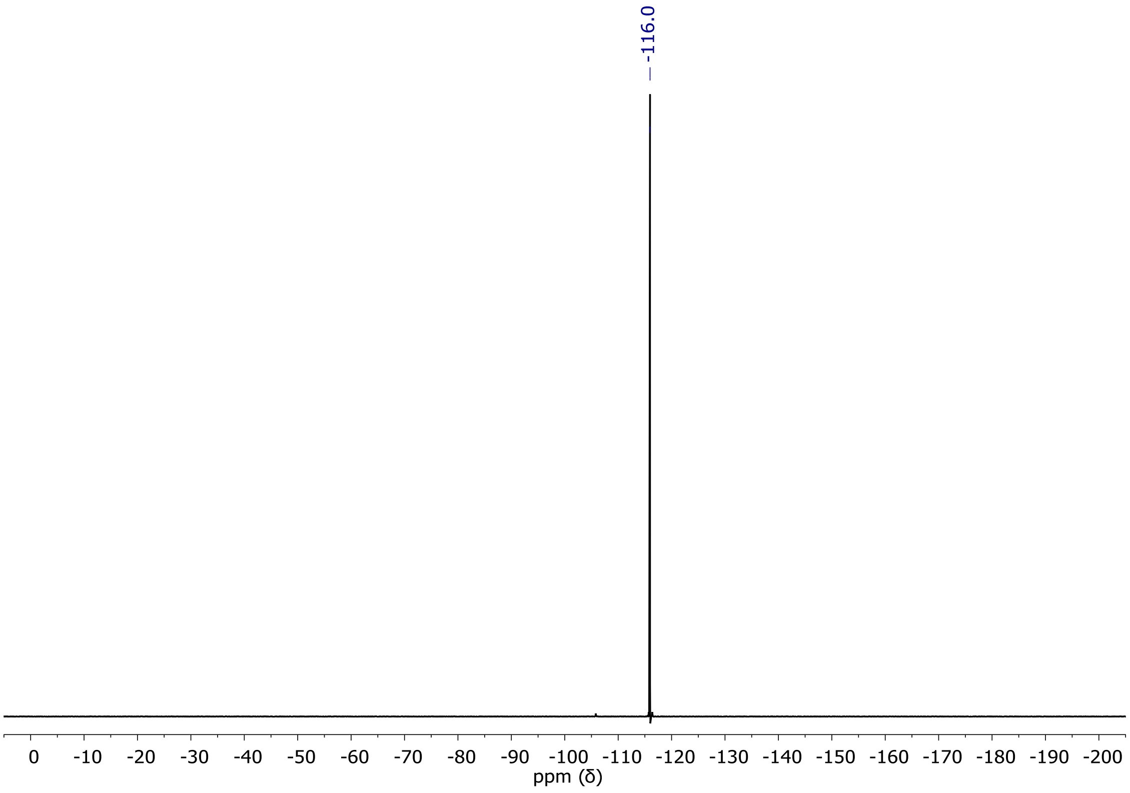

Figure S31. ^{1}H NMR Spectrum (400 MHz, $CDCl_3$) for 4-(4-fluorophenyl)acetophenone (**12**)

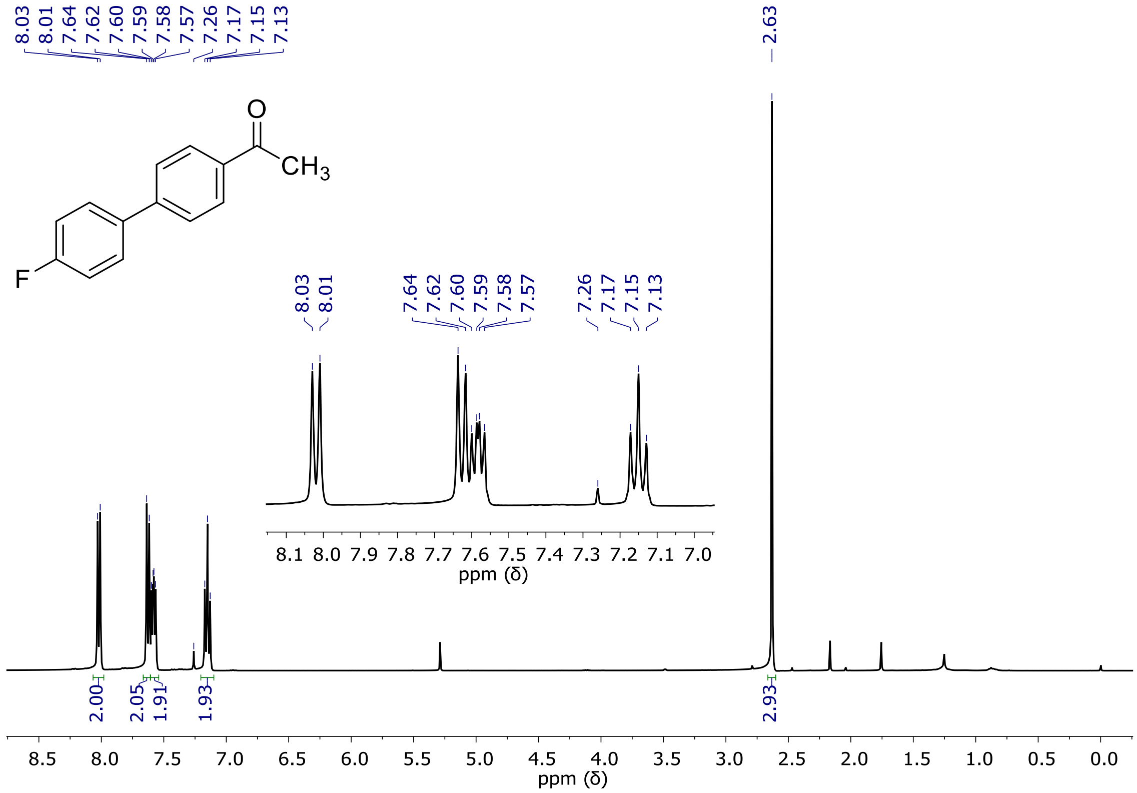

Figure S32. ^{13}C NMR Spectrum (100 MHz, $CDCl_3$) for 4-(4-fluorophenyl)acetophenone (**12**)

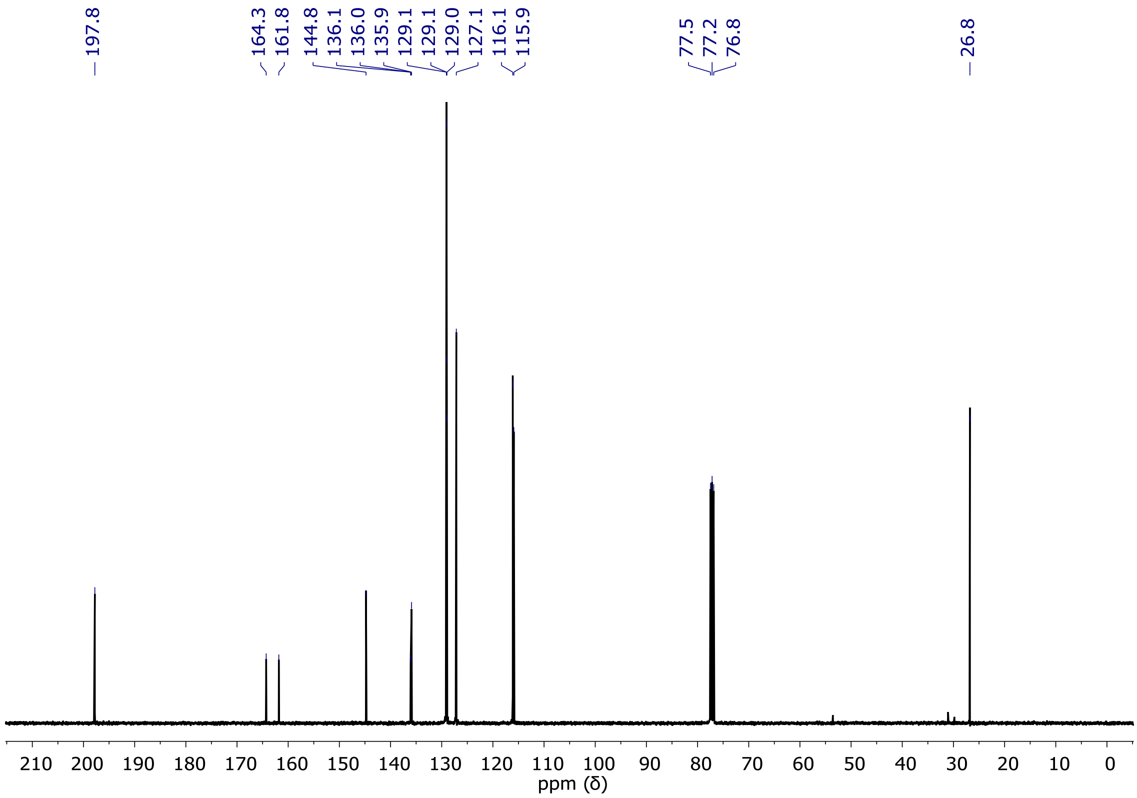

Figure S33. ^{19}F NMR Spectrum (376 MHz, $CDCl_3$) for 4-(4-fluorophenyl)acetophenone (**12**)

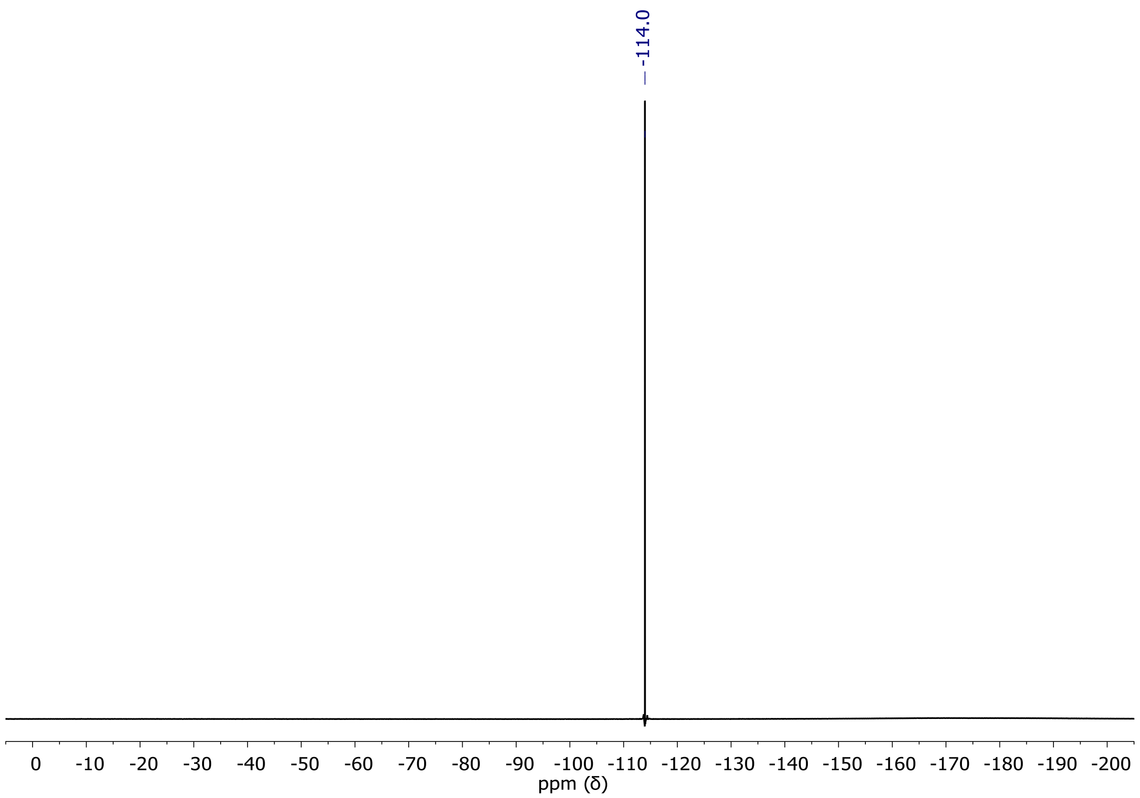

Figure S34. ^{1}H NMR Spectrum (400 MHz, $CDCl_3$) for 3-(4-fluorophenyl)acetophenone (**13**)

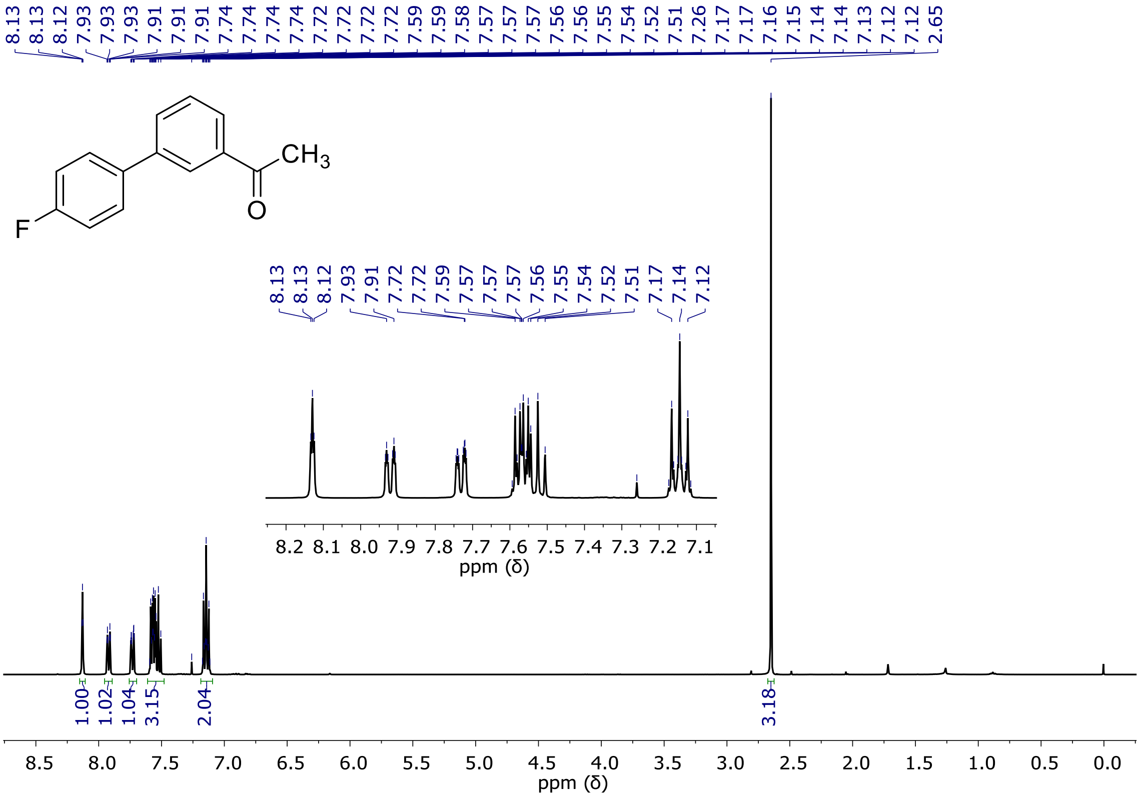

Figure S35. ^{13}C NMR Spectrum (100 MHz, $CDCl_3$) for 3-(4-fluorophenyl)acetophenone (**13**)

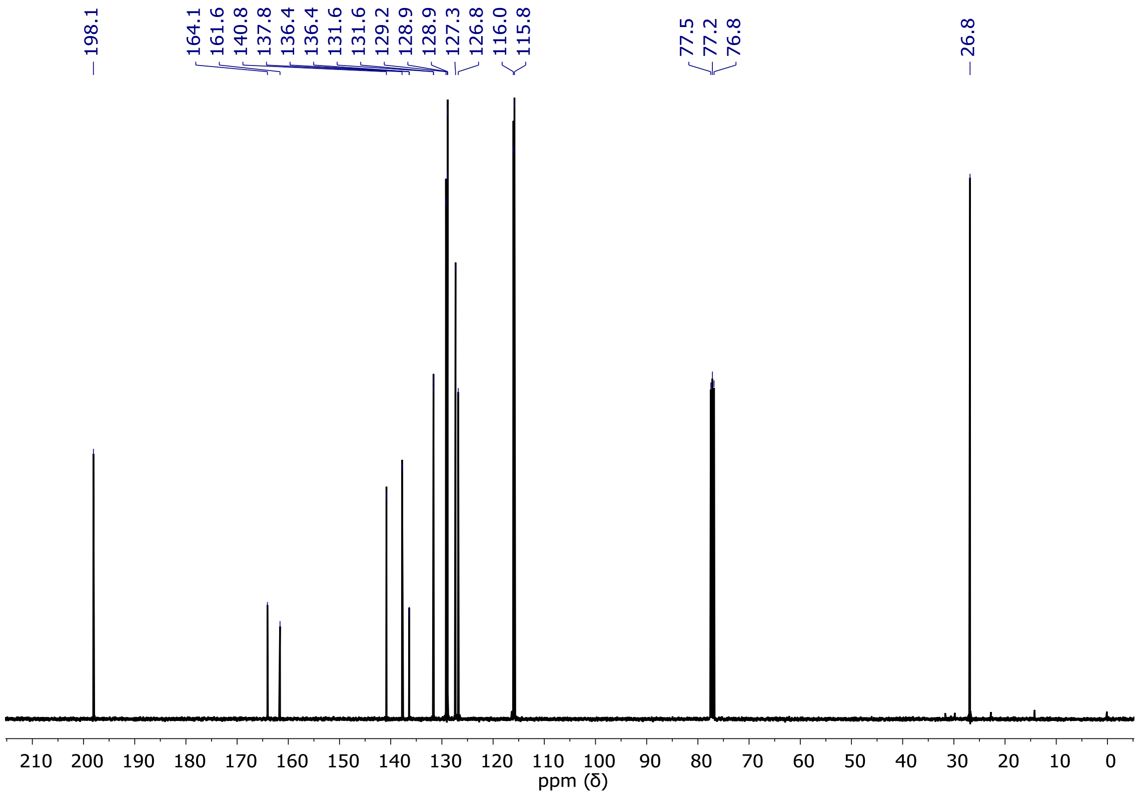

Figure S36. ^{19}F NMR Spectrum (376 MHz, $CDCl_3$) for 3-(4-fluorophenyl)acetophenone (**13**)

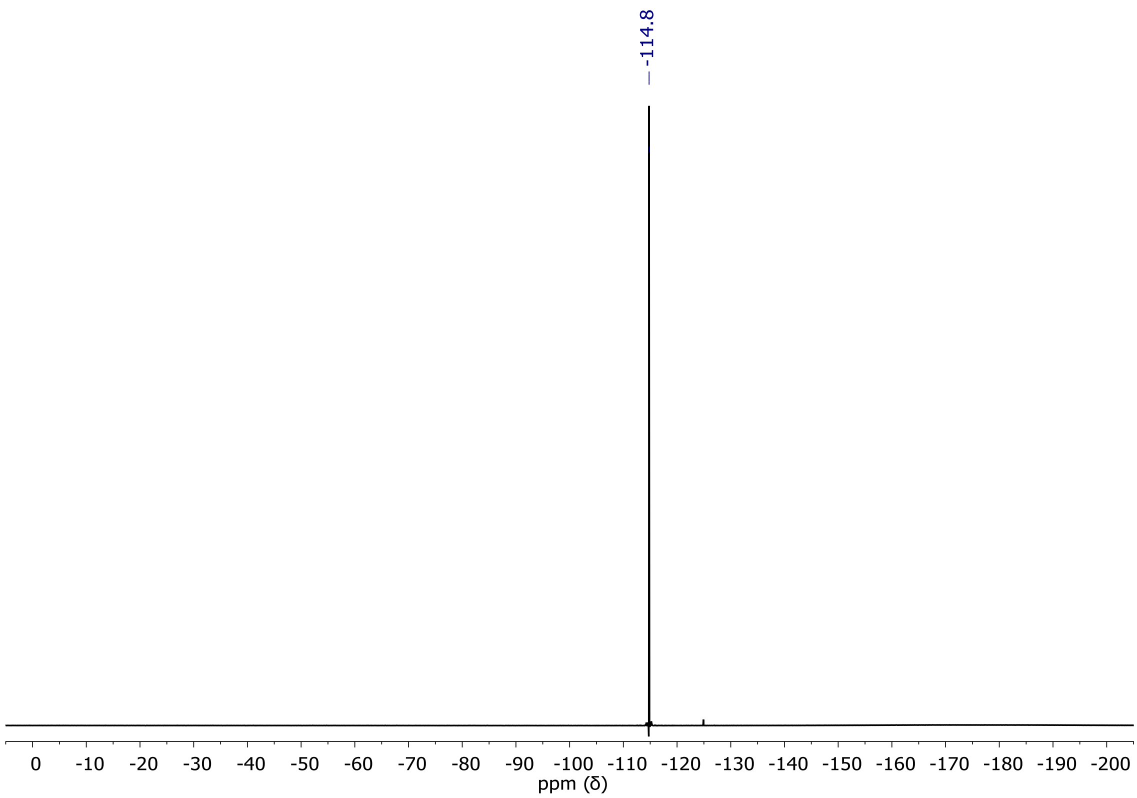

Figure S37. ^{1}H NMR Spectrum (400 MHz, $CDCl_3$) for 2-(4-fluorophenyl)acetophenone (**14**)

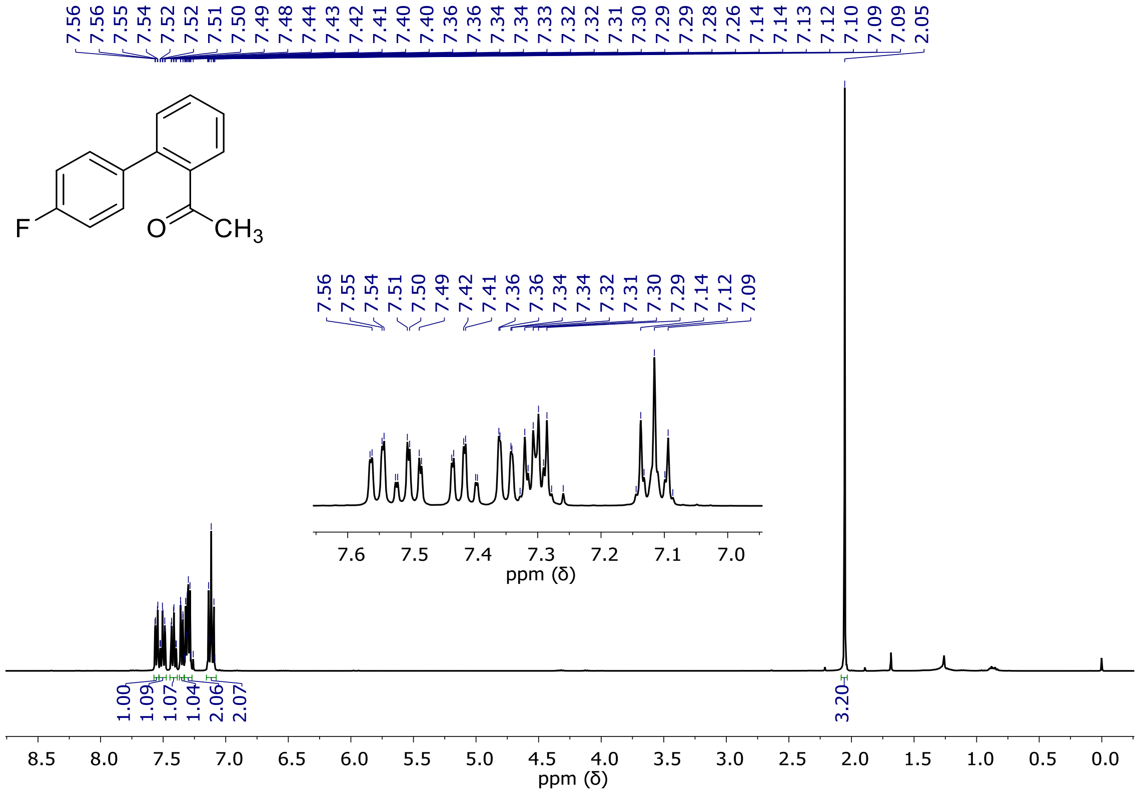

Figure S38. ^{13}C NMR Spectrum (100 MHz, $CDCl_3$) for 2-(4-fluorophenyl)acetophenone (**14**)

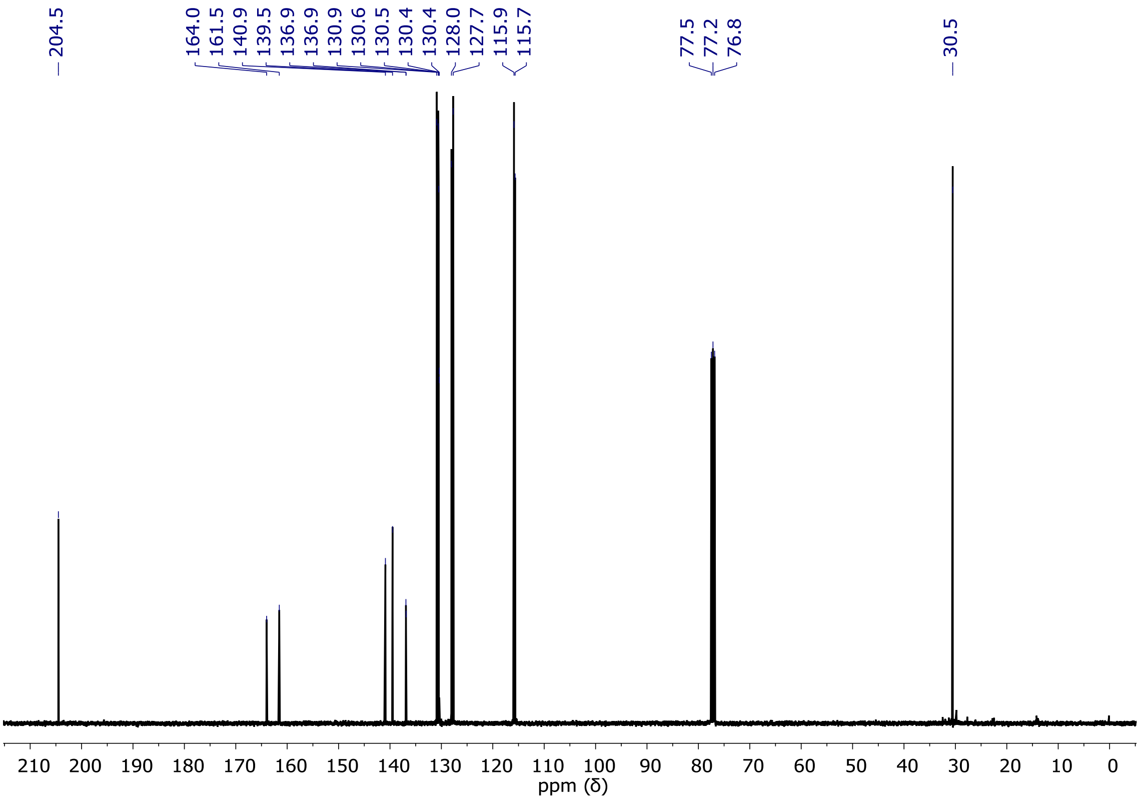

Figure S39. ^{19}F NMR Spectrum (376 MHz, $CDCl_3$) for 2-(4-fluorophenyl)acetophenone (**14**)

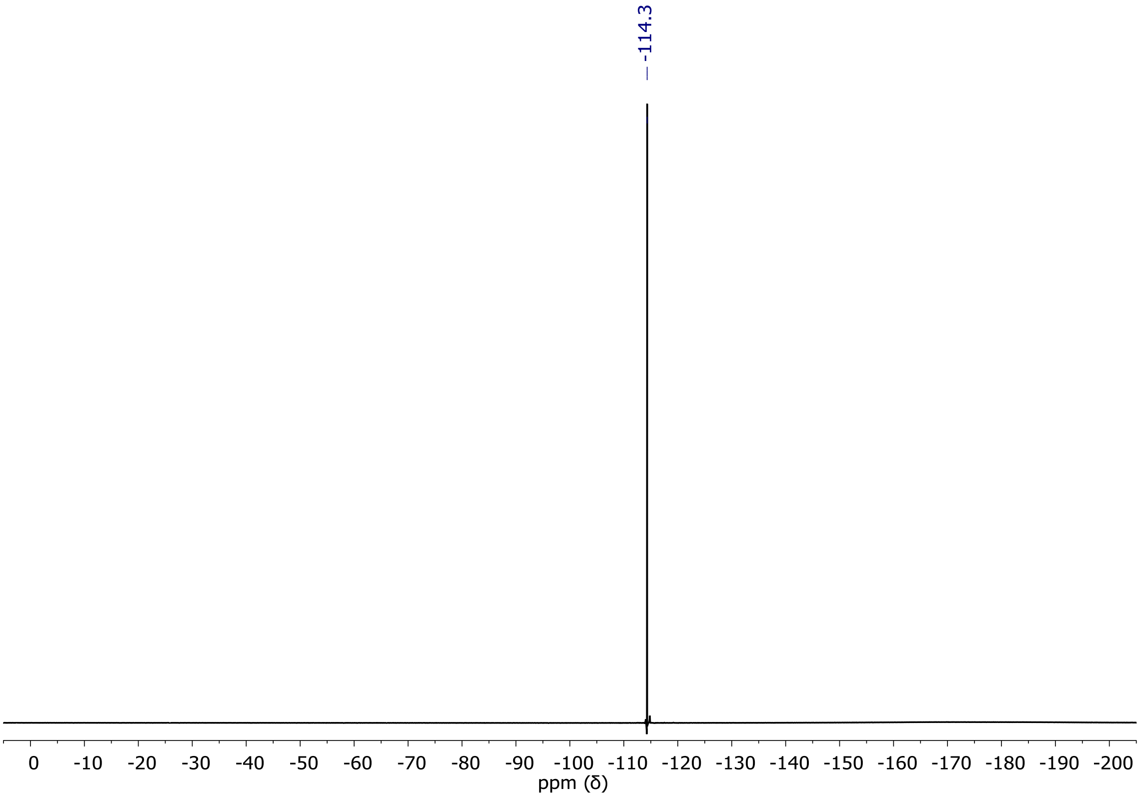

Figure S40. ^{1}H NMR Spectrum (400 MHz, $CDCl_3$) for 4-(4-fluorophenyl)benzaldehyde (**15**)

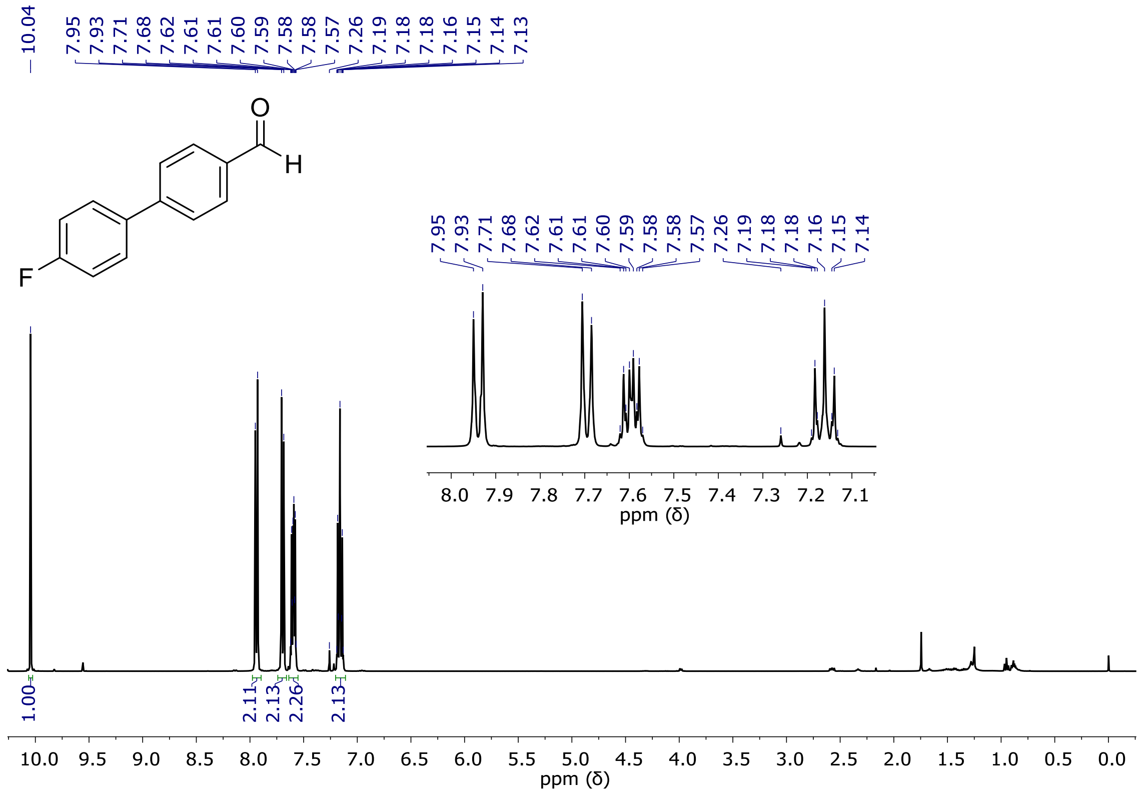

Figure S41. ^{13}C NMR Spectrum (100 MHz, $CDCl_3$) for 4-(4-fluorophenyl)benzaldehyde (**15**)

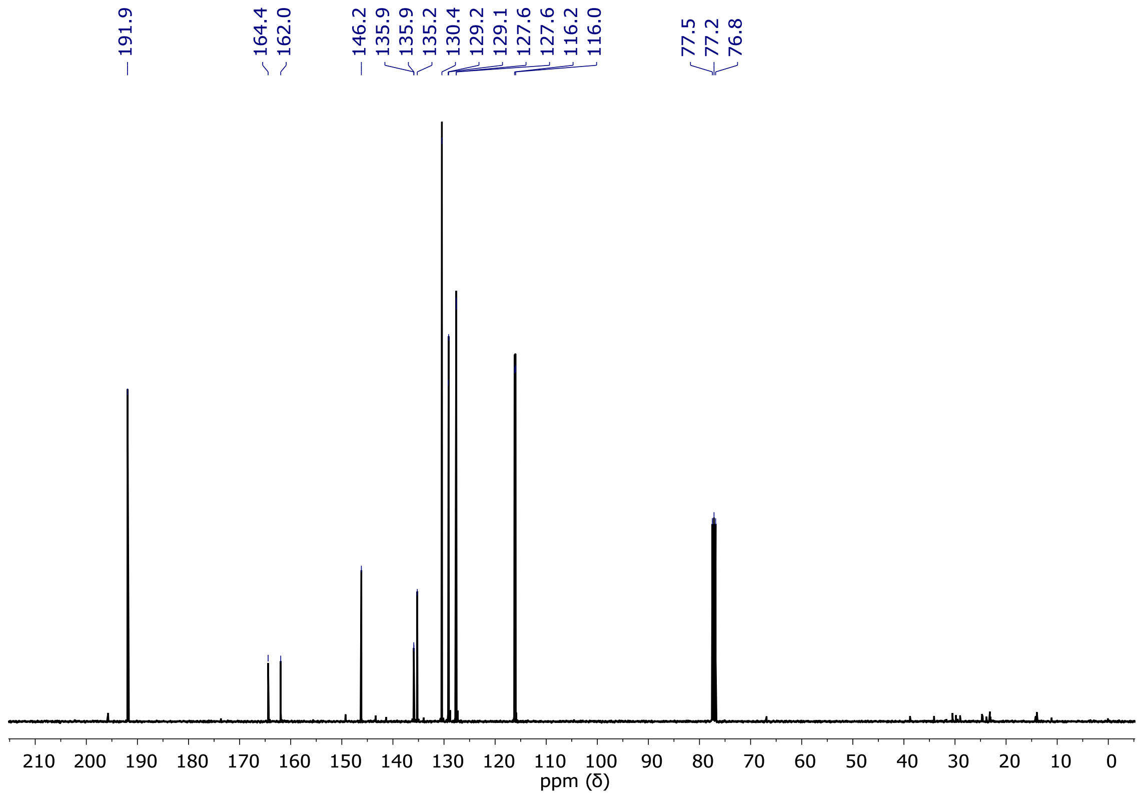

Figure S42. ^{19}F NMR Spectrum (376 MHz, $CDCl_3$) for 4-(4-fluorophenyl)benzaldehyde (**15**)

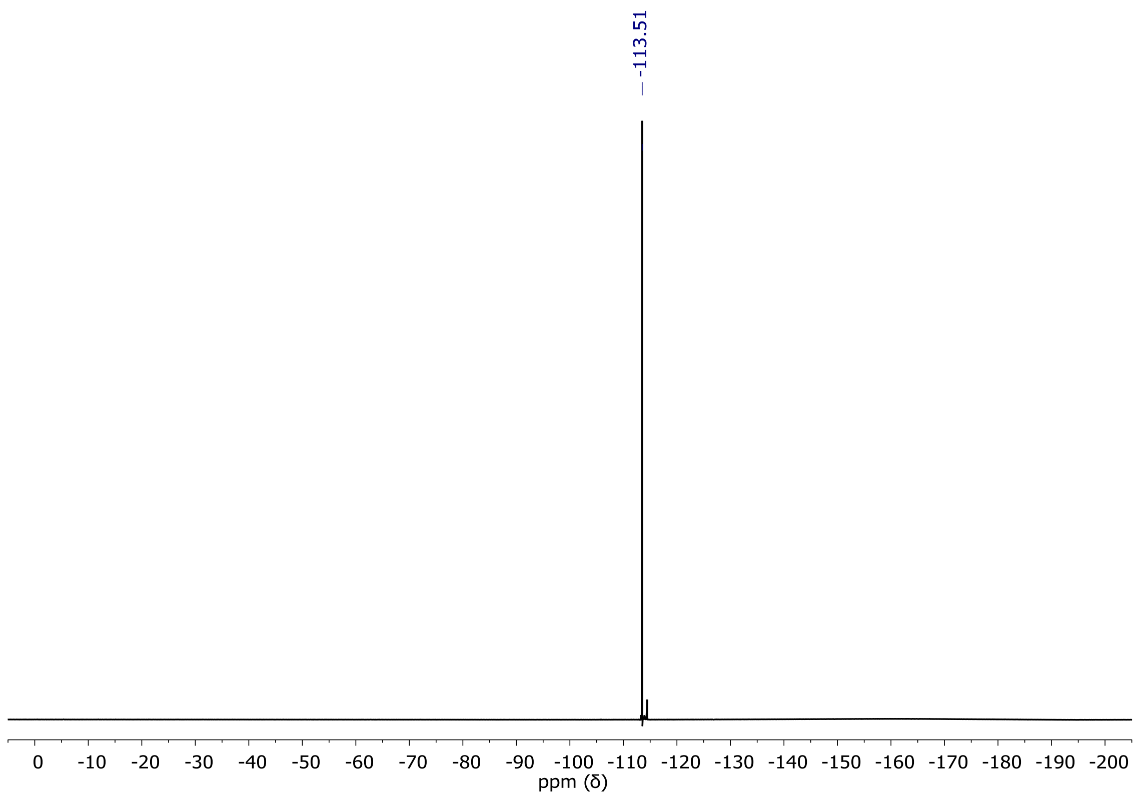

Figure S43. ^{1}H NMR Spectrum (400 MHz, $CDCl_3$) for ethyl 4-(4-fluorophenyl)benzoate (**16**)

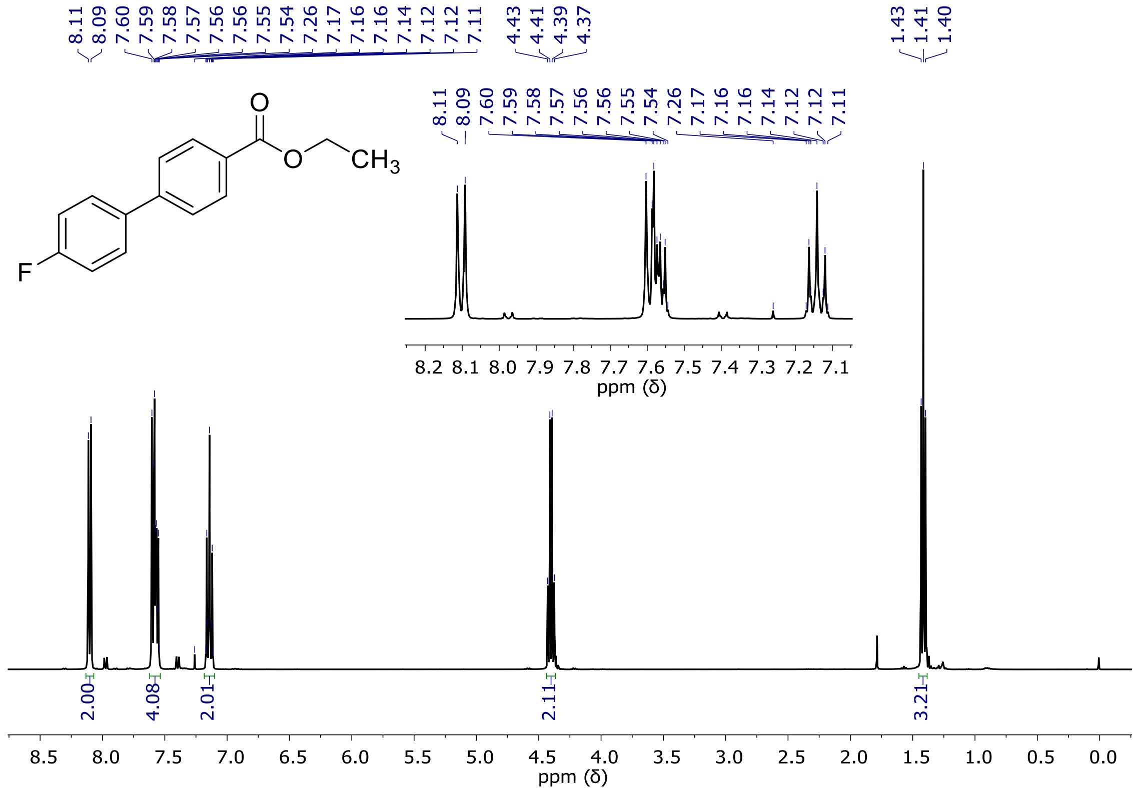

Figure S44. ^{13}C NMR Spectrum (100 MHz, $CDCl_3$) for ethyl 4-(4-fluorophenyl)benzoate (**16**)

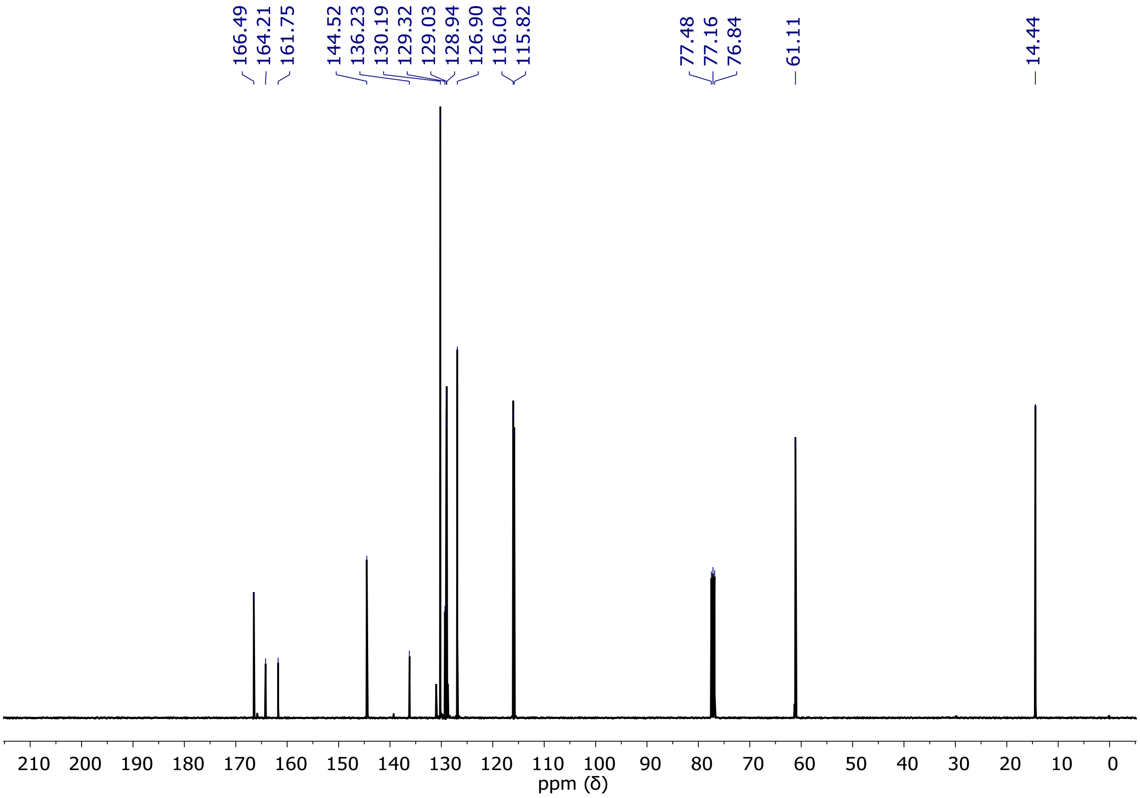

Figure S45. ^{19}F NMR Spectrum (376 MHz, $CDCl_3$) for ethyl 4-(4-fluorophenyl)benzoate (**16**)

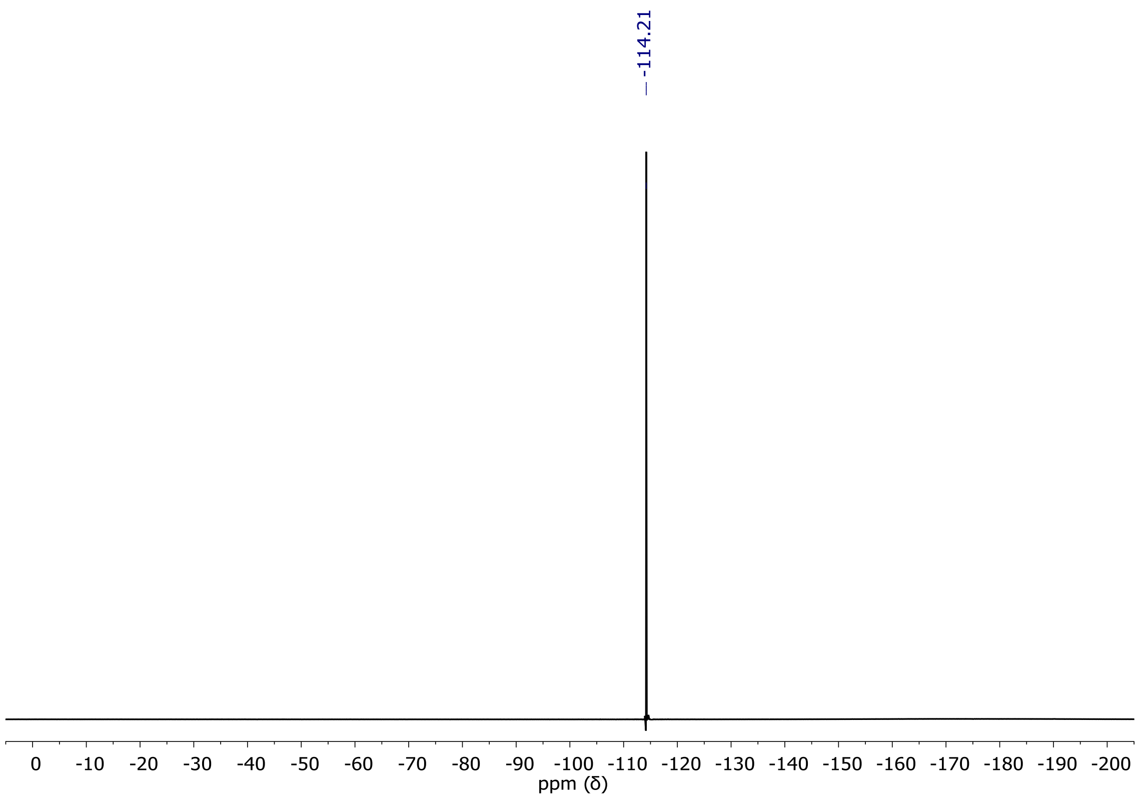

Figure S46. ^{1}H NMR Spectrum (400 MHz, $CDCl_3$) for 4-(4-fluorophenyl)benzene acetonitrile (**17**)

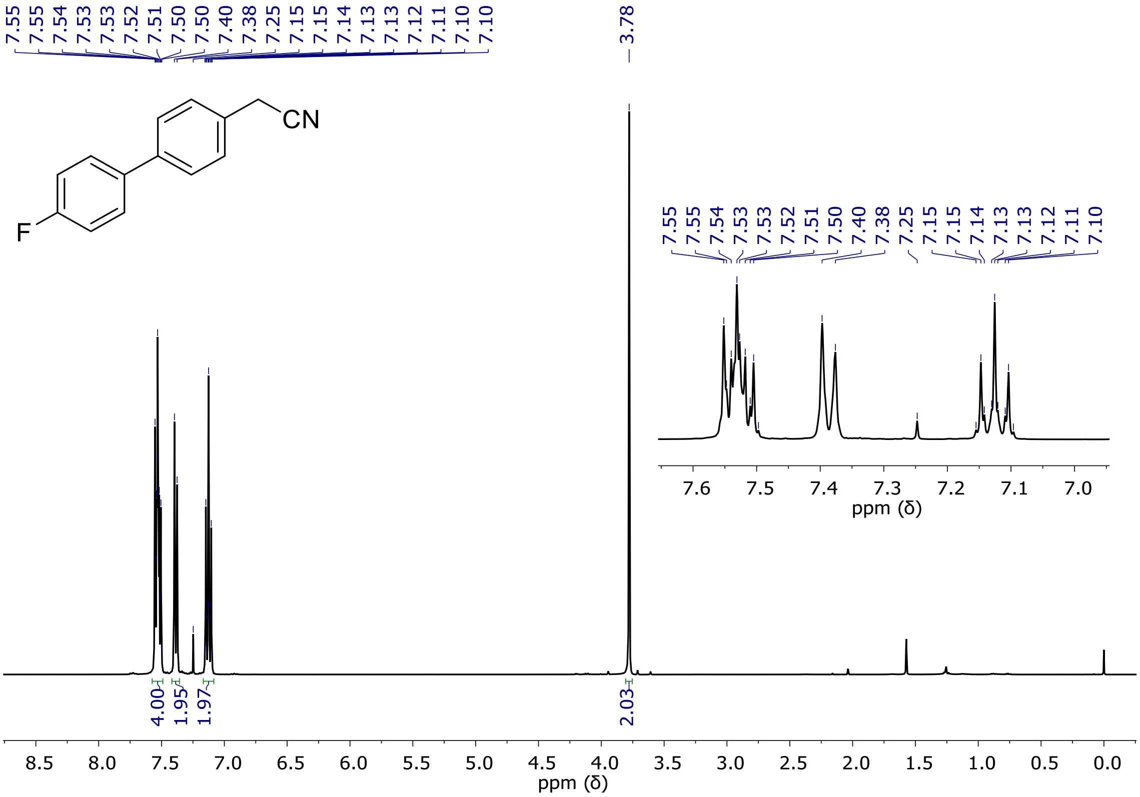

Figure S47. ^{13}C NMR Spectrum (100 MHz, $CDCl_3$) for 4-(4-fluorophenyl)benzene acetonitrile (**17**)

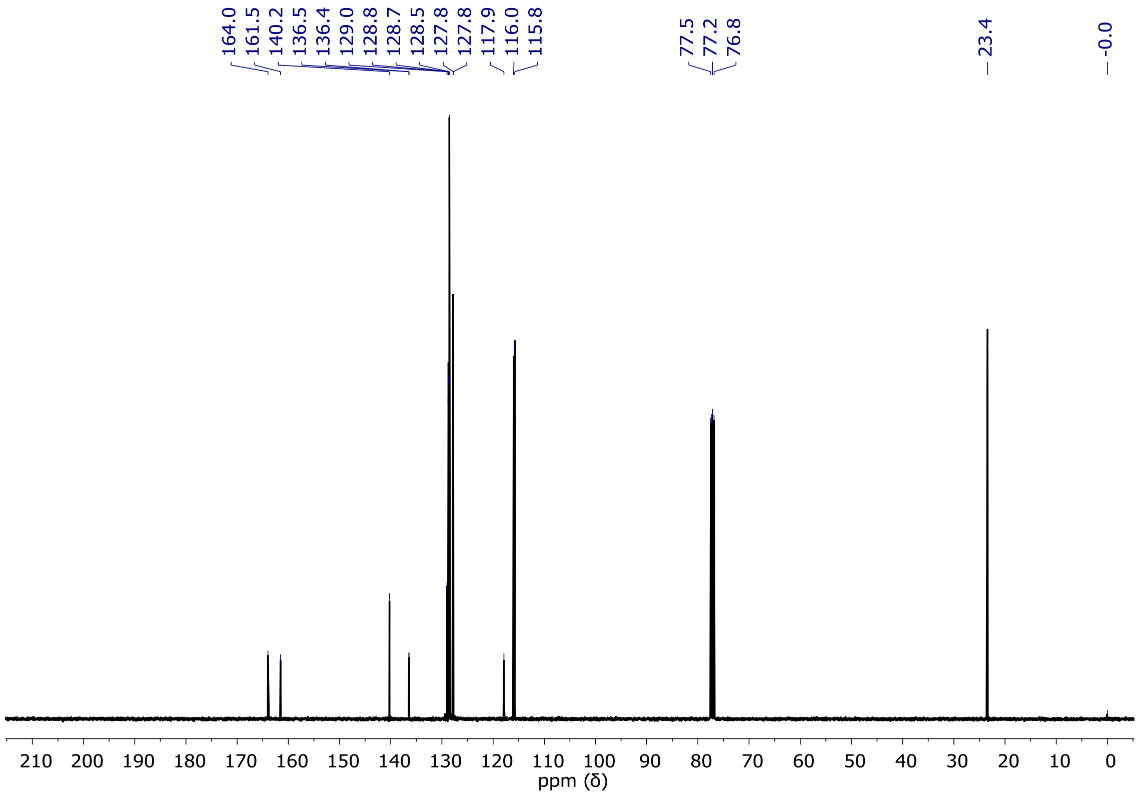

Figure S48. ^{19}F NMR Spectrum (376 MHz, $CDCl_3$) for 4-(4-fluorophenyl)benzene acetonitrile (**17**)

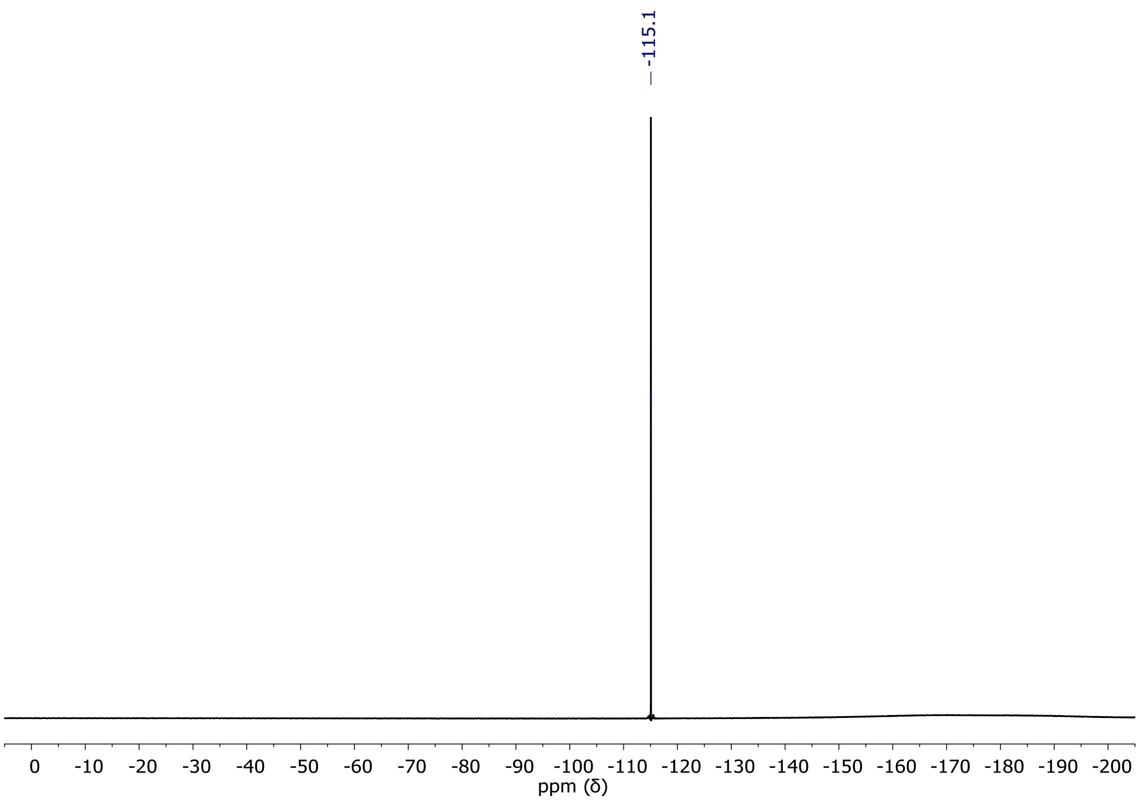

Figure S49. ^{1}H NMR Spectrum (400 MHz, $CDCl_3$) for 3-(4-fluorophenyl)pyridine (**18**)

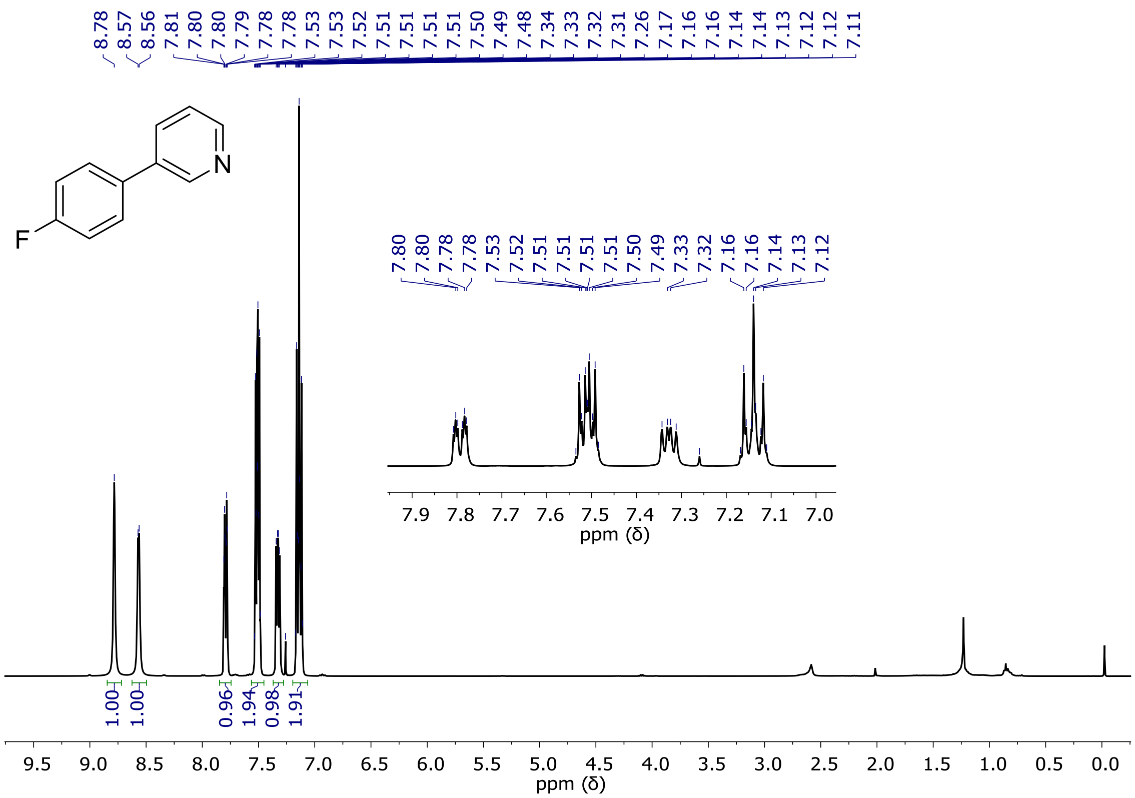

Figure S50. ^{13}C NMR Spectrum (100 MHz, $CDCl_3$) for 3-(4-fluorophenyl)pyridine (**18**)

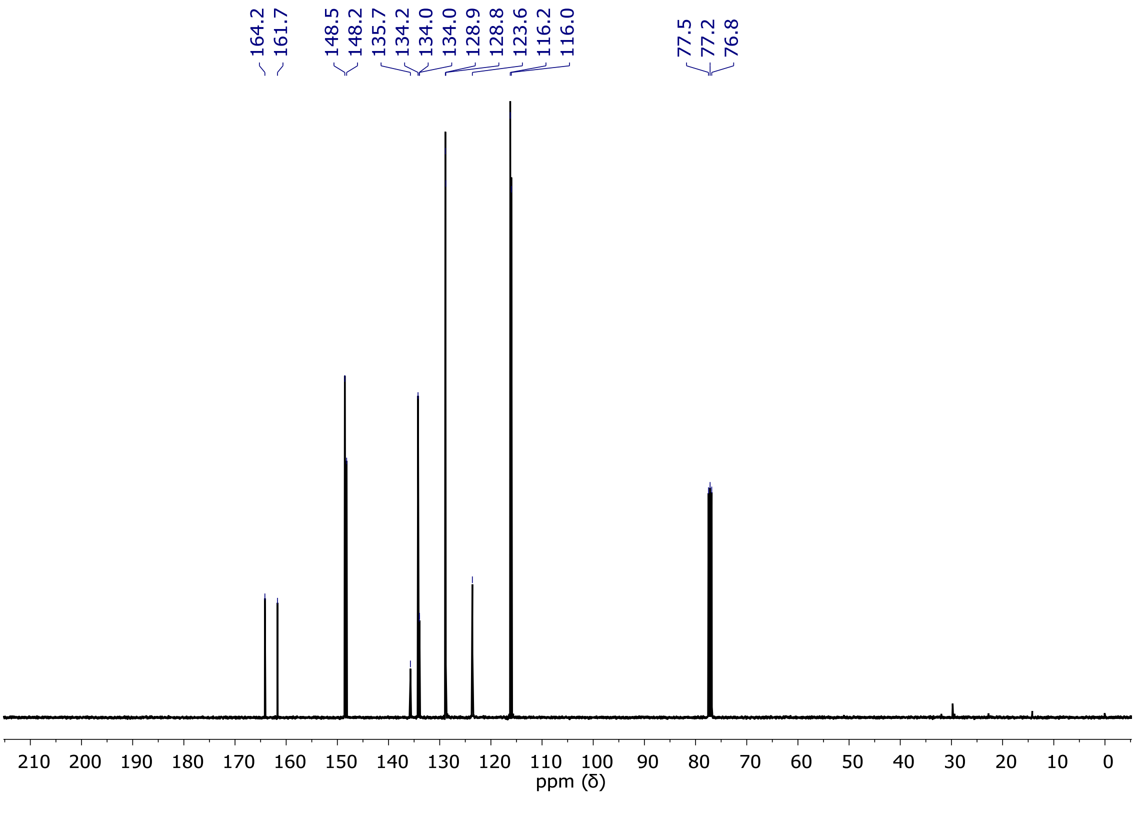

Figure S51. ^{19}F NMR Spectrum (376 MHz, $CDCl_3$) for 3-(4-fluorophenyl)pyridine (**18**)

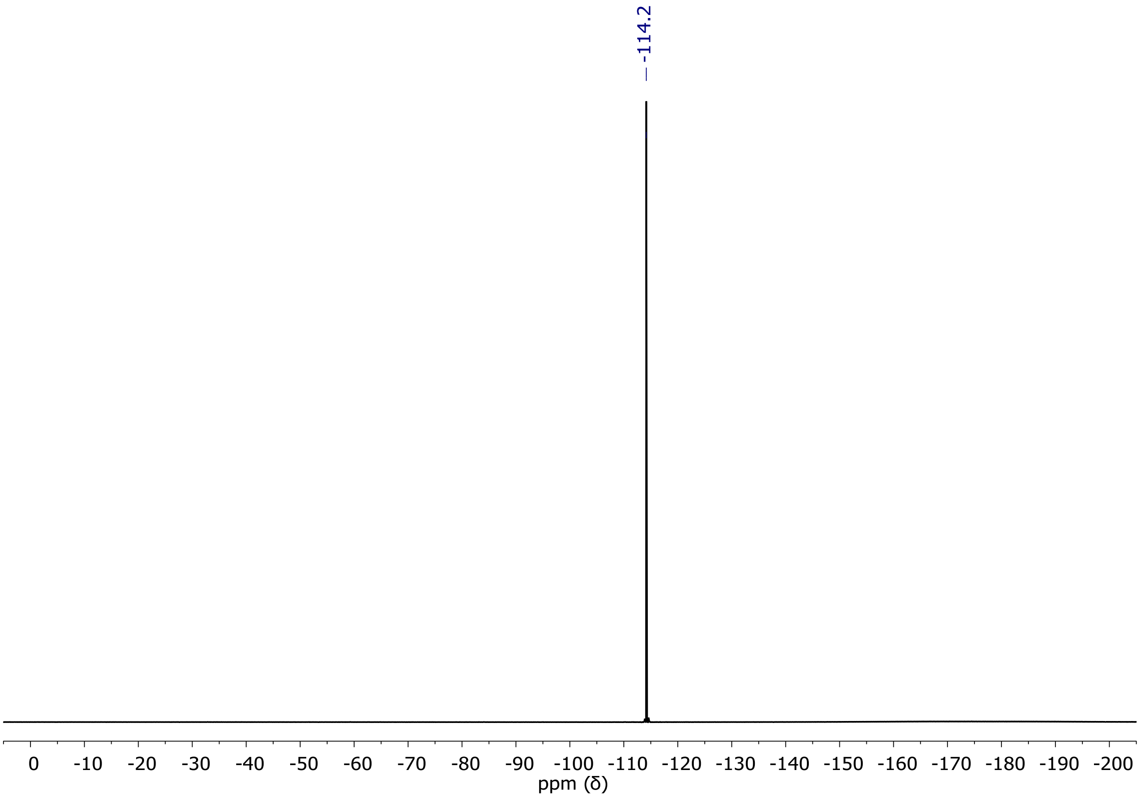

Figure S52. ^{1}H NMR Spectrum (400 MHz, $CDCl_3$) for 6-(4-fluorophenyl)piperonyl alcohol (**19**)

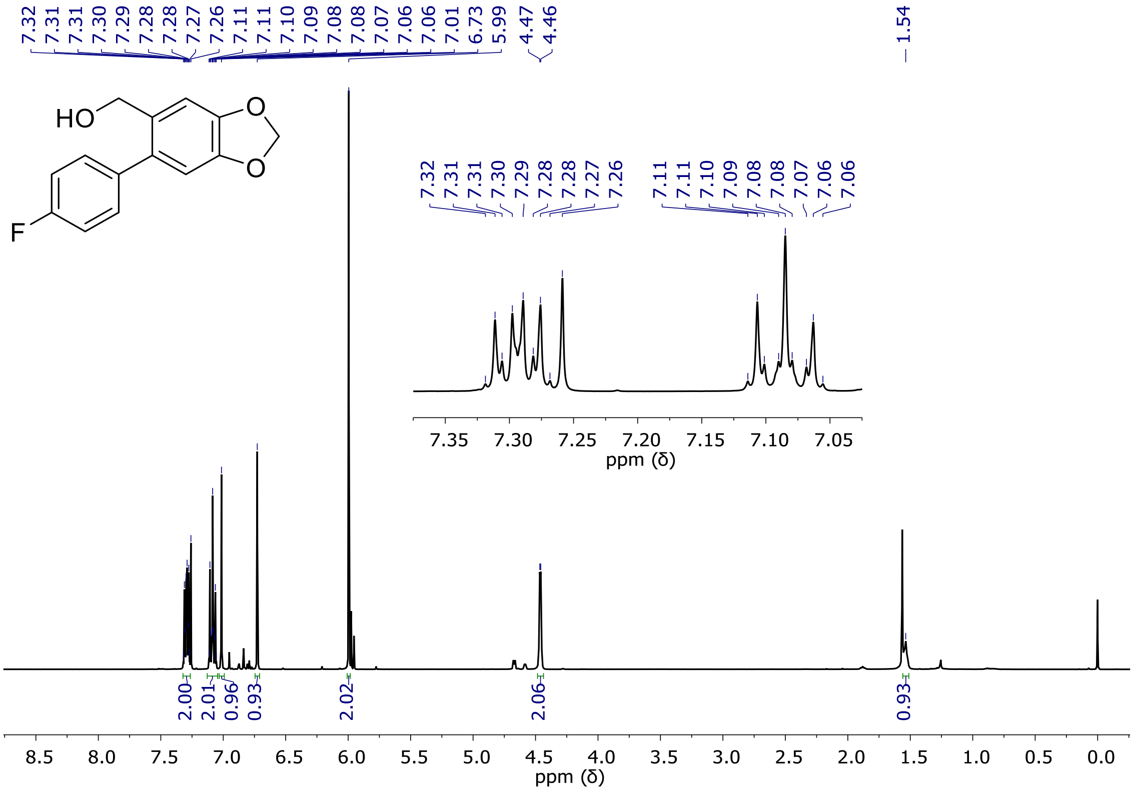

Figure S53. ^{13}C NMR Spectrum (100 MHz, $CDCl_3$) for 6-(4-fluorophenyl)piperonyl alcohol (**19**)

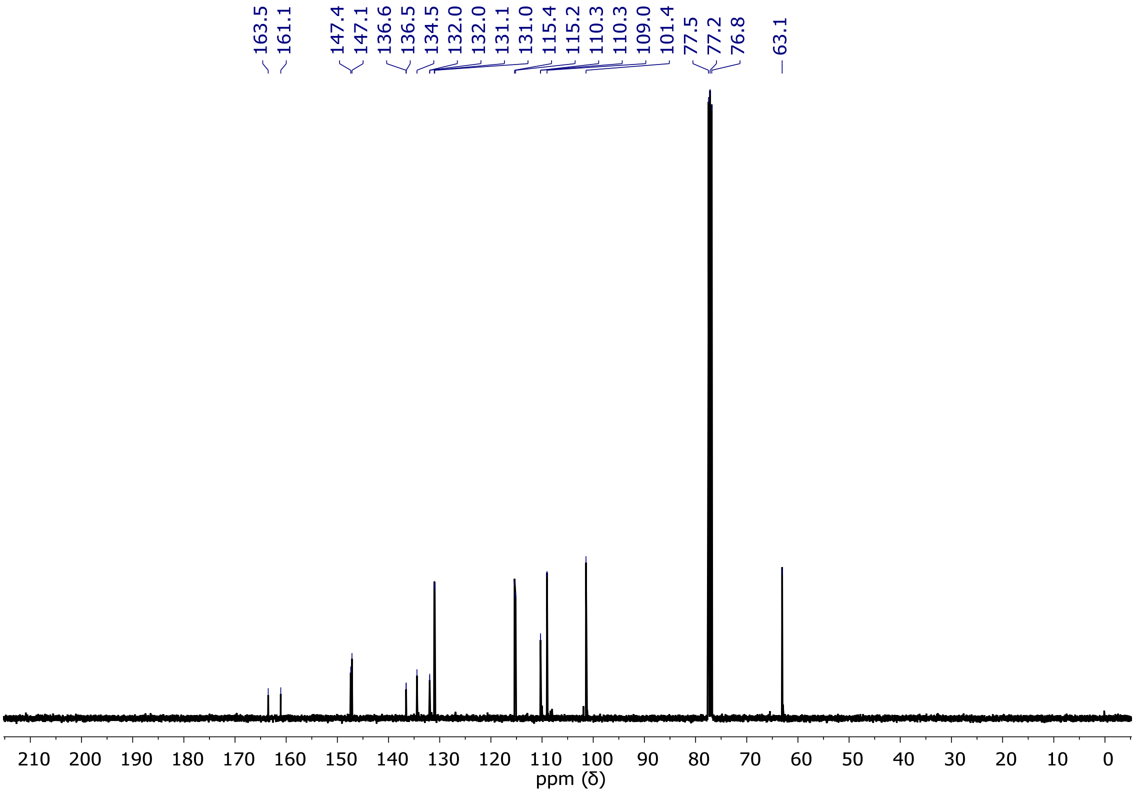

Figure S54. ^{19}F NMR Spectrum (376 MHz, $CDCl_3$) for 6-(4-fluorophenyl)piperonyl alcohol (**19**)

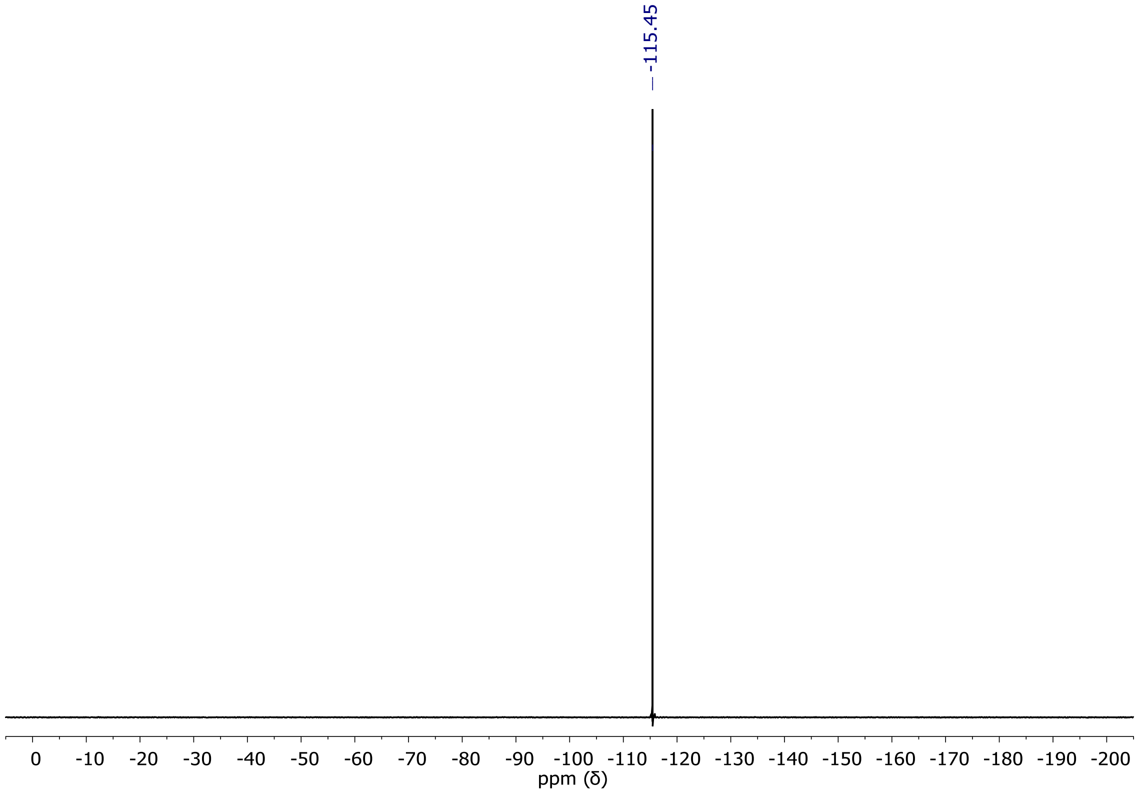

Figure S55. ^{1}H NMR Spectrum (400 MHz, $CDCl_3$) for 6-(4-fluorophenyl)piperonyl aldehyde

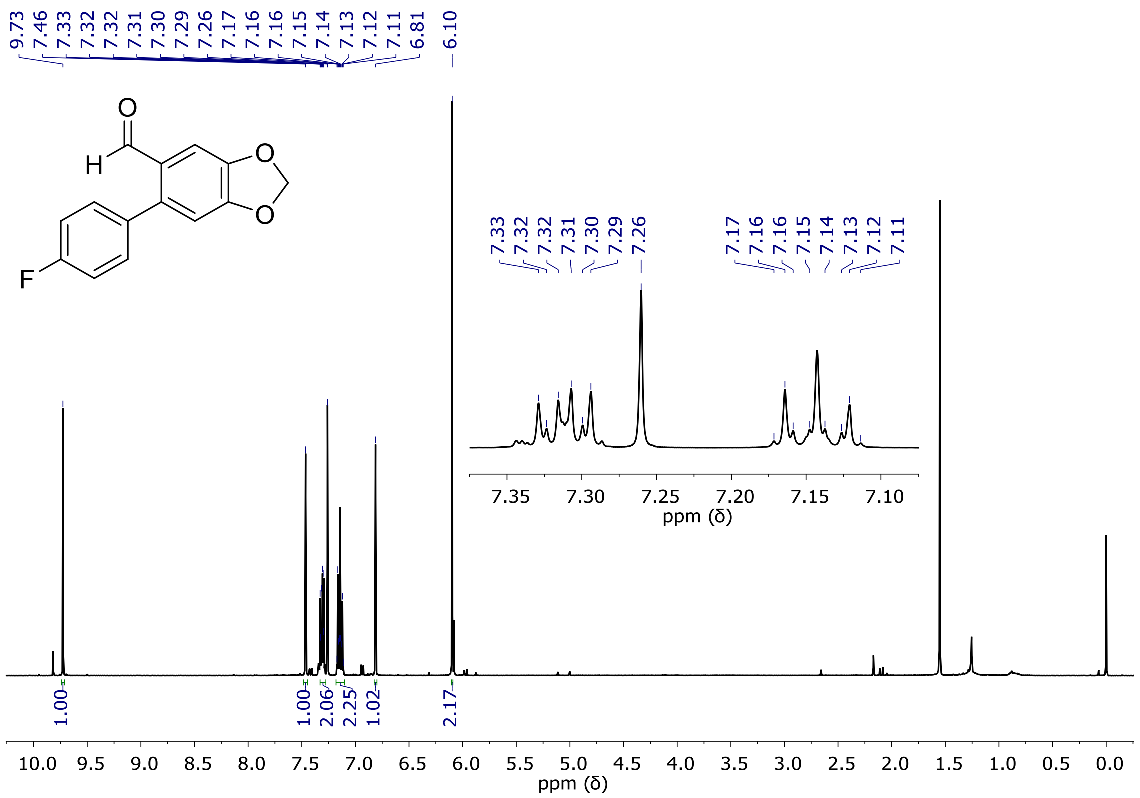

Figure S56. ^{13}C NMR Spectrum (100 MHz, $CDCl_3$) for 6-(4-fluorophenyl)piperonyl aldehyde

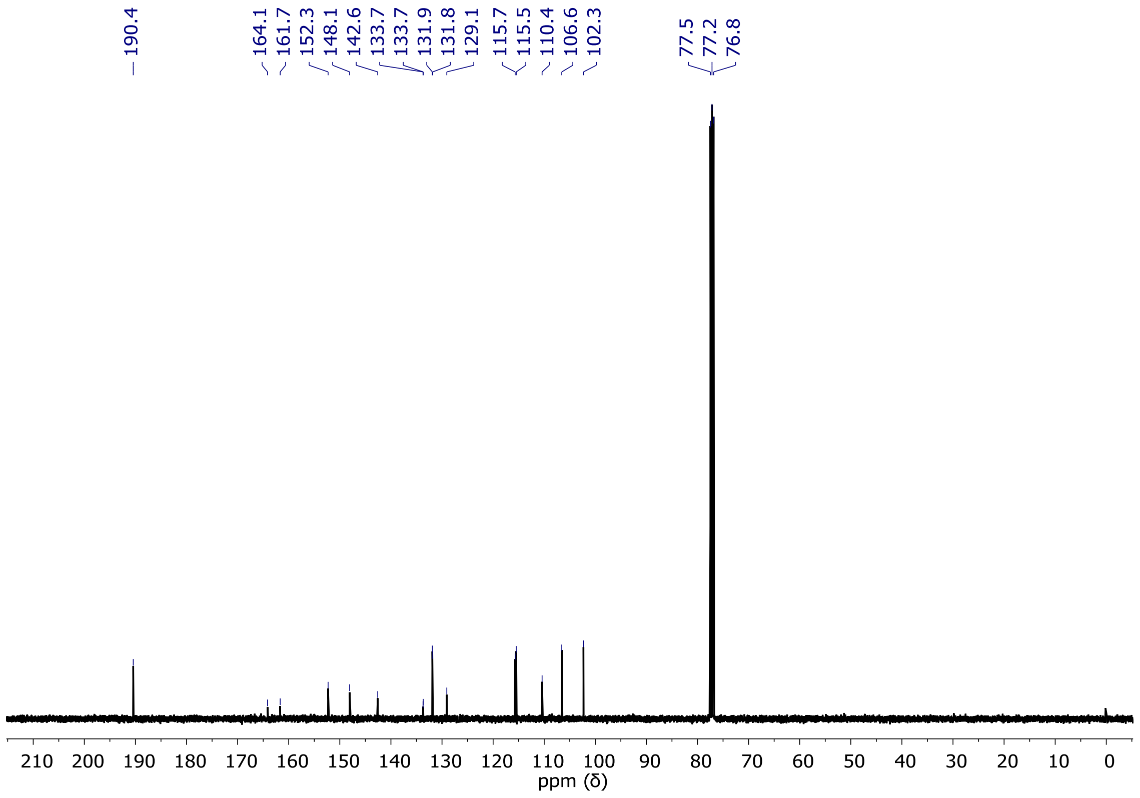

Figure S57. ^{19}F NMR Spectrum (376 MHz, $CDCl_3$) for 6-(4-fluorophenyl)piperonyl aldehyde

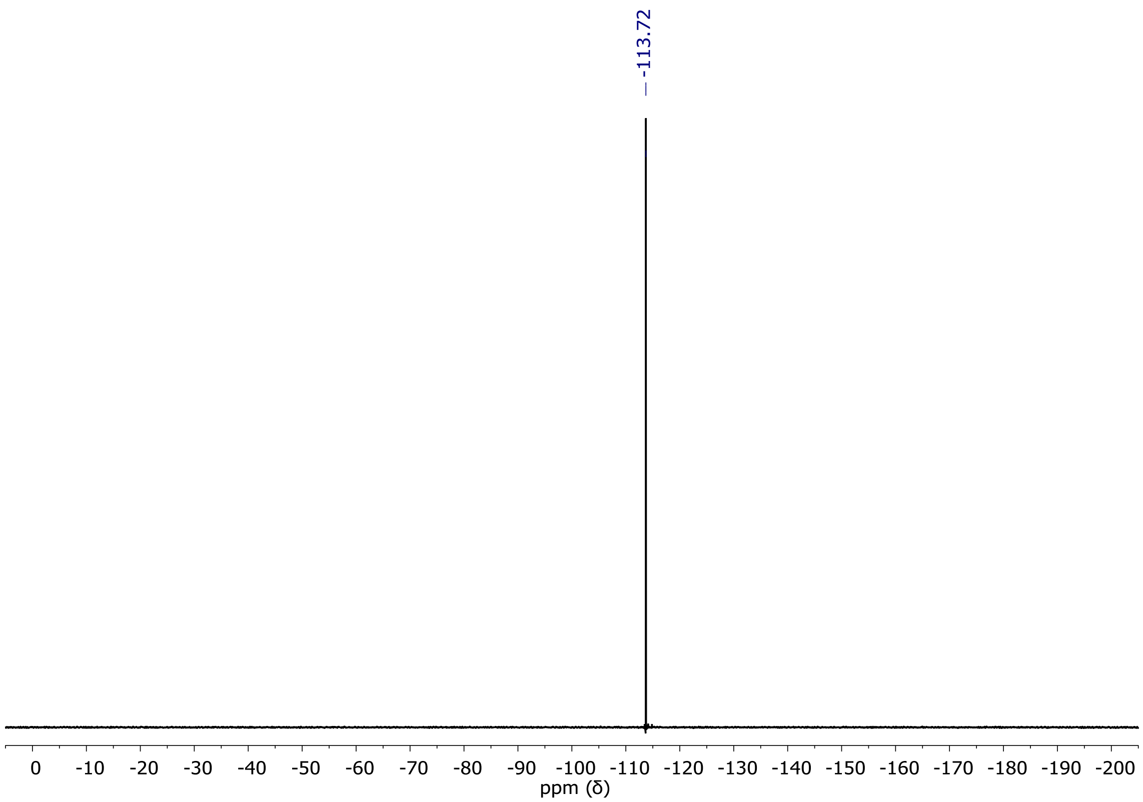

Figure S58. ^{1}H NMR Spectrum (400 MHz, $CDCl_3$) for 4'-(4-fluorophenyl)chalcone (**20**)

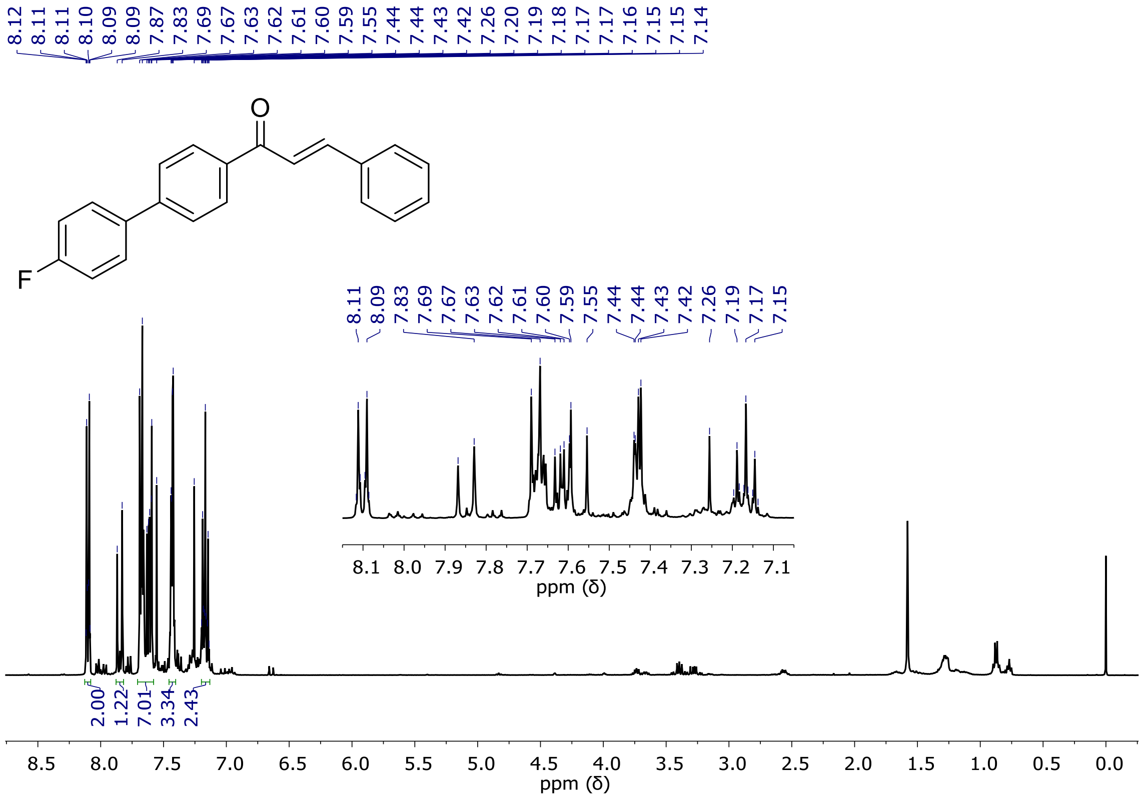

Figure S59. ^{13}C NMR Spectrum (100 MHz, $CDCl_3$) for 4'-(4-fluorophenyl)chalcone (**20**)

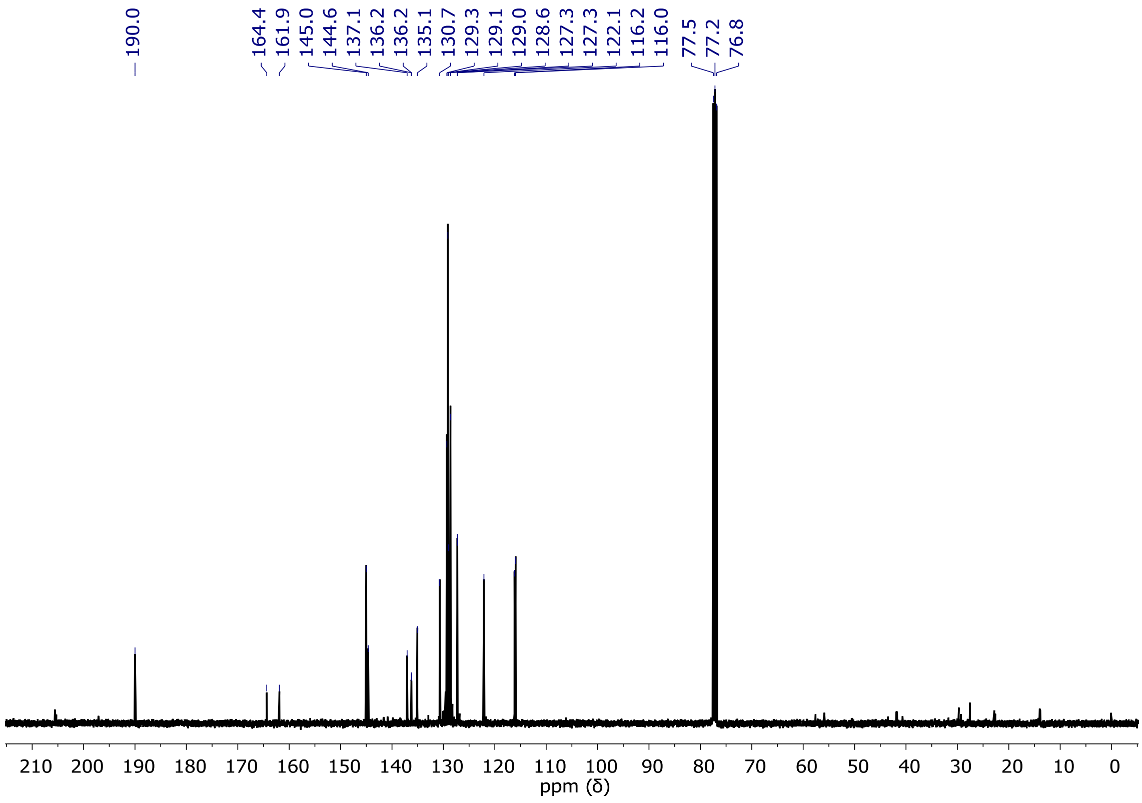

Figure S60. ^{19}F NMR Spectrum (376 MHz, $CDCl_3$) for 4'-(4-fluorophenyl)chalcone (**20**)

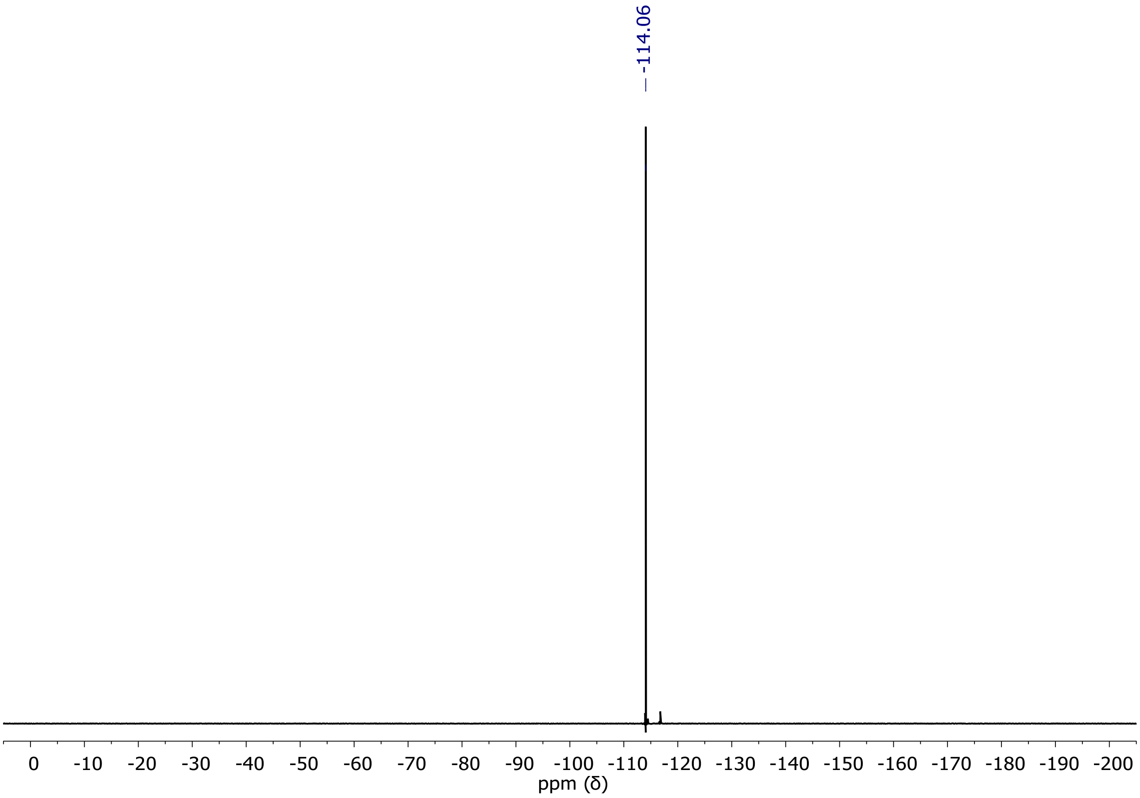

Figure S61. ^{1}H NMR Spectrum (400 MHz, $CDCl_3$) for 4-(3-fluorophenyl)acetophenone (**21**)

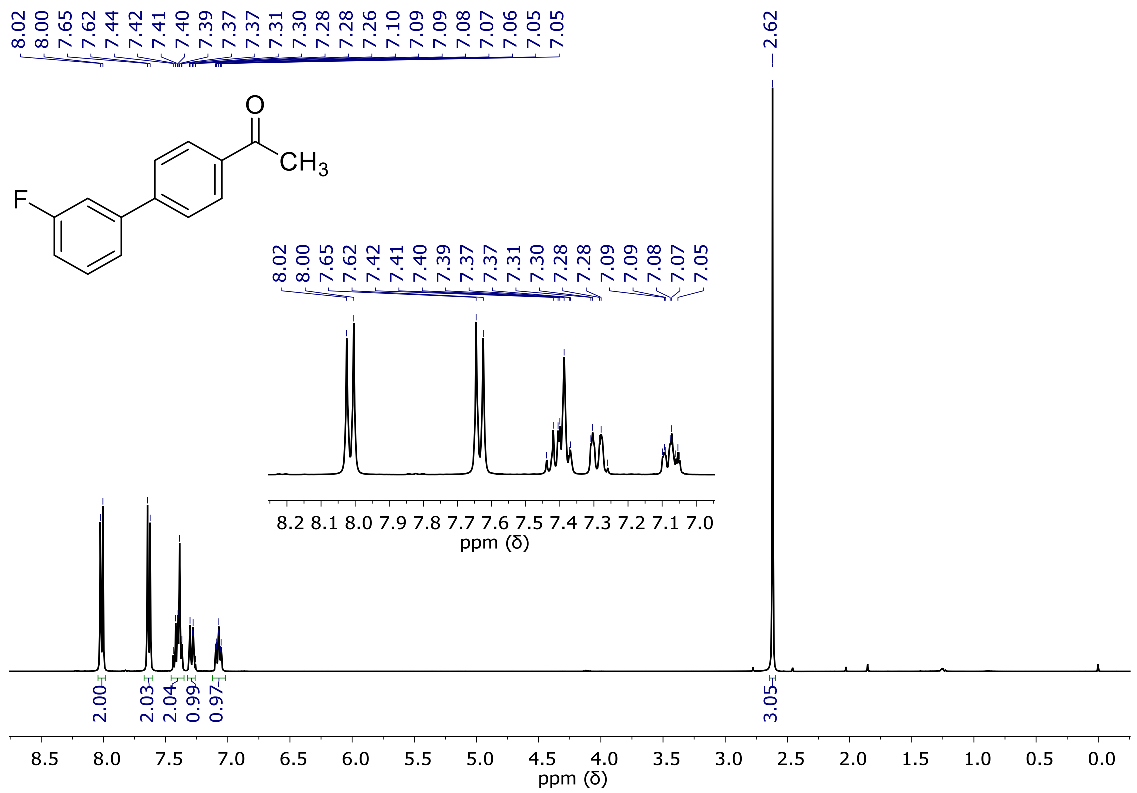

Figure S62. ^{13}C NMR Spectrum (100 MHz, $CDCl_3$) for 4-(3-fluorophenyl)acetophenone (**21**)

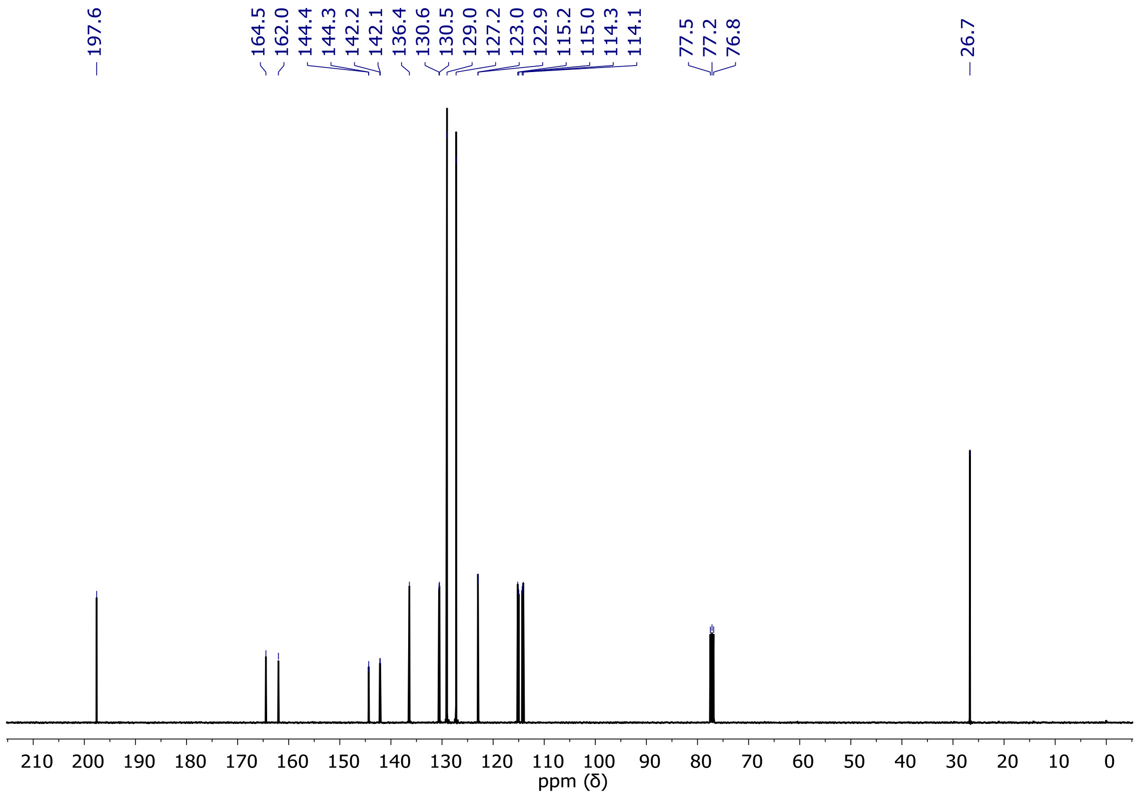

Figure S63. ^{19}F NMR Spectrum (376 MHz, $CDCl_3$) for 4-(3-fluorophenyl)acetophenone (**21**)

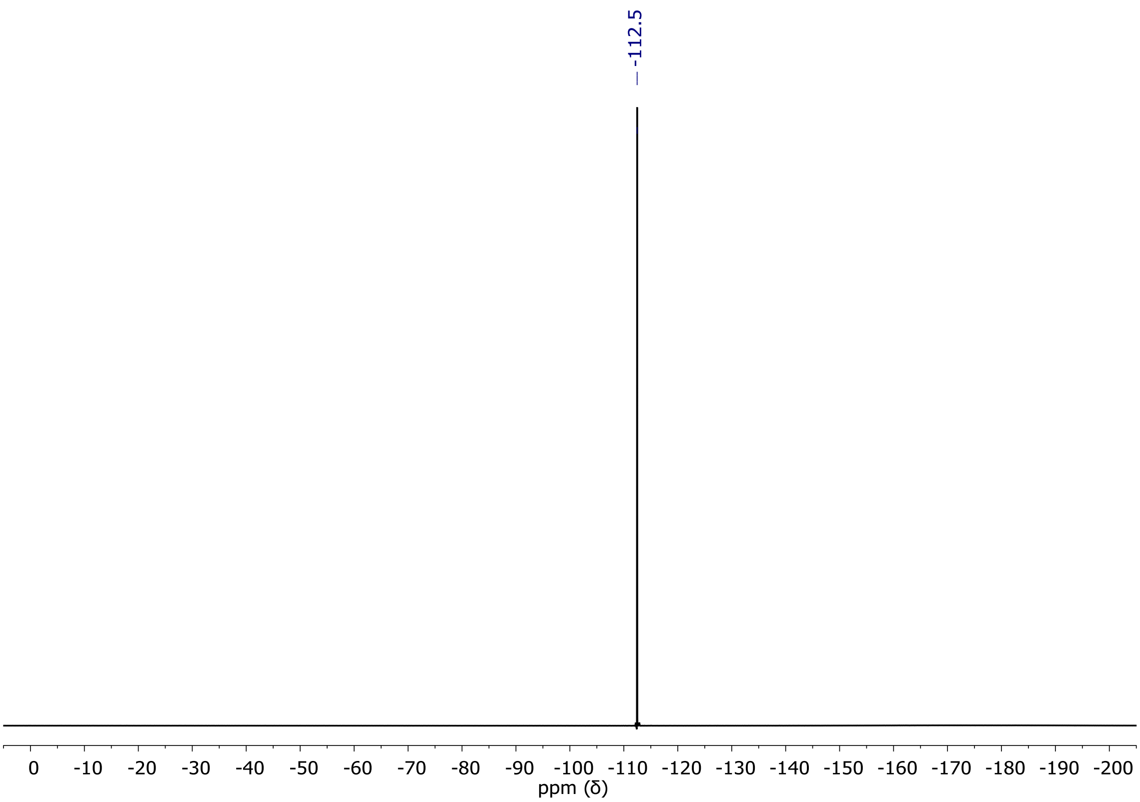

Figure S64. ^{1}H NMR Spectrum (400 MHz, $CDCl_3$) for 4-(2-fluorophenyl)acetophenone (**22**)

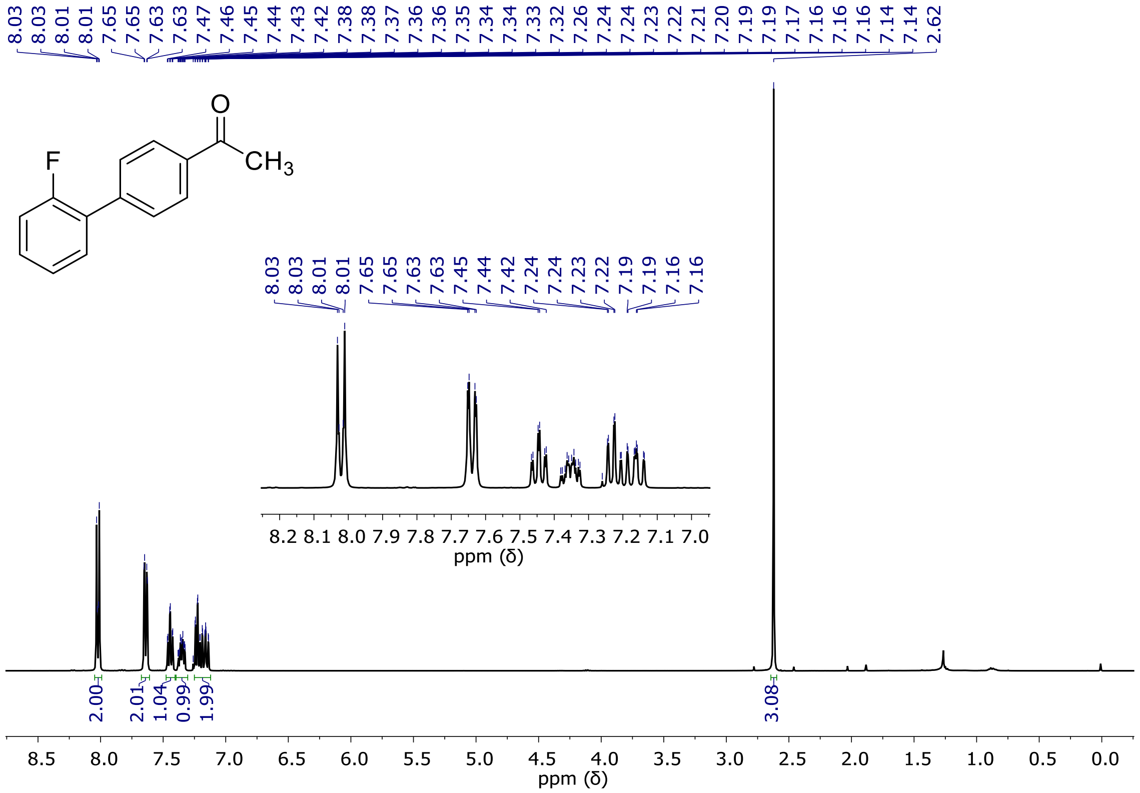

Figure S65. ^{13}C NMR Spectrum (100 MHz, $CDCl_3$) for 4-(2-fluorophenyl)acetophenone (**22**)

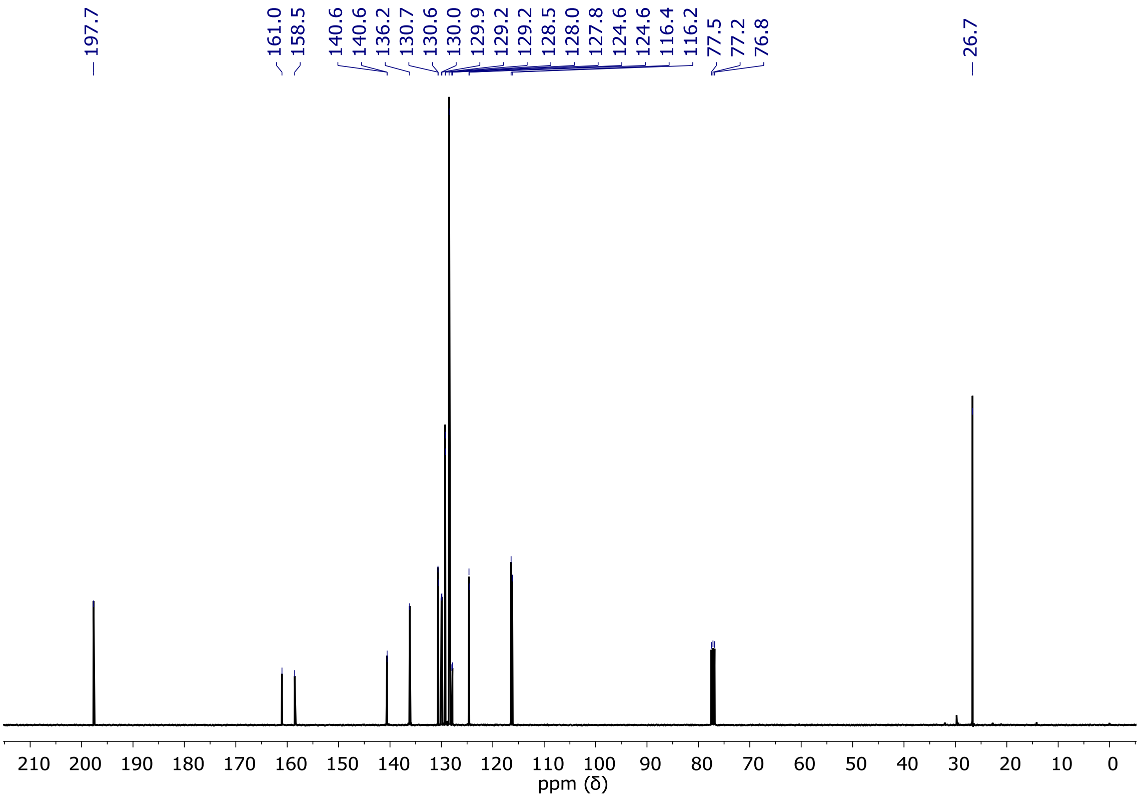

Figure S66. ^{19}F NMR Spectrum (376 MHz, $CDCl_3$) for 4-(2-fluorophenyl)acetophenone (**22**)

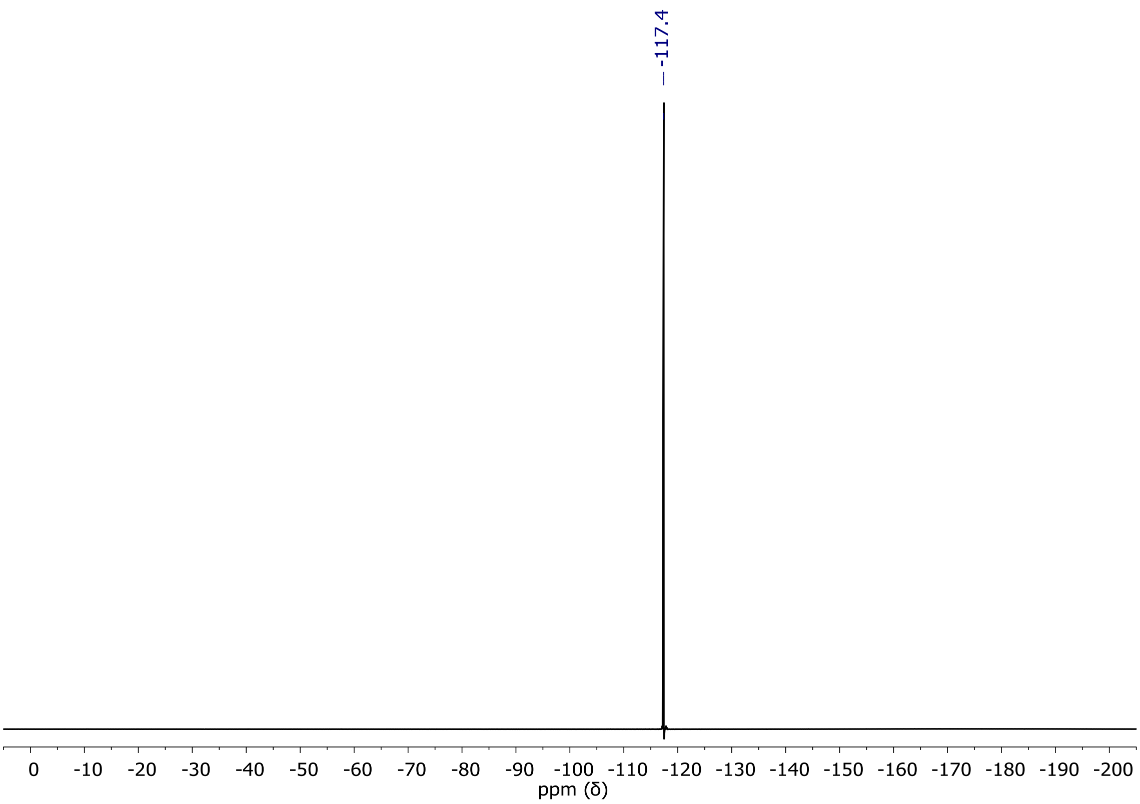

Figure S67. ^{1}H NMR Spectrum (400 MHz, $CDCl_3$) for 4-(2-naphthyl)acetophenone (**23**)

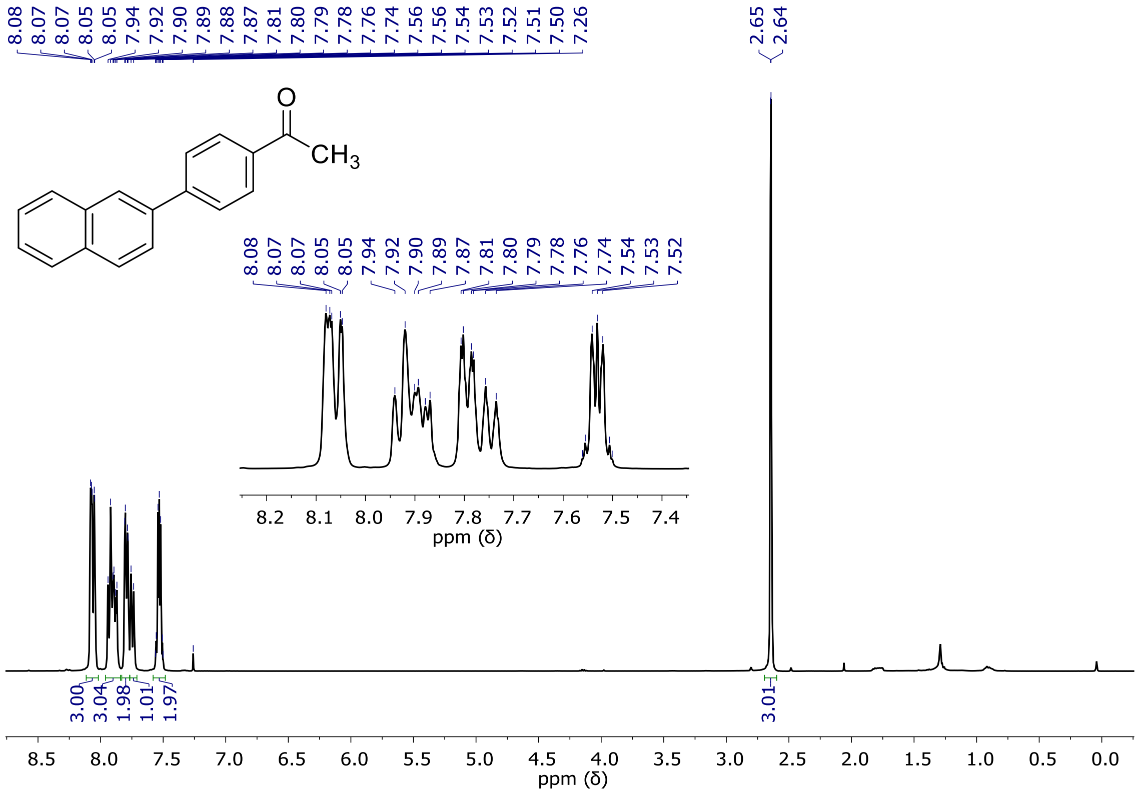

Figure S68. ^{13}C NMR Spectrum (100 MHz, $CDCl_3$) for 4-(2-naphthyl)acetophenone (**23**)

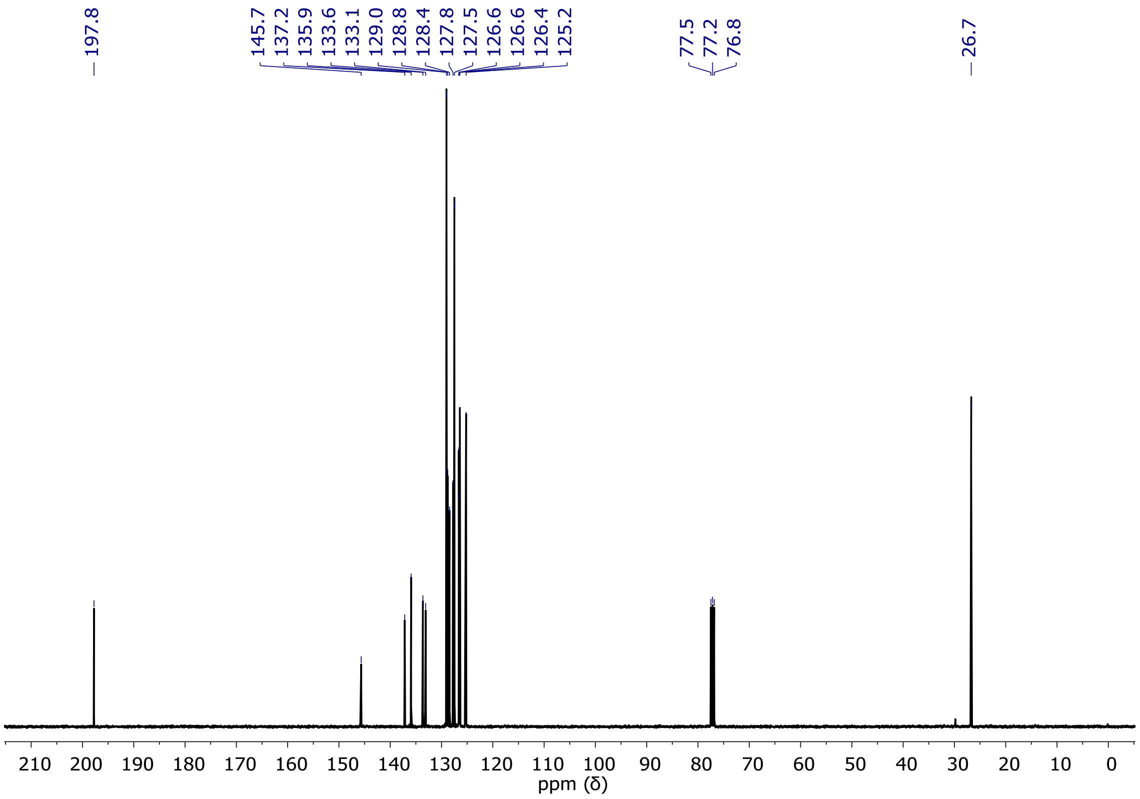

Figure S69. ^{1}H NMR Spectrum (400 MHz, $CDCl_3$) for 4-(4-isopropoxyphenyl) acetophenone (**24**)

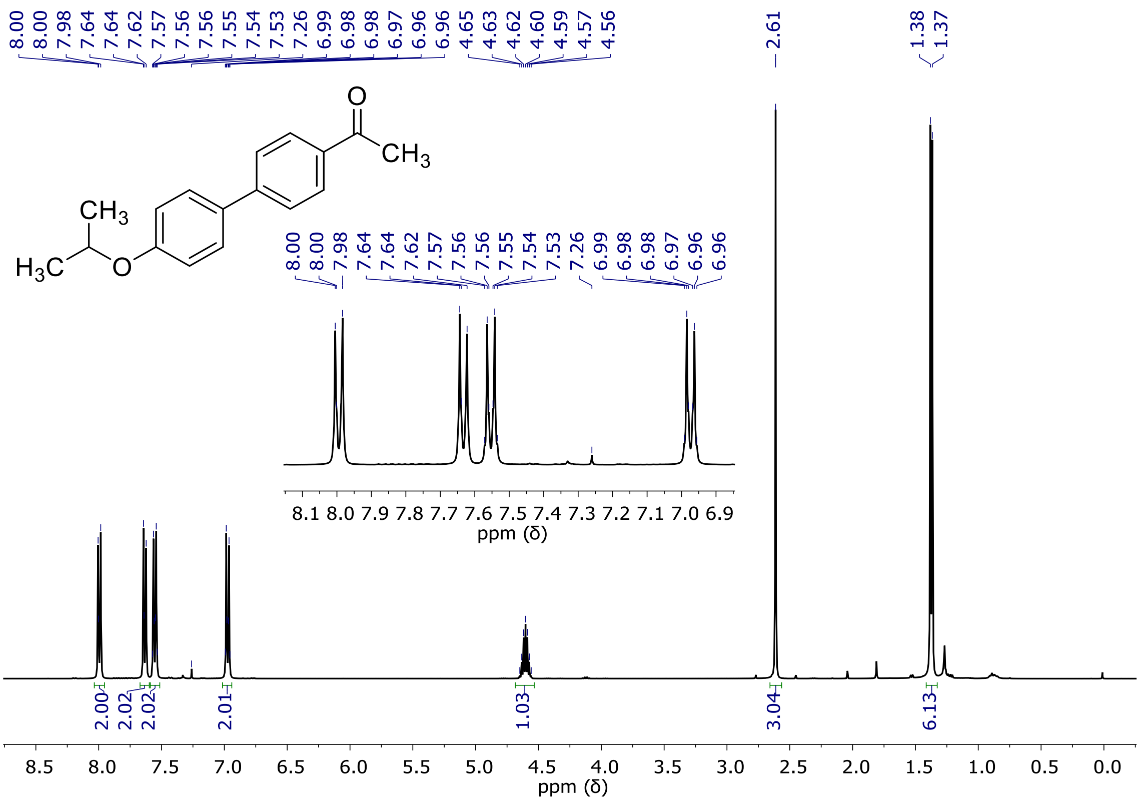

Figure S70. ^{13}C NMR Spectrum (100 MHz, $CDCl_3$) for 4-(4-isopropoxyphenyl) acetophenone (**24**)

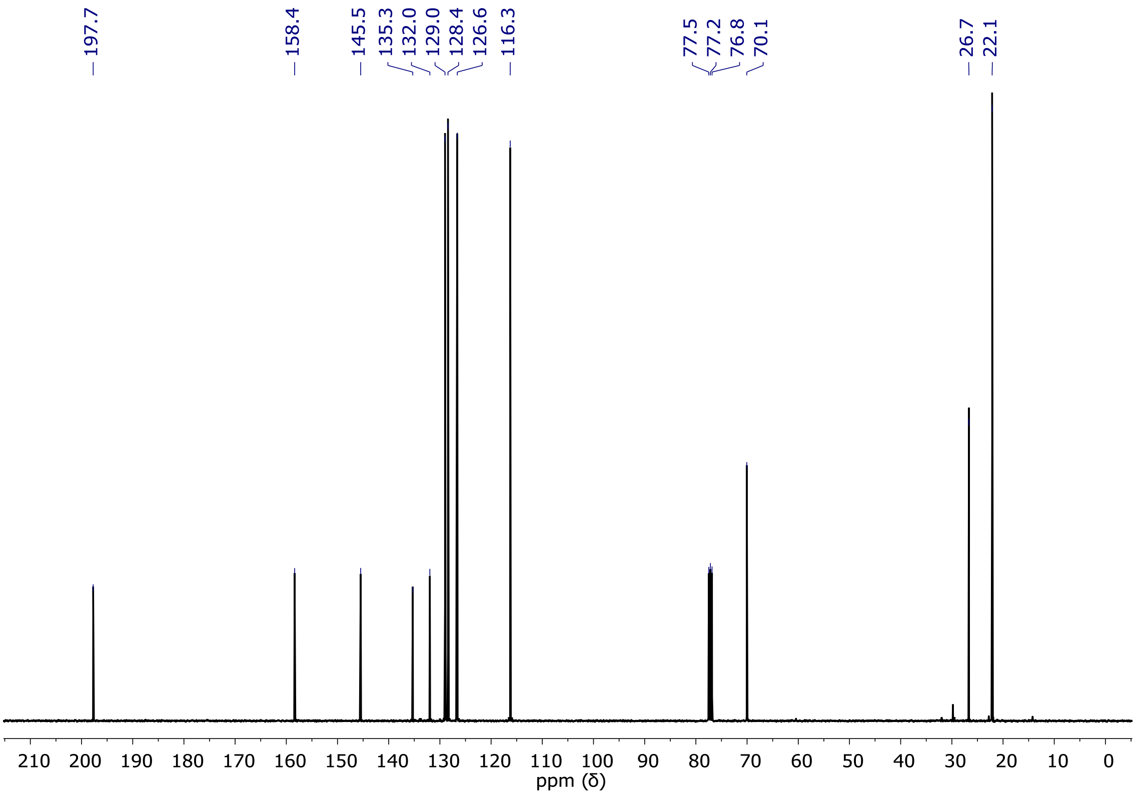

Figure S71. ^{1}H NMR Spectrum (400 MHz, $CDCl_3$) for 4-(3-isopropoxyphenyl) acetophenone (**25**)

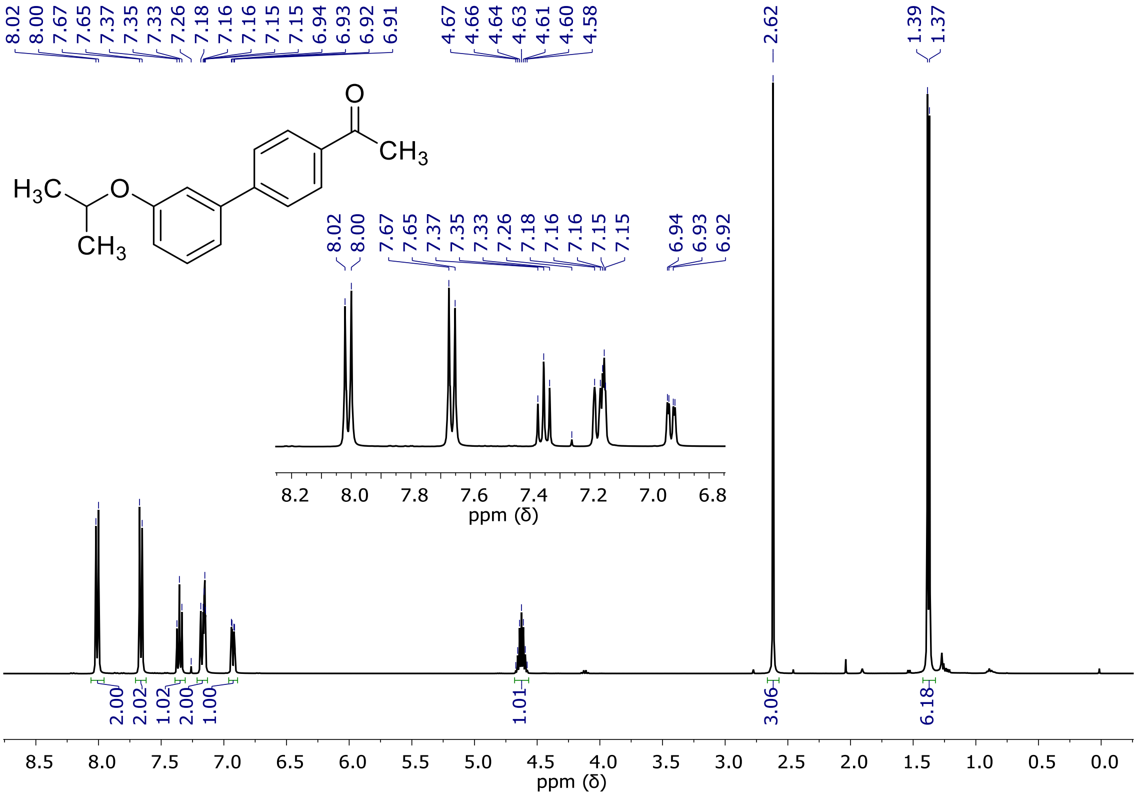

Figure S72. ^{13}C NMR Spectrum (100 MHz, $CDCl_3$) for 4-(3-isopropoxyphenyl) acetophenone (**25**)

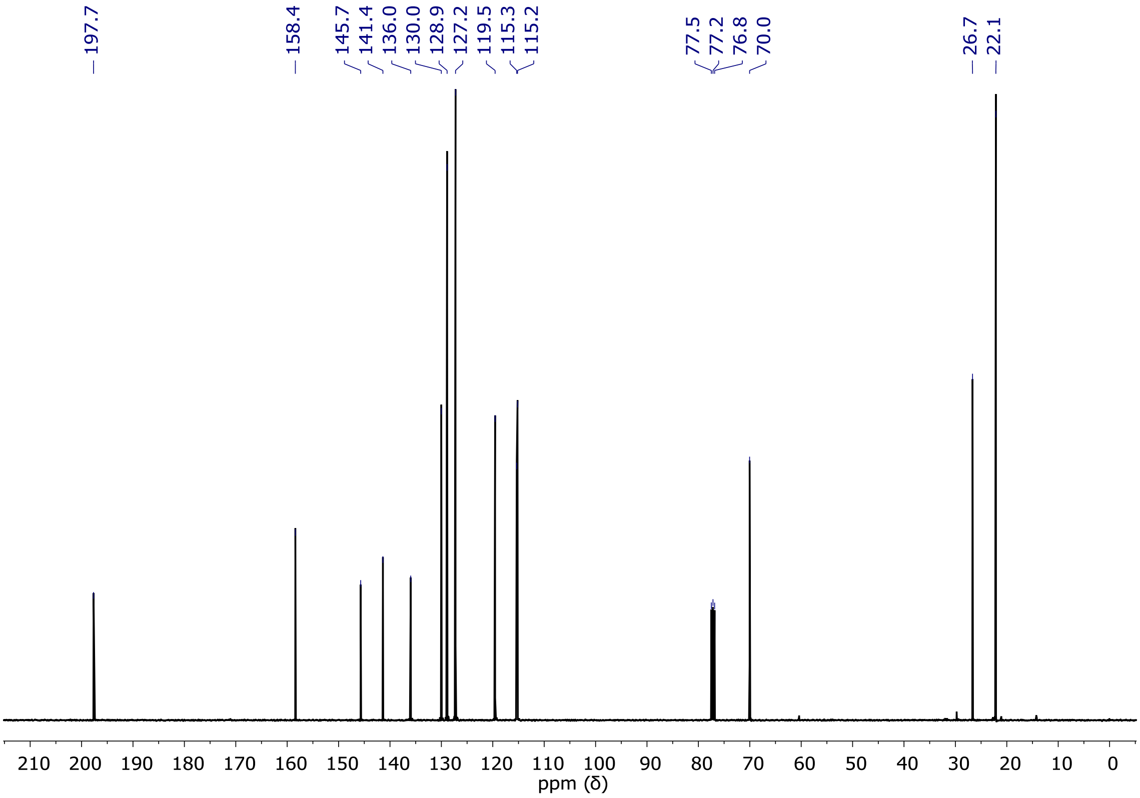

Figure S73. ^{1}H NMR Spectrum (400 MHz, $CDCl_3$) for 4-(2-isopropoxyphenyl) acetophenone (**26**)

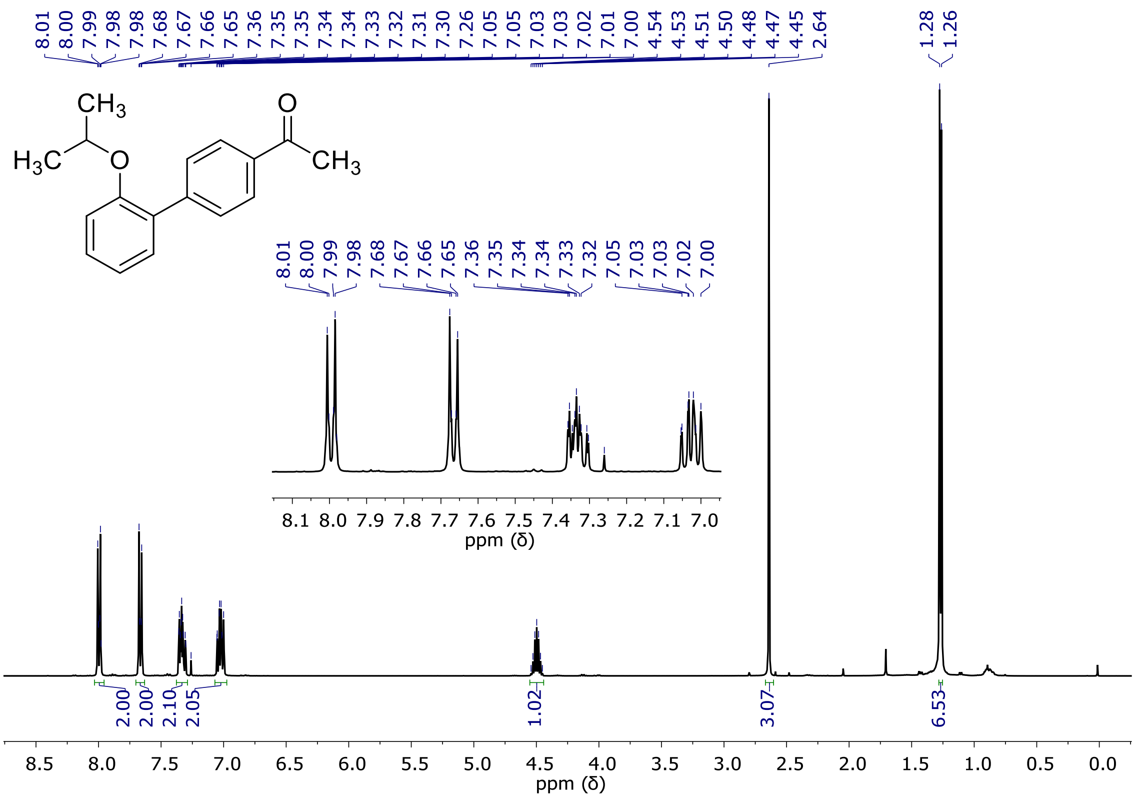

Figure S74. ^{13}C NMR Spectrum (100 MHz, $CDCl_3$) for 4-(2-isopropoxyphenyl) acetophenone (**26**)

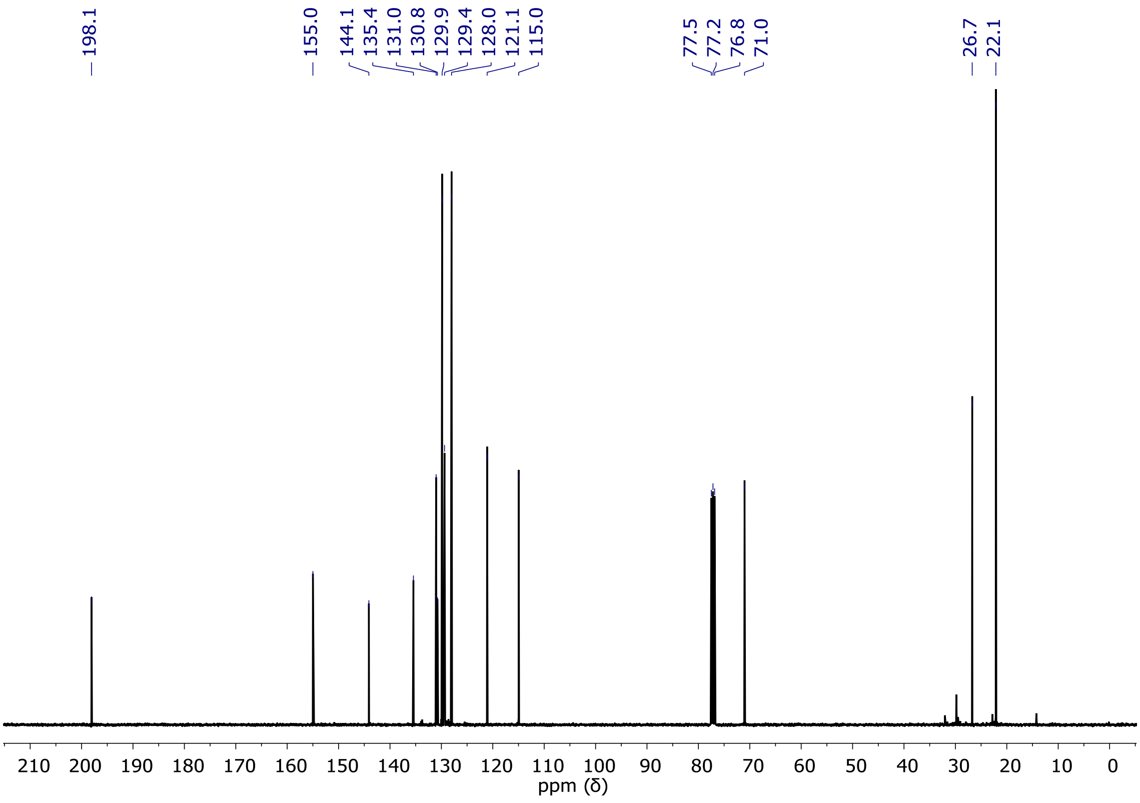

Figure S75. ^{1}H NMR Spectrum (400 MHz, $CDCl_3$) for 4-(4-phenoxyphenyl) acetophenone (**27**)

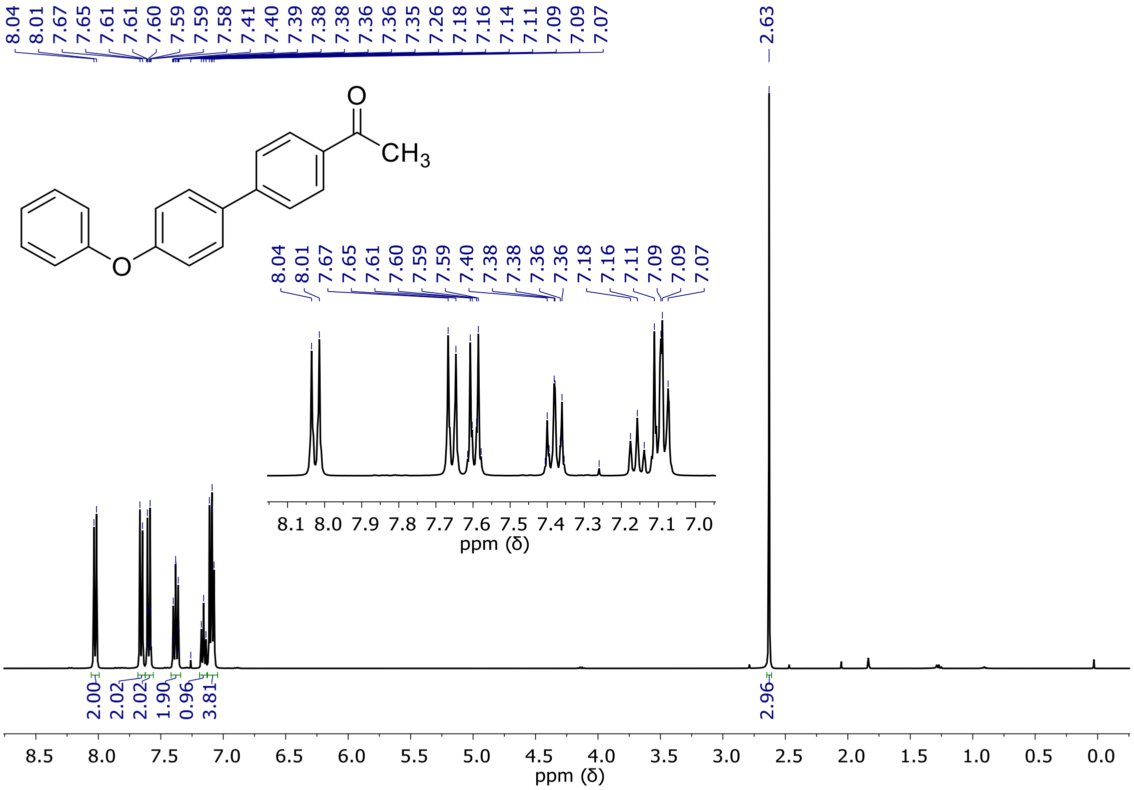

Figure S76. ^{13}C NMR Spectrum (100 MHz, $CDCl_3$) for 4-(4-phenoxyphenyl) acetophenone (**27**)

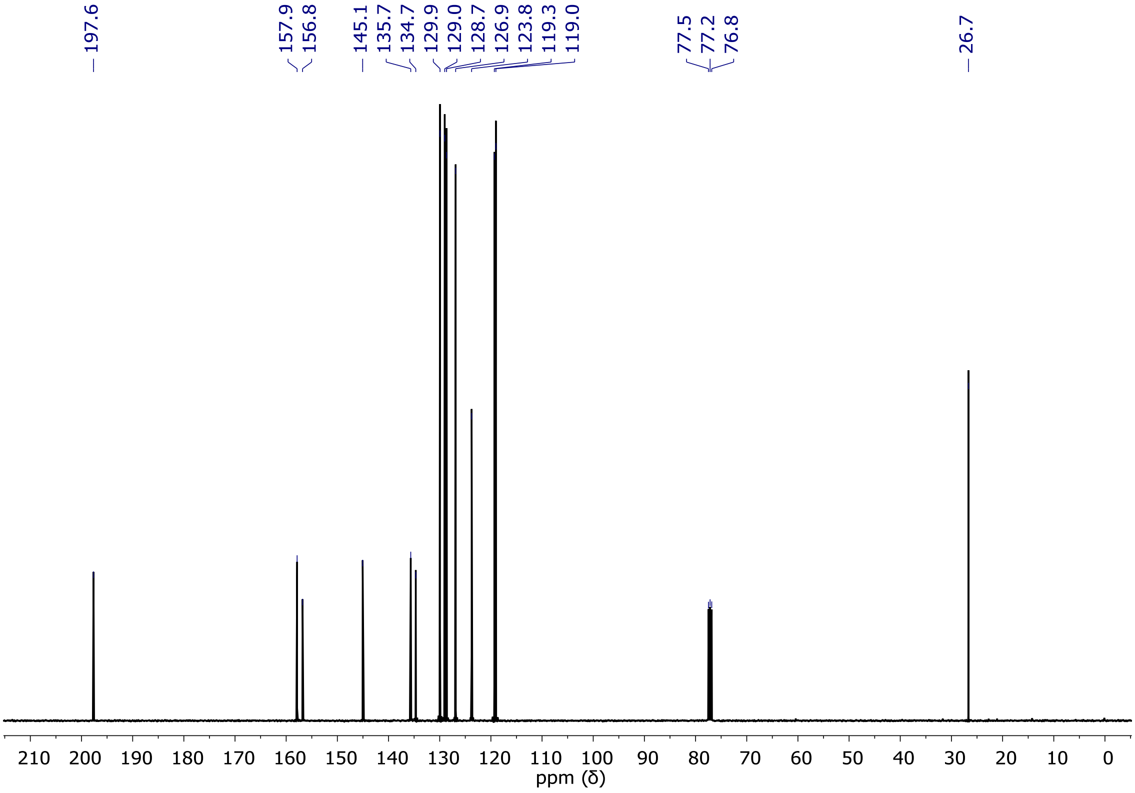

Figure S77. ^{1}H NMR Spectrum (400 MHz, $CDCl_3$) for 4-(4-ethoxycarbonylphenyl) acetophenone (**28**)

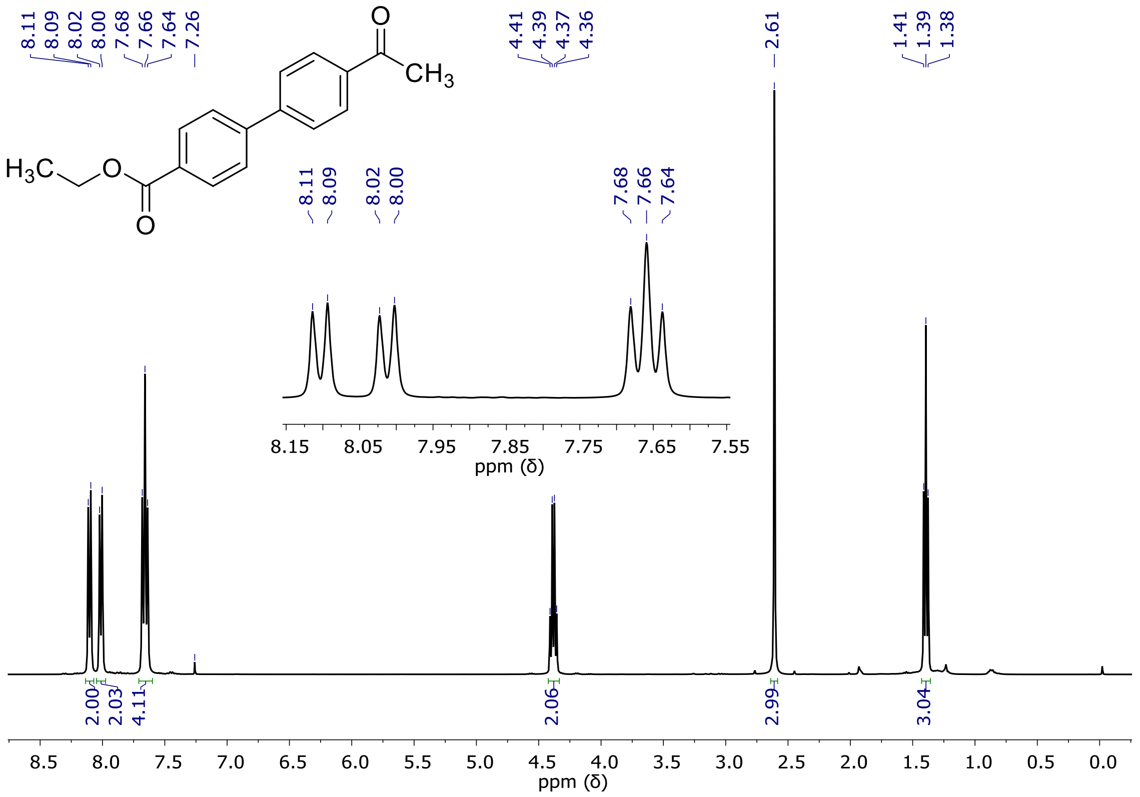

Figure S78. ^{13}C NMR Spectrum (100 MHz, $CDCl_3$) for 4-(4-ethoxycarbonylphenyl) acetophenone (**28**)

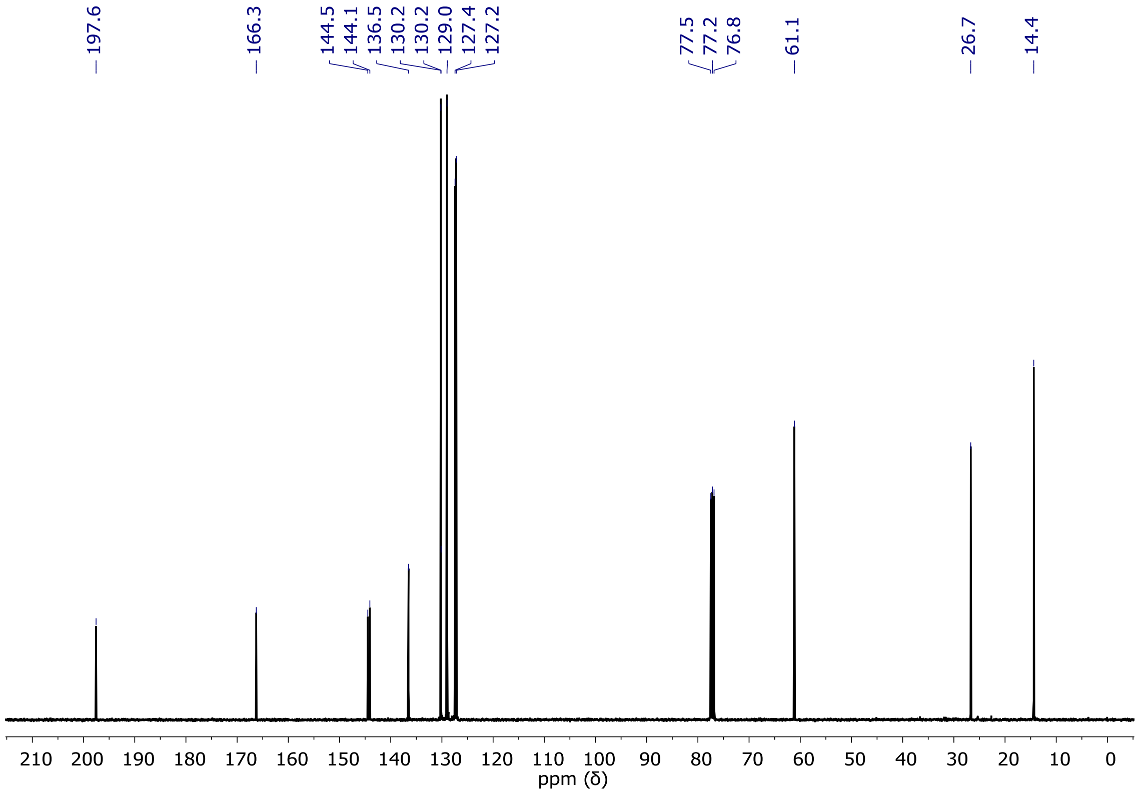

Figure S79. ^{1}H NMR Spectrum (400 MHz, $CDCl_3$) for 4-(4-cyanophenyl)acetophenone (**29**)

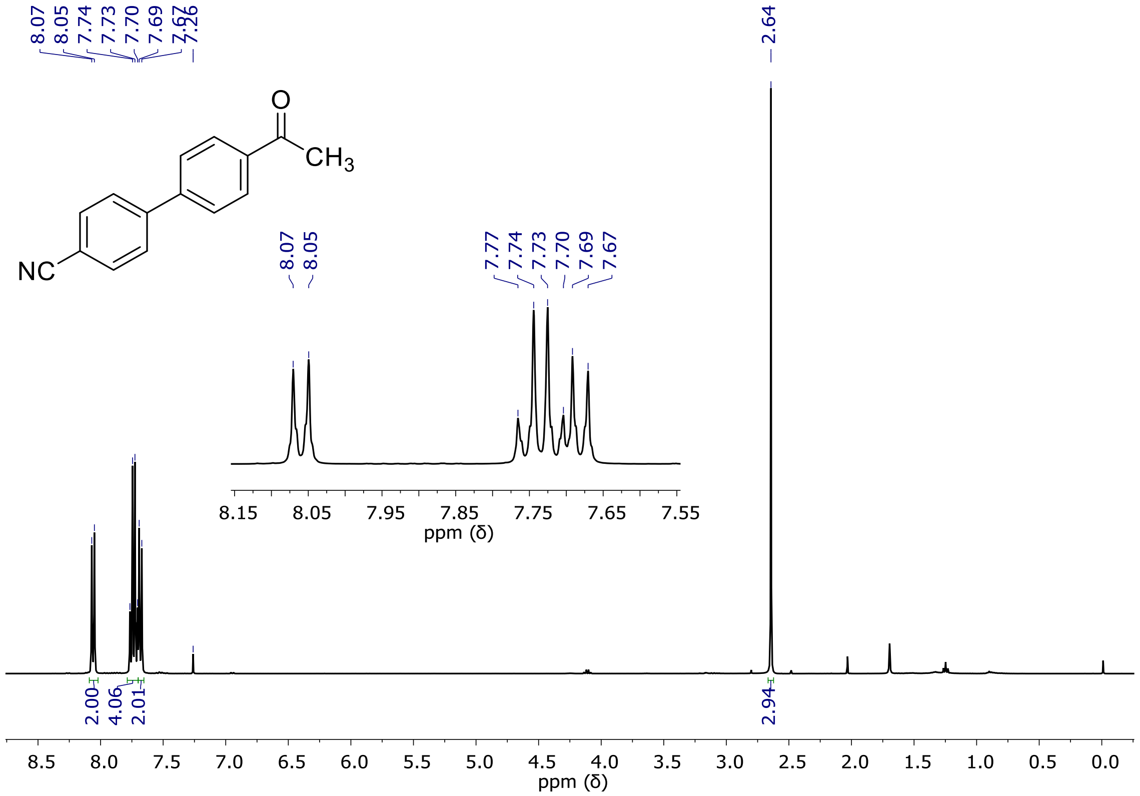

Figure S80. ^{13}C NMR Spectrum (100 MHz, $CDCl_3$) for 4-(4-cyanophenyl)acetophenone (**29**)

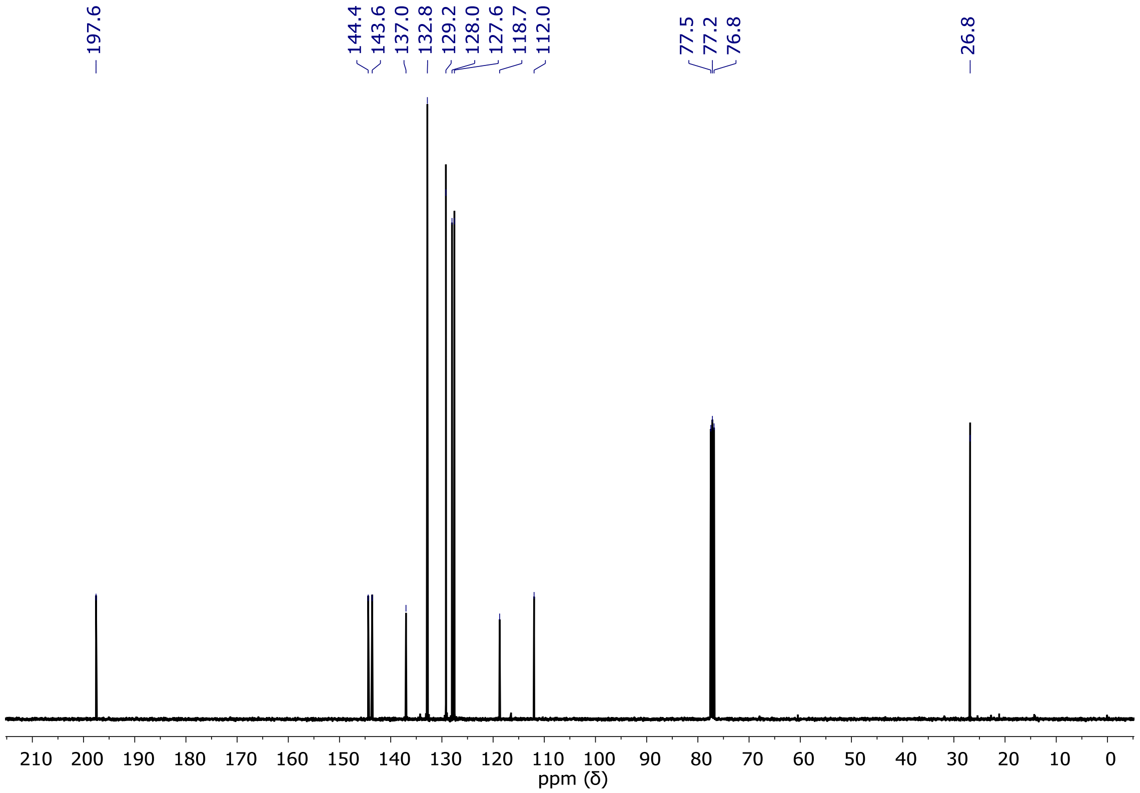

Figure S81. ^{1}H NMR Spectrum (400 MHz, $CDCl_3$) for 4-(3,4-(methylenedioxy)phenyl) acetophenone (**30**)

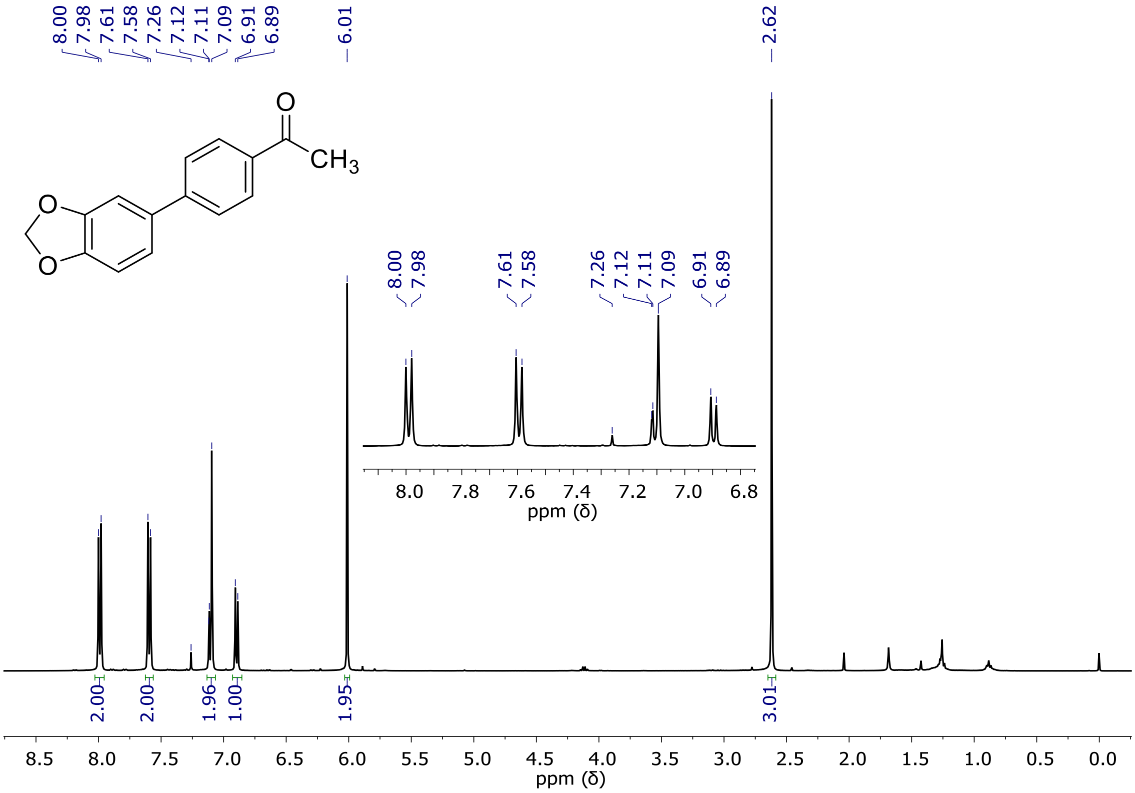

Figure S82. ^{13}C NMR Spectrum (100 MHz, $CDCl_3$) for 4-(3,4-(methylenedioxy)phenyl) acetophenone (**30**)

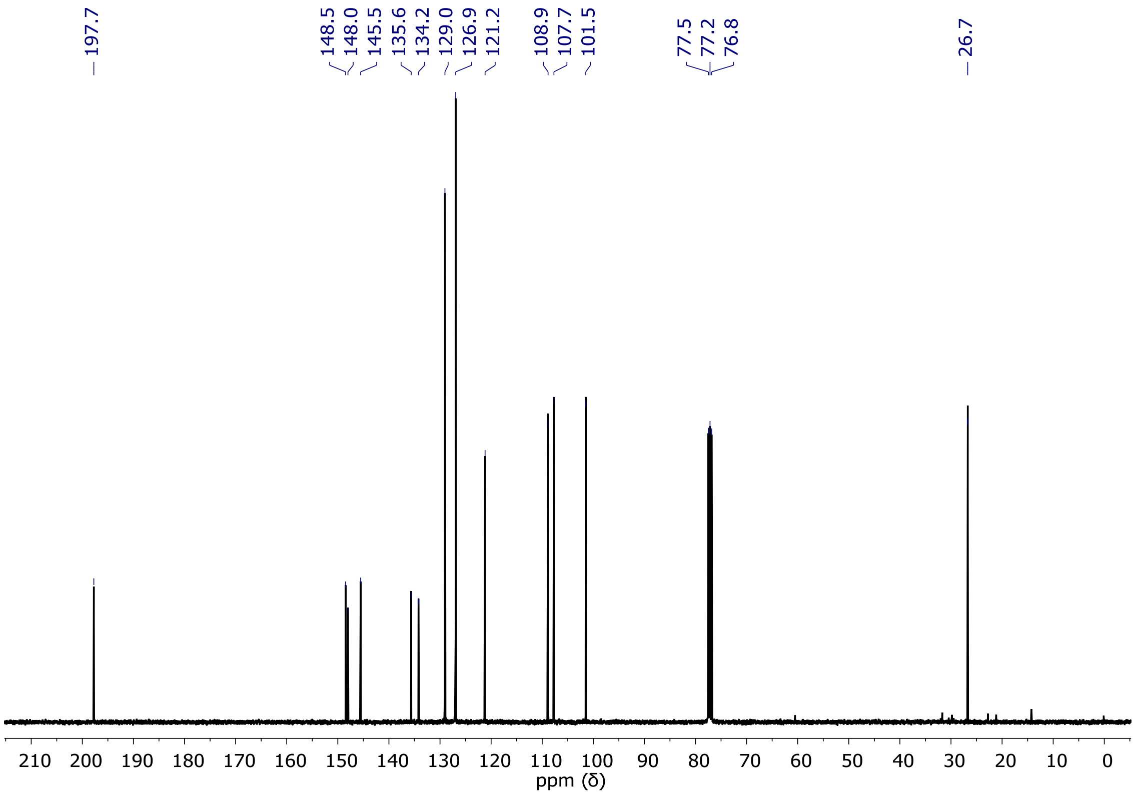

Figure S83. ^{1}H NMR Spectrum (400 MHz, $CDCl_3$) for 4-(2-furanyl)acetophenone (**31**)

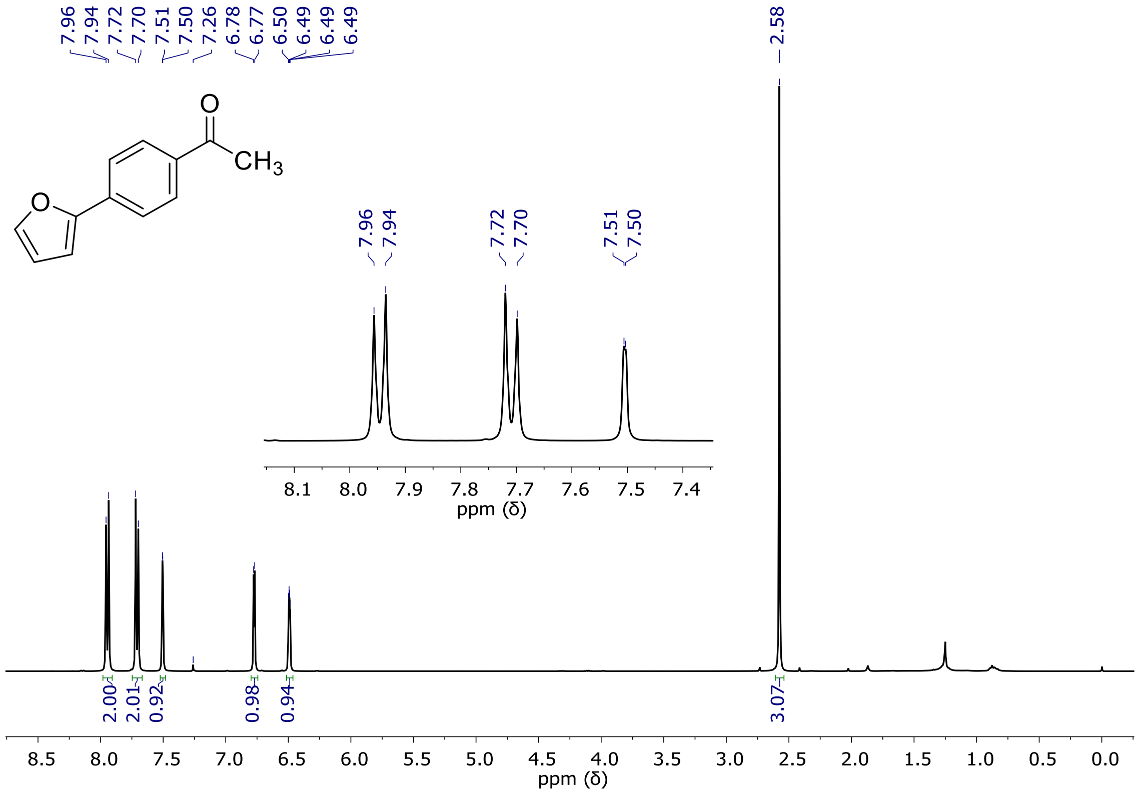

Figure S84. ^{13}C NMR Spectrum (100 MHz, $CDCl_3$) for 4-(2-furanyl)acetophenone (**31**)

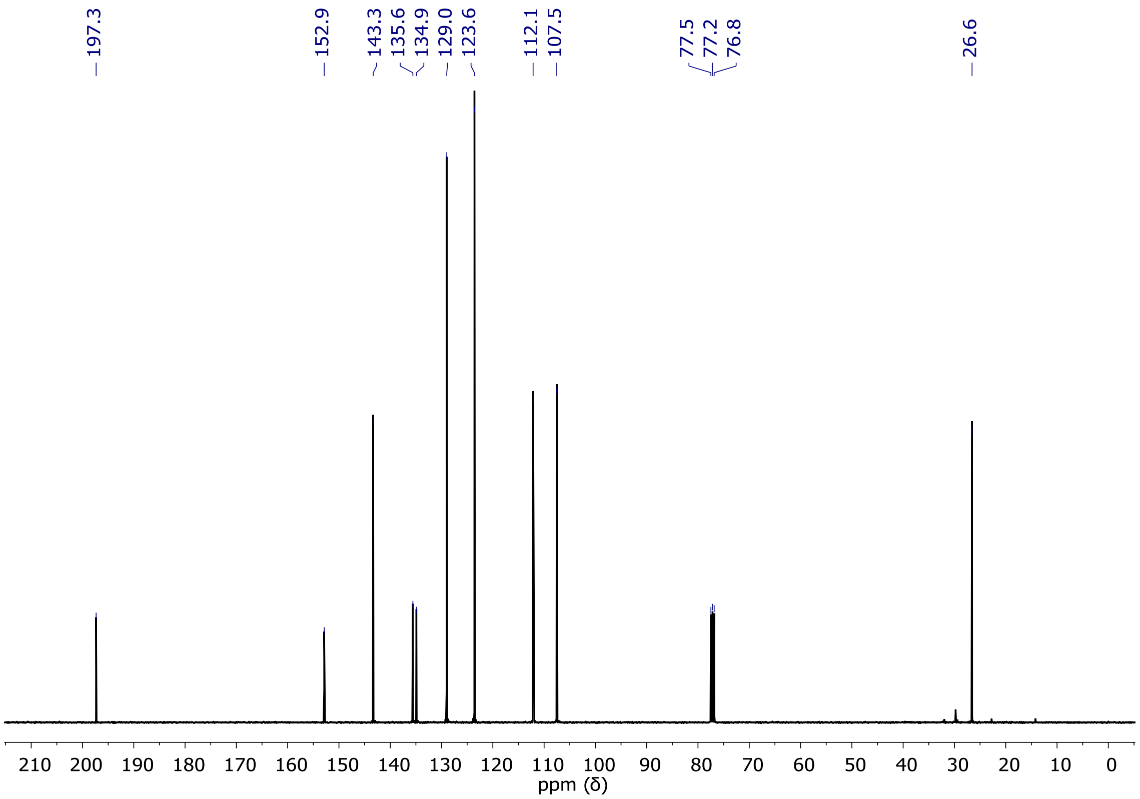

Figure S85. ^{1}H NMR Spectrum (400 MHz, $CDCl_3$) for 4-(3-thianaphthenyl) acetophenone (**32**)

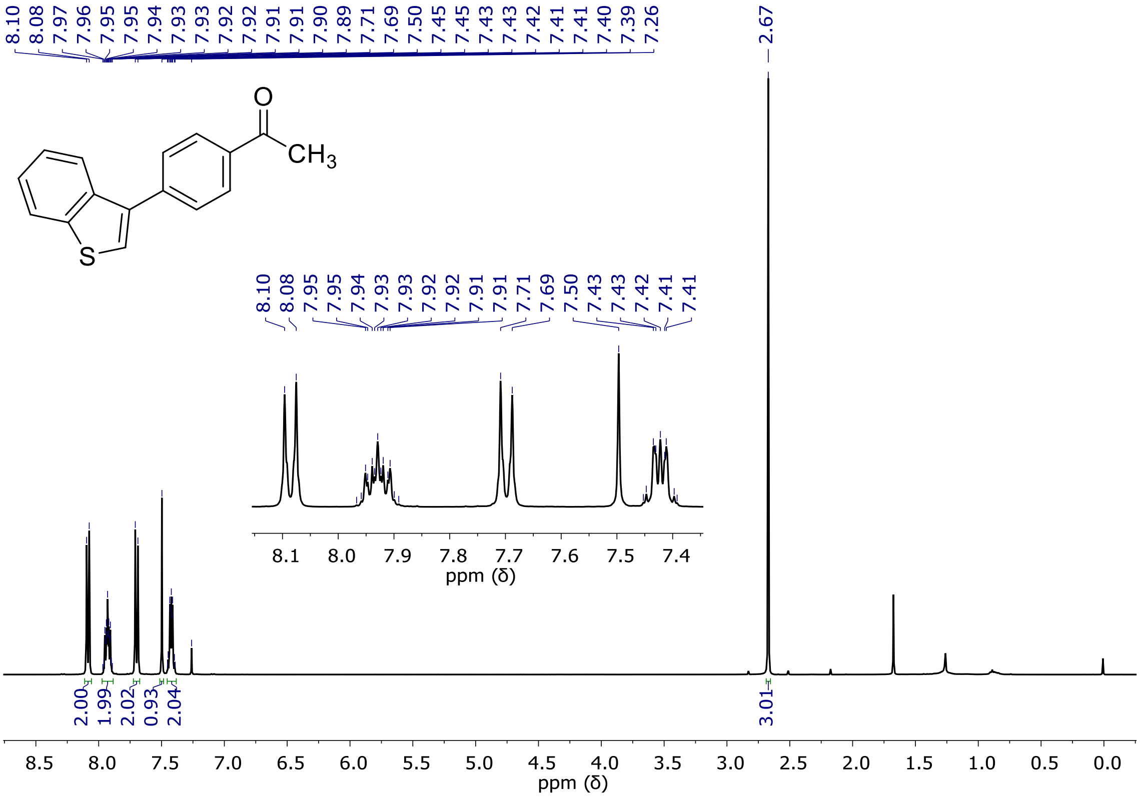

Figure S86. ^{13}C NMR Spectrum (100 MHz, $CDCl_3$) for 4-(3-thianaphthenyl) acetophenone (**32**)

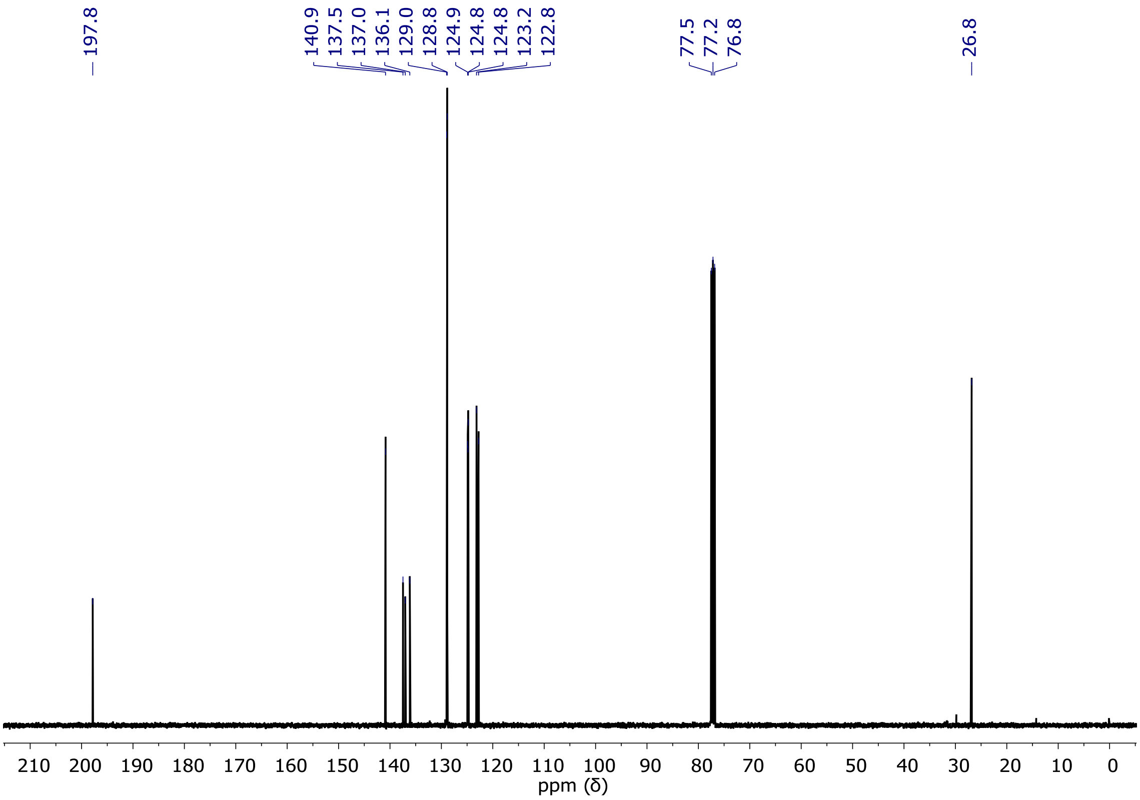

Figure S87. ^{1}H NMR Spectrum (400 MHz, $CDCl_3$) for 3-(2-furanyl)acetophenone (**33**)

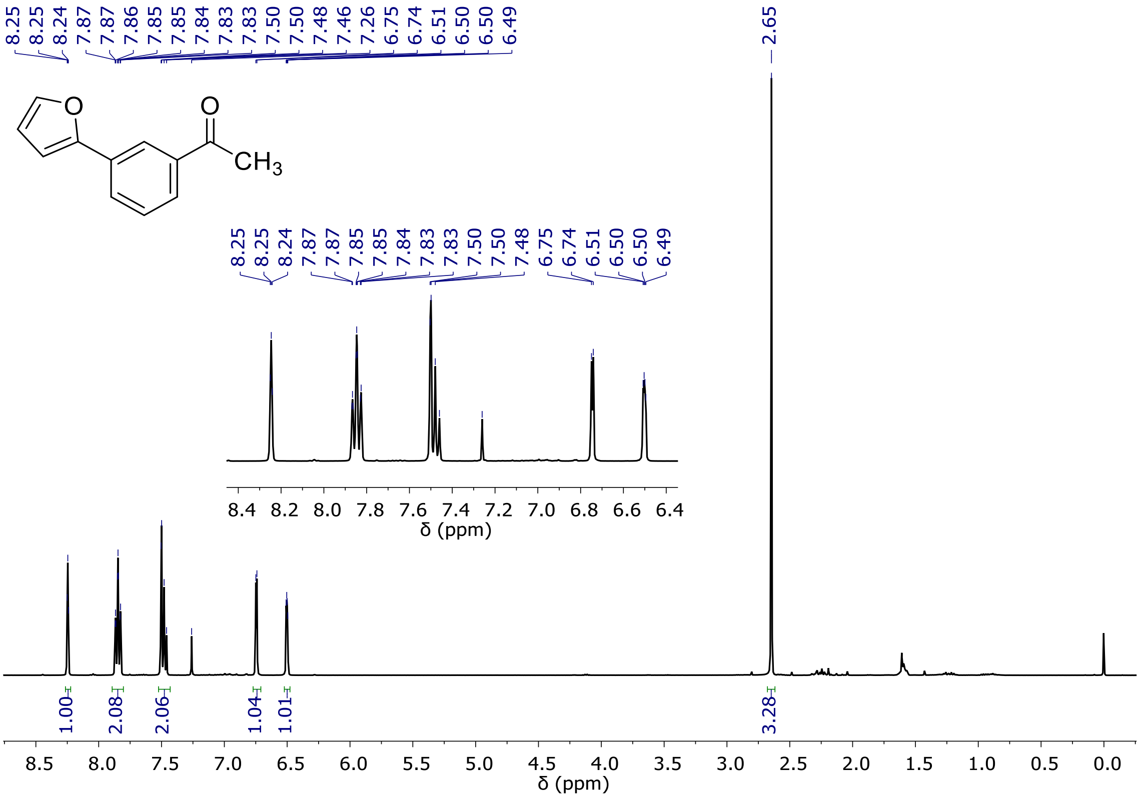

Figure S88. ^{13}C NMR Spectrum (400 MHz, $CDCl_3$) for 3-(2-furanyl)acetophenone (**33**)

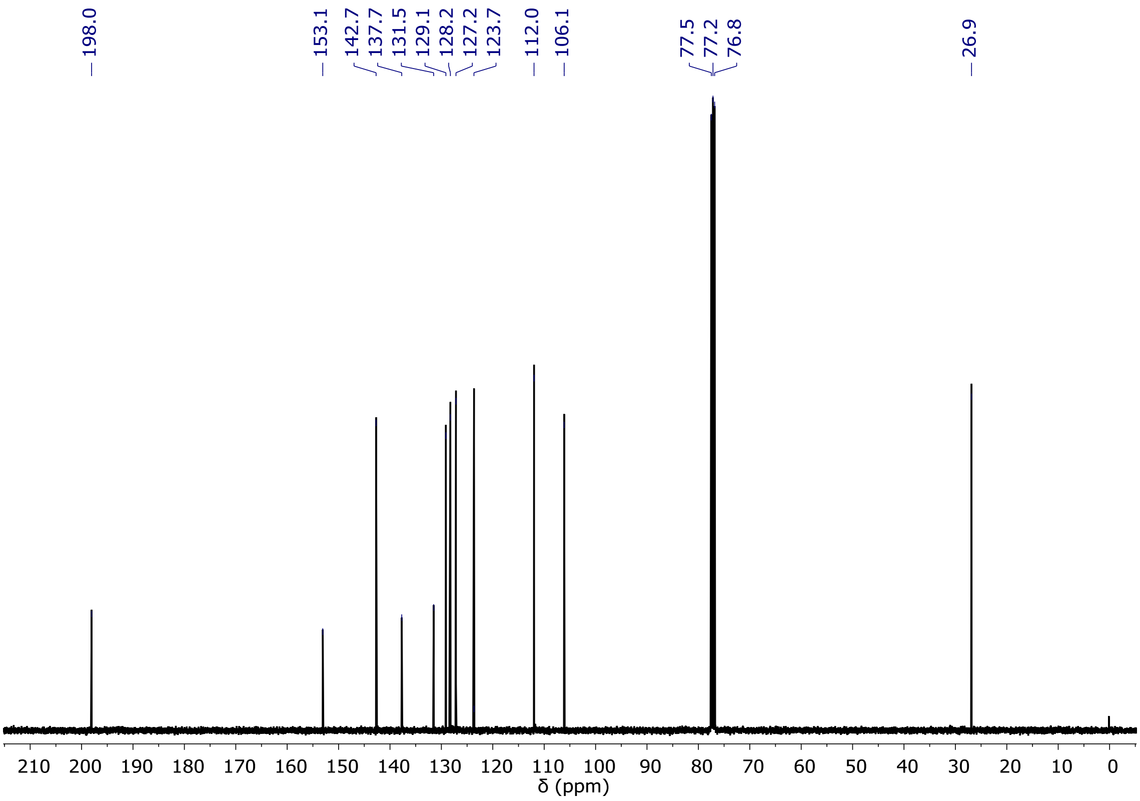

Figure S89. ^{1}H NMR Spectrum (400 MHz, $CDCl_3$) for 2,2'-bifuran*

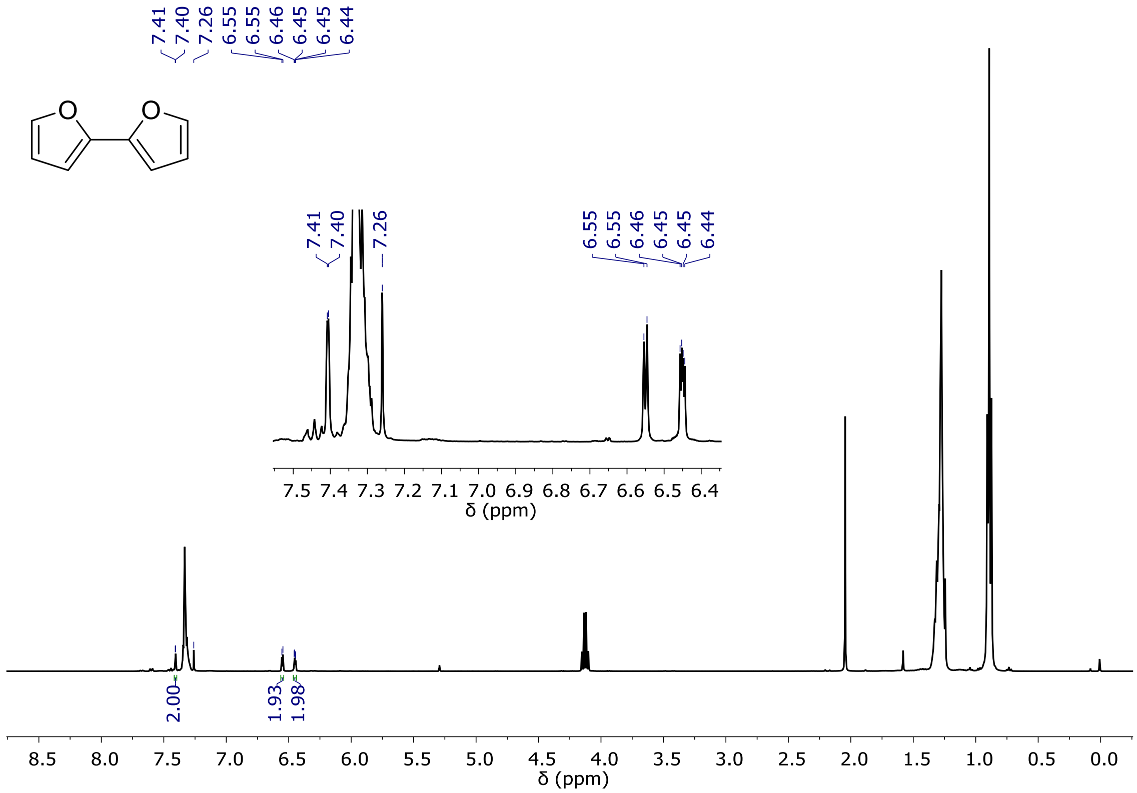

*Nb: Due to the volatility of the product, we were unable to remove solvent residues, including dichloromethane and ethyl acetate.

Figure S90. ^{1}H NMR Spectrum (400 MHz, $CDCl_3$) for 1-(3,4,5-trifluorophenyl) naphthalene (**34**)

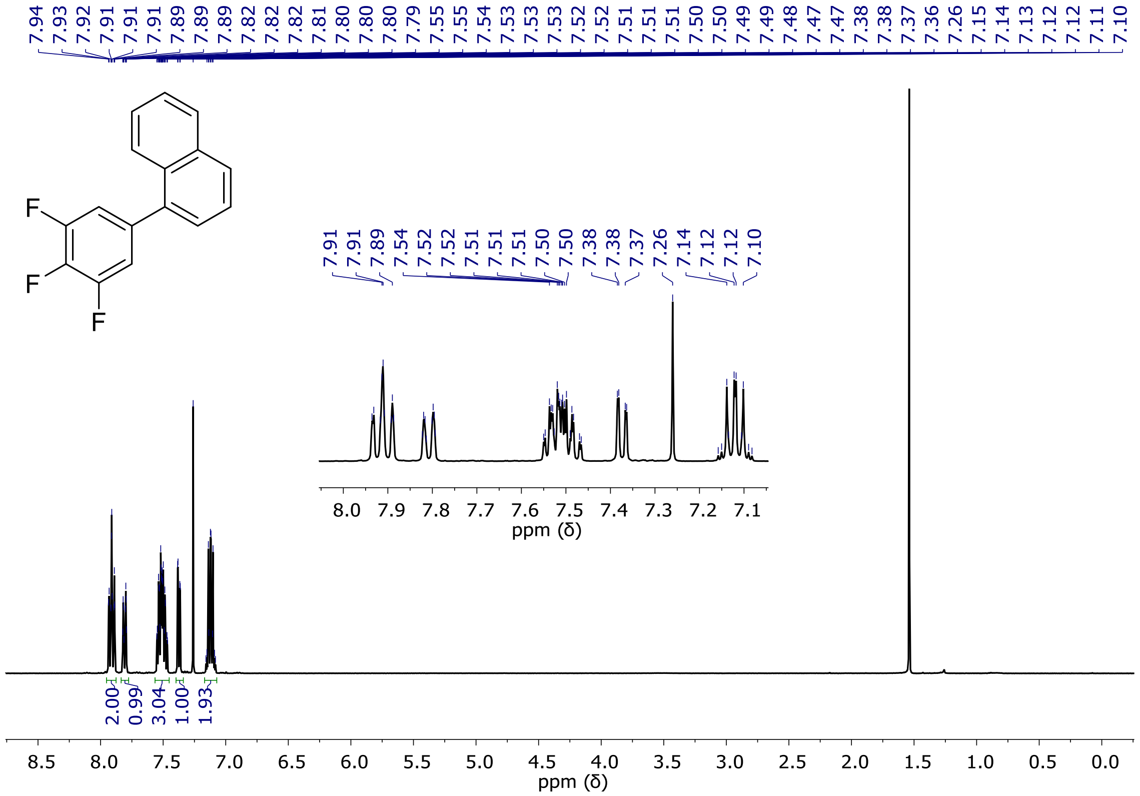

Figure S91. ^{13}C NMR Spectrum (100 MHz, $CDCl_3$) for 1-(3,4,5-trifluorophenyl) naphthalene (**34**)

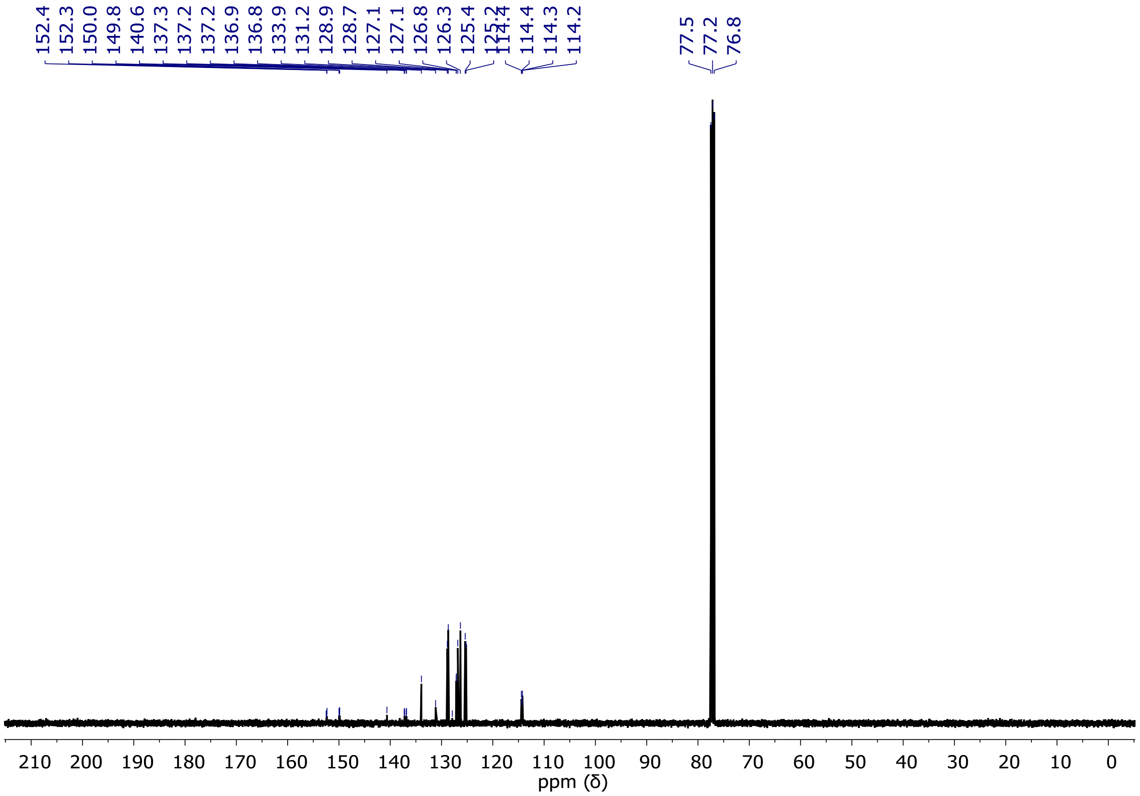

Figure S92. ^{19}F NMR Spectrum (376 MHz, $CDCl_3$) for 1-(3,4,5-trifluorophenyl) naphthalene (**34**)

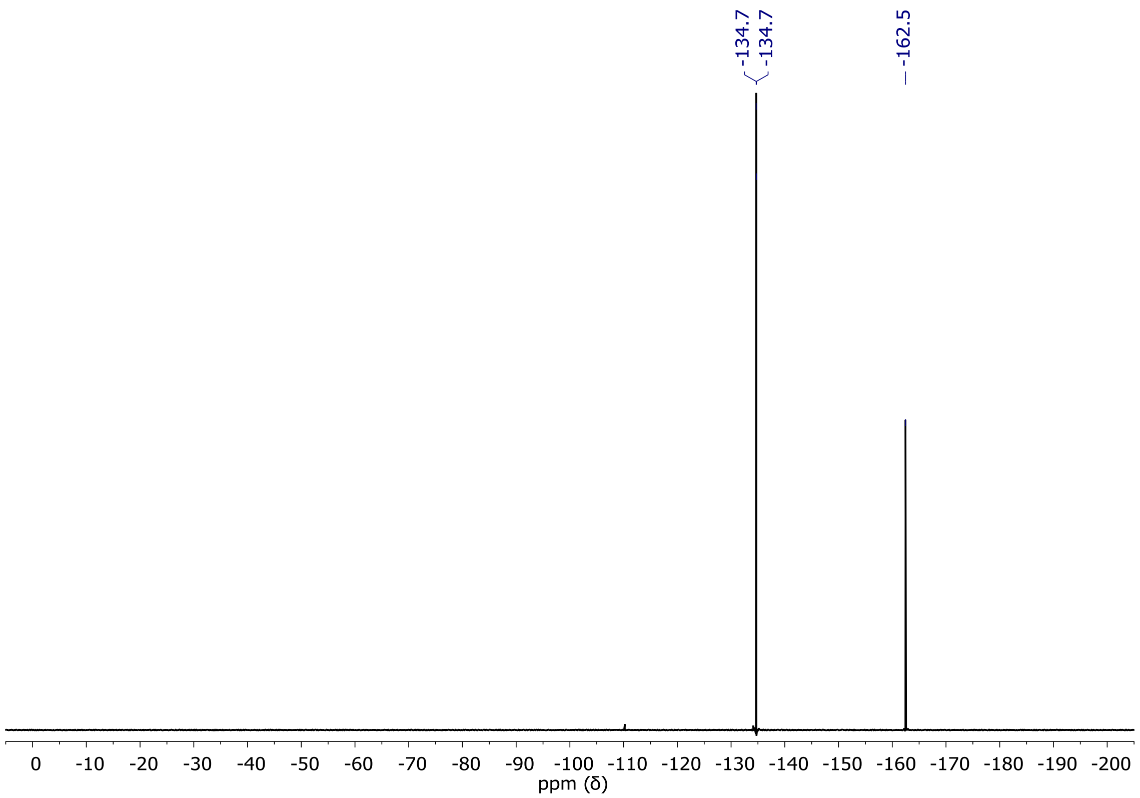

Figure S93. ^{1}H NMR Spectrum (400 MHz, $CDCl_3$) for 3-(3-thianaphthenyl)pyridine (**35**)

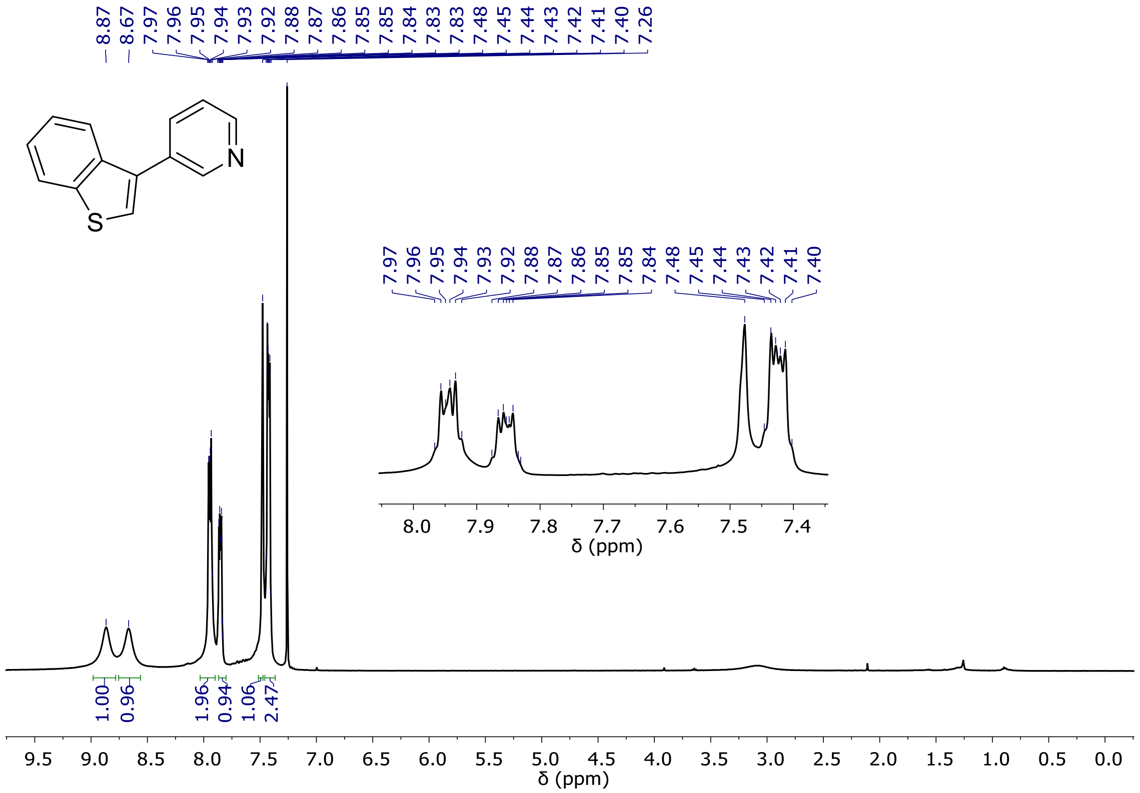

Figure S94. ^{13}C NMR Spectrum (100 MHz, $CDCl_3$) for 3-(3-thianaphthenyl)pyridine (**35**)

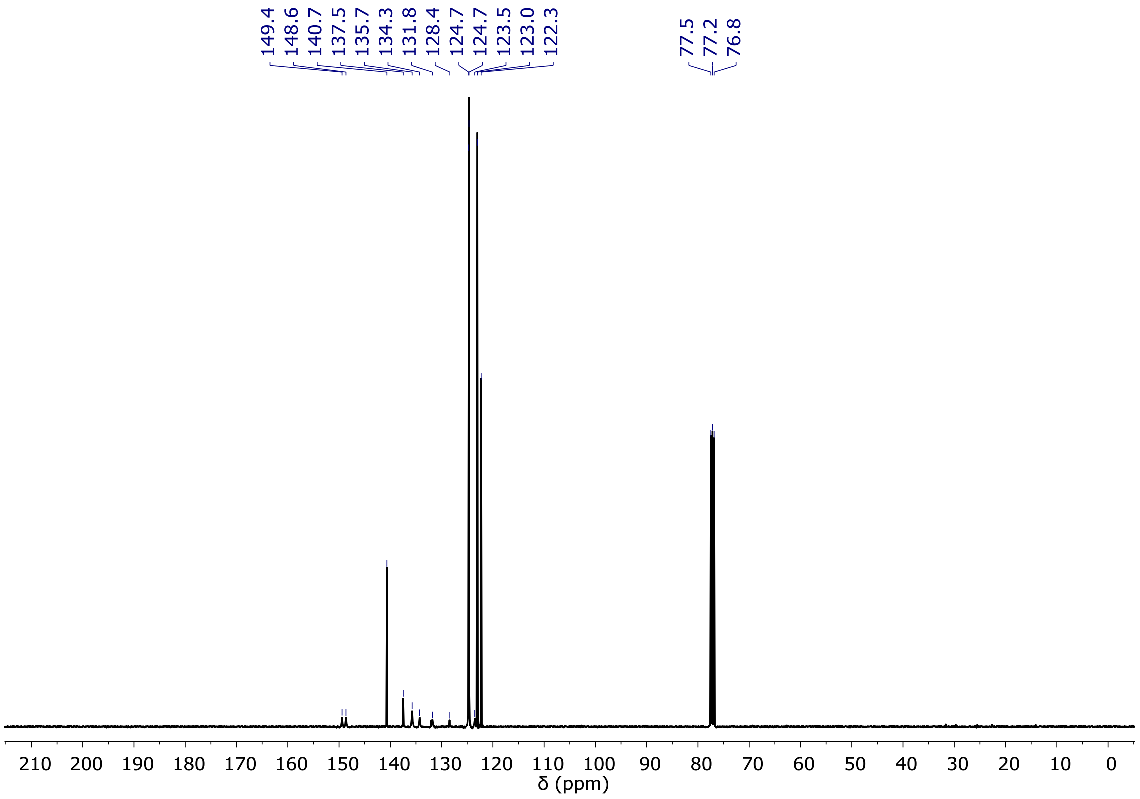

Figure S95. ^{1}H NMR Spectrum (400 MHz, $CDCl_3$) for 3-(3-fluorophenyl)isopropoxy benzene (**36**)

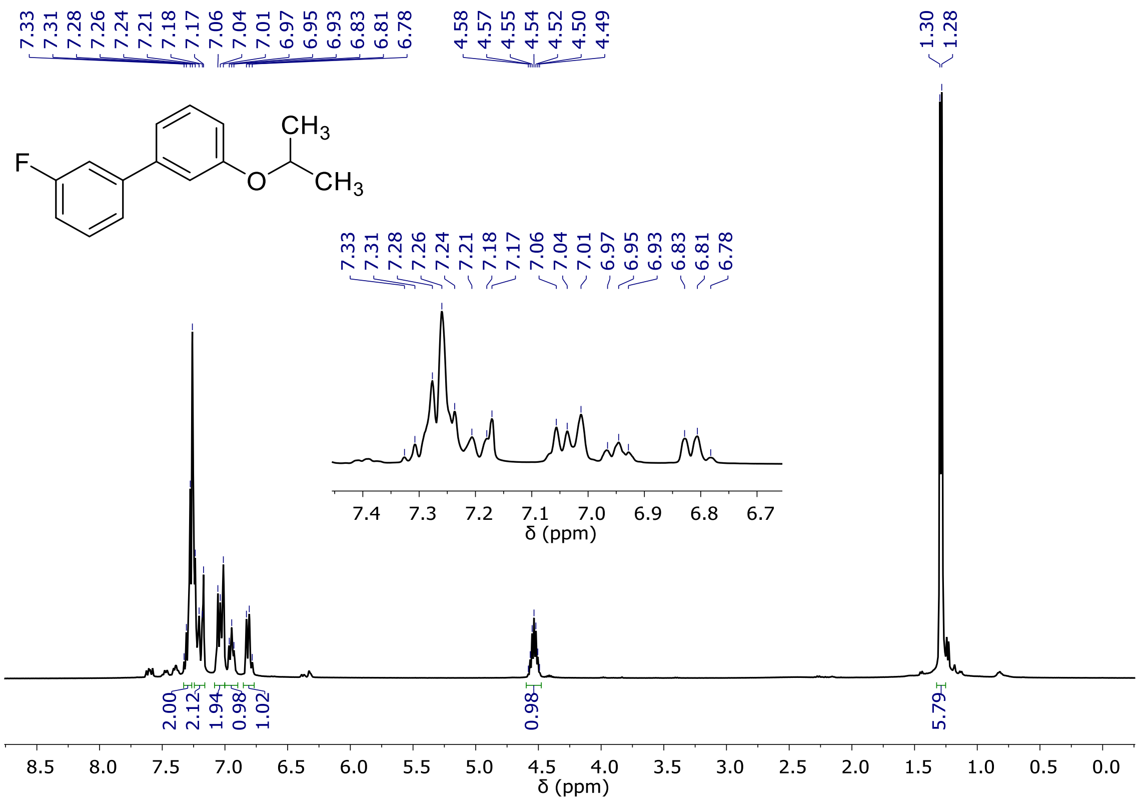

Figure S96. ^{13}C NMR Spectrum (100 MHz, $CDCl_3$) for 3-(3-fluorophenyl)isopropoxy benzene (**36**)

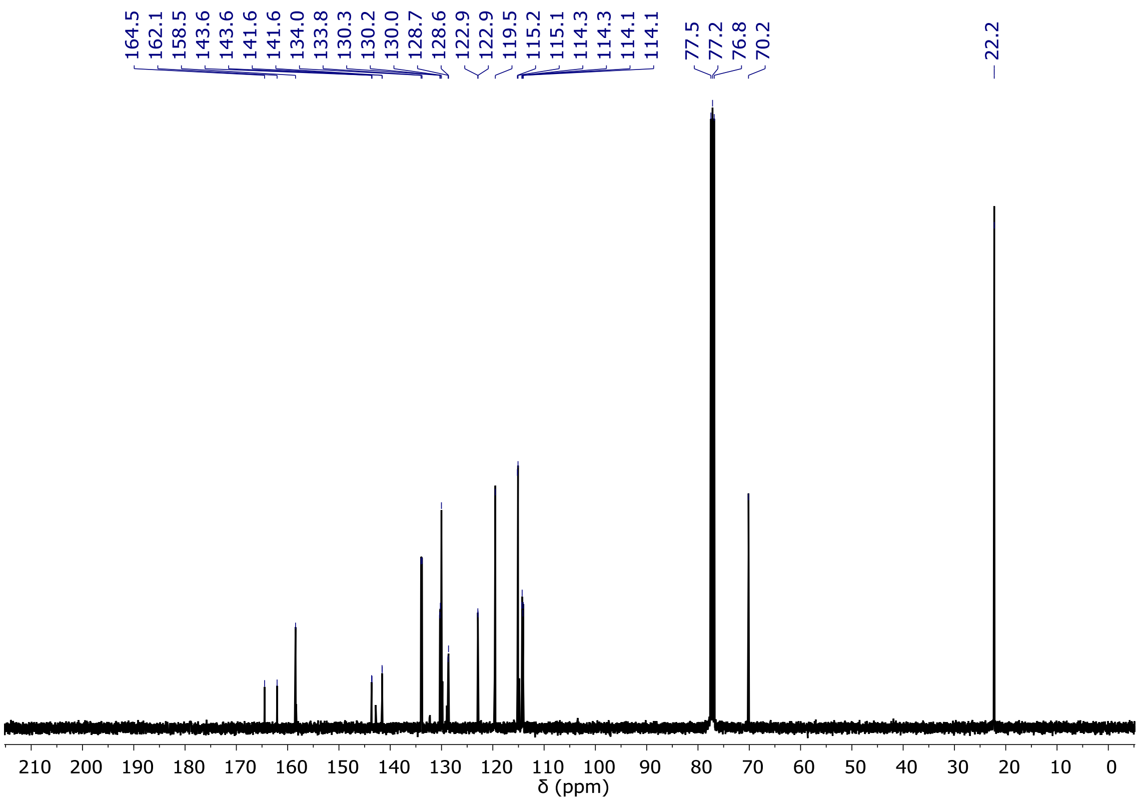

Figure S97. ^{19}F NMR Spectrum (376 MHz, $CDCl_3$) for 3-(3-fluorophenyl)isopropoxy benzene (**36**)

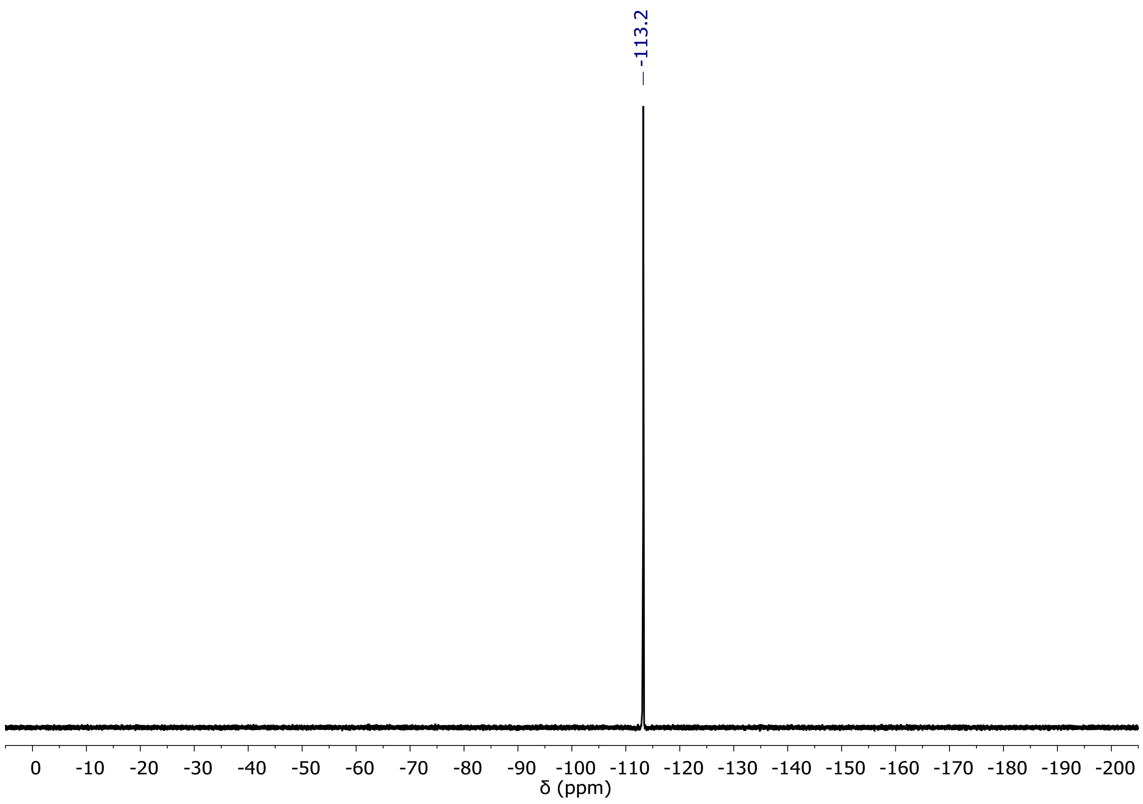

Figure S98. ^{1}H NMR Spectrum (400 MHz, $CDCl_3$) for 4-(2-naphthalenyl)benzonitrile (**37**)

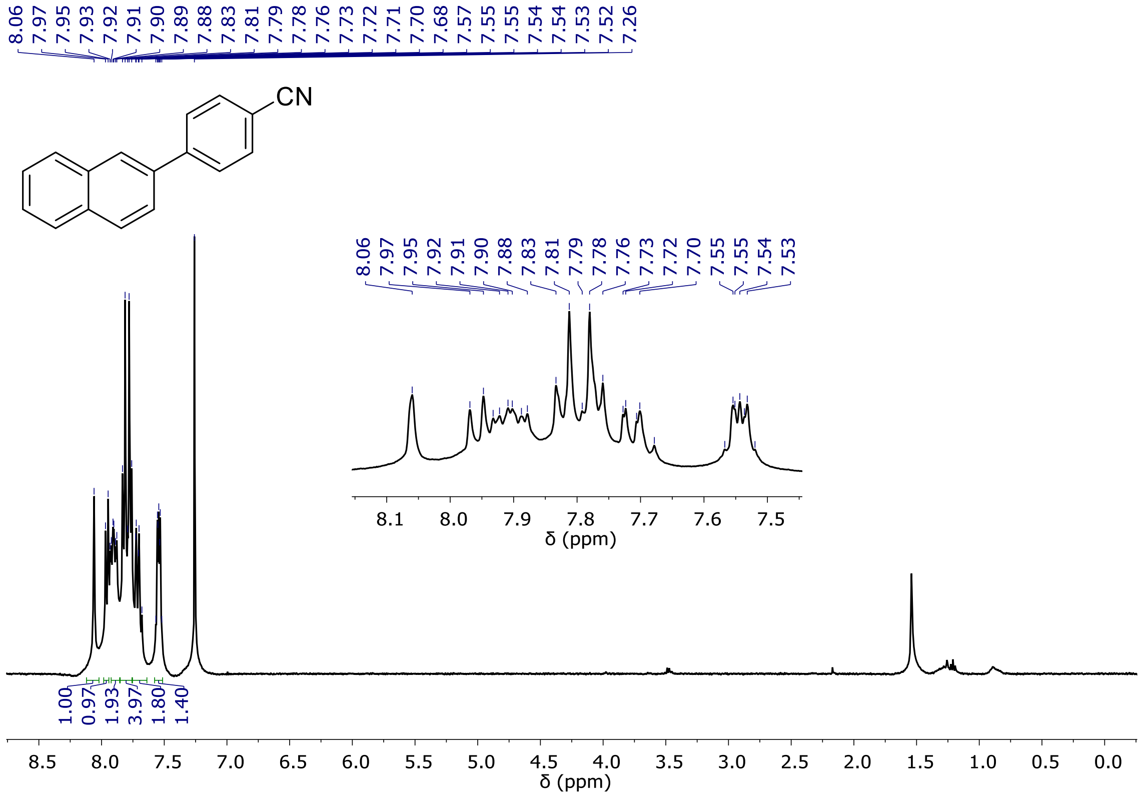

Figure S99. ^{13}C NMR Spectrum (100 MHz, $CDCl_3$) for 4-(2-naphthalenyl)benzonitrile (**37**)

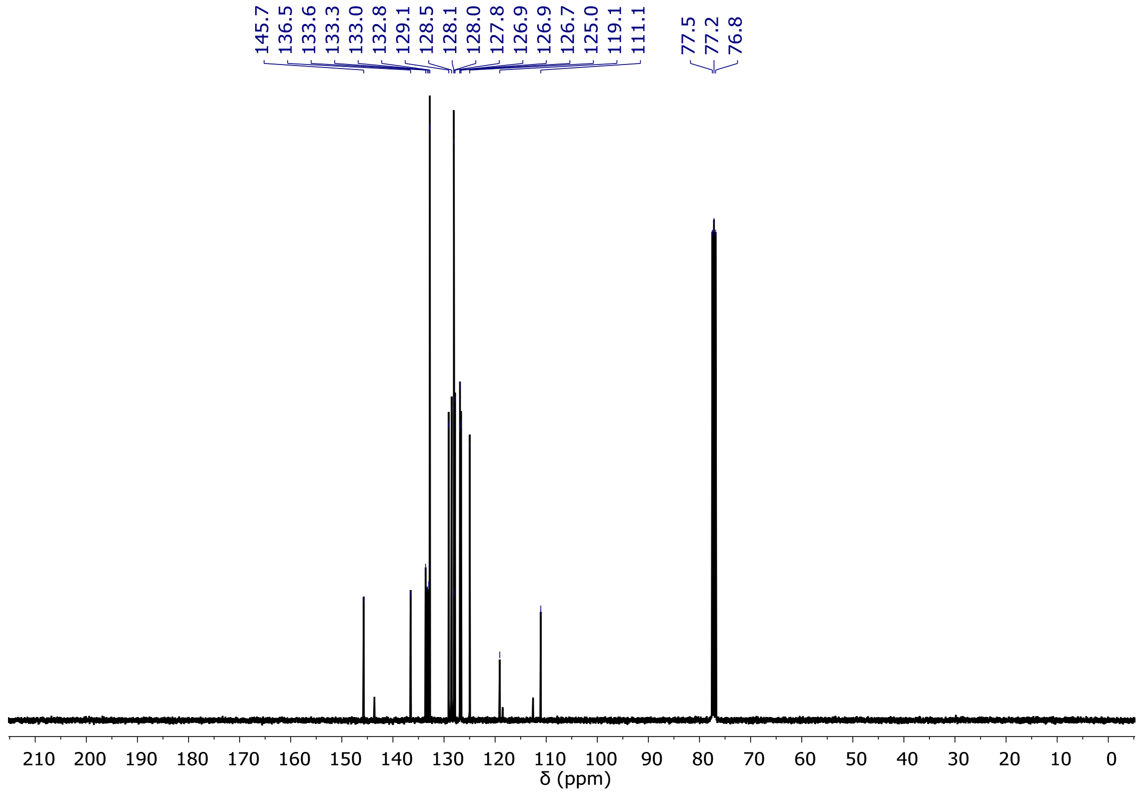

Figure S100. ^{1}H NMR Spectrum (400 MHz, $CDCl_3$) for 3-(4-methoxyphenyl)pyridine (**38**)

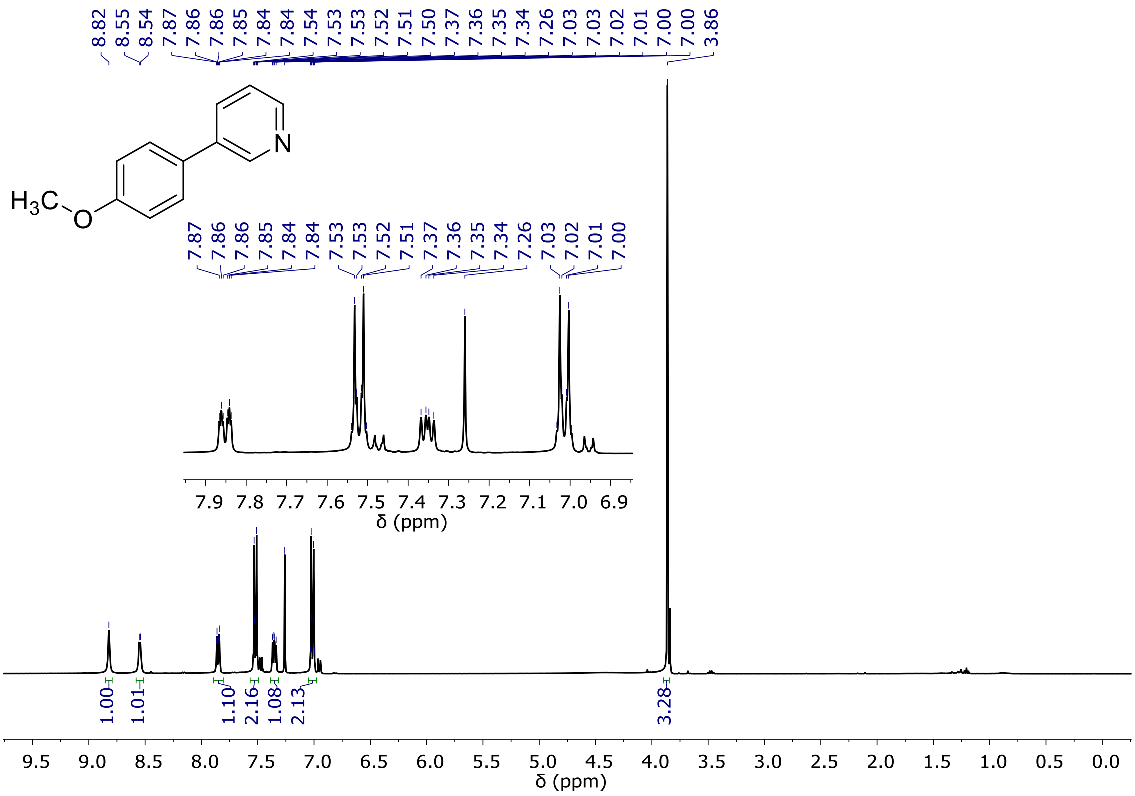

Figure S101. ^{13}C NMR Spectrum (100 MHz, $CDCl_3$) for 3-(4-methoxyphenyl)pyridine (**38**)

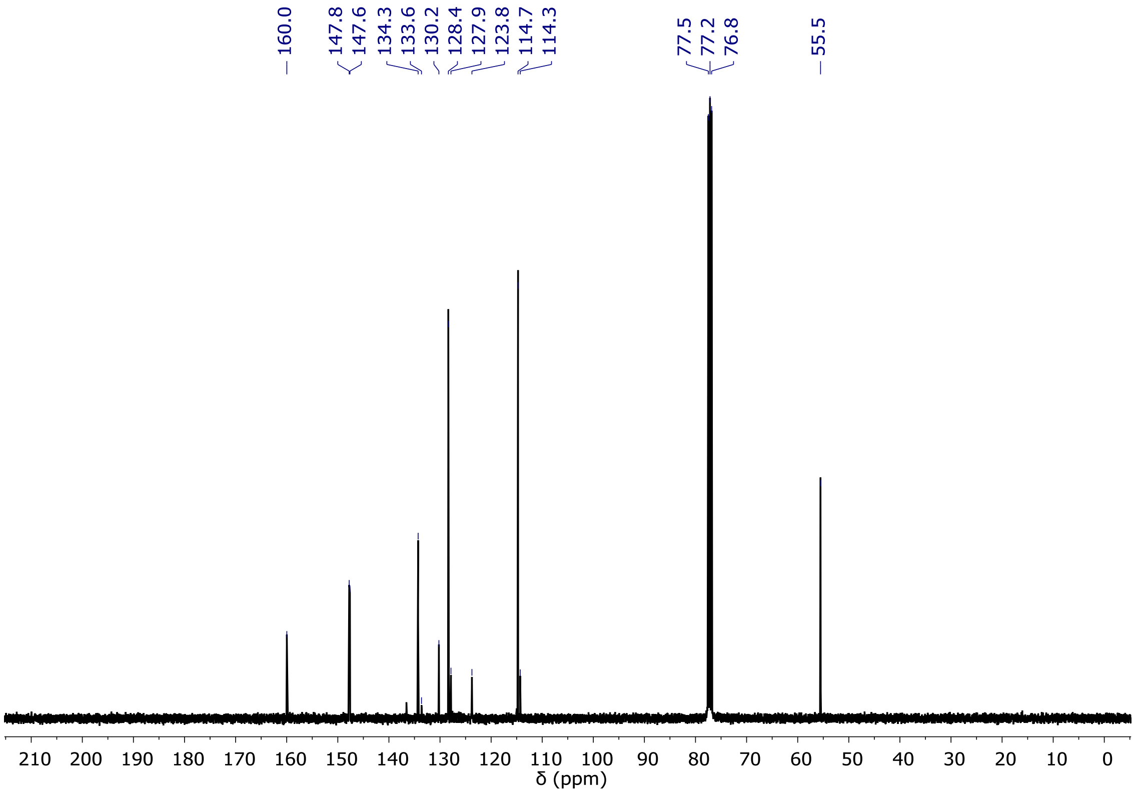

References

1 Bolt, R. R. A.; Raby-Buck, S.; Ingram, K.; Leitch, J. A.; Browne, D. L. Temperature-Controlled Mechanochemistry for the Nickel-Catalyzed Suzuki–Miyaura-Type Coupling of Aryl Sulfamates via Ball Milling and Twin-Screw Extrusion. *Angew. Chem. Int. Ed.* **2022**, *61* (44), e202210508. DOI: 10.1002/anie.202210508

2 Guan, B.-T.; Wang, Y.; Li, B.-J.; Yu, D.-G.; Shi, Z.-J. Biaryl Construction via Ni-Catalyzed C−O Activation of Phenolic Carboxylates. *J. Am. Chem. Soc.* **2008**, *130* (44), 14468–14470. DOI: 10.1021/ja8056503

3 Quasdorf, K. W.; Antoft-Finch, A.; Liu, P.; Silberstein, A. L.; Komaromi, A.; Blackburn, T.; Ramgren, S. D.; Houk, K. N.; Snieckus, V.; Garg, N. K. Suzuki−Miyaura Cross-Coupling of Aryl Carbamates and Sulfamates: Experimental and Computational Studies. *J. Am. Chem. Soc.* **2011**, *133* (16), 6352–6363. DOI: 10.1021/ja200398c

4 Patra, T.; Agasti, S.; Modaka, A.; Maiti, D. Nickel-catalyzed hydrogenolysis of unactivated carbon–cyano bonds. *Chem. Commun.* **2013**, *49* (75), 8362–8364. DOI: 10.1039/C3CC44562C

5 Yue, H.; Guo, L.; Liao, H.-H.; Cai, Y.; Zhu, C.; Rueping, M. Catalytic Ester and Amide to Amine Interconversion: Nickel-Catalyzed Decarbonylative Amination of Esters and Amides by C−O and C−C Bond Activation. *Angew. Chem. Int. Ed.* **2017**, *56* (15), 4282–4285. DOI: 10.1002/anie.201611819

6 Hoggard, L. R.; Zhang, Y.; Zhang, M.; Panic, V.; Wisniewski, J. A.; Ji, H. Rational Design of Selective Small-Molecule Inhibitors for β-Catenin/B-Cell Lymphoma 9 Protein–Protein Interactions. *J. Am. Chem. Soc.* **2015**, *137* (38), 12249–12260. DOI: 10.1021/jacs.5b04988

7 Chen, Y.-Z.; Ding, T.-H.; Li, Q.-Q.; Qu, J.-P.; Kang, Y.-B. Ambient Temperature Dehydrogenative C(Ar)–H Carbonylative Lactamization of 2-Arylanilines Using DMF as C1-Source. *Org. Lett.* **2023**, *25* (15), 2611–2615. DOI: 10.1021/acs.orglett.3c00585

8 Denmark, S. E.; Smith, R. C.; Chang, W.-T. T.; Muhuhi, J. M. Cross-Coupling Reactions of Aromatic and Heteroaromatic Silanolates with Aromatic and Heteroaromatic Halides. *J. Am. Chem. Soc.* **2009**, *131* (8), 3104–3118. DOI: 10.1021/ja8091449

9 Wang, X.; Zhang, X.; Xue, L.; Wang, Q.; You, F.; Dai, L.; Wu, J.; Kramer, S.; Lian, Z. Mechanochemical Synthesis of Aryl Fluorides by Using Ball Milling and a Piezoelectric Material as the Redox Catalyst. *Angew. Chem. Int. Ed.* **2023**, *62* (39), e202307054. DOI: 10.1002/anie.202307054

10 Jiang, W.; Huang, W.; Xu, M.; Leng, X.; Lu, L.; Shen, Q. Diimidazolium Salt HBDIM: An Easily Available, Low-Cost, CageCarbene Precursor with Broad Applications in Transition Metal-Catalyzed Reactions. *Chem. Eur. J.* **2023**, *29* (40), e202300991. DOI: 10.1002/chem.202300991

11 Ren, C.; Zenga, J.; Zou, G. Nickel-catalyzed cross-coupling of *O*,*N*-chelated diarylborinates with aryl chlorides and mesylates. *New J. Chem.* **2019**, *43* (47), 1589–1596. DOI: 10.1039/C8NJ05503C

12 Dong, Y.; Li, W.-H.; Dong, Y.-B. Dual-Metal N-Heterocyclic Carbene Complex (M = Au and Pd)-Functionalized UiO-67 MOF for Alkyne Hydration–Suzuki Coupling Tandem Reaction. *J. Org. Chem.* **2021**, *86* (2), 1818–1826. DOI: 10.1021/acs.joc.0c02641

13 Gao, K.; Lee, P.-S.; Long, C.; Yoshikai, N. Cobalt-Catalyzed *Ortho*-Arylation of Aromatic Imines with Aryl Chlorides. *Org. Lett.* **2012**, *14* (16), 4234–4237. DOI: 10.1021/ol301934y

14 More, S. A.; Kardile, R. D.; Kuo, T.-C.; Cheng, M.-J.; Liu, R.-S. Gold Catalysts Can Generate Nitrone Intermediates from a Nitrosoarene/Alkene Mixture, Enabling Two Distinct Catalytic Reactions: A Nitroso-Activated Cycloheptatriene/Benzylidene Rearrangement. *Org. Lett.* **2021**, 23 (14), 5506–5511. DOI: 10.1021/acs.orglett.1c01857

15 Amatore, M.; Gosmini, C. Efficient Cobalt-Catalyzed Formation of Unsymmetrical Biaryl Compounds and Its Application in the Synthesis of a Sartan Intermediate. *Angew. Chem. Int. Ed.* **2008**, *47* (11), 2089–2092. DOI: 10.1002/anie.200704402

16 X. Li; Y. Liu; L. Zhang; Y. Dong; Q. Liu; D. Zhang; L. Chen; Z. Zhao; H. Liu. A novel electromagnetic mill promoted mechanochemical solid-state Suzuki–Miyaura cross-coupling reaction using ultra-low catalyst loading. *Green Chem.* **2022**, *24* (15), 6026–6035. DOI: 10.1039/D2GC01427K

17 Sharma, S.; Kumar, M.; Bhalla, V. Pyrazine Derivative as Supramolecular Host for Immobilization of Palladium Nanoparticles for Efficient Suzuki Coupling. *Eur. J. Org. Chem.* **2023**, *26* (31), e202300594. DOI: 10.1002/ejoc.202300594

18 Ke, H.; Chen, X.; Zou, G. *N*-Heterocyclic Carbene-Assisted, Bis(phosphine)nickel-Catalyzed Cross-Couplings of Diarylborinic Acids with Aryl Chlorides, Tosylates, and Sulfamates. *J. Org. Chem.* **2014**, *79* (15), 7132–7140. DOI: 10.1021/jo501291y

19 Ichikawa, T.; Netsu, M.; Mizuno, M.; Mizusaki, T.; Takagi, Y.; Sawama, Y.; Monguchi, Y.; Sajiki, H. Development of a Unique Heterogeneous Palladium Catalyst for the Suzuki–Miyaura Reaction using (Hetero)aryl Chlorides and Chemoselective Hydrogenation. *Adv. Synth. Catal.* **2017**, *359* (13), 2269–2279. DOI: 10.1002/adsc.201700156

20 Edwards, G. A.; Trafford, M. A.; Hamilton, A. E.; Buxton, A. M.; Bardeaux, M. C.; Chalker, J. M. Melamine and Melamine-Formaldehyde Polymers as Ligands for Palladium and Application to Suzuki–Miyaura Cross-Coupling Reactions in Sustainable Solvents. *J. Org. Chem.* **2014**, *79* (5), 2094–2104. DOI: 10.1021/jo402799t

21 Lu, Z.; Jasinski, J. B.; Handa, S.; Hammond, G. B. Recyclable cellulose-palladium nanoparticles for clean cross-coupling chemistry. *Org. Biomol. Chem.* **2018**, *16* (15), 2748–2752. DOI: 10.1039/C8OB00527C

22 Janaagal, A.; Sanyam; Mondal, A.; Gupta, I. Robust Zinc(II)porphyrin Catalyst for Visible Light Induced C–H Arylation of Heteroarenes. *J. Org. Chem.* **2023**, *88* (13), 9424–9431. DOI: 10.1021/acs.joc.3c00385

23 Albano, G.; Decandia, G.; Capozzi, M. A. M.; Zappimbulso, N.; Punzi, A.; Farinola, G. M. Infrared Irradiation-Assisted Solvent-Free Pd-Catalyzed (Hetero)aryl-aryl Coupling via C−H Bond Activation. *ChemSusChem* **2021**, *14* (16), 3391–3401. DOI: 10.1002/cssc.202101070

24 Wang, L.; Shen, J.; Yang, S.; Liu, W.; Chen, Q.; He, M. C–H arylation reactions through aniline activation catalysed by a PANI-g-C_3N_4-TiO_2 composite under visible light in aqueous medium. *Green Chem.* **2018**, *20* (6), 1290–1296. DOI: 10.1039/C8GC00012C

25 Zhen, S.; Lu, B.; Xu, J.; Zhanga, S.; Li, Y. Poly(mono-, bi- or trifuran): effect of oligomer chain length on the electropolymerization performances and polymer properties. *RSC Adv.* **2014**, *4* (27), 14001–14012. DOI: 10.1039/C4RA00437J

26 Surgenor, R. R.; Lee, H. Synthesis of (Hetero)biaryls via Nickel Catalyzed Reductive Cross-Electrophile Coupling Between (Hetero)aryl Iodides and Bromides. *Chem. Eur. J.* **2024**, *30* (44), e202401552. DOI: 10.1002/chem.202401552

27 Fu, Y.; Guo, L.-L.; Chen, X.; Chen, H.; Liu, J.-J.; Li, F.-R.; Xiao, C.-Q.; Du, Z. Pd/Cu Co-Catalyzed *ortho*-Arylation of Aryl Iodides *via* Radical Intermediates with Aryl Diazonium Salts. *Adv. Synth. Catal.* **2023**, *365* (23), 4248–4255. DOI: 10.1002/adsc.202300883

28 Kitahara, M.; Umeda, N.; Hirano, K.; Satoh, T.; Miura, M. Copper-Mediated Intermolecular Direct Biaryl Coupling. *J. Am. Chem. Soc.* **2011**, *133* (7), 2160–2162. DOI: 10.1021/ja111401h
